# Calcium Bistriflimide-Mediated Sulfur(VI)–Fluoride Exchange (SuFEx): Mechanistic Insights toward Instigating Catalysis

**DOI:** 10.1021/acs.inorgchem.2c01230

**Published:** 2022-06-14

**Authors:** Brian Han, Samuel R. Khasnavis, Matthew Nwerem, Michael Bertagna, Nicholas D. Ball, O. Maduka Ogba

**Affiliations:** † Chemistry and Biochemistry Program, Schmid College of Science and Technology, Chapman University, One University Drive, Orange, California 92866, United States; ‡ Department of Chemistry, Pomona College, 645 North College Avenue, Claremont, California 91711, United States

## Abstract

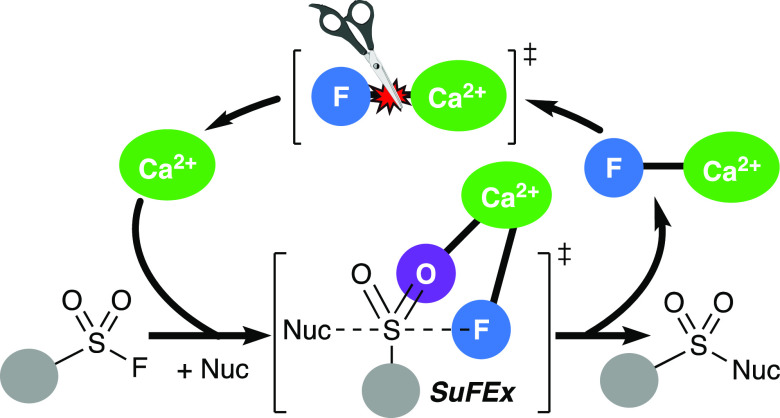

We report a mechanistic investigation of calcium bistriflimide-mediated sulfur(VI)–fluoride exchange (SuFEx) between sulfonyl fluorides and amines. We determine the likely pre-activation resting state—a calcium bistriflimide complex with ligated amines—thus allowing for corroborated calculation of the SuFEx activation barrier at ∼21 kcal/mol, compared to 21.5 ± 0.14 kcal/mol derived via kinetics experiments. Transition state analysis revealed: (1) a two-point calcium-substrate contact that activates the sulfur(VI) center and stabilizes the leaving fluoride and (2) a 1,4-diazabicyclo[2.2.2]octane additive that provides Brønsted-base activation of the nucleophilic amine. Stable Ca–F complexes upon sulfonamide formation are likely contributors to inhibited catalytic turnover, and a proof-of-principle redesign provided evidence that sulfonamide formation is feasible with 10 mol % calcium bistriflimide.

## Abstract 2

We report a computational and experimental mechanistic study of sulfur(VI)-fluoride exchange mediated by a calcium salt. Ca^2+^ activates the substrate via two critical Lewis acid−base interactions. Stable fluoride-ligated Ca^2+^ complexes are formed upon sulfonamide formation, and computations indicate that heat and/or fluoride scavengers will facilitate regeneration of the active Ca^2+^ species. These calculations guided an experimental redesign, thus demonstrating improved catalytic efficiency using 10 mol % Ca^2+^.

## Introduction

**Figure 1:**
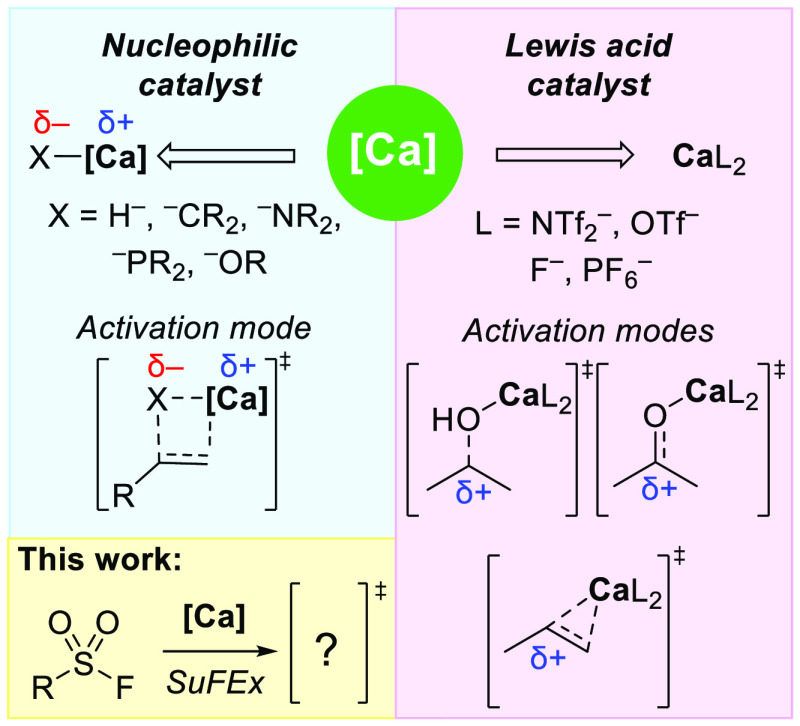
Ca^2+^ salts can serve as nucleophilic and Lewis acid catalysts. The activation modes for known substrates are shown. The work reported herein investigates Ca^2+^ activation for sulfur(VI) fluoride substrates in SuFEx reactions.

Calcium (Ca^2+^) salts have gained significant attention in the last two decades as catalysts in a wide variety of chemical transformations.^1,2^ Use of this early main group metal is desirable because calcium is significantly cheaper, more abundant, and more sustainable than typical transition metals utilized for modern homogenous catalysis.^3^ Our current understanding of how calcium salts activate substrates and facilitate chemical reactions is based on two fundamental electronic properties between the Ca^2+^ center and the corresponding anion (Figure 1). First, like alkali metal ions, Ca^2+^ is involved in an ionic interaction with a coordinating anion whereby the anion maintains its charge and nucleophilicity. This feature has been harnessed to engender carbanion, amide, and hydride nucleophiles for styrene polymerizations,^4−8^ olefin hydroaminations,^9−12^ and carbonyl reductions.^13^ Second, like group 3 compounds, Ca^2+^ is a strong Lewis acid, especially when coordinated to weakly binding ligands such as fluorides (F^–^), triflates (OTf^–^), bistriflimides (NTf_2_^–^), or 1:1 bistriflimide/hexafluorophosphate (PF_6_^–^) counterions. These Ca^2+^ salts form Lewis acid/base adducts to activate otherwise weakly electrophilic compounds such as alcohols,^14−19^ carbonyls,^20−27^ olefins,^28−30^ and boronic acids^31^ for subsequent coupling with nucleophilic reagents, notably also with relatively high tolerance to air and moisture. In our recent reports, we employed Ca^2+^ salts for the first time to activate a different class of compounds—sulfur(VI) fluorides.^32,33^

**Figure 2:**
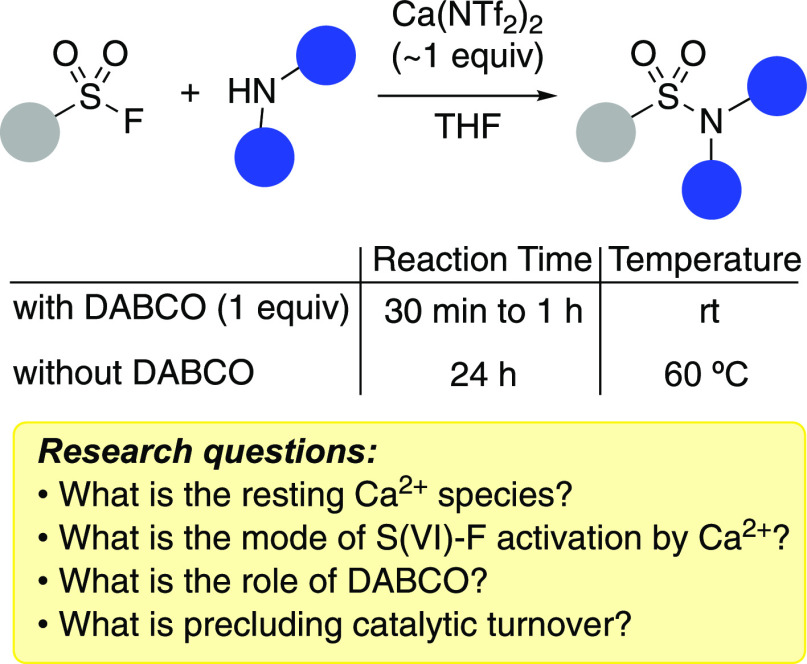
Ca(NTf_2_)_2_ and DABCO-mediated SuFEx between sulfur(VI) fluorides and amines.

Sulfur(VI) fluorides are an emerging class of compounds with various applications from materials to drug targets.^34,35^ Their stability to hydrolysis, redox chemistry, and decomposition compared to other sulfur(VI) halides has made sulfur(VI) fluorides an attractive functional group in synthesis.^36−38^ Sulfur(VI)–fluoride exchange (SuFEx) has served as an important strategy for click chemistry applications,^39^ especially in their development as selective covalent enzyme inhibitors^40−42^ in drug discovery and chemical cross-linking strategies.^43^ Recently, we developed a Ca(NTf_2_)_2_-mediated method to activate sulfur(VI) fluorides toward the formation of nitrogen-containing sulfur(VI) compounds,^32,33^ representing a new SuFEx approach using metal Lewis acids and a departure from hydrogen-bond or nucleophilic activation of the sulfur center.^35^ The first report^33^ demonstrated that a myriad of sulfonyl fluorides could be activated by Ca(NTf_2_)_2_ in the presence of amines, resulting in sulfonamides. After 24 h at 60 °C in *t*-amyl alcohol, sulfonamides are formed in good to excellent yields. The follow-up report^32^ demonstrated that addition of 1,4-diazabicyclo[2.2.2]octane (DABCO) and using tetrahydrofuran (THF) as a solvent enabled broad activation of diverse sulfur(VI) fluorides under significantly milder conditions (e.g., at room temperature, Figure 2). This work was the first to apply calcium salts in sulfur and fluorine chemistry. However, in contrast to many examples in the literature of catalytic transformations with Ca(NTf_2_)_2_, stoichiometric amounts were required for efficient SuFEx. Lower equivalents of Ca^2+^ resulted in the poor conversion of sulfur(VI) fluoride to the desired product; thereby, catalytic turnover has remained elusive.

In our goal to improve the efficiency of this reaction, we employed computational techniques to elucidate plausible mechanisms for Ca(NTf_2_)_2_-mediated sulfur(VI) fluoride activation to uncover the mode of Ca^2+^ activation of sulfur(VI) fluorides and to explore origins of catalytic turnover inhibition (Figure 2). We performed a systematic exploration of solvent and ligand coordination to identify the likely pre-activation resting state of the calcium salt, which then provided a baseline for establishing the SuFEx activation barrier. In the SuFEx transition state, we observed a two-point interaction between Ca^2+^ and the sulfonyl fluoride, which activates the sulfur(VI) and stabilizes the fluoride leaving group. We investigated the role of DABCO in facilitating SuFEx and discussed how stable Ca–F complexes formed upon sulfur(VI) fluoride activation are likely contributors to the inhibited catalytic turnover. These mechanistic insights led to a proof-of-principle redesign, demonstrating catalytic turnover of Ca(NTf_2_)_2_. This report represents the first systematic study of Ca^2+^ activation of organosulfur and fluorinated compounds and thus a foundational platform for understanding future catalysis with Ca^2+^ Lewis acids and Lewis acid-activation of organic sulfur-fluorides.

## Experimental Section

### Computational Details

Conformational searches at each stationary point on the computed potential energy surface were performed in *Schrodinger Macromodel*(44) using the optimized polarizable liquid simulation (OPLS) molecular mechanics force field.^45^ While several hundred conformers were generated for each state, many relaxed into redundant geometries upon quantum mechanical treatment. Quantum mechanical geometry optimizations, vibrational frequencies, and thermochemical values reported in this paper were carried out in the gas phase under the B3LYP^46,47^/6-31G(d,p)^48−51^ level of theory; electronic energies on the optimized geometries were performed using the dispersion-corrected ωB97XD^52,53^ functional and the triple-zeta def2-TZVP^54−56^ basis set, incorporating the polarized continuum model^57,58^ for the THF solvent. Structures at the ground and transition states along the reaction coordinate were verified by analyzing vibrational frequencies. All thermochemical energies were calculated at 298 K and 1 atm and reported in kcal/mol units. All quantum mechanical calculations were carried out in *Gaussian 16, Revision B.01*.^59^

### General Experimental Methods

All commercially available chemicals, reagents, and solvents were used as received. Reagents were purchased from Sigma Aldrich, Enamine, Matrix Scientific, and TCI America. Reactions were monitored by thin-layer chromatography (TLC) performed on Merck silica gel plates (60 F254) (80:20 hexanes: ethyl acetate mobile phase) and were visualized with ultraviolet (UV) light (254 nm). Proton nuclear magnetic resonance (^1^H NMR) spectra, carbon nuclear magnetic resonance (^13^C NMR) spectra, and fluorine nuclear magnetic resonance (^19^F NMR) spectra were recorded on a Bruker 400 (400.00, 100.61, and 376.50 MHz, respectively) equipped with cryoprobes using the Bruker Topspin 1.3 software. Chemical shifts are reported in parts per million (ppm) relative to chloroform (^1^H δ = 7.26 and ^13^C δ = 77.16). The NMR peak multiplicities were reported as follows: singlet (s), doublet (d), triplet (t), and quartet (q). High-resolution mass spectra (HRMS) were acquired on an Agilent model 6220 MS(TOF). Column chromatography was performed on a Teledyne ISCO Combi*Flash* NextGen 300 system using a pre-packed 25 g 60 Å silica column. Aluminum heating blocks were used for reactions that required elevated temperatures.

### General Procedure for Synthesizing and Isolating Sulfonamides **21–24**

The procedure used to synthesize and isolate sulfonamides was adapted from the one previously reported by our group.^32^ To a 3.7 mL (1 dram) scintillating glass vial, the amine substrate (1.05 equiv), Ca(NTf_2_)_2_ (0.1 equiv), DABCO (0.2 or 0.5 equiv), and 1,1,3,3-tetramethyldisiloxane (TMDS) (2.0 equiv.) were added and dissolved in anhydrous THF (0.25 M). Sulfonyl fluoride (1.0 equiv) was added to the reaction mixture, and the reaction was stirred for 24 h at 50 °C. The reaction mixture was diluted with 20 mL of ethyl acetate, and the organic layer was washed once with saturated NH_4_Cl and then with saturated brine. The organic layer was dried over anhydrous MgSO_4_ or Na_2_SO_4_, concentrated under reduced pressure, and then loaded onto a flash chromatography column (silica gel cartridge for dry loading, EtOAc/hexane mobile phase). The product fractions were identified by TLC, concentrated under reduced pressure, and dried under high vacuum to yield product.

#### 1-(Phenylsulfonyl)-4-(6-(trifluoromethyl)pyridin-2-yl)piperazine **21**

The reaction was performed using the general procedure with commercially available **1** (32.0 mg, 24 μL, 0.200 mmol, 1 equiv), **3** (48.6 mg, 0.210 mmol, 1.05 equiv), Ca(NTf_2_)_2_ (12.0 mg, 0.020 mmol, 0.1 equiv), DABCO (4.5 mg, 0.040 mmol, 0.2 equiv), and TMDS (53.7 mg, 71 μL, 0.400 mmol, 2 equiv) in THF (0.80 mL) for 24 h with stirring at 50 °C. The reaction was run in duplicate. Purification of the duplicate samples by column chromatography using dry loading gave the product (55 mg, 0.149 mmol, 75% yield) as a white solid. A control reaction, without Ca(NTf_2_)_2_, was also performed, but no product was found in the CombiFlash fraction vials by TLC. The ^1^H NMR, ^13^C NMR, and ^19^F NMR spectra were consistent with those previously reported.^32^^1^H NMR (CDCl_3_, 400 MHz): δ 7.80–7.75 (m, 2H), 7.63–7.51 (m, 4H), 6.95 (d, *J* = 7.2 Hz, 1H), 6.72 (d, *J* = 8.4 Hz, 1H), 3.74–3.68 (m, 4H), 3.15–3.09 (m, 4H). ^13^C NMR (CDCl_3_, 101 MHz): δ 158.1, 146.5 (q, *J*_CF_ = 34.1 Hz), 138.8, 135.4, 133.2, 129.3, 127.9, 121.5 (q, *J*_CF_ = 274.0 Hz), 109.8 (q, *J*_CF_ = 3.1 Hz), 109.6 (d, *J*_CF_ = 1.1 Hz), 45.9, 44.3. ^19^F NMR (CDCl_3_, 376 MHz): δ −68.2. HRMS (TOF+) *m*/*z*: [M^+^] Calcd for C_16_H_16_N_3_O_2_F_3_S, 371.09098; found, 371.09232.

#### 1-((4-Methoxyphenyl)sulfonyl)-4-(6-(trifluoromethyl)pyridin-2-yl)piperazine **22**

The reaction was performed using the general procedure with commercially available 4-methoxybenzenesulfonyl fluoride (38 mg, 28.4 μL, 0.200 mmol, 1 equiv), **3** (48.6 mg, 0.210 mmol, 1.05 equiv), Ca(NTf_2_)_2_ (12.0 mg, 0.020 mmol, 0.1 equiv), DABCO (4.5 mg, 0.040 mmol, 0.2 equiv), and TMDS (53.7 mg, 71 μL, 0.400 mmol, 2 equiv) in THF (0.80 mL) for 24 h with stirring at 50 °C. Purification of the duplicate samples by column chromatography using solid loading gave the product (41 mg, 0.102 mmol, 51% yield) as white crystals. A control reaction, without Ca(NTf_2_)_2_, was also performed, and the product (5 mg, 0.012 mmol, 7% yield) was isolated in the same manner. The ^1^H NMR, ^13^C NMR, and ^19^F NMR spectra were consistent with those previously reported.^32^^1^H NMR (CDCl_3_, 400 MHz): δ 7.72–7.69 (m, 2H), 7.57 (t, *J* = 8.0 Hz, 1H), 7.01–6.96 (m, 3H), 6.75 (d, *J* = 8.0 Hz, 1H), 3.86 (s, 3H), 3.72–3.70 (m, 4H), 3.12–3.09 (m, 4H). ^13^C NMR (CDCl_3_, 101 MHz): δ163.4, 158.1, 146.6 (q, JCF = 34.34 Hz), 138.8, 130.1, 126.9, 121.6 (q, JCF = 275.1 Hz), 114.5, 109.8, 109.7, 55.8, 45.9, 44.4. ^19^F NMR (CDCl_3_, 376 MHz): δ −68.17. HRMS (TOF+) *m*/*z*: [M+] Calcd for C_17_H_18_N_3_O_3_F_3_S, 401.100155; found, 401.10328.

#### 1-(Phenylsulfonyl)-4-(5-(trifluoromethyl)pyridin-2-yl)piperazine **23**

The reaction was performed using the general procedure except washes were performed with 1 M HCl and saturated brine. Commercially available **1** (32.0 mg, 24 μL, 0.200 mmol, 1 equiv), **3** (48.6 mg, 0.210 mmol, 1.05 equiv), Ca(NTf_2_)_2_ (12.0 mg, 0.020 mmol, 0.1 equiv), DABCO (4.5 mg, 0.040 mmol, 0.2 equiv), and TMDS (53.7 mg, 71 μL, 0.400 mmol, 2 equiv) in THF (0.80 mL) for 24 h with stirring at 50 °C. The reaction was run in duplicate. Purification of the duplicate samples by column chromatography using liquid loading gave the product (39 mg, 0.104 mmol, 53% yield) as a white solid. A control reaction, without Ca(NTf_2_)_2_, was also performed, and the product (0.3 mg, 0.807 μmol, <1% yield) was isolated in the same manner. The ^1^H NMR, ^13^C NMR, and ^19^F NMR spectra were consistent with those previously reported.^32 1^H NMR (CDCl_3_, 400 MHz): δ 8.35 (s, 1H), 7.78–7.76 (m, 2H), 7.64–7.60 (m, 2H), 7.56–7.53 (m, 2H), 6.61 (d, J = 8.0 Hz, 1H), 3.77–3.75 (m, 4H), 3.13–3.11 (m, 4H). ^13^C NMR (CDCl_3_, 101 MHz): δ 159.8, 145.8 (q, JCF = 4.4 Hz), 135.4, 134.9 (q, JCF = 3.0 Hz), 133.3, 129.4, 127.9, 124.4 (q, JCF = 271.7 Hz), 116.1 (q, JCF = 33.0 Hz), 105.9, 45.8, 44.3. ^19^F NMR (CDCl_3_, 376 MHz): δ −60.69. HRMS (TOF^+^) *m*/*z*: [M^+^] 371.09098; found, 371.09252.

#### 4-((4-(6-(Trifluoromethyl)pyridin-2-yl)piperazin-1-yl)sulfonyl)benzonitrile **24**

The reaction was performed using the general procedure with commercially available 4-cyanobenzenesulfonyl fluoride (37 mg, 0.200 mmol, 1 equiv), **3** (48.6 mg, 0.210 mmol, 1.05 equiv), Ca(NTf_2_)_2_ (12.0 mg, 0.020 mmol, 0.1 equiv), DABCO (4.5 mg, 0.040 mmol, 0.2 equiv), and TMDS (53.7 mg, 71 μL, 0.400 mmol, 2 equiv.) in THF (0.80 mL) for 24 h with stirring at 50 °C. Purification of the duplicate samples by column chromatography using solid loading gave the product (53 mg, 0.330 mmol, 67% yield) as a white powder. A control reaction, without Ca(NTf_2_)_2_, was also performed, and the product (19.1 mg, 0.048 mmol, 24% yield) was isolated in the same manner. The ^1^H NMR, ^13^C NMR, and ^19^F NMR spectra were consistent with those previously reported.^32 1^H NMR (CDCl_3_, 400 MHz): δ 7.89 (d, J = 8 Hz, 2 H), 7.84 (d, J = 8.0 Hz, 2 H), 7.59 (t, J = 8.0 Hz, 1 H), 6.99 (d, J = 8.0 Hz, 1 H), 6.76 (d, J = 8.0 Hz, 1 H), 3.73 (t, J = 4.0 Hz, 4 H), 3.18–3.15 (m, 4 H). ^13^C NMR (CDCl_3_, 101 MHz): δ 157.9, 146.6 (q, JCF = 34.3 Hz), 140.2, 139.0, 133.1, 128.4, 121.5 (q, JCF = 275.4 Hz), 117.3, 117.0, 110.2 (q, JCF = 3.1 Hz), 109.9, 45.7, 44.5. ^19^F NMR (CDCl_3_, 376 MHz): δ −68.15. HRMS (TOF+) *m*/*z*: [M+] 396.08623; found, 396.08784.

## Results and Discussion

### Computational Model

**Figure 3:**
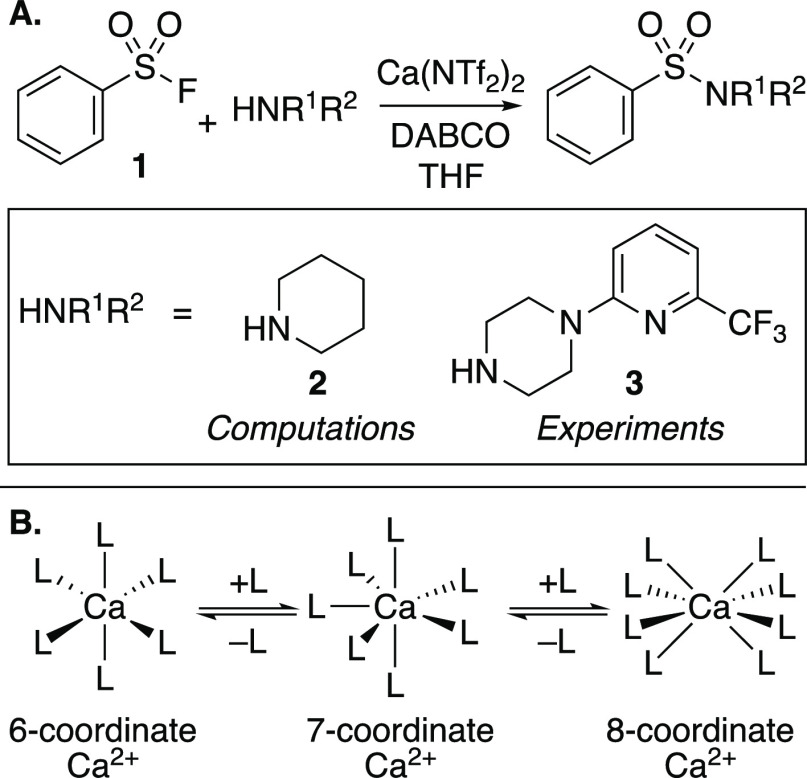
(A) Reaction of study. (B) Mononuclear 6-, 7-, and 8- coordinate Ca^2+^ complexes were computed at each stationary point of the reaction.

In our previous report, we demonstrated that sulfonyl fluorides (RSO_2_F), sulfamoyl fluorides (R_2_NSO_2_F), and fluorosulfates (ROSO_2_F) could be successfully converted to nitrogen-containing sulfur(VI) compounds using a wide variety of predominant secondary amines as nucleophilic reagents. To study this Ca(NTf_2_)_2_-mediated SuFEx reaction computationally, we chose a generalizable model SuFEx reaction that may explain the robust reactivity (Figure 3a). Specifically, most of the results reported herein arise from our computational study of Ca(NTf_2_)_2_- and DABCO- mediated SuFEx of benzenesulfonyl fluoride **1** with piperidine **2** in THF—the solvent used in our published study.^32^ Wherein experimental kinetics data were reported, we employed 1-(6-(trifluoromethyl)pyridin-2-yl)piperazine **3** from our 2020 study, with the CF_3_ group aiding in ^19^F NMR measurements.

All previous reports studying mechanisms of calcium-mediated reactions have invoked direct (i.e., the first coordination sphere) calcium-substrate interactions in explaining modes of substrate activation.^13,28,60−62^ Likewise, in our reaction of study, we hypothesized that Ca^2+^ facilitates the chemical reaction by providing direct Lewis acid stabilization to the reagents during S(VI)–F activation. Therefore, in our computational approach, we focused on generating geometries and coordination isomers at the first coordination sphere around the Ca^2+^ center.

Previous crystallographic and computational data on Ca^2+^ first coordination geometries have inferred that the most prevalent Ca^2+^ centers involve hexa- (6-), hepta- (7-), and octa- (8-) coordination modes.^63^ Moreover, we found with order studies that indeed our SuFEx reaction is first-order in Ca(NTf_2_)_2_ (up to ∼0.8 equiv), supporting a mononuclear Ca^2+^ species in the reaction (see Supporting Information). Therefore, at each stationary point along the reaction coordinate, we accounted for and quantified the relative stability of mononuclear 6-, 7-, and 8- coordination Ca^2+^ complexes using the appropriate number of coordinating solvent molecules (Figure 3b). For example, in modeling Ca(NTf_2_)_2_, we know from our calculations and previously reported crystallographic data^64^ that each NTf_2_^–^ ligand binds in a bidentate fashion to the calcium center, occupying four coordination sites. Therefore, accounting for 6-, 7-, and 8- Ca^2+^ coordination, we computed the relative stability of Ca(NTf_2_)_2_ complexes with 2, 3, and 4 coordinating THF ligands.

### Pre-SuFEx Ca^2+^ Resting State

**Figure 4:**
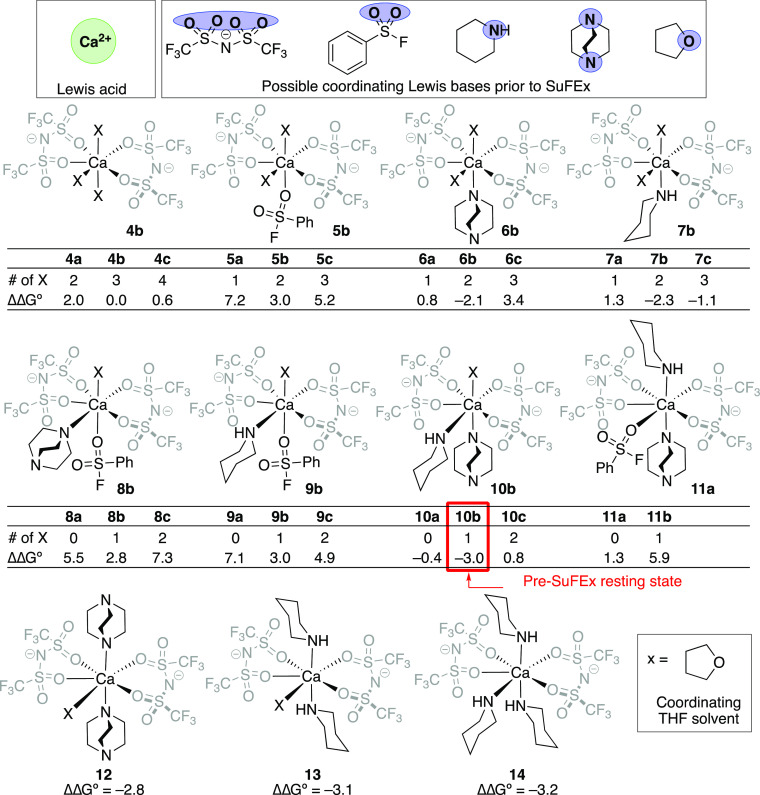
Computed thermodynamic stability of pre-SuFEx Ca^2+^ complexes. Relative Gibbs free energies (ΔΔ*G*°) are reported in kcal/mol units with respect to the seven-coordinate solvated Ca(NTf_2_)_2_ salt—Ca(NTf_2_)_2_(THF)_3_**4b**.

To accurately compute the barrier for sulfur(VI) fluoride activation by the calcium salt, we set out first to determine the most probable pre-activation Ca^2+^ resting state. In addition to the coordinating NTf_2_^–^ anions, four unique ligands in the reaction can coordinate to the Ca^2+^ center (Figure 4). With each NTf_2_^–^ coordinating in a bidentate fashion, there are two, three, or four sites for additional ligands to form 6-, 7-, and 8- coordinated Ca^2+^ complexes. As a result, we initially identified eight families of Ca(NTf_2_)_2_ complexes distinguished by ligand identity (**4**–**11**), within which the remaining coordination sites are occupied by THF molecules to form 6-, 7-, and 8- coordinate Ca^2+^ complexes (labeled as **a**, **b**, and **c**, respectively). We considered (i) Ca(NTf_2_)_2_ solvated by THF (complexes **4a**, **4b**, and **4c**, respectively); (ii) Ca(NTf_2_)_2_ with one reagent—benzenesulfonyl fluoride **1** (complexes **5a**–**c**), DABCO (complexes **6a–c**), and piperidine **2** (complexes **7a–c**); (iii) Ca(NTf_2_)_2_ with two different reagents—with benzenesulfonyl fluoride **1** and DABCO (complexes **8a–c**), benzenesulfonyl fluoride **1** and piperidine **2** (complexes **9a–c**), and DABCO and piperidine **2** (complexes **10a-c**); and finally (iv) Ca(NTf_2_)_2_ with benzenesulfonyl fluoride **1**, DABCO, and piperidine **2** (complexes **11a-b**).

To compare the relative stabilities of the Ca^2+^ complexes, we modeled chemical equilibria in which non-coordinating ligands were computed separately (i.e., not interacting with the Ca^2+^ complex or with other species, see Supporting Information).

This search illuminated trends that improve our understanding of Ca^2+^-substrate interactions. We first focused on THF-solvated Ca(NTf_2_)_2_**4a–c**. The lowest energy species features a seven-coordinate Ca^2+^ complex with three THF molecules, **4b**. This solvated species was 2.0 kcal/mol lower in energy than the six-coordinate two THF species **4a** and 0.6 kcal/mol lower than the eight-coordinate four THF species **4c**. From this starting point, benzene sulfonyl fluoride **1**, DABCO, and piperidine **2** were systematically added, and the ground state energies were determined. Similar to **4**, within each family (i.e., **a**–**c** in complexes **5–11**), seven-coordinate Ca^2+^ was thermodynamically preferred over six- and eight-coordinate Ca^2+^ (see Supporting Information for enthalpic and entropic contributions to the reported thermodynamic stabilities in Figure 4).

Next, we address the speciation of Ca(NTf_2_)_2_ in the presence of other reagents in the reaction compared to THF-solvated Ca^2+^ (**4b**). The lowest energy Ca(NTf_2_)_2_ complex with coordinating benzenesulfonyl fluoride (**5b**) is 3.0 kcal/mol less stable than **4b**, while the Ca(NTf_2_)_2_ with a coordinating DABCO (**6b**) or piperidine (**7b**) is more stable than **4b** by 2.1 kcal/mol and 2.3 kcal/mol, respectively. These data reveal that displacing THF is thermodynamically disfavored when with benzenesulfonyl fluoride **1** but favored with either of the amines (DABCO or piperidine **2**). Moreover, Ca(NTf_2_)_2_ complexes with benzenesulfonyl fluoride and either of the amines (complexes **8a–c**, **9a–c**, and **11a-b**) are less stable than complexes with DABCO and piperidine (complexes **10a-c**).

Considering the 2.31:1 ratio of amine (DABCO + piperazine) to Ca^2+^ present in the reaction,^43^ we next investigated whether coordinating excess amines to the Ca^2+^ center would yield more stable complexes. Indeed, the most stable pre-SuFEx complexes are seven-coordinate Ca^2+^ salts, which feature the coordination of either two DABCO molecules (**12**), one DABCO and one piperidine (**10b**), or two or three piperidine molecules (**13** and **14**, respectively). However, given that these complexes are almost equienergetic (ΔΔ*G*° ≤ 0.4 kcal/mol of each other) and approximately equal concentrations of amines are used in the reaction, we conclude that the most likely pre-SuFEx resting state is complex **10b** with one coordinating DABCO, one piperidine, and one THF.

### SuFEx Activation Mechanism and Barrier

**Figure 5:**
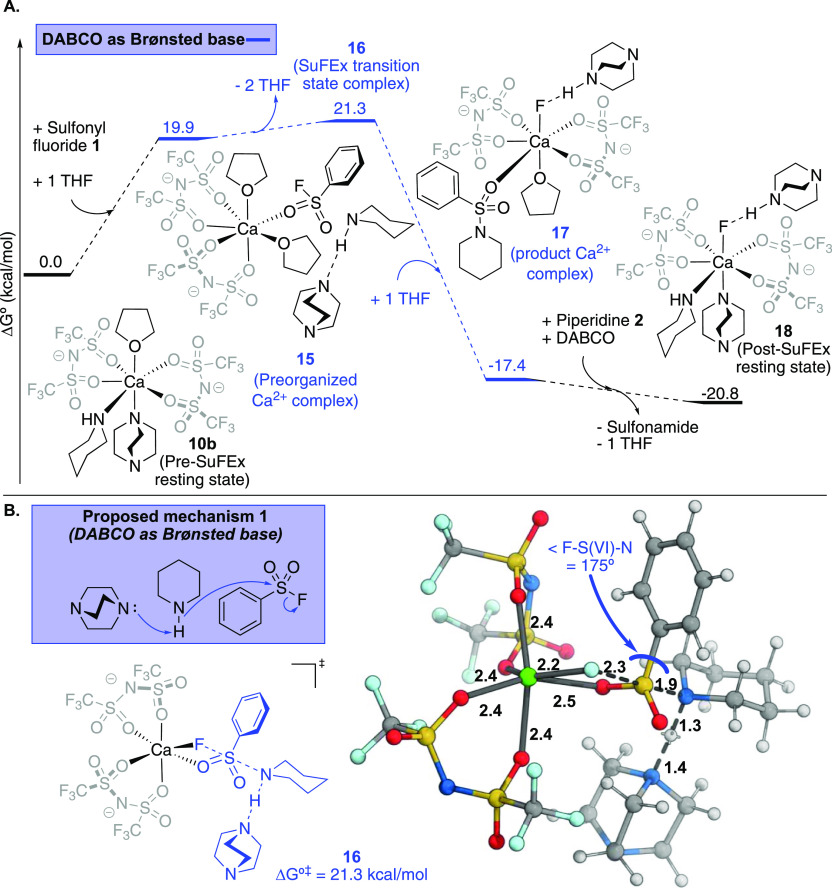
(A) Computed minimum energy reaction coordinate for Ca(NTf_2_)_2_- and DABCO- mediated SuFEx with benzenesulfonyl fluoride **1** and piperidine **2** in THF. Shown in (B) is the lowest energy transition state structure at the rate-determining step of the DABCO-as-the-Brønsted-base mechanism. All distances reported in the Ångström (Å) unit. Two-point Ca^2+^ activation and DABCO as the Bronsted base.

With the identification of pre-SuFEx resting-state complex **10b**, we investigated the activation of benzenesulfonyl fluoride **1** by the Ca(NTf_2_)_2_ complex toward sulfonamide formation (Figure 5a). In the computed minimum energy pathway, the coordinating piperidine and DABCO in **10b** are displaced from the first Ca^2+^ coordination sphere by sulfonyl fluoride **1** and THF, both coordinated in a monodentate fashion, thus forming complex **15** preorganized for SuFEx. The nucleophilic substitution transition state **16** is preceded by displacement of two THF molecules, presumably allowing for bidentate coordination of **1** at the sulfonyl oxygen and fluorine at the transition state (vide infra) and resulting in the fluoride-ligated, post-SuFEx Ca^2+^ product complex **17**. Ligand exchange of the sulfonamide product and THF with piperidine **2** and DABCO led to the post-SuFEx resting state **18**. Overall, the reaction is thermodynamically favored (Δ*G*_rxn_^°^ = −20.8 kcal/mol), and the computed Gibbs free energy barrier (Δ*G*°^‡^) from resting-state complex **10b** to transition state complex **16** is 21.3 kcal/mol. To ensure that experimental observations corroborate our computed barrier, we performed kinetics experiments on the analogous SuFEx reaction with piperazine **3**. Using an Eyring plot, the experimental Gibbs free energy activation barrier (ΔG_exp_^°‡^) for this reaction was determined to be 21.5 ± 0.14 kcal/mol (see Supporting Information). We were encouraged that our computed activation barrier for the model piperidine is consistent with the experimentally derived barrier of the piperazine substrate. Furthermore, both the computed and experimental barriers presented above are consistent with the estimated barriers for sulfonamide formation with the reported amines in the 2020 study (est. Δ*G*_exp_^‡^ range from 21 to 22 kcal/mol, see Supporting Information).

Analogous to the search for resting-state complexes, we accounted for the possibility of a 6-, 7-, or 8- coordinate Ca^2+^ complex in the search for the transition state complex **16** (Figure 5b). In doing so, we computed 57 unique transition state conformational isomers with 0 coordinating THF, 58 isomers with 1 THF, and 58 isomers with 2 THF. We uncovered that in the transition state, the six-coordinate Ca(NTF_2_)_2_ complex with 0 coordinating THF was the most energetically favored.

We analyzed each unique transition state conformational isomer within 3.0 kcal/mol to the lowest energy complex **16** and realized a series of conserved features.

### Core Bond-Forming/Breaking Process

At the transition state, the forming S(VI)–N bond distance between sulfonyl fluoride **1** and incoming piperidine **2** is at 1.9 Å, the breaking S(VI)–F bond distance is at 2.3 Å, and the N–S(VI)–F angle is at 175°. This core bond-forming/breaking geometry was consistent across all computed isomers, with deviations of ±0.1 Å for the distances and ± 2° for the angles from those in complex **16**. The linear geometry supports either an S_N_2 (i.e., concerted) or an addition–elimination (i.e., stepwise) nucleophilic substitution process. However, intrinsic reaction coordinate (IRC) analyses^65^ from all computed 6-, 7-, and 8- coordinate Ca^2+^ SuFEx transition state geometries yielded complexes **15** and **17** (Figure 5a) as the lowest-energy-connecting ground state structures. Neither **15** nor **17** features a five-coordinate sulfur(VI) intermediate that would be predicted in an addition–elimination mechanism. Where five-coordinate sulfur(VI) complexes were isolated from IRC analysis, the refined ωB97XD energies of these complexes were higher than the transition state complex **16** and hence not energetically likely stationary points on the computed SuFEx reaction coordinate. Taken together, while we cannot completely rule out the addition–elimination mechanism, the data support a concerted S_N_2 process for Ca(NTf_2_)_2_-mediated sulfur(VI)–fluoride exchange.

### Lewis-Acid Activation

The S(VI)–F distance in transition state complex **16** is elongated by 0.7 Å from the ground state geometry of benzenesulfonyl fluoride **1**. The natural charge, obtained through natural population analysis,^66^ of the F atom at the transition state is −0.828, depicting an increase in magnitude from −0.485 in the ground state (see Supporting Information). The dissociation of the fluoride from the sulfur(VI) center and charge buildup at the fluoride in **16** suggest that the departing anion is stabilized at the Ca^2+^ center in the transition state. Indeed, we see in complex **16** that benzenesulfonyl fluoride is coordinated in a bidentate fashion to Ca^2+^ via the departing fluoride (Ca–F = 2.3 Å) and one sulfonyl oxygen (Ca–O = 2.7 Å). Here again, the bidentate coordination geometry was remarkably consistent (±0.0 Å for Ca–F and ±0.1 Å Ca–O) across all computed transition state isomers. Moreover, to our best effort, we could not isolate a SuFEx transition state geometry without this bidentate coordination. Taken together, the data reveal the mode and the importance of Ca(NTf_2_)_2_ in activating sulfonyl fluorides for nucleophilic substitution—Ca^2+^ stabilizes the developing charges of benzenesulfonyl fluoride at two contact points: the SO_2_ (S=O) moiety and the departing fluoride.

### Brønsted-Base Activation

In our computed transition state, the nucleophilic addition of piperidine to sulfur(VI) occurs concurrently with proton transfer from piperidine to DABCO. This is evident by the transferring proton being equidistant to the nitrogen on piperidine and DABCO (1.3 and 1.4 Å, respectively in **16**). In the lowest energy complex **16**, piperidine adds to sulfur(VI) in the equatorial position of the ring and transfers the proton to DABCO in the axial position, although we do see the inverse being the case in higher energy isomers. Regardless, the computed transition states support the conclusion that DABCO serves as a Brønsted base activator, deprotonating the nucleophilic piperidine during addition to sulfur(VI).

### DABCO Does Not Provide Lewis-Base Activation

**Figure 6:**
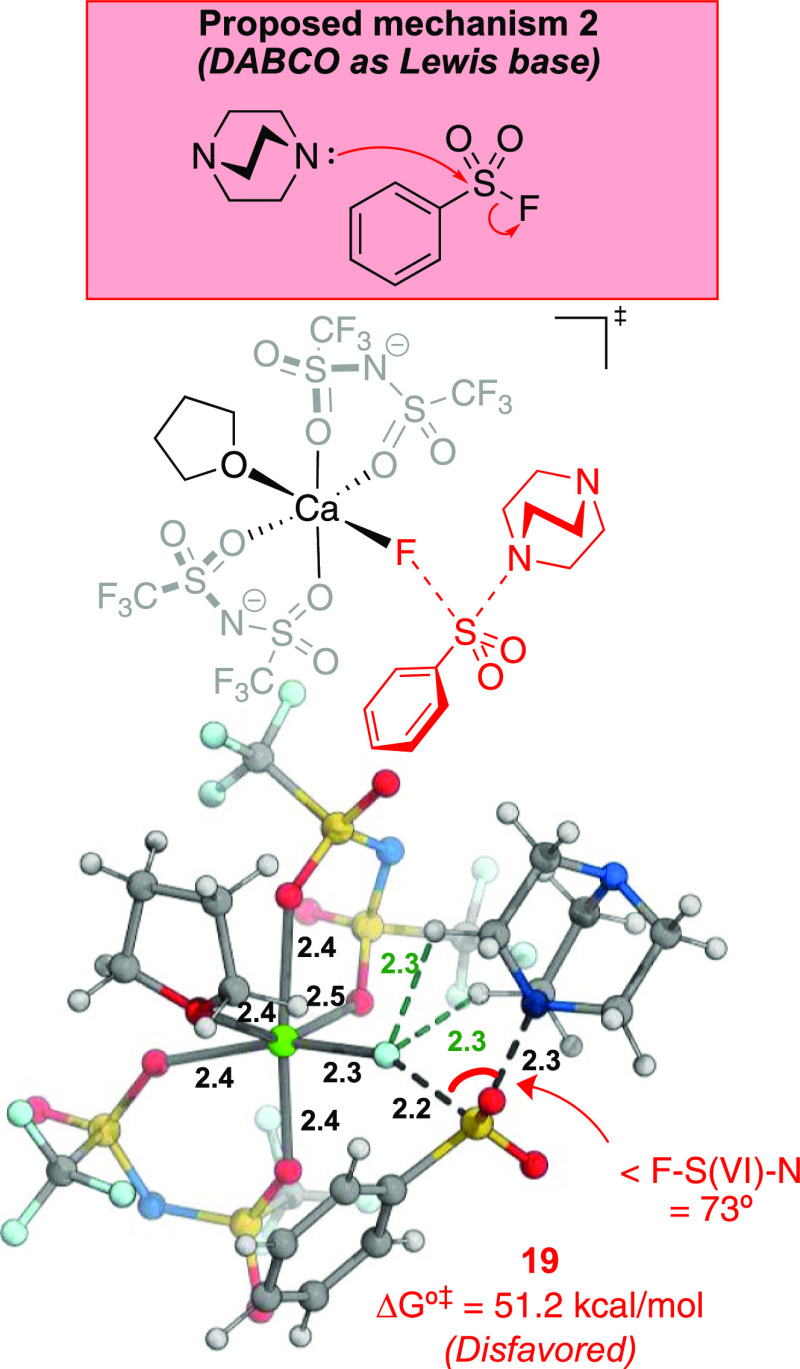
Computed lowest-energy transition state structure at the rate-determining step of the DABCO-*as*-the-Lewis-base mechanism. All distances reported in the Ångström (Å) unit.

We envisioned a second plausible mechanism for sulfonamide formation distinguished by the role of DABCO in the reaction (Figure 6). DABCO serves as a Lewis-base activator in this mechanism, displacing the fluoride at sulfur(VI) via nucleophilic addition. This activated electrophile may undergo a second nucleophilic substitution by piperidine and subsequent proton transfer to form the sulfonamide product. We anticipated that the formation of the activated sulfonium ion is rate-limiting and hence computed the activation barrier for this elementary step to determine whether this mechanism is energetically viable based on reaction conditions. We isolated transition state complex **19** and determined the barrier from complex **10b** to be 51.2 kcal/mol, which is significantly disfavored by 29.9 kcal/mol when compared to the transition state complex **19** for the DABCO-*as*-the-Brønsted-base mechanism. Given that the Ca(NTf_2_)_2_ and DABCO-mediated SuFEx reactions are performed at room temperature at 30 min to 1 h reaction times with high yields, it is not likely that the DABCO-*as*-the-Lewis-base mechanism is energetically accessible in this reaction.

A closer look at complex **19** reveals that, unlike in **16**, the incoming (DABCO) nucleophile lacks transferring N–H protons and is not activated by an exogenous base. Also, DABCO inserts directly into the S(VI)–F bond, as evident by the compressed 73° N–S(VI)–F angle. This distorted geometry allows for the developing positive charge on DABCO to be partially stabilized by ionic C–H···F interactions with the departing fluoride.^67^ Luy and Tonner recently reported in a computational study the SuFEx reaction between methanesulfonyl fluoride and methylamine without an exogenous base and showed a similarly compressed transition state geometry to achieve intramolecular base activation; a high activation barrier was also reported.^68^ Similar to their conclusions, we propose that transition state complex **19** is destabilized with respect to **16,** primarily due to the geometric and electronic constraints imposed on SuFEx transition states that lack exogenous base activation.

### Post-SuFEx Ca-F Complexes and Catalytic Turnover.

**Figure 7:**
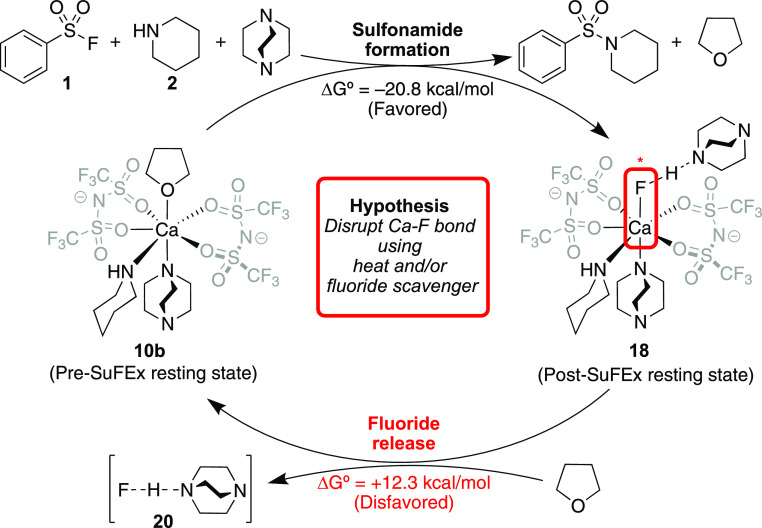
Proposed computed catalytic cycle describing post-SuFEx Ca-F complexes and catalytic turnover.

In contrast to ample examples in the literature of Ca(NTf_2_)_2_ catalyzing organic transformations, our reported SuFEx reaction required stoichiometric Ca(NTf_2_)_2_ to achieve high yields, precluding catalysis.^32^ From our computations, Ca(NTf_2_)_2_-mediated sulfonamide formation is thermodynamically favored (Δ*G*° = −20.8 kcal/mol, Figure 7), and the post-SuFEx resting-state complex **18** features a coordinating fluoride that is further stabilized via hydrogen bonding with protonated DABCO. Dissociation of this fluoride from Ca^2+^ by way of a DABCO-HF adduct **20** is energetically uphill by 12.3 kcal/mol, thus showing that regenerating Ca(NTf_2_)_2_ from stable Ca–F product calcium species is disfavored and is a likely contributor to inhibited catalytic turnover in the SuFEx process.

With these data, we hypothesized that catalytic efficiency of Ca(NTf_2_)_2_ may be improved by disrupting the stable Ca–F product complexes using heat or silanes/siloxanes as fluoride scavengers,^69^ thereby enabling the use of substoichiometric Ca(NTf_2_)_2_ and DABCO. We designed a series of experiments to test this hypothesis, employing piperazine **3** as the nucleophilic reagent (Table 1).

**Table 1:**
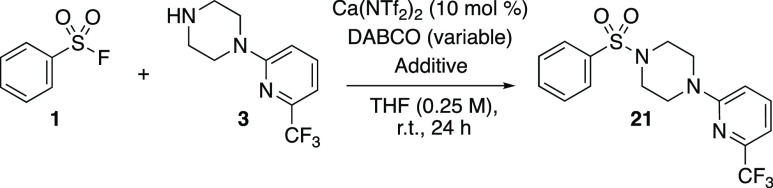
Catalytic Turnover in the Presence of Bases and Additives^a^^,^^b^

entry	base (equiv)	conditions/additives (equiv)	yield (%)^a^	excess yield due to Ca(NTf_2_)_2_ (%)^c^
1	DABCO (1.5)	No Ca	trace^b^	
2	DABCO (1.5)		42	42
3	DABCO (0.2)		35	35
4	DABCO (0.2)	50 °C	65	64
5	DABCO (0.2)	50 °C + MDES^d^(2.0)	64	62
6	DABCO (0.2)	50 °C + TMDS^e^(2.0)	71	71

a Yields were determined by ^19^F NMR spectroscopy with 3-iodobenzotrifluoride as an internal standard. Yields are an average of two runs.

b < 1% yield by ^19^F NMR spectroscopy with 3-iodobenzotrifluoride as an internal standard.

c Excess yield due to Ca(NTf_2_)_2_ is reaction yield with Ca(NTf_2_)_2_ minus control reaction yield (without Ca(NTf_2_)).

d MDES = methyldiethoxysilane.

e TMDS = 1,1,3,3-tetramethyldisiloxane.

We redesigned experimental conditions from our 2020 study this time by using 10 mol % Ca(NTf_2_)_2_ with varying equivalents of DABCO (Table 1). To demonstrate that catalysis is possible with low Ca(NTf_2_)_2_ and DABCO loading, we sought to observe how our conditions affected the yield of sulfonamide **21** monitoring the reaction by ^19^F NMR spectroscopy. To demonstrate that calcium-based catalysis is possible, we monitored and compared the yield of sulfonamide **21** with and without substoichiometric quantities of calcium (more details in the Supporting Information). Our initial reaction demonstrated that 1.5 equiv of DABCO gave sulfonamide **21** from SuFEx of benzenesulfonyl fluoride **1** and piperazine **3** in 42% yield (Table 1, entry 2). Notably, in the absence of Ca, only a trace amount of **21** was formed (Table 1, entry 1).

**Figure 8:**
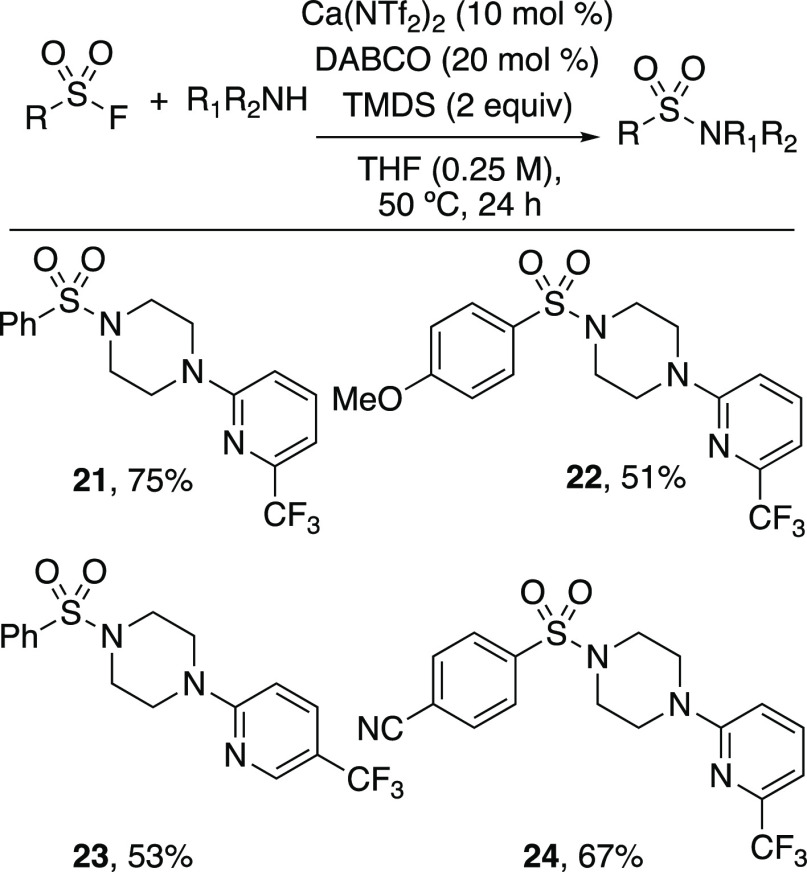
Isolated yields of sulfonamides using 10 mol % Ca(NTf_2_)_2_, 20 mol % DABCO, and 2 equiv TMDS. Yields are an average of two runs.

Next, we aimed to understand if we could facilitate catalysis by also lowering the equivalents of DABCO. A wide range of equivalents of DABCO were attempted (see Supporting Information), although we found that 0.2 equivalent or 20% mol of DABCO gave good yields of sulfonamide **21**. At room temperature, lowering the equivalents of DABCO from 1.5 to 0.2 decreased the yield of **21** to 35% (Table 1, entry 3). Gratifyingly, heating the reaction to 50 °C dramatically increased yields to 65% (Table 1, entry 4). At 50 °C, adding silane MDES did not significantly increase yields of **21** (Table 1, entry 5). Interestingly, using TMDS at 50 °C did result in increased yields (71%, Table 1, entry 6); suggesting that at elevated temperatures, adding a fluoride trap could further assist catalysis. Notably, no detectable conversion of starting material was detected by ^19^F NMR spectroscopy at 50 °C in both the presence and absence of silicon reagents. Lastly, to demonstrate the potential of this catalysis, we successfully applied the DABCO/TMDS/heat reaction conditions to obtain isolated yields of **21** and three other sulfonamides (**22**–**24**) using different piperazine and electronically diverse sulfonyl fluorides in good yield (Figure 8). While the formation of sulfonamides **21–23** from electron neutral and electron-rich sulfonyl fluorides had little to no background reaction in the absence of Ca(NTf_2_)_2_; *p*-cyanosulfonyl fluoride yielded higher background formation of sulfonamide **24** (24% yield, see Supporting Information). Nevertheless, in the presence of calcium, sulfonamide formation was considerably boosted. Collectively, these data demonstrate that Ca^2+^ catalysis is feasible with SuFEx chemistry, and efforts are ongoing toward further optimization. Those data will be featured in a future report.

## Conclusions

We have developed a reactivity model for the Ca(NTf_2_)_2_- and DABCO-mediated SuFEx conversion of sulfonyl fluorides to medicinally relevant sulfonamides. Ca(NTf_2_)_2_ activates the substrate via a critical two-point activation mode where the Lewis acidic Ca^2+^ center stabilizes the developing negative charges at the leaving fluoride and the oxygen at the SO_2_F moiety during the SuFEx process. In this model, the DABCO additive facilitates the reaction by providing additional Brønsted-base activation of the amine reagent. We hypothesized that incorporation of fluoride scavengers to disrupt the stable Ca–F complexes formed post SuFEx will improve catalytic efficiency by lowering the equivalents of the calcium salt and DABCO—a hypothesis supported by proof-of-principle experiments demonstrating a good catalytic turnover using 10 mol % of Ca(NTf_2_)_2_ and 20 mol % of DABCO at 50 °C with two equivalents of TMDS. The work presented represents the first comprehensive mechanistic investigation of metal-mediated SuFEx reactions and serves as a foundational platform for future developments in calcium catalysis, organosulfur, and fluorine chemistry.

## Supporting Information Available

The following files are available free of charge. Additional computational and experimental details; Cartesian coordinates for all computed structures;^1^H, ^19^F, and ^13^C NMR spectra for all compounds; and HRMS and IR data (PDF)

## References

[ref1] Harder S. From Limestone to Catalysis: Application of Calcium Compounds as Homogeneous Catalysts. Chem. Rev. 2010, 110, 3852–3876. 10.1021/cr9003659. 20420358

[ref2] Begouin J.-M.; Niggemann M. Calcium-Based Lewis Acid Catalysts. Chem.—Eur. J. 2013, 19, 8030–8041. 10.1002/chem.201203496. 23712417

[ref3] Cotton F. A.; Wilkinson G.; Gaus P. L. Basic Inorganic Chemistry, 3rd ed.; Wiley, 1995.

[ref4] Piesik D. F. J.; Häbe K.; Harder S. Ca-Mediated Styrene Polymerization: Tacticity Control by Ligand Design. Eur. J. Inorg. Chem. 2007, 2007, 5652–5661. 10.1002/ejic.200700802.

[ref5] Feil F.; Harder S. Hypersilyl-Substituted Complexes of Group 1 and 2 Metals: Syntheses, Structures and Use in Styrene Polymerisation. Eur. J. Inorg. Chem. 2003, 2003, 3401–3408. 10.1002/ejic.200300149.

[ref6] Harder S.; Feil F. Dimeric Benzylcalcium Complexes: Influence of THF in Stereoselective Styrene Polymerization. Organometallics 2002, 21, 2268–2274. 10.1021/om020092w.

[ref7] Harder S.; Feil F.; Knoll K. Novel Calcium Half-Sandwich Complexes for the Living and Stereoselective Polymerization of Styrene. Angew. Chem. 2001, 113, 4391–4394. 10.1002/1521-3757(20011119)113:22<4391::AID-ANGE4391>3.0.CO;2-H. 29712082

[ref8] Harder S.; Feil F.; Weeber A. Structure of a Benzylcalcium Diastereomer: An Initiator for the Anionic Polymerization of Styrene. Organometallics 2001, 20, 1044–1046. 10.1021/om000945p.

[ref9] Crimmin M. R.; Arrowsmith M.; Barrett A. G. M.; Casely I. J.; Hill M. S.; Procopiou P. A. Intramolecular Hydroamination of Aminoalkenes by Calcium and Magnesium Complexes: A Synthetic and Mechanistic Study. J. Am. Chem. Soc. 2009, 131, 9670–9685. 10.1021/ja9003377. 19552442

[ref10] Datta S.; Gamer M. T.; Roesky P. W. Aminotroponiminate Complexes of the Heavy Alkaline Earth and the Divalent Lanthanide Metals as Catalysts for the Hydroamination/Cyclization Reaction. Organometallics 2008, 27, 1207–1213. 10.1021/om701014d.

[ref11] Datta S.; Roesky P. W.; Blechert S. Aminotroponate and Aminotroponiminate Calcium Amides as Catalysts for the Hydroamination/Cyclization Catalysis. Organometallics 2007, 26, 4392–4394. 10.1021/om700507h.

[ref12] Crimmin M. R.; Casely I. J.; Hill M. S. Calcium-Mediated Intramolecular Hydroamination Catalysis. J. Am. Chem. Soc. 2005, 127, 2042–2043. 10.1021/ja043576n. 15713071

[ref13] Brand S. Calcium-Catalyzed Arene C−H Bond Activation by Low-Valent AlI. Angew. Chem. Int. Ed. 2019, 58, 15496–15503. 10.1002/anie.201908978 PMC6856855 31465144

[ref14] Morcillo S. P.; Leboeuf D.; Bour C.; Gandon V. Calcium-Catalyzed Synthesis of Polysubstituted 2-Alkenylfurans from β-Keto Esters Tethered to Propargyl Alcohols. Chem.—Eur. J. 2016, 22, 16974–16978. 10.1002/chem.201603929. 27735089

[ref15] Meyer V. J.; Niggemann M. Highly Chemoselective Calcium-Catalyzed Propargylic Deoxygenation. Chem.—Eur. J. 2012, 18, 4687–4691. 10.1002/chem.201103691. 22378484

[ref16] Haubenreisser S.; Niggemann M. Calcium-Catalyzed Direct Amination of π-Activated Alcohols. Adv. Synth. Catal. 2011, 353, 469–474. 10.1002/adsc.201000768.

[ref17] Meyer V. J.; Niggemann M. Calcium-Catalyzed Direct Coupling of Alcohols with Organosilanes. Eur. J. Org. Chem. 2011, 2011, 3671–3674. 10.1002/ejoc.201100231.

[ref18] Niggemann M.; Meel M. J. Calcium-Catalyzed Friedel-Crafts Alkylation at Room Temperature. Angew. Chem., Int. Ed. 2010, 49, 3684–3687. 10.1002/anie.200907227. 20391443

[ref19] Lebœuf D.; Marin L.; Michelet B.; Perez-Luna A.; Guillot R.; Schulz E.; Gandon V. Harnessing the Lewis Acidity of HFIP through Its Cooperation with a Calcium(II) Salt: Application to the Aza-Piancatelli Reaction. Chem.—Eur. J. 2016, 22, 16165–16171. 10.1002/chem.201603592. 27690181

[ref20] Basson A. J.; McLaughlin M. G. Sustainable Access to 5-Amino-Oxazoles and Thiazoles via Calcium-Catalyzed Elimination-Cyclization with Isocyanides. ChemSusChem 2021, 14, 1696–1699. 10.1002/cssc.202100225. 33605021 PMC8048476

[ref21] Halpani C. G.; Mishra S. Lewis Acid Catalyst System for Claisen-Schmidt Reaction under Solvent Free Condition. Tetrahedron Lett. 2020, 61, 152175 10.1016/j.tetlet.2020.152175.

[ref22] Uno B. E.; Dicken R. D.; Redfern L. R.; Stern C. M.; Krzywicki G. G.; Scheidt K. A. Calcium(II)-Catalyzed Enantioselective Conjugate Additions of Amines. Chem. Sci. 2018, 9, 1634–1639. 10.1039/C7SC05205G. 29675209 PMC5887857

[ref23] Forkel N. V.; Henderson D. A.; Fuchter M. J. Calcium-Mediated Stereoselective Reduction of α,β-Epoxy Ketones. Tetrahedron Lett. 2014, 55, 5511–5514. 10.1016/j.tetlet.2014.08.050.

[ref24] Forkel N. V.; Henderson D. A.; Fuchter M. J. Lanthanide Replacement in Organic Synthesis: Luche-Type Reduction of α,β-Unsaturated Ketones in the Presence of Calcium Triflate. Green Chem. 2012, 14, 2129–2132. 10.1039/C2GC35619H.

[ref25] Vanden Eynden M. J.; Kunchithapatham K.; Stambuli J. P. Calcium-Promoted Pictet-Spengler Reactions of Ketones and Aldehydes. J. Org. Chem. 2010, 75, 8542–8549. 10.1021/jo1019283. 21090691

[ref26] Chitra S.; Pandiarajan K. Calcium Fluoride: An Efficient and Reusable Catalyst for the Synthesis of 3,4-Dihydropyrimidin-2(1H)-Ones and Their Corresponding 2(1H)Thione: An Improved High Yielding Protocol for the Biginelli Reaction. Tetrahedron Lett. 2009, 50, 2222–2224. 10.1016/j.tetlet.2009.02.162.

[ref27] Vanden Eynden M. J.; Stambuli J. P. Calcium-Catalyzed Pictet–Spengler Reactions. Org. Lett. 2008, 10, 5289–5291. 10.1021/ol802173r. 18954062

[ref28] Qi C.; Gandon V.; Lebœuf D. Calcium(II)-Catalyzed Intermolecular Hydroarylation of Deactivated Styrenes in Hexafluoroisopropanol. Angew. Chem. 2018, 130, 14441–14445. 10.1002/ange.201809470. 30187622

[ref29] Kena Diba A.; Begouin J.-M.; Niggemann M. Calcium Catalyzed Hydroalkoxylation. Tetrahedron Lett. 2012, 53, 6629–6632. 10.1016/j.tetlet.2012.08.129.

[ref30] Niggemann M.; Bisek N. Calcium-Catalyzed Hydroarylation of Alkenes at Room Temperature. Chem.—Eur. J. 2010, 16, 11246–11249. 10.1002/chem.201001375. 20730750

[ref31] Yang S.; Bour C.; Lebœuf D.; Gandon V. DFT Analysis into the Calcium(II)-Catalyzed Coupling of Alcohols With Vinylboronic Acids: Cooperativity of Two Different Lewis Acids and Counterion Effects. J. Org. Chem. 2021, 86, 9134–9144. 10.1021/acs.joc.1c01263. 34152770

[ref32] Mahapatra S.; Woroch C. P.; Butler T. W.; Carneiro S. N.; Kwan S. C.; Khasnavis S. R.; Gu J.; Dutra J. K.; Vetelino B. C.; Bellenger J.; am Ende C. W.; Ball N. D. SuFEx Activation with Ca(NTf_2_)_2_: A Unified Strategy to Access Sulfamides, Sulfamates, and Sulfonamides from S(VI) Fluorides. Org. Lett. 2020, 22, 4389–4394. 10.1021/acs.orglett.0c01397. 32459499 PMC7294807

[ref33] Mukherjee P.; Woroch C. P.; Cleary L.; Rusznak M.; Franzese R. W.; Reese M. R.; Tucker J. W.; Humphrey J. M.; Etuk S. M.; Kwan S. C.; am Ende C. W.; Ball N. D. Sulfonamide Synthesis via Calcium Triflimide Activation of Sulfonyl Fluorides. Org. Lett. 2018, 20, 3943–3947. 10.1021/acs.orglett.8b01520. 29888600 PMC9233624

[ref34] Lee C.; Cook A. J.; Elisabeth J. E.; Friede N. C.; Sammis G. M.; Ball N. D. The Emerging Applications of Sulfur(VI) Fluorides in Catalysis. ACS Catal. 2021, 11, 6578–6589. 10.1021/acscatal.1c01201. 34123485 PMC8185885

[ref35] Dong J.; Krasnova L.; Finn M. G.; Sharpless K. B. Sulfur(VI) Fluoride Exchange (SuFEx): Another Good Reaction for Click Chemistry. Angew. Chem., Int. Ed. 2014, 53, 9430–9448. 10.1002/anie.201309399. 25112519

[ref36] Chinthakindi P. K.; Arvidsson P. I. Sulfonyl Fluorides (SFs): More Than Click Reagents?. Eur. J. Org. Chem. 2018, 2018, 3648–3666. 10.1002/ejoc.201800464.

[ref37] Liang D. D.; Streefkerk D. E.; Jordaan D.; Wagemakers J.; Baggerman J.; Zuilhof H. Silicon-Free SuFEx Reactions of Sulfonimidoyl Fluorides: Scope, Enantioselectivity, and Mechanism. Angew. Chem., Int. Ed. 2020, 59, 7494–7500. 10.1002/anie.201915519. PMC7216998 32157791

[ref38] Smedley C. J.; Homer J. A.; Gialelis T. L.; Barrow A. S.; Koelln R. A.; Moses J. E. Accelerated SuFEx Click Chemistry For Modular Synthesis. Angew. Chem., Int. Ed. 2022, 61, e202112375 10.1002/anie.202112375. PMC8867595 34755436

[ref39] Barrow A. S.; Smedley C. J.; Zheng Q.; Li S.; Dong J.; Moses J. E. The Growing Applications of SuFEx Click Chemistry. Chem. Soc. Rev. 2019, 48, 4731–4758. 10.1039/C8CS00960K. 31364998

[ref40] Jones L. H.; Kelly J. W. Structure-Based Design and Analysis of SuFEx Chemical Probes. RSC Med. Chem. 2020, 11, 10–17. 10.1039/C9MD00542K. 33479601 PMC7460715

[ref41] Zheng Q.; Woehl J. L.; Kitamura S.; Santos-Martins D.; Smedley C. J.; Li G.; Forli S.; Moses J. E.; Wolan D. W.; Sharpless K. B. SuFEx-Enabled, Agnostic Discovery of Covalent Inhibitors of Human Neutrophil Elastase. Proc. Natl. Acad. Sci. U.S.A. 2019, 116, 18808–18814. 10.1073/pnas.1909972116. 31484779 PMC6754619

[ref42] Narayanan A.; Jones L. H. Sulfonyl Fluorides as Privileged Warheads in Chemical Biology. Chem. Sci. 2015, 6, 2650–2659. 10.1039/C5SC00408J. 28706662 PMC5489032

[ref43] Yang B.; Wu H.; Schnier P. D.; Liu Y.; Liu J.; Wang N.; DeGrado W. F.; Wang L. Proximity-Enhanced SuFEx Chemical Cross-Linker for Specific and Multitargeting Cross-Linking Mass Spectrometry. Proc. Natl. Acad. Sci. U.S.A. 2018, 115, 11162–11167. 10.1073/pnas.1813574115. 30322930 PMC6217395

[ref44] Schrödinger. Schrödinger Release 2021-3: Macromodel; Schrödinger, LLC: New York, NY, 2021.

[ref45] Jorgensen W. L.; Tirado-Rives J. The OPLS [Optimized Potentials for Liquid Simulations] Potential Functions for Proteins, Energy Minimizations for Crystals of Cyclic Peptides and Crambin. J. Am. Chem. Soc. 1988, 110, 1657–1666. 10.1021/ja00214a001. 27557051

[ref46] Becke A. D. Density-Functional Thermochemistry. III. The Role of Exact Exchange. J. Chem. Phys. 1993, 98, 5648–5652. 10.1063/1.464913.

[ref47] Lee C.; Yang W.; Parr R. G. Development of the Colle-Salvetti Correlation-Energy Formula into a Functional of the Electron Density. Phys. Rev. B 1988, 37, 785–789. 10.1103/physrevb.37.785. 9944570

[ref48] Rassolov V. A.; Ratner M. A.; Pople J. A.; Redfern P. C.; Curtiss L. A. 6-31G* Basis Set for Third-Row Atoms. J. Comput. Chem. 2001, 22, 976–984. 10.1002/jcc.1058.

[ref49] Gordon M. S.; Binkley J. S.; Pople J. A.; Pietro W. J.; Hehre W. J. Self-Consistent Molecular-Orbital Methods. 22. Small Split-Valence Basis Sets for Second-Row Elements. J. Am. Chem. Soc. 1982, 104, 2797–2803. 10.1021/ja00374a017.

[ref50] Francl M. M.; Pietro W. J.; Hehre W. J.; Binkley J. S.; Gordon M. S.; DeFrees D. J.; Pople J. A. Self-Consistent Molecular Orbital Methods. XXIII. A Polarization-Type Basis Set for Second-Row Elements. J. Chem. Phys. 1982, 77, 3654–3665. 10.1063/1.444267.

[ref51] Binkley J. S.; Pople J. A.; Hehre W. J. Self-Consistent Molecular Orbital Methods. 21. Small Split-Valence Basis Sets for First-Row Elements. J. Am. Chem. Soc. 1980, 102, 939–947. 10.1021/ja00523a008.

[ref52] Chai J.-D.; Head-Gordon M. Long-Range Corrected Hybrid Density Functionals with Damped Atom-Atom Dispersion Corrections. Phys. Chem. Chem. Phys. 2008, 10, 6615–6620. 10.1039/b810189b. 18989472

[ref53] Chai J.-D.; Head-Gordon M. Systematic Optimization of Long-Range Corrected Hybrid Density Functionals. J. Chem. Phys. 2008, 128, 084106 10.1063/1.2834918. 18315032

[ref54] Weigend F.; Ahlrichs R. Balanced Basis Sets of Split Valence, Triple Zeta Valence and Quadruple Zeta Valence Quality for H to Rn: Design and Assessment of Accuracy. Phys. Chem. Chem. Phys. 2005, 7, 3297–3305. 10.1039/b508541a. 16240044

[ref55] Schäfer A.; Huber C.; Ahlrichs R. Fully Optimized Contracted Gaussian Basis Sets of Triple Zeta Valence Quality for Atoms Li to Kr. J. Chem. Phys. 1994, 100, 5829–5835. 10.1063/1.467146.

[ref56] Schäfer A.; Horn H.; Ahlrichs R. Fully Optimized Contracted Gaussian Basis Sets for Atoms Li to Kr. J. Chem. Phys. 1992, 97, 2571–2577. 10.1063/1.463096.

[ref57] Mennucci B.; Tomasi J.; Cammi R.; Cheeseman J. R.; Frisch M. J.; Devlin F. J.; Gabriel S.; Stephens P. J. Polarizable Continuum Model (PCM) Calculations of Solvent Effects on Optical Rotations of Chiral Molecules. J. Phys. Chem. A 2002, 106, 6102–6113. 10.1021/jp020124t.

[ref58] Mennucci B.; Tomasi J. Continuum Solvation Models: A New Approach to the Problem of Solute’s Charge Distribution and Cavity Boundaries. J. Chem. Phys. 1997, 106, 5151–5158. 10.1063/1.473558.

[ref59] Frisch M. J.; Trucks G. W.; Schlegel H. B.; Scuseria G. E.; Robb M. A.; Cheeseman J. R.; Scalmani G.; Barone V.; Mennucci B.; Petersson G. A.; Nakatsuji H.; Caricato M.; Li X.; Hratchian H. P.; Izmaylov A. F.; Bloino J.; Zheng G.; Sonnenberg J. L.; Hada M.; Ehara M.; Toyota K.; Fukuda R.; Hasegawa J.; Ishida M.; Nakajima T.; Honda Y.; Kitao O.; Nakai H.; Vreven T.; Montgomery J. A. Jr.; Peralta J. E.; Ogliaro F.; Bearpark M.; Heyd J. J.; Brothers E.; Kudin K. N.; Staroverov V. N.; Kobayashi R.; Normand J.; Raghavachari K.; Rendell A.; Burant J. C.; Iyengar S. S.; Tomasi J.; Cossi M.; Rega N.; Millam J. M.; Klene M.; Knox J. E.; Cross J. B.; Bakken V.; Adamo C.; Jaramillo J.; Gomperts R.; Stratmann R. E.; Yazyev O.; Austin A. J.; Cammi R.; Pomelli C.; Ochterski J. W.; Martin R. L.; Morokuma K.; Zakrzewski V. G.; Voth G. A.; Salvador P.; Dannenberg J. J.; Dapprich S.; Daniels A. D.; Farkas Ö.; Foresman J. B.; Ortiz J. V.; Cioslowski J.; Fox D. J. Gaussian 16, Revision B.01; Gaussian, Inc.: Wallingford CT, 2016.

[ref60] Ward B. J.; Hunt P. A. Hydrophosphination of Styrene and Polymerization of Vinylpyridine: A Computational Investigation of Calcium-Catalyzed Reactions and the Role of Fluxional Noncovalent Interactions. ACS Catal. 2017, 7, 459–468. 10.1021/acscatal.6b02251.

[ref61] Presset M.; Michelet B.; Guillot R.; Bour C.; Bezzenine-Lafollée S.; Gandon V. Gallium(III)- and Calcium(II)-Catalyzed Meyer-Schuster Rearrangements Followed by Intramolecular Aldol Condensation or endo-Michael Addition. Chem. Commun. 2015, 51, 5318–5321. 10.1039/C4CC09514F. 25503868

[ref62] Qi C.; Hasenmaile F.; Gandon V.; Lebœuf D. Calcium(II)-Catalyzed Intra- and Intermolecular Hydroamidation of Unactivated Alkenes in Hexafluoroisopropanol. ACS Catal. 2018, 8, 1734–1739. 10.1021/acscatal.7b04271.

[ref63] Katz A. K.; Glusker J. P.; Beebe S. A.; Bock C. W. Calcium Ion Coordination: A Comparison with That of Beryllium, Magnesium, and Zinc. J. Am. Chem. Soc. 1996, 118, 5752–5763. 10.1021/ja953943i.

[ref64] Xue L.; DesMarteau D. D.; Pennington W. T. Synthesis and Structures of Alkaline Earth Metal Salts of Bis[(Trifluoromethyl)Sulfonyl]Imide. Solid State Sci. 2005, 7, 311–318. 10.1016/j.solidstatesciences.2004.10.029.

[ref65] Fukui K. The Path of Chemical Reactions - the IRC Approach. Acc. Chem. Res. 1981, 14, 363–368. 10.1021/ar00072a001.

[ref66] Reed A. E.; Weinstock R. B.; Weinhold F. Natural Population Analysis. J. Chem. Phys. 1985, 83, 735–746. 10.1063/1.449486.

[ref67] Cannizzaro C. E.; Houk K. N. Magnitudes and Chemical Consequences of R_3_N^+^–C–H···OC Hydrogen Bonding. J. Am. Chem. Soc. 2002, 124, 7163–7169. 10.1021/ja012417q. 12059242

[ref68] Luy J.-N.; Tonner R. Complementary Base Lowers the Barrier in SuFEx Click Chemistry for Primary Amine Nucleophiles. ACS Omega 2020, 5, 31432–31439. 10.1021/acsomega.0c05049. 33324855 PMC7726939

[ref69] Wei M.; Liang D.; Cao X.; Luo W.; Ma G.; Liu Z.; Li L. A Broad-Spectrum Catalytic Amidation of Sulfonyl Fluorides and Fluorosulfates. Angew. Chem., Int. Ed. 2021, 60, 7397–7404. 10.1002/anie.202013976. 33337566

