# Supplementary material for: Calcium Bistriflimide-Mediated Sulfur(VI)–Fluoride Exchange (SuFEx): Mechanistic Insights toward Instigating Catalysis
Source: Inorg Chem. 2022 Jun 14;61(25):9746–55. doi: 10.1021/acs.inorgchem.2c01230 (PMC9241145; doi:10.1021/acs.inorgchem.2c01230)
Supplement: Supplementary file 1 — ic2c01230_si_001.pdf [file ic2c01230_si_001.pdf]

Supporting Information

Calcium Bistriflimide Mediated Sulfur(VI)-Fluoride Exchange (SuFEx): Mechanistic Insights Toward Instigating Catalysis

Brian Han,† Samuel R. Khasnavis,‡ Matthew Nwerem,† Michael Bertagna,† Nicholas D. Ball,‡ and O. Maduka Ogba†**

† Chemistry and Biochemistry Program, Schmid College of Science and Technology, Chapman University, One University Drive, Orange, CA 92866

‡ Department of Chemistry, Pomona College, 645 North College Avenue, Claremont, CA 91711

Corresponding authors' email addresses:
Nicholas D. Ball‡*, nicholas.ball@pomona.edu
O. Maduka Ogba†*, ogba@chapman.edu

Table of Contents

Full Authorship of Gaussian 16 3

Conformational Search Protocol 4

Theory Benchmark 5

Lowest Energy States 7

Calculation Parameters, Geometries, Energies, and Vibrational Frequencies 7
Single Point Energies in ωB97xd/def2-TZVP/PCM(THF) 80

Determining Relative Stability of Ca^{2+} Complexes 82

Enthalpic and Entropic Contributions To Ground State $Ca(NTf_2)_2$ Complexes 83

Estimated Gibbs Free Energy Barriers 86

DABCO-As-Bronsted-Base Transition States Within 3.0 kcal/mol 87

Calculation Parameters, Geometries, Energies, and Vibrational Frequencies 88
Single Point Energies in ωB97x-D/def2-TZVP/PCM(THF) 98

Natural Charges from Natural Population Analysis 99

General Experimental Methods: 102

General ^{19}F NMR Kinetic Trial Procedure and Data Extraction: 102

Pulse parameters for the "zgkinetics_F" NMR method 103

Variable Temperature ^{19}F NMR Setup Procedure 105

Activation Parameters Experimental Determination 105

$Ca(NTf_2)_2$ Rate Order Experimentation 108

^{19}F NMR Yield Method 110

Additive and Reaction Conditions Screening with Secondary Amine Nucleophile 111

Replacing DABCO with Alternative Nitrogenous Bases 113

Catalytic Conditions with Diverse Sulfonyl Fluorides and Amines 115

References 116

NMR Spectra of Compounds 21-24 117

Full Authorship of Gaussian 16

Gaussian 16, Revision B.01, M. J. Frisch, G. W. Trucks, H. B. Schlegel, G. E. Scuseria, M. A. Robb, J. R. Cheeseman, G. Scalmani, V. Barone, G. A. Petersson, H. Nakatsuji, X. Li, M. Caricato, A. V. Marenich, J. Bloino, B. G. Janesko, R. Gomperts, B. Mennucci, H. P. Hratchian, J. V. Ortiz, A. F. Izmaylov, J. L. Sonnenberg, D. Williams-Young, F. Ding, F. Lipparini, F. Egidi, J. Goings, B. Peng, A. Petrone, T. Henderson, D. Ranasinghe, V. G. Zakrzewski, J. Gao, N. Rega, G. Zheng, W. Liang, M. Hada, M. Ehara, K. Toyota, R. Fukuda, J. Hasegawa, M. Ishida, T. Nakajima, Y. Honda, O. Kitao, H. Nakai, T. Vreven, K. Throssell, J. A. Montgomery, Jr., J. E. Peralta, F. Ogliaro, M. J. Bearpark, J. J. Heyd, E. N. Brothers, K. N. Kudin, V. N. Staroverov, T. A. Keith, R. Kobayashi, J. Normand, K. Raghavachari, A. P. Rendell, J. C. Burant, S. S. Iyengar, J. Tomasi, M. Cossi, J. M. Millam, M. Klene, C. Adamo, R. Cammi, J. W. Ochterski, R. L. Martin, K. Morokuma, O. Farkas, J. B. Foresman, and D. J. Fox, Gaussian, Inc., Wallingford CT, **2016**.

Conformational Search Protocol

Conformational searches at all stationary points studied were performed using the parameters described below to locate computed configurational and conformational isomers of the Ca^{2+} complexes **4-19** reported in the manuscript. These structures were subject to further structural and energy refinements using quantum mechanics, from which the lowest energy ground and transition state structures were obtained (*vide infra*).

Conformational Search Parameters

Potential:

Force field: Optimized Polarizable Liquid Simulation (OPLS)
Solvent: None
Electrostatic cutoff: None

Minimization:

Minimization method: Polak-Ribier Conjugate Gradient (PRCG)
Maximum iteration: 2500
Convergence on gradient with threshold of 0.05

Conformational Sampling:

Method: Torsional sampling (MCMM)
Maximum number of steps: 1000
Energy window: 5.00 kcal/mol
Redundancy window: 1.0 Å maximum atom deviation

Theory Benchmark

Shown in **Figure S1** is a comparative geometric analysis of transition state complex **16** re-optimized using the following levels of theory.

Theory 1: B3LYP/6-311+G(d,p)/LANL2DZ(Ca)
Theory 2: ωB97XD/6-31G(d,p)

Theory 1 evaluates the effect of explicit treatment of non-Ca atoms using the triple zeta basis set. Theory 2 evaluates the effect of using the hybrid meta-GGA ωB97XD functional on the geometry.

Regardless of the level of theory, electronic energies on the single-point geometries were computed at the ωB97XD/def2-TZVP/PCM(THF) level of theory; the same one employed in the manuscript.

An overlay of these structures using *PyMOL* shows that at the global level, the optimized geometries are similar (0.195 Å and 0.183 Å RMSD for Theory 1 and Theory 2, respectively) to that using the employed level of theory in the manuscript, suggesting that basis sets and functional changes may not alter the global geometries of these complexes. However, we did observe more subtle changes in the key forming and breaking bonds – a 0.4 Å S-F bond elongation and a 0.1 Å Ca-F bond compression was observed using the 6-311+G(d,p) basis set (Theory 1). This suggests to us that the triple zeta basis set more strongly emphasizes the stabilization derived from the key Ca-F interaction discussed in the manuscript. On the other hand, using Theory 2, we see a 0.2 Å S-F bond compression with no significant change to the Ca-F bond length, suggesting the ωB97XD method may slightly deemphasize the Ca-F stabilization. The energetic barriers from the re-optimized resting state complex **10b** were also computed using Theories 1 and 2, and a $\Delta G^{\ddagger}$ of 15.6 and 23.0 kcal/mol, respectively, was found. Taken together with the geometric changes, the energetic data suggests that levels of theory that place more emphasis on Ca-F stabilization would yield lower activation barriers.

Both re-computed barriers are within ± 6 kcal/mol from the 21.3 kcal/mol barrier reported using the employed level of theory in the manuscript. The employed level of theory performs better at reproducing the experimentally observed barrier through kinetics studies ($\Delta G^{\ddagger}$ = 21.5 ± 0.14 kcal/mol) and the estimated activation barriers using reaction time, yield, and temperature data from the 2020 studies ($\Delta G^{\ddagger}$ ~ 21 to 22 kcal/mol).

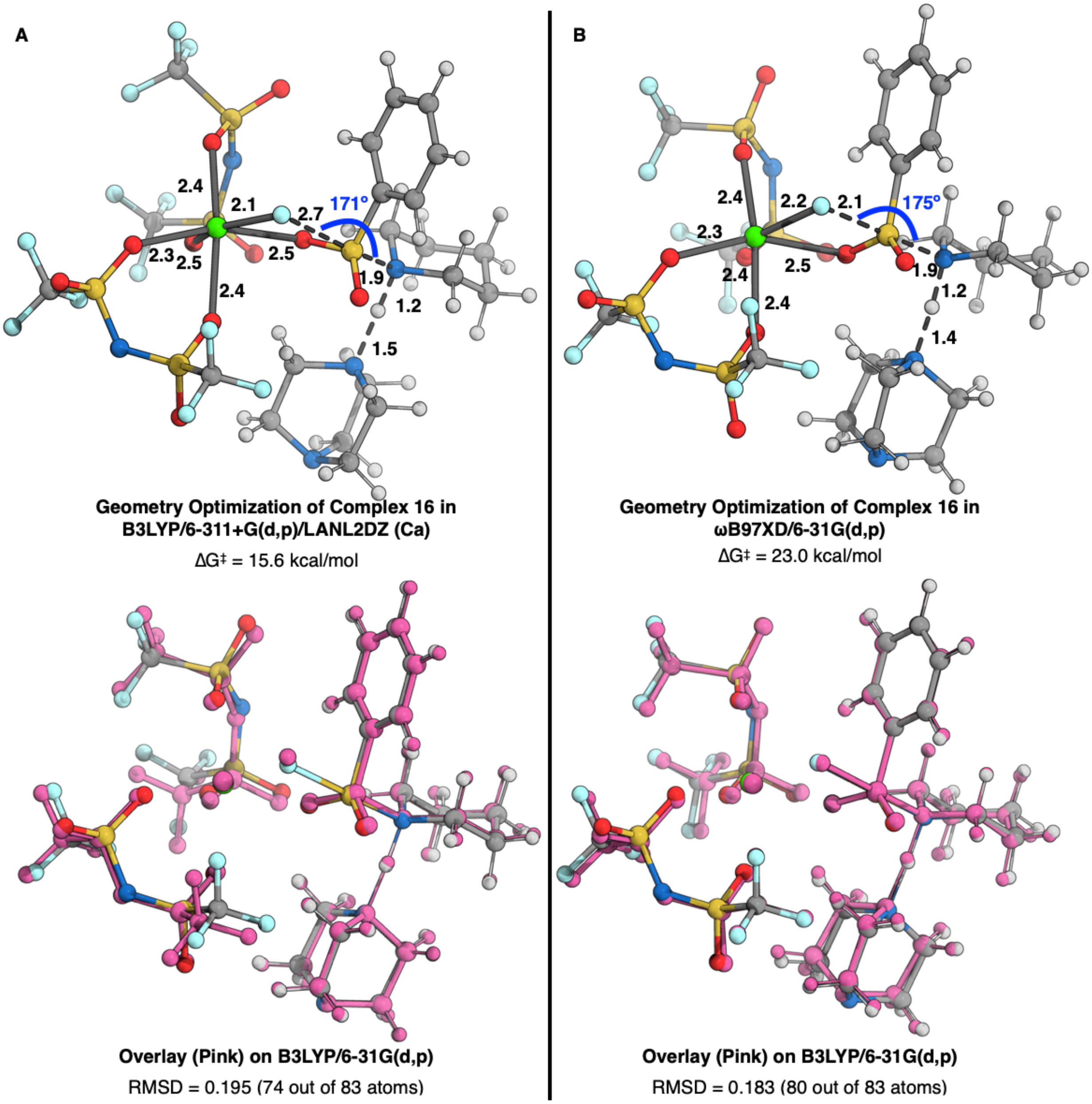

Figure S1. Geometries of the nucleophilic substitution transition state complex **16** re-optimized under two levels of theory – (A) B3LYP/6-31G(d,p), and (B) ωB97XD/6-31G(d,p). The free energy activation barrier ($\Delta G^{\ddagger}$) from resting state complex **10b** re-optimized under the corresponding levels of theory is reported. An overlay of the complex **16** geometries computed using the two levels of theory above (in pink) on that optimized using the B3LYP/6-31G(d,p) level of theory employed in the manuscript is shown with the RMSD value as calculated using the *align* feature in PyMOL.

Lowest Energy States

Calculation Parameters, Geometries, Energies, and Vibrational Frequencies

Benzenesulfonyl fluoride 1

Gaussian 16: ES64L-G16RevB.01 20-Dec-2017

B3LYP/6-31G(d,p) gfprint gfinput scf=(direct,tight,maxcycle=300,xqc) opt=(maxcycle=250) freq=noraman 0-Jul-2020\0\\# B3LYP/6-31G(d,p) gfprint gfinput scf=(direct,tight,max cycle=300,xqc) opt=(maxcycle=250) freq=noraman\\PhSO2F_1.xyz\\0,1\C,1.

Full point group C1 NOp 1
Stoichiometry C6H5FO2S Framework group C1[X(C6H5FO2S)]

Num atoms: 15
Charge = 0 Multiplicity = 1

SCF = -880.072009251 l Predicted change in Energy=-3.728679D-07

Optimization completed.
Maximum Force 0.000334 0.000450 YES
RMS Force 0.000057 0.000300 YES
Maximum Displacement 0.001544 0.001800 YES
RMS Displacement 0.000267 0.001200 YES

Atom Coordinates (in Angstroms)

Type	X	Y	Z
C	1.418220	0.005485	-0.079016
C	0.701460	1.202841	-0.031568
C	-0.692676	1.232700	0.040175
C	-1.383466	0.022575	0.072907
C	-0.683491	-1.185618	0.032735
C	0.711186	-1.195195	-0.045363
H	2.499659	0.022529	-0.152417
H	-1.215305	2.182306	0.057353
H	-2.467445	0.023919	0.125268
H	-1.228003	-2.124594	0.057172
H	1.249234	-2.136852	-0.084586
S	1.592773	2.739793	-0.070986
O	0.719183	3.814563	-0.503614
F	1.814593	2.966443	1.521444
O	2.917180	2.536706	-0.627906

Statistical Thermodynamic Analysis
Temperature 298.150 Kelvin. Pressure 1.00000 Atm.

SCF = -880.072009251 l Predicted change in Energy=-3.728679D-07
Zero-point correction (ZPE) = -879.968634251 0.103375
Internal Energy (U) = -879.960279251 0.11173
Enthalpy (H) = -879.959335251 0.112674
Gibbs Free Energy (G) = -880.002946251 0.069063

Frequencies
40.2324 115.3280 183.6042
270.7754 311.5498 334.8621
413.0718 437.6316 467.2300
517.6790 574.7499 625.7195
693.5976 718.3725 761.3847
795.3844 861.2888 946.7726
987.6959 1013.3876 1014.6265
1047.9219 1098.9733 1108.7994
1183.6619 1192.4036 1206.7671
1339.9106 1360.7117 1390.1213
1489.7474 1519.5455 1641.5652
1642.6085 3193.0024 3205.1246
3213.3231 3228.0415 3229.0878

Piperidine 2

Gaussian 16: ES64L-G16RevB.01 20-Dec-2017

# B3LYP/6-31G(d,p) gfprint gfinput
scf=(direct,tight,maxcycle=300,xqc)
opt=(maxcycle=250) freq=noraman
eb-2021\0\\# B3LYP/6-31G(d,p) gfprint gfinput
scf=(direct,tight,maxcyc
le=300,xqc) opt=(maxcycle=250)
freq=noraman\\piperidine_1.xyz\\0,1\C,0

Full point group C1 NOp 1
Stoichiometry C5H11N Framework group
C1[X(C5H11N)]

Num atoms: 17
Charge = 0 Multiplicity = 1

SCF = -251.921209206 l Predicted change in
Energy=-5.898907D-08

Optimization completed.
Maximum Force 0.000025
0.000450 YES
RMS Force 0.000009 0.000300
YES
Maximum Displacement 0.001391
0.001800 YES
RMS Displacement 0.000226
0.001200 YES

Atom Type	X	Y	Z
C	0.182027	-1.541435	-2.415288
C	-0.552702	-0.269394	-1.978216
N	-1.499237	-0.591815	-0.908119
C	-0.827292	-1.110951	0.285186
C	-0.103770	-2.417337	-0.059515
C	0.882829	-2.200747	-1.217350
H	0.906243	-1.301157	-3.202681
H	-0.549896	-2.237761	-2.842768
H	-1.110507	0.156963	-2.819550
H	0.197912	0.485974	-1.668142
H	-0.089693	-0.395470	0.702537
H	-1.581272	-1.285822	1.060882
H	0.417451	-2.799188	0.826333
H	-0.852483	-3.165121	-0.348599
H	1.342980	-3.150378	-1.514609
H	1.700491	-1.549611	-0.876054
H	-2.038783	0.234751	-0.666249

(Coordinates in Angstroms)

Statistical Thermodynamic Analysis
Temperature 298.150 Kelvin. Pressure
1.00000 Atm.

SCF = -251.921209206 l Predicted change in
Energy=-5.898907D-08
Zero-point correction (ZPE) = -251.761828206
0.159381
Internal Energy (U) = -251.756275206
0.164934
Enthalpy (H) = -251.755331206   0.165878
Gibbs Free Energy (G) = -251.790457206
0.130752

Frequencies
245.1791 249.9071 400.6382
434.9666 452.0246 546.8265
771.9084 824.5768 824.6769
876.2796 882.2004 914.0905
986.1449 1052.5737 1065.1197
1068.3857 1139.3147 1173.9055
1174.3074 1188.4960 1295.0131
1299.5962 1319.6489 1354.3236
1370.8827 1389.4300 1390.5074
1435.7781 1483.8351 1492.9420
1496.9048 1507.6941 1511.9698
1525.1286 2902.5587 2909.2226
3019.0113 3038.7140 3038.9539
3069.2654 3069.2806 3072.9797
3083.3014 3088.5598 3523.1643

DABCO

Gaussian 16: ES64L-G16RevB.01 20-Dec-
2017

# B3LYP/6-31G(d,p) gfprint gfinput
scf=(direct,tight,maxcycle=300,xqc)
opt=(maxcycle=250) freq=noraman
ep-2020\0\\# B3LYP/6-31G(d,p) gfprint gfinput
scf=(direct,tight,maxcyc
le=300,xqc) opt=(maxcycle=250)
freq=noraman\\DABCO_1.xyz\\0,1\N,-1.252

Full point group C1 NOp 1
Stoichiometry C6H12N2 Framework group
C1[X(C6H12N2)]

Num atoms: 20
Charge = 0 Multiplicity = 1

SCF = -345.345304817 l Predicted change in Energy=-1.514776D-07

Optimization completed.
Maximum Force 0.000120 0.000450 YES
RMS Force 0.000022 0.000300 YES
Maximum Displacement 0.000540 0.001800 YES
RMS Displacement 0.000147 0.001200 YES

Atom Coordinates (in Angstroms)
Type X Y Z

N -1.252603 -0.637235 -0.007307
C -0.259243 -1.478797 0.683397
C 1.135885 -0.769088 0.691453
N 1.046927 0.532566 0.006000
C 0.049204 1.367026 0.699110
C -1.345975 0.657290 0.691026
C -0.796058 -0.399977 -1.388335
C 0.599040 0.309746 -1.380269
H -0.620578 -1.666445 1.701041
H -0.215350 -2.445642 0.168943
H 1.490977 -0.592317 1.713234
H 1.896186 -1.371477 0.181112
H 0.404082 1.544151 1.720901
H 0.008519 2.339112 0.194364
H -1.707500 0.469997 1.708665
H -2.103036 1.264938 0.182109
H -0.752216 -1.366564 -1.903275
H -1.553034 0.207418 -1.897665
H 1.359200 -0.292416 -1.891080
H 0.558472 1.281615 -1.885426

Statistical Thermodynamic Analysis
Temperature 298.150 Kelvin. Pressure 1.00000 Atm.

SCF = -345.345304817 l Predicted change in Energy=-1.514776D-07
Zero-point correction (ZPE) = -345.161563817 0.183741
Internal Energy (U) = -345.155298817 0.190006
Enthalpy (H) = -345.154354817 0.19095
Gibbs Free Energy (G) = -345.191757817 0.153547

Frequencies
99.6468 339.8729 341.0873
428.2693 428.4628 589.5811
589.7977 607.1382 762.7232
811.5921 812.8097 834.0938
834.3358 893.5936 894.0643
961.3266 996.0778 1030.8212
1040.7460 1040.8827 1078.9135
1079.2156 1198.4314 1209.7220
1209.9022 1268.4533 1332.3653
1332.4441 1335.7369 1335.9221
1350.1455 1350.3604 1356.7667
1356.8454 1367.5565 1392.4034
1493.7096 1494.0177 1503.8212
1503.8973 1507.1390 1514.9007
3033.3706 3033.4094 3036.0099
3040.7129 3040.7673 3048.7294
3071.6776 3074.7065 3074.7140
3091.7762 3098.6913 3098.7015

Sulfonamide product

Gaussian 16: ES64L-G16RevB.01 20-Dec-2017

B3LYP/6-31G(d,p) gfprint gfinput scf=(direct,tight,maxcycle=300,xqc) opt=(maxcycle=250) freq=noraman \20-Jul-2020\0\\# B3LYP/6-31G(d,p) gfprint gfinput scf=(direct,tight,m axcycle=300,xqc) opt=(maxcycle=250) freq=noraman\\PhSO2NHPh_1.xyz\\0,1

Full point group C1 NOp 1
Stoichiometry C12H11NO2S Framework group C1[X(C12H11NO2S)]

Num atoms: 27
Charge = 0 Multiplicity = 1

SCF = -1067.23918254 l Predicted change in Energy=-5.192702D-10

Optimization completed.
Maximum Force 0.000005 0.000450 YES
RMS Force 0.000001 0.000300 YES
Maximum Displacement 0.000475 0.001800 YES
RMS Displacement 0.000103 0.001200 YES

Atom Coordinates (in Angstroms)
Type X Y Z

C	-0.807138	-0.525884	-0.033485
C	-1.108302	0.338342	-1.084966
C	-0.955532	1.721674	-0.923910
C	-0.508310	2.235204	0.301508
C	-0.239575	1.362497	1.354615
C	-0.380338	-0.017551	1.193793
H	-0.919219	-1.596969	-0.173127
H	-1.460137	-0.057734	-2.034231
N	-1.201823	2.588113	-2.026127
H	-0.374503	3.304266	0.411917
H	0.102975	1.766364	2.302778
H	-0.153949	-0.690314	2.015121
H	-1.278687	2.125054	-2.926403
S	-2.305666	3.881275	-1.971886
O	-2.315986	4.387156	-3.344947
O	-1.939268	4.713743	-0.827229
C	-6.391870	2.024474	-1.119859
C	-5.549143	2.361633	-0.058575
C	-4.305465	2.941695	-0.307306
C	-3.920605	3.171314	-1.630507
C	-4.755592	2.843842	-2.701440
C	-5.998222	2.268871	-2.437537
H	-7.360929	1.577383	-0.919416
H	-5.862058	2.180392	0.964986
H	-3.648628	3.228668	0.505509
H	-4.438657	3.060053	-3.715767
H	-6.661073	2.018939	-3.260211

Statistical Thermodynamic Analysis
Temperature 298.150 Kelvin. Pressure 1.00000 Atm.

SCF = -1067.23918254 l Predicted change in Energy=-5.192702D-10
Zero-point correction (ZPE) = -1067.03052054 0.208662
Internal Energy (U) = -1067.01709654 0.222086
Enthalpy (H) = -1067.01615254 0.22303
Gibbs Free Energy (G) = -1067.07270554 0.166477

Frequencies
23.8818 31.5075 40.2084
108.6482 123.8121 183.2078
214.4443 273.1954 284.9505
306.5491 347.0392 407.1507
413.6347 421.0097 464.8339
496.9350 528.0057 545.5393
590.5231 626.1801 631.4840
633.2066 699.0109 705.5637
721.9648 760.7797 768.4470
823.1299 845.7352 861.1967
877.2094 918.4094 943.3396
970.7565 984.4931 997.3571
1009.5813 1013.5480 1015.4189
1048.0555 1056.2370 1087.5393
1106.8018 1110.7715 1143.9522
1187.5168 1190.3084 1204.9974
1205.7584 1247.4577 1296.6569
1338.2800 1346.6664 1361.8537
1366.8624 1418.0658 1487.2220
1510.8955 1519.0483 1536.9525
1640.3763 1641.5845 1644.4042
1657.0331 3174.6529 3186.3842
3187.8273 3195.6657 3199.3391
3208.9020 3209.1601 3222.5301
3230.3674 3236.7229 3573.4375

$Ca(NTf_2)_2(THF)_2$ 4a

Gaussian 16: ES64L-G16RevB.01 20-Dec-2017

B3LYP/6-31G(d,p) gfprint gfinput scf=(direct,tight,maxcycle=300,xqc) opt=(maxcycle=250) freq=noraman
MAN.EDU\26-Sep-2020\0\\# B3LYP/6-31G(d,p) gfprint gfinput scf=(direct, tight,maxcycle=300,xqc) opt=(maxcycle=250) freq=noraman\\ca2plus_2NTf2

Full point group C1 NOp 1
Stoichiometry C12H16CaF12N2O10S4
Framework group
C1[X(C12H16CaF12N2O10S4)]

Num atoms: 57
Charge = 0 Multiplicity = 1

SCF = -4796.96726569 l Predicted change in Energy=-1.400723D-08

Optimization completed on the basis of negligible forces.

Maximum Force 0.000004 0.000450 YES
RMS Force 0.000001 0.000300 YES
Maximum Displacement 0.016181 0.001800 NO
RMS Displacement 0.002184 0.001200 NO

Atom Type	Coordinates (in Angstroms) X	Y	Z
Ca	7.595338	0.539688	0.153597
S	9.160293	0.448562	-3.010697
O	9.124304	0.986003	-1.611637
N	7.723536	0.497283	-3.748334
C	9.980571	1.824113	-3.974246
O	9.949495	-0.766782	-3.207673
S	6.395325	-0.181524	-3.159291
O	5.213765	0.484358	-3.693600
C	6.373267	-1.927979	-3.834362
O	6.444337	-0.421026	-1.682050
F	6.449820	-1.904337	-5.160518
F	7.412476	-2.605480	-3.335893
F	5.239462	-2.520112	-3.458625
F	9.339024	2.977048	-3.765794
F	11.232504	1.932555	-3.529326
F	9.987404	1.540965	-5.270619
S	6.462193	-0.319756	3.395604
O	6.159505	0.039832	1.972849
N	7.478964	0.679909	4.147261
C	4.855576	0.051035	4.278269
O	6.767180	-1.722797	3.661520
F	3.893845	-0.688153	3.724274
F	4.967755	-0.251130	5.566948
F	4.553140	1.344127	4.143986
S	8.388329	1.796892	3.442314
O	8.702722	1.501278	2.009622
C	7.313109	3.327514	3.309846
O	9.495810	2.162762	4.308313
F	6.952725	3.744961	4.518000
F	7.997848	4.292444	2.687862
F	6.213609	3.050125	2.590533
C	9.285352	-3.855677	0.613321
C	10.352618	-3.036719	1.356686
C	10.171371	-1.653976	0.741292
O	8.738683	-1.537632	0.512952
C	8.125753	-2.863845	0.488099
H	9.650800	-4.147340	-0.376758
H	8.990069	-4.761471	1.148295
H	11.365345	-3.422966	1.216987
H	10.137139	-3.005600	2.429615
H	10.469745	-0.830272	1.394742
H	10.687271	-1.561013	-0.221571
H	7.570521	-2.962542	-0.448677
H	7.437083	-2.922607	1.336071
C	4.994457	3.675804	-1.945334
C	6.410022	4.292456	-2.128554
C	7.190904	3.819912	-0.895364
O	6.525161	2.603147	-0.483339
C	5.107613	2.839327	-0.659782
H	4.217506	4.438116	-1.847852
H	4.741592	3.037364	-2.793803
H	6.390188	5.383240	-2.192168
H	6.878264	3.909833	-3.037957
H	8.233217	3.569569	-1.100140
H	7.145456	4.541024	-0.069394
H	4.732754	3.377178	0.219709
H	4.618686	1.865215	-0.722207

Statistical Thermodynamic Analysis
Temperature 298.150 Kelvin. Pressure 1.00000 Atm.

SCF = -4796.96726569 l Predicted change in Energy=-1.400723D-08
Zero-point correction (ZPE) = -4796.61975169 0.347514
Internal Energy (U) = -4796.57381369 0.393452
Enthalpy (H) = -4796.57286969 0.394396
Gibbs Free Energy (G) = -4796.71354669 0.253719

Frequencies
4.1190 10.1048 13.3723
16.3033 20.7633 21.4845
26.1883 32.9595 34.4384
35.7635 37.9391 38.9237
40.0899 43.4738 45.5134
49.9167 58.3673 60.1868
61.4026 65.6498 68.0317
78.5159 83.8505 96.7876
104.9993 108.3894 115.3058
123.8990 135.1071 140.3938
162.4036 169.1054 176.8635
182.2875 198.0175 202.2622
213.1264 232.5879 235.6715
256.0917 260.9335 268.3273
269.0043 279.3733 286.0503
287.7165 291.8112 295.7234
304.6037 313.8170 317.6926
328.4410 332.9586 346.6650
362.4018 391.0716 404.9391
423.8123 444.3334 493.5023
500.6143 504.8111 515.6155
536.8477 537.2809 549.2820
550.8939 554.2572 560.4638
563.2497 566.2779 580.9591
583.4577 590.8465 630.4821
639.1676 646.5183 665.1039
690.3284 717.0721 717.5470
761.0782 765.1826 782.8776
786.6977 814.6502 856.4006
867.1172 881.0591 890.8542
908.2777 922.2191 923.3389
930.8704 952.6312 970.8203
980.4904 1010.4413 1012.5164
1048.1501 1049.8586 1056.2523
1062.2937 1065.9160 1071.5375
1086.0601 1095.4671 1154.7564
1176.0249 1196.6989 1201.0294
1201.4475 1206.7180 1207.2323
1208.6151 1223.1808 1230.6120
1237.9018 1243.2773 1250.1086
1253.3989 1265.7849 1268.5291
1269.9358 1270.7390 1271.7326
1271.8595 1275.6440 1284.5177
1287.0201 1289.6701 1298.6554
1318.2176 1329.8152 1331.6284
1335.8566 1360.4346 1378.1707
1386.6059 1409.2576 1416.5808
1500.7332 1504.0244 1510.5059
1519.4182 1520.7355 1533.5845
1540.2524 1546.2650 3043.1568
3047.4013 3053.0624 3068.3971
3071.4063 3073.4707 3085.4043
3092.9465 3119.6984 3119.8797
3126.2015 3129.9691 3133.4684
3138.3764 3141.6665 3151.3703

$Ca(NTf_2)_2(THF)_3$ 4b

Gaussian 16: ES64L-G16RevB.01 20-Dec-2017

B3LYP/6-31G(d,p) gfprint gfinput scf=(direct,tight,maxcycle=300,xqc) opt=(maxcycle=250) freq=noraman
MAN.EDU\28-Sep-2020\0\\# B3LYP/6-31G(d,p) gfprint gfinput scf=(direct, tight,maxcycle=300,xqc) opt=(maxcycle=250) freq=noraman\\ca2plus_2NTf2

Full point group C1 NOp 1
Stoichiometry C16H24CaF12N2O11S4
Framework group C1[X(C16H24CaF12N2O11S4)]

Num atoms: 70
Charge = 0 Multiplicity = 1

SCF = -5029.44846463 l Predicted change in Energy=-9.270541D-09

Optimization completed.
Maximum Force 0.000013 0.000450 YES
RMS Force 0.000002 0.000300 YES
Maximum Displacement 0.001636 0.001800 YES
RMS Displacement 0.000243 0.001200 YES

Atom Coordinates (in Angstroms)
Type X Y Z

Ca 6.976379 2.537845 0.509995
S 5.825587 1.758332 3.919227

O	6.507255	2.328450	5.077398
N	6.213669	0.211051	3.744750
C	4.011592	1.732356	4.375215
O	5.838623	2.565889	2.665771
S	6.530891	-0.566495	2.371794
O	7.696780	-1.428459	2.530811
C	5.072418	-1.718053	2.161945
O	6.464637	0.270042	1.139161
F	4.935009	-2.487888	3.237260
F	3.953347	-1.002792	1.983515
F	5.280149	-2.478462	1.088052
F	3.290451	1.245646	3.356626
F	3.815856	0.979065	5.452703
F	3.616278	2.983082	4.626118
S	7.285563	0.991927	-2.838394
O	6.531913	1.572176	-1.689360
N	8.592849	1.828160	-3.275912
C	8.014948	-0.572489	-2.116839
O	6.515444	0.599304	-4.009138
F	8.809260	-0.239032	-1.084560
F	7.030862	-1.354558	-1.677386
F	8.729518	-1.219965	-3.029636
S	9.285696	2.980392	-2.403106
O	10.727530	2.997356	-2.603232
C	8.638276	4.549110	-3.187976
O	8.797737	3.102545	-0.995960
F	9.002633	4.629350	-4.460491
F	7.297205	4.575263	-3.106246
F	9.122754	5.607728	-2.520330
C	2.863972	2.060229	-1.168396
C	2.936529	3.480739	-1.747631
C	4.335022	3.936060	-1.322430
O	4.650325	3.193401	-0.110131
C	3.596079	2.224244	0.158141
H	1.840723	1.698962	-1.037549
H	3.403785	1.357690	-1.810641
H	2.167943	4.118058	-1.296735
H	2.809828	3.511088	-2.832480
H	5.087958	3.687016	-2.074416
H	4.391274	5.005080	-1.094020
H	2.944324	2.625529	0.942974
H	4.061407	1.307758	0.523141
C	7.579018	7.150299	0.303267
C	6.200494	7.128734	1.018443
C	6.108215	5.712118	1.625539
O	7.144810	4.943750	0.970145
C	8.246424	5.854765	0.770685
H	7.456144	7.129786	-0.782760
H	8.174380	8.031124	0.556106
H	5.388707	7.314271	0.310443
H	6.131881	7.889375	1.800364
H	6.305359	5.720155	2.704415
H	5.165104	5.196643	1.443967
H	8.921511	5.403072	0.045871
H	8.772267	5.993265	1.726029
C	9.984416	1.175706	1.651516
C	11.027991	1.305046	2.772140
C	10.263488	2.037224	3.887564
C	9.353843	2.958862	3.086843
O	8.939972	2.156010	1.954046
H	10.392105	1.400547	0.662471
H	9.507296	0.193444	1.638342
H	11.879709	1.905863	2.436734
H	11.407019	0.329736	3.086058
H	9.656940	1.339803	4.473377
H	10.919253	2.584388	4.569893
H	8.455710	3.271894	3.620789
H	9.896663	3.843889	2.725410

Statistical Thermodynamic Analysis
Temperature 298.150 Kelvin. Pressure 1.00000 Atm.

SCF = -5029.44846463 l Predicted change in Energy=-9.270541D-09
Zero-point correction (ZPE) = -5028.98175963 0.466705
Internal Energy (U) = -5028.92954463 0.51892
Enthalpy (H) = -5028.92859963 0.519865
Gibbs Free Energy (G) = -5029.08098163 0.367483

Frequencies
11.1089 14.7637 17.4463
18.9124 20.8928 25.5724
27.3696 30.6936 33.9880
34.1918 35.3243 37.8169
40.0217 42.7134 46.5306
49.2100 51.2370 53.1295
56.0402 64.8944 67.2356
71.5581 75.7678 79.3697
84.5045 88.8409 91.8043
103.2678 104.5121 110.7680
112.6105 120.2295 122.3694
125.7006 140.4203 145.2289
173.6838 186.7220 194.7771
196.6262 203.0871 204.9162

211.0231 212.5095 228.3861
242.1689 244.8372 257.4772
265.6367 268.4096 276.4233
280.2274 282.8004 286.9347
293.3823 295.5912 300.9737
308.6620 317.4426 322.6369
325.1456 328.1901 334.3923
392.6374 396.3007 396.8048
409.5378 494.6311 499.4625
521.6475 521.9316 537.9423
539.7464 548.3338 549.3090
561.0560 561.8016 563.5499
573.5968 579.3405 580.3241
587.8271 593.6340 613.5855
629.8068 630.6038 678.5133
680.8984 692.2682 715.6461
720.9198 759.3792 762.1358
780.0052 781.7659 806.8080
842.3642 850.2075 872.5354
878.0718 884.0026 889.9506
891.0451 909.7178 922.3459
925.5597 928.2624 933.0260
933.1584 947.1734 969.3118
977.8704 980.3109 1015.0679
1017.4488 1047.9287 1050.1501
1053.5045 1054.0376 1056.9421
1065.7525 1075.4424 1086.3205
1088.7740 1099.8324 1155.3749
1172.8420 1173.3680 1195.6160
1197.6127 1198.9673 1205.1479
1207.9257 1209.1104 1213.5608
1215.1587 1221.8870 1225.5969
1228.5091 1235.6300 1242.3926
1248.8097 1260.2607 1262.2419
1263.9677 1267.5406 1268.7117
1270.6720 1275.0793 1277.5920
1277.9329 1286.4303 1290.5969
1300.0421 1304.1623 1319.2837
1321.6369 1329.9196 1331.6268
1331.9528 1354.1433 1361.6670
1375.5591 1382.5416 1386.5814
1408.8667 1415.2193 1417.1740
1500.6022 1502.2588 1503.7254
1510.3443 1513.7569 1520.2961
1524.5492 1530.1703 1532.2149
1542.2760 1543.3034 1547.3355
3015.7643 3023.3689 3048.9802
3052.9647 3060.3282 3067.2237
3068.2869 3077.1401 3077.2698
3077.5817 3081.4687 3086.2112
3116.3186 3116.8900 3121.8136
3128.5107 3131.3859 3135.6085
3135.7460 3143.4438 3147.2982
3148.8799 3154.4338 3176.3174

$Ca(NTf_2)_2(THF)_4$ 4c

Gaussian 16: ES64L-G16RevB.01 20-Dec-2017

B3LYP/6-31G(d,p) gfprint gfinput scf=(direct,tight,maxcycle=300,xqc) opt=(maxcycle=250) freq=noraman
AN.EDU\03-Mar-2021\0\\# B3LYP/6-31G(d,p) gfprint gfinput scf=(direct,t ight,maxcycle=300,xqc) opt=(maxcycle=250) freq=noraman\\000_ca_2ntf2_4

Full point group C1 NOp 1
Stoichiometry C20H32CaF12N2O12S4
Framework group C1[X(C20H32CaF12N2O12S4)]

Num atoms: 83
Charge = 0 Multiplicity = 1

SCF = -5261.91985400 l Predicted change in Energy=-2.987420D-08

Optimization completed.
Maximum Force 0.000017 0.000450 YES
RMS Force 0.000003 0.000300 YES
Maximum Displacement 0.001396 0.001800 YES
RMS Displacement 0.000345 0.001200 YES

Atom Coordinates (in Angstroms)
Type X Y Z

Ca -1.796053 0.818008 -0.758004
S -3.773020 -2.331808 -1.339688
O -3.806622 -3.372156 -0.311398
O -2.554979 -1.476203 -1.384859
N -5.161941 -1.531316 -1.541735

S -5.472924 -0.100730 -0.886371
O -6.521751 0.576389 -1.641064
O -4.264691 0.688607 -0.528602
C -6.214454 -0.484071 0.788244
F -7.373906 -1.118548 0.652455
F -5.369083 -1.248692 1.493092
F -6.408330 0.663261 1.449900
C -3.728931 -3.226771 -2.978979
F -2.601807 -3.947714 -3.013051
F -4.775794 -4.032686 -3.108518
F -3.708064 -2.354686 -3.994899
S 0.747702 2.885926 1.148057
O 1.550748 2.457995 2.288267
O -0.528990 2.166071 0.897597
N 1.679620 3.055849 -0.146337
S 1.129881 3.005362 -1.664866
O 0.900074 4.313049 -2.279665
O 0.099317 1.959617 -1.914064
C 2.655301 2.308302 -2.487898
F 2.959588 1.106322 -1.982216
F 2.379776 2.165526 -3.789832
F 3.690530 3.126000 -2.340015
C 0.161961 4.615607 1.555808
F -0.711219 4.550669 2.568266
F 1.186542 5.386894 1.903200
F -0.453971 5.147502 0.490994
C 1.832456 -2.112104 0.535490
C 1.240690 -0.692038 0.450112
C 0.642053 -1.680686 -1.539041
C 1.356569 -2.802273 -0.772623
H 2.921846 -2.075786 0.614953
H 1.457488 -2.644721 1.413433
H 1.985381 0.030005 0.097911
H 0.808240 -0.323966 1.380113
H -0.227160 -2.014418 -2.106344
H 1.335348 -1.141211 -2.199117
H 2.181614 -3.224722 -1.351880
H 0.656980 -3.611954 -0.549134
O 0.171463 -0.781613 -0.517534
C -3.388105 1.682864 -5.245925
C -1.898265 1.344433 -5.528432
C -1.473351 0.516339 -4.307941
O -2.332491 0.965956 -3.243518
C -3.642042 1.120306 -3.834155
H -3.552907 2.763121 -5.276773
H -4.064168 1.227471 -5.973908
H -1.300562 2.257495 -5.591861
H -1.760139 0.789967 -6.460200
H -1.640365 -0.556990 -4.473652
H -0.444115 0.687661 -3.991614
H -4.218466 1.775433 -3.181517
H -4.135143 0.143070 -3.876817
C -1.476684 -1.406271 3.449552
C -1.697828 0.082733 3.823828
C -2.602949 0.595379 2.701850
O -2.191666 -0.156182 1.546054
C -1.993778 -1.516037 1.996901
H -0.420488 -1.678093 3.526050
H -2.034773 -2.078359 4.106990
H -0.751381 0.629941 3.809674
H -2.149668 0.210032 4.811053
H -3.663693 0.394164 2.905738
H -2.466215 1.653705 2.476124
H -1.297703 -1.981268 1.299723
H -2.946050 -2.052960 1.946193
C -4.720308 4.419388 -0.906665
C -3.665935 3.755511 -0.018719
O -2.746666 3.157622 -0.950019
C -2.557092 4.124363 -2.008837
C -3.908596 4.860850 -2.152598
H -5.482417 3.685212 -1.181573
H -5.217254 5.254291 -0.405705
H -4.062152 2.960423 0.614171
H -3.129360 4.488382 0.599527
H -1.751007 4.809587 -1.728888
H -2.245691 3.567678 -2.892098
H -4.424895 4.585105 -3.076009
H -3.754915 5.943088 -2.175663

Statistical Thermodynamic Analysis
Temperature 298.150 Kelvin. Pressure 1.00000 Atm.

SCF = -5261.91985400 l Predicted change in Energy=-2.987420D-08
Zero-point correction (ZPE) = -5261.334213 0.585641
Internal Energy (U) = -5261.275048 0.644806
Enthalpy (H) = -5261.274104 0.64575
Gibbs Free Energy (G) = -5261.443259 0.476595

Frequencies
12.5764 17.7742 18.1121
20.1942 26.9807 27.1809
28.2759 31.5107 33.9060
36.0695 36.1882 36.3675

36.6260 38.7342 39.4013
42.5297 42.5513 46.0382
48.1373 49.6746 53.1549
57.7207 60.4852 61.5941
64.3818 67.9705 71.4235
74.7612 76.7486 86.0582
91.8338 95.8238 99.1113
103.5156 104.6549 105.3676
111.6974 112.3741 118.4209
118.4764 125.2173 132.4406
143.4950 146.9169 174.1799
175.7874 187.1853 188.5293
193.9868 203.7682 212.0133
219.3358 220.9527 227.4384
238.6835 274.4253 275.2159
282.9393 284.1996 292.2042
292.9901 294.7390 296.3009
297.3396 301.7328 315.4168
315.6366 321.5855 322.0369
343.8751 344.1535 392.4869
394.3532 424.8605 425.4663
493.0481 493.8487 511.1160
511.2385 538.0105 538.0146
549.6033 549.7056 554.0485
554.1599 560.5138 561.3755
588.7888 590.6589 620.9025
620.9888 637.2713 637.5728
642.0687 642.2666 667.6928
667.7738 684.0477 684.0934
718.5264 718.6138 761.4936
761.5920 781.5318 781.5403
804.6792 804.8028 809.9202
810.0968 871.2164 871.7693
874.7766 875.3579 910.5872
910.9202 911.2501 911.2938
923.9861 924.8682 929.0543
932.7808 944.8010 945.9847
949.7698 950.9460 970.6749
971.4014 972.3427 972.5593
1015.0067 1017.1520 1054.7874
1056.3869 1061.6835 1061.8756
1065.8878 1071.2911 1072.9959
1075.2040 1078.2446 1086.7997
1099.8168 1106.5127 1152.9754
1153.4382 1153.7947 1154.1153
1203.9285 1203.9513 1215.4716
1215.7374 1224.2118 1224.5059
1226.9467 1227.0342 1227.4213
1228.3330 1230.1600 1231.2639
1241.9124 1242.4971 1244.5614
1245.0610 1254.6532 1255.0339
1261.5596 1262.9791 1263.7047
1264.2161 1265.2441 1266.3756
1281.8694 1282.0172 1286.7548
1288.3708 1293.6274 1293.9859
1302.0340 1307.2021 1318.7489
1318.9725 1321.7527 1322.1964
1333.8998 1334.4731 1340.8521
1341.4537 1376.8818 1378.0878
1378.9000 1379.5457 1412.4514
1413.6751 1416.0933 1416.4874
1502.4850 1502.6315 1502.8544
1502.8581 1515.0552 1515.0831
1517.1745 1518.3344 1521.2392
1521.4172 1522.3334 1523.4922
1537.8922 1538.6583 1543.3210
1543.6204 3027.2291 3028.1094
3030.5310 3030.8562 3070.0808
3070.1813 3072.8887 3072.9057
3074.5944 3074.6163 3078.6554
3078.7206 3083.4779 3083.6840
3084.2848 3084.4318 3112.1146
3112.1223 3114.5361 3114.5851
3131.7924 3131.9835 3132.7842
3132.8638 3144.5617 3146.1739
3152.1831 3152.8767 3160.6116
3160.7093 3161.9459 3161.9511

$Ca(NTf_2)_2(PhSO_2F)(THF)$ 5a

Gaussian 16: ES64L-G16RevB.01 20-Dec-2017

B3LYP/6-31G(d,p) gfprint gfinput scf=(direct,tight,maxcycle=300,xqc) opt=(maxcycle=250) freq=noraman MAN.EDU\30-Sep-2020\0\\# B3LYP/6-31G(d,p) gfprint gfinput scf=(direct, tight,maxcycle=300,xqc) opt=(maxcycle=250) freq=noraman\\ca2plus_2NTf2

Full point group C1 NOp 1
Stoichiometry C14H13CaF13N2O11S5
Framework group C1[X(C14H13CaF13N2O11S5)]

Num atoms: 59
Charge = 0 Multiplicity = 1

SCF = -5444.57341146 l Predicted change in Energy=-1.199336D-08

Optimization completed.
Maximum Force 0.000015 0.000450 YES
RMS Force 0.000003 0.000300 YES
Maximum Displacement 0.001738 0.001800 YES
RMS Displacement 0.000447 0.001200 YES

Atom Coordinates (in Angstroms)
Type X Y Z

Ca 7.742691 -0.617856 4.218628
S 10.106600 0.053919 1.510267
O 10.214640 1.067058 0.466088
N 9.954810 -1.422515 0.895967
C 11.765740 -0.038633 2.367581
O 9.169668 0.373451 2.631773
S 8.926066 -2.555005 1.380392
O 8.376803 -2.366357 2.759663
C 7.417399 -2.247757 0.317646
O 9.421545 -3.877633 1.040280
F 6.439987 -3.077790 0.688227
F 7.004301 -0.980839 0.508423
F 7.698249 -2.430738 -0.965310
F 11.717028 -0.993325 3.300284
F 12.723143 -0.312971 1.489983
F 12.009547 1.137236 2.948146
S 9.371299 -0.413598 7.469581
O 9.126414 -0.957080 6.098480
N 8.962830 1.126395 7.677971
C 8.089960 -1.310937 8.495503
O 10.659274 -0.701307 8.077378
F 8.209454 -0.995451 9.777371
F 6.859714 -0.975760 8.066552
F 8.247939 -2.631647 8.343978
S 8.134405 2.042824 6.652972
O 7.376694 3.061167 7.362380
C 9.475528 2.929910 5.696681
O 7.411931 1.292493 5.576005
F 10.195254 3.696150 6.506728
F 10.268646 2.020590 5.114092
F 8.909231 3.685772 4.751250
S 5.042212 1.598618 2.939450
O 5.856526 0.379118 3.052076
F 4.228733 1.360557 1.568573
O 4.067537 1.919867 3.958046
C 7.651053 5.129993 1.881206
C 7.955720 3.869866 1.359620
C 7.154098 2.775217 1.671111
C 6.055896 2.977522 2.512142
C 5.731833 4.227726 3.045558
C 6.547284 5.309237 2.719212
H 8.283488 5.978363 1.638799
H 8.822251 3.722763 0.724081
H 7.393292 1.792506 1.286211
H 4.879574 4.339393 3.705576
H 6.323953 6.289333 3.127199
C 5.055583 -4.101024 5.908864
C 4.397848 -2.882845 6.576071
C 4.797867 -1.748954 5.636721
O 6.114011 -2.120092 5.135614
C 6.384735 -3.526578 5.421842
H 5.198463 -4.941950 6.591747
H 4.453026 -4.447697 5.062630
H 4.823207 -2.714219 7.570264
H 3.313623 -2.975562 6.674734
H 4.115319 -1.661578 4.783811
H 4.882993 -0.775548 6.125392
H 7.155887 -3.569942 6.197467
H 6.765097 -3.988356 4.507822

Statistical Thermodynamic Analysis
Temperature 298.150 Kelvin. Pressure 1.00000 Atm.

SCF = -5444.57341146 l Predicted change in Energy=-1.199336D-08
Zero-point correction (ZPE) = -5444.24033146 0.33308
Internal Energy (U) = -5444.19092546 0.382486
Enthalpy (H) = -5444.18998146 0.38343
Gibbs Free Energy (G) = -5444.33707046 0.236341

Frequencies
6.1697 9.7754 15.7894
17.3694 21.2813 23.1816
24.3933 25.2305 29.8929
30.5795 34.5600 37.3861
39.2133 40.0429 43.2325

44.6956 50.2688 52.6893
56.8646 61.5589 62.1925
77.4251 78.4411 105.5158
107.5952 110.8460 115.3535
121.4920 128.3223 133.1567
154.3081 183.1406 192.3431
195.6108 199.0204 202.6683
204.0184 210.6862 212.7861
214.8298 241.3609 257.3870
266.0770 267.0407 268.9795
275.6082 285.4565 287.2699
291.8702 294.2352 295.7902
308.8449 311.0802 312.8891
323.5438 324.7776 328.1405
328.8731 345.7676 394.4790
396.1236 397.9921 403.2473
409.4530 432.5539 485.0572
499.8793 503.5284 515.4473
522.9565 523.3440 537.6744
538.3154 548.5753 549.2207
563.4036 566.4834 573.1089
576.1941 579.1394 579.3176
589.2822 594.9688 608.4882
613.4588 622.8443 685.8717
687.1463 718.0431 719.2326
720.8971 759.9196 761.7090
762.5755 779.7207 783.0001
811.5064 855.6151 856.9686
879.0748 889.2331 920.4063
930.3679 947.2089 975.5397
992.5451 1011.5522 1016.9033
1021.1089 1022.4216 1043.8571
1046.4951 1052.6183 1069.3664
1078.5998 1082.1741 1088.9937
1093.4197 1114.5141 1163.2302
1174.0786 1194.2471 1197.1727
1199.7950 1200.1309 1202.9543
1205.9818 1207.3597 1211.8781
1219.9681 1229.6834 1234.3675
1250.8728 1268.6089 1269.5663
1271.3161 1272.5924 1275.0906
1275.5806 1293.0089 1303.9857
1315.2765 1325.0920 1330.8311
1344.1982 1355.0970 1360.5119
1370.9078 1385.1336 1414.8797
1490.2271 1501.8800 1510.0997
1515.5516 1532.2627 1546.7194
1635.3487 1639.8801 3055.5907
3068.5045 3072.8883 3077.4934
3122.8594 3124.5582 3133.2603
3140.5746 3199.5701 3214.1004
3222.8097 3233.4131 3252.0877

$Ca(NTf_2)_2(PhSO_2F)(THF)_2$ 5b

Gaussian 16: ES64L-G16RevB.01 20-Dec-2017

B3LYP/6-31G(d,p) gfprint gfinput scf=(direct,tight,maxcycle=300,xqc) opt=(maxcycle=250) freq=noraman MAN.EDU\14-Oct-2020\0\\# B3LYP/6-31G(d,p) gfprint gfinput scf=(direct, tight,maxcycle=300,xqc) opt=(maxcycle=250) freq=noraman\\ca2plus_2NTf2

Full point group C1 NOp 1
Stoichiometry C18H21CaF13N2O12S5
Framework group C1[X(C18H21CaF13N2O12S5)]

Num atoms: 72
Charge = 0 Multiplicity = 1

SCF = -5677.05819745 l Predicted change in Energy=-9.251751D-09

Optimization completed.
Maximum Force 0.000008 0.000450 YES
RMS Force 0.000001 0.000300 YES
Maximum Displacement 0.001357 0.001800 YES
RMS Displacement 0.000301 0.001200 YES

Atom Type	Coordinates (in Angstroms) X	Y	Z
Ca	8.897471	-1.202281	5.936461
S	9.659439	2.407109	6.136856
O	8.759262	1.218070	6.137903
N	10.997192	2.241034	7.012083
C	10.316302	2.427761	4.385953
O	9.046770	3.707235	6.367975

S	11.564155	0.854319	7.590242
O	13.017516	0.796860	7.503478
C	11.151851	0.986249	9.407819
O	10.819192	-0.369438	7.169787
F	11.564907	-0.126668	10.029725
F	11.748680	2.040381	9.949736
F	9.824223	1.095595	9.558815
F	10.978884	1.278183	4.161327
F	9.299567	2.520423	3.529871
F	11.144383	3.451238	4.207684
S	7.491702	-3.712209	3.626379
O	7.990177	-5.076103	3.493730
N	7.957434	-2.835062	2.367056
C	5.629038	-3.810471	3.483392
O	7.665352	-3.039521	4.945445
F	5.281663	-4.389279	2.338563
F	5.149848	-4.521053	4.507308
F	5.117944	-2.573432	3.531037
S	8.034296	-1.224437	2.373807
O	8.645467	-0.644177	3.604532
C	9.358730	-1.011363	1.073117
O	6.838904	-0.542927	1.869747
F	9.574689	0.297036	0.924848
F	10.492352	-1.600227	1.465745
F	8.962516	-1.532790	-0.082205
S	5.708362	0.490404	6.954084
O	6.525684	-0.642676	6.501346
F	4.214368	-0.128101	6.967438
O	5.921950	1.076196	8.260429
C	5.396527	3.619203	3.712518
C	5.503687	2.271457	3.359498
C	5.587996	1.295571	4.348691
C	5.563713	1.706207	5.683834
C	5.463416	3.047037	6.061155
C	5.377385	4.006170	5.054593
H	5.338768	4.375215	2.935451
H	5.542638	1.967538	2.319424
H	5.685961	0.252000	4.077540
H	5.477462	3.325856	7.107948
H	5.311605	5.055696	5.320831
C	12.724171	-3.288899	4.042628
C	12.398338	-4.320481	5.133844
C	10.933878	-4.010712	5.454480
O	10.749894	-2.601010	5.137732
C	11.957553	-2.065316	4.532566
H	13.794591	-3.094645	3.938353
H	12.334824	-3.616790	3.072957
H	13.030958	-4.154031	6.012394
H	12.535393	-5.353995	4.806216
H	10.243537	-4.590614	4.834877
H	10.676220	-4.160847	6.507358
H	12.516820	-1.508098	5.292859
H	11.653067	-1.384885	3.735348
C	7.365958	-3.733213	9.602401
C	8.801565	-3.532297	10.109439
C	9.292975	-2.391924	9.222714
O	8.622026	-2.583578	7.944735
C	7.522740	-3.530847	8.096536
H	6.953060	-4.715526	9.845288
H	6.702798	-2.970503	10.024611
H	9.398247	-4.435618	9.940746
H	8.855872	-3.281902	11.171822
H	9.005388	-1.415152	9.625770
H	10.368933	-2.391093	9.041613
H	7.799641	-4.457474	7.582650
H	6.641520	-3.109280	7.610167

Statistical Thermodynamic Analysis
Temperature 298.150 Kelvin. Pressure 1.00000 Atm.

SCF = -5677.05819745 l Predicted change in Energy=-9.251751D-09
Zero-point correction (ZPE) = -5676.60655545 0.451642
Internal Energy (U) = -5676.55037345 0.507824
Enthalpy (H) = -5676.54942945 0.508768
Gibbs Free Energy (G) = -5676.71314345 0.345054

Frequencies
5.8850 10.1632 12.3921
14.9160 16.3798 17.3488
22.1032 23.8243 29.9106
31.0094 32.9122 35.5854
37.4673 39.6673 42.3350
44.3488 48.3330 51.3316
52.6151 55.2099 58.9361
59.0004 63.7488 65.5185
72.3547 79.1860 82.2952
94.9423 99.5965 101.9761
108.5548 109.9014 114.3752
116.4904 121.3240 130.6094
150.6159 152.5047 172.4709
182.2009 186.8428 194.0113
201.1821 204.2934 209.0500
214.0757 223.7403 231.8753

244.4367 256.4390 263.4347
267.2615 269.8174 275.1176
277.3470 286.6998 287.4566
295.5685 298.0019 310.4396
313.1668 316.2219 324.0225
325.5926 327.0387 345.4650
347.0484 394.2826 398.3092
400.4215 409.2074 430.8653
435.5351 483.9893 494.3908
498.6141 512.2253 516.7178
522.1218 537.8678 538.3290
548.5728 550.3994 554.7370
561.4017 563.5742 571.4960
577.5250 579.3470 581.9361
588.5880 594.0971 616.8765
622.9996 637.4551 682.8174
687.1270 687.7347 719.9350
720.1743 721.3687 759.8334
761.1655 763.7826 780.5935
784.8913 802.9321 852.9180
854.4905 856.3896 879.0967
881.9376 889.2663 895.3318
919.7884 921.9545 932.1298
932.9879 947.2237 973.3360
978.2634 991.6453 1011.4472
1015.4233 1020.2347 1020.8379
1044.0107 1046.4901 1047.1309
1054.9291 1059.6514 1072.9739
1082.0827 1088.6786 1090.8740
1100.9817 1113.5939 1163.0616
1172.5071 1175.6249 1192.1785
1195.4550 1197.5522 1199.0292
1200.2092 1203.6460 1204.8964
1206.6768 1210.6585 1215.0023
1226.3730 1231.8458 1235.6652
1244.5104 1264.0995 1265.1795
1266.6309 1268.2476 1269.5369
1270.9495 1272.7061 1274.0079
1282.6947 1297.1938 1301.4996
1315.6469 1329.7444 1330.0249
1341.1061 1352.6323 1357.0448
1358.3463 1369.4497 1385.1241
1386.6992 1415.0468 1416.8757
1490.2334 1501.1401 1501.4674
1510.2394 1511.2930 1514.4440
1531.7518 1538.7869 1544.4461
1551.3653 1636.3850 1641.3128
3058.5154 3061.4270 3062.1404
3062.6753 3067.1640 3067.9554
3070.2645 3070.3040 3111.4041
3122.7110 3124.8772 3127.1693
3131.6190 3140.2493 3142.1390
3143.2607 3197.7425 3214.3895
3224.8442 3235.9866 3246.3379

$Ca(NTf_2)_2(PhSO_2F)(THF)_3$ 5c

Gaussian 16: ES64L-G16RevB.01 20-Dec-2017

B3LYP/6-31G(d,p) gfprint gfinput scf=(direct,tight,maxcycle=300,xqc) opt=(maxcycle=250) freq=noraman
MAN.EDU\28-Nov-2020\0\\# B3LYP/6-31G(d,p) gfprint gfinput scf=(direct, tight,maxcycle=300,xqc) opt=(maxcycle=250) freq=noraman\\010_ca2plus_2

Full point group C1 NOp 1
Stoichiometry C22H29CaF13N2O13S5
Framework group C1[X(C22H29CaF13N2O13S5)]

Num atoms: 85
Charge = 0 Multiplicity = 1

SCF = -5909.53379914 l Predicted change in Energy=-1.192073D-08

Optimization completed.
Maximum Force 0.000014 0.000450 YES
RMS Force 0.000002 0.000300 YES
Maximum Displacement 0.001317 0.001800 YES
RMS Displacement 0.000176 0.001200 YES

Atom Coordinates (in Angstroms)
Type X Y Z

Ca 10.542568 -2.082640 5.907451
S 13.021751 0.712801 5.620552
O 12.502214 -0.643102 5.287375
N 12.773831 1.178037 7.129891
C 14.872636 0.471084 5.506576

O 12.724805 1.804988 4.693977
S 12.375915 0.165453 8.324852
O 13.489070 -0.220763 9.192757
C 11.311357 1.330221 9.325638
O 11.438988 -0.920662 7.927251
F 10.271901 1.754883 8.601766
F 10.850215 0.650261 10.383698
F 12.019411 2.372951 9.744890
F 15.258320 -0.458561 6.391638
F 15.185296 0.052470 4.275482
F 15.507819 1.610657 5.760821
S 9.042709 -5.114936 4.412768
O 10.014545 -5.916008 3.670587
N 8.060001 -4.321165 3.416824
C 7.903697 -6.360817 5.217744
O 9.542937 -4.332316 5.575905
F 8.611559 -7.074230 6.101170
F 6.916671 -5.729669 5.867990
F 7.381597 -7.175009 4.306770
S 7.633992 -2.776947 3.564567
O 7.206209 -2.270619 2.265330
C 6.085161 -2.761972 4.623802
O 8.585969 -1.939436 4.342380
F 5.520824 -1.554682 4.547428
F 5.222873 -3.680777 4.190896
F 6.395351 -3.018642 5.901113
S 8.516457 1.104963 5.226824
O 7.161442 0.759665 5.605333
F 8.799211 2.583654 5.807399
O 9.653547 0.323757 5.719658
C 8.861594 1.856283 0.781234
C 7.660650 1.387420 1.315866
C 7.544750 1.165997 2.687250
C 8.657745 1.417394 3.490855
C 9.876836 1.874833 2.980161
C 9.962986 2.098853 1.607802
H 8.943197 2.028365 -0.287706
H 6.814278 1.183737 0.668851
H 6.628096 0.786750 3.121889
H 10.732789 2.043805 3.626302
H 10.895410 2.459589 1.185913
C 11.526247 -2.861796 1.280599
C 12.377171 -4.014820 1.881493
C 12.483112 -3.661782 3.376005
O 11.398233 -2.747114 3.628888
C 11.324320 -1.903037 2.460835
H 12.018646 -2.365262 0.440165
H 10.562312 -3.239736 0.931599
H 13.366990 -4.086305 1.422517
H 11.870628 -4.973251 1.754647
H 12.349245 -4.516618 4.037560
H 13.432025 -3.158677 3.609243
H 12.120065 -1.148664 2.517687
H 10.354483 -1.405657 2.475205
C 7.878579 -1.623253 9.771322
C 7.599683 -3.122798 9.595175
C 8.600292 -3.518942 8.505849
O 8.839070 -2.319025 7.726186
C 8.160224 -1.189528 8.337162
H 7.041730 -1.077119 10.214488
H 8.764673 -1.460771 10.394230
H 6.573799 -3.278935 9.244616
H 7.736488 -3.702578 10.511718
H 9.556572 -3.840961 8.937134
H 8.231884 -4.298263 7.836811
H 8.817644 -0.324253 8.261962
H 7.238855 -0.991421 7.778560
C 14.350292 -4.608426 7.434290
C 13.250376 -5.561564 7.921645
C 12.064070 -5.152182 7.050572
O 12.238305 -3.731601 6.792250
C 13.569413 -3.315314 7.217320
H 15.165403 -4.481452 8.151057
H 14.777117 -4.967839 6.491160
H 13.032260 -5.379788 8.979865
H 13.503649 -6.618296 7.802853
H 12.060060 -5.683941 6.092479
H 11.092218 -5.301422 7.524115
H 13.476467 -2.736584 8.140828
H 13.984216 -2.671048 6.440992

Statistical Thermodynamic Analysis
Temperature 298.150 Kelvin. Pressure 1.00000 Atm.

SCF = -5909.53379914 I Predicted change in Energy=-1.192073D-08
Zero-point correction (ZPE) = -5908.96317014 0.570629
Internal Energy (U) = -5908.90046314 0.633336
Enthalpy (H) = -5908.89951914 0.63428
Gibbs Free Energy (G) = -5909.07597514 0.457824

Frequencies
9.2213 13.6397 14.3358
19.8867 22.2728 24.7939

28.8429 31.6817 31.8105
32.0598 34.8836 36.6624
37.6822 38.8771 42.0810
45.3385 45.9444 46.8721
50.8169 52.0638 52.7596
55.2363 58.1918 60.8772
61.4557 66.1602 69.7853
72.9174 74.0918 82.6879
84.7296 89.4984 94.7361
97.4471 102.1158 104.6638
110.7366 112.1781 118.3928
126.1190 126.9478 127.4382
133.0552 150.7628 161.4050
176.7347 180.9528 182.8158
186.6473 200.9878 202.9785
207.2789 215.4757 216.9996
227.9072 230.8935 242.5384
264.2837 269.1857 274.2470
275.7595 278.4881 280.6586
283.3939 294.1518 296.0901
299.3110 309.4124 313.4983
317.8038 325.1022 326.5188
335.7555 339.4238 344.2860
390.5071 398.1544 408.1860
409.2524 423.1172 430.2539
486.3927 492.2965 495.5112
512.5481 518.5033 519.2635
538.3216 539.1259 548.4777
549.9289 554.6284 559.7832
561.3811 565.9167 573.3530
579.1227 579.7132 582.1823
589.8268 623.6482 629.1474
638.5105 641.7074 667.0994
678.3604 684.1103 690.7302
718.3548 719.4070 721.2265
759.7169 761.4739 763.2484
779.7861 783.3990 805.6095
812.3066 851.0627 854.2745
864.1574 870.4041 879.4900
881.7930 890.6403 899.7164
911.4622 919.9746 921.1425
929.1691 933.7231 935.2906
952.1928 953.3341 970.9268
973.5716 980.5344 994.1111
1010.5459 1011.8280 1017.0412
1017.3062 1046.7746 1046.8033
1047.9372 1059.1185 1064.3750
1065.9106 1070.9045 1077.2799
1088.5806 1093.2354 1095.7404
1103.5439 1118.1185 1154.3826
1168.2308 1172.2069 1174.4729
1193.6174 1194.1384 1195.2611
1199.6447 1202.1207 1204.3048
1205.6258 1214.5809 1215.1022
1216.9972 1225.3748 1226.7217
1231.6525 1234.6382 1235.8263
1239.0400 1255.2317 1260.6826
1261.9689 1262.5981 1265.3330
1267.7879 1269.1583 1272.4513
1276.0480 1280.4710 1285.5994
1287.0454 1292.2696 1307.0075
1324.2091 1329.2810 1330.0448
1330.7637 1348.6198 1353.6888
1356.6819 1357.1170 1371.3545
1371.9546 1381.6470 1384.4525
1404.2940 1414.3236 1414.9158
1490.2589 1499.9944 1501.2725
1501.5176 1510.7330 1511.4242
1514.1645 1520.2835 1522.4161
1533.5334 1536.4818 1538.0369
1546.5171 1552.0819 1638.4364
1641.0377 3020.0835 3036.9056
3037.5604 3057.1366 3058.8513
3064.4392 3064.5435 3064.8944
3070.6174 3078.1866 3083.8714
3086.7120 3118.9567 3119.8304
3122.5430 3125.1487 3126.9185
3134.0589 3138.6315 3141.2207
3150.8399 3155.3134 3161.7473
3166.4708 3194.3176 3205.7021
3213.2902 3218.5625 3241.2889

$Ca(NTf_2)_2$(DABCO)(THF) 6a

Gaussian 16: ES64L-G16RevB.01 20-Dec-2017

B3LYP/6-31G(d,p) gfprint gfinput scf=(direct,tight,maxcycle=300,xqc) opt=(maxcycle=250) freq=noraman
N.EDU\26-Mar-2021\0\\# B3LYP/6-31G(d,p) gfprint gfinput scf=(direct,ti ght,maxcycle=300,xqc) opt=(maxcycle=250) freq=noraman\\010b_ca_2ntf2_d
----
Full point group C1 NOp 1

Stoichiometry C14H20CaF12N4O9S4
Framework group
C1[X(C14H20CaF12N4O9S4)]

Num atoms: 64
Charge = 0 Multiplicity = 1

SCF = -4909.85155431 l Predicted change in Energy=-3.309803D-08

Optimization completed.
Maximum Force 0.000031 0.000450 YES
RMS Force 0.000004 0.000300 YES
Maximum Displacement 0.001795 0.001800 YES
RMS Displacement 0.000425 0.001200 YES

Atom Coordinates (in Angstroms)

Type	X	Y	Z
C	-2.220737	-4.897192	2.328213
F	-2.787793	-5.793722	3.127144
F	-1.368150	-4.145998	3.025375
F	-1.557881	-5.524804	1.349386
S	-3.531934	-3.790207	1.578536
O	-2.724674	-2.873990	0.713633
O	-4.257547	-3.192060	2.690704
N	-4.497123	-4.769625	0.751799
S	-4.284413	-5.142963	-0.804132
O	-3.375876	-6.255754	-1.084030
O	-4.130687	-3.941371	-1.688418
C	-6.022773	-5.727200	-1.167790
F	-6.888984	-4.733279	-0.972924
F	-6.057998	-6.101011	-2.447232
F	-6.332347	-6.757187	-0.390159
C	-0.773768	2.431191	-1.531468
F	-2.073939	2.690694	-1.346849
F	-0.156729	2.435997	-0.349551
F	-0.241032	3.354451	-2.320667
S	-0.568578	0.729681	-2.276269
O	-1.335141	-0.141520	-1.327377
O	0.864472	0.464626	-2.370985
N	-1.313360	0.994325	-3.680296
S	-2.134735	-0.089293	-4.529025
O	-2.958841	0.580780	-5.521818
O	-2.787508	-1.148319	-3.698402
C	-0.842543	-1.067919	-5.465503
F	-0.006062	-1.641067	-4.585487
F	-1.444237	-2.022015	-6.176731
F	-0.155218	-0.270626	-6.273656
Ca	-2.811959	-1.976374	-1.487320
N	-6.765578	1.070007	-0.055293
C	-6.289165	1.464826	-1.388961
C	-5.117377	0.541786	-1.847273
C	-6.058329	-1.269691	-0.537386
C	-7.224064	-0.325828	-0.106508
C	-5.654527	1.174873	0.902621
C	-4.467603	0.258640	0.468804
H	-7.130923	1.407558	-2.086715
H	-5.970179	2.511291	-1.345667
H	-5.357712	0.005570	-2.770398
H	-4.203459	1.114939	-2.026925
H	-6.296331	-1.820294	-1.452850
H	-5.835269	-2.005389	0.240474
H	-8.061100	-0.386881	-0.809803
H	-7.604358	-0.601253	0.882398
H	-5.343242	2.223332	0.955351
H	-6.026132	0.891627	1.892587
H	-3.561195	0.838517	0.268699
H	-4.231201	-0.486443	1.234283
N	-4.821427	-0.470275	-0.788370
C	-0.773560	-4.481674	-2.842686
O	-0.871230	-3.331142	-1.970213
C	0.423890	-3.202325	-1.335811
C	0.870589	-4.644950	-1.049820
C	0.049302	-5.506767	-2.050467
H	-1.788655	-4.811237	-3.068226
H	-0.272573	-4.173579	-3.768686
H	1.102122	-2.685151	-2.024902
H	0.295423	-2.585061	-0.444907
H	1.949179	-4.755786	-1.186060
H	0.631772	-4.925013	-0.021757
H	-0.617564	-6.186973	-1.517025
H	0.684272	-6.101398	-2.711914

Statistical Thermodynamic Analysis
Temperature 298.150 Kelvin. Pressure 1.00000 Atm.

SCF = -4909.85155431 l Predicted change in Energy=-3.309803D-08
Zero-point correction (ZPE) = -4909.43717931 0.414375

Internal Energy (U) = -4909.38966431
0.46189
Enthalpy (H) = -4909.38872031   0.462834
Gibbs Free Energy (G) = -4909.53058231
0.320972

Frequencies
10.2282 12.3468 13.9725
20.5973 23.8688 26.4589
29.6565 30.8798 33.6886
35.3892 35.6850 38.5538
39.2887 42.6332 48.4807
53.9511 58.1402 60.0712
64.4089 69.8421 76.5408
79.3060 83.2199 89.5783
93.0531 97.3473 103.0279
109.8059 116.7597 119.2720
160.8937 166.4004 176.6670
181.4266 190.3290 205.6658
210.5216 230.7282 234.5316
241.2265 261.8217 273.4203
283.4209 285.1131 285.8532
293.4325 295.1764 305.4261
314.8379 316.4108 322.7841
328.6984 330.0310 337.7765
351.0895 360.7877 395.8529
406.4469 424.1517 425.7168
427.2517 440.5490 494.0154
500.8272 506.3478 516.1432
537.0203 537.6975 550.4687
550.6542 554.2809 556.5959
562.9884 566.1087 585.7445
587.0968 589.7668 590.7545
619.0991 628.4242 638.7313
647.6396 657.4778 715.4899
717.5996 763.6364 765.0488
780.2087 783.3437 786.9598
803.8336 812.8718 817.5659
839.4449 843.0111 865.2544
906.0309 907.8158 909.0799
922.9066 951.2317 968.8419
977.9410 1004.3413 1012.0674
1018.1080 1022.5011 1026.1426
1031.0622 1053.5255 1058.5003
1063.3734 1070.3852 1076.8279
1077.7538 1086.9627 1095.0043
1153.1636 1200.4767 1201.6295
1204.8524 1206.9019 1208.0981
1209.0500 1211.7172 1229.7733
1233.6135 1238.7893 1240.2931
1241.5722 1254.7351 1268.4826
1269.2633 1271.3319 1272.3818
1274.8372 1277.1440 1284.5836
1286.6161 1290.0901 1302.3372
1313.3667 1328.8008 1334.3196
1336.9961 1339.7978 1347.0621
1348.0621 1356.8523 1358.5999
1363.0135 1364.1131 1378.7433
1383.0835 1407.4695 1410.5561
1494.4162 1498.4823 1503.1847
1505.2496 1508.6550 1513.6144
1520.1646 1520.7716 1525.3007
1540.1031 3048.0149 3052.7316
3052.9425 3055.5632 3056.3468
3064.7873 3065.7566 3070.1055
3085.2005 3093.1600 3095.0030
3097.8142 3098.3577 3119.2328
3124.2923 3125.1813 3125.4805
3136.9352 3144.5494 3152.3718

$Ca(NTf_2)_2(DABCO)(THF)_2$ 6b

Gaussian 16: ES64L-G16RevB.01 20-Dec-2017

B3LYP/6-31G(d,p) gfprint gfinput scf=(direct,tight,maxcycle=300,xqc) opt=(maxcycle=250) freq=noraman
AN.EDU\02-Apr-2021\0\\# B3LYP/6-31G(d,p) gfprint gfinput scf=(direct,t
ight,maxcycle=300,xqc) opt=(maxcycle=250) freq=noraman\\010b_ca_2ntf2_

Full point group C1 NOp 1
Stoichiometry C18H28CaF12N4O10S4
Framework group
C1[X(C18H28CaF12N4O10S4)]

Num atoms: 77
Charge = 0 Multiplicity = 1

SCF = -5142.33004237 l Predicted change in Energy=-2.779214D-08

Optimization completed.
Maximum Force   0.000024
0.000450   YES

RMS Force 0.000003 0.000300
YES
Maximum Displacement 0.000886
0.001800 YES
RMS Displacement 0.000156
0.001200 YES

Atom Coordinates (in Angstroms)
Type X Y Z

C -3.496197 2.810332 0.614646
F -3.513185 1.965379 1.658374
F -4.745902 3.120738 0.283636
F -2.836011 3.913537 0.963129
S -2.614159 2.008141 -0.827955
O -1.344656 1.537234 -0.216492
O -2.534036 3.032977 -1.861141
N -3.611347 0.832597 -1.308441
S -3.433519 -0.733182 -1.026322
O -2.231715 -1.107532 -0.222798
O -3.682503 -1.516185 -2.234670
C -4.831806 -1.155849 0.139935
F -4.762591 -2.461482 0.423882
F -4.706141 -0.458507 1.275660
F -6.005601 -0.883780 -0.418793
C 3.327126 3.246401 -0.820809
F 2.466757 4.252811 -0.662896
F 4.570921 3.693388 -0.674080
F 3.179309 2.727050 -2.044514
S 2.962128 1.930694 0.457882
O 3.179605 2.552135 1.759180
O 1.572977 1.513334 0.111565
N 4.060893 0.787113 0.186348
S 3.702596 -0.526272 -0.677894
O 3.943542 -0.437351 -2.119146
O 2.419142 -1.173552 -0.262959
C 4.995930 -1.675317 0.025249
F 4.850315 -1.792364 1.344351
F 4.811655 -2.875838 -0.541216
F 6.216385 -1.235493 -0.257394
Ca 0.105204 -0.437085 -0.101778
N -0.057651 -0.410371 2.525281
C -0.137964 1.000422 3.018269
C -0.157988 1.016814 4.577754
C 1.079354 -1.031354 4.665674
C 1.161160 -1.045645 3.106920
C -1.256053 -1.144737 3.024814
C -1.312123 -1.079165 4.583434
H 0.718344 1.548185 2.619382
H -1.042567 1.443649 2.594127
H 0.714614 1.544987 4.975042
H -1.050496 1.526260 4.956176
H 1.938507 -0.507748 5.096954
H 1.077064 -2.049111 5.070763
H 2.033491 -0.492833 2.746586
H 1.229455 -2.065037 2.711487
H -2.141669 -0.699636 2.563249
H -1.186525 -2.178838 2.672334
H -2.217752 -0.565482 4.922677
H -1.322459 -2.083850 5.020027
N -0.147659 -0.355185 5.111836
C 1.070770 -3.739136 -1.033469
O 0.126856 -2.990321 -0.206714
C -0.888135 -3.896429 0.292261
C -0.934198 -5.029603 -0.724718
C 0.552887 -5.183075 -1.077001
H 1.079866 -3.274626 -2.024288
H 2.068341 -3.644215 -0.602977
H -0.584108 -4.268715 1.281466
H -1.816949 -3.334468 0.383717
H -1.378989 -5.941676 -0.318299
H -1.516540 -4.723491 -1.600389
H 0.720935 -5.648637 -2.051379
H 1.058641 -5.795188 -0.322670
C -0.432295 -1.079933 -3.565232
O 0.002082 -0.177988 -2.523714
C 0.462089 1.020216 -3.204006
C -0.522072 1.218450 -4.367828
C -1.151809 -0.186495 -4.584520
H 0.451012 -1.567078 -4.001291
H -1.077786 -1.830006 -3.105293
H 0.457464 1.829400 -2.473679
H 1.488892 0.848971 -3.548677
H -1.289396 1.946686 -4.097452
H -0.004684 1.585121 -5.258038
H -1.014523 -0.557590 -5.603437
H -2.222925 -0.167930 -4.372600

Statistical Thermodynamic Analysis
Temperature 298.150 Kelvin. Pressure
1.00000 Atm.

SCF = -5142.33004237 l Predicted change in
Energy=-2.779214D-08
Zero-point correction (ZPE) = -5141.79694537
0.533097

Internal Energy (U) = -5141.74278037
0.587262
Enthalpy (H) = -5141.74183637 0.588206
Gibbs Free Energy (G) = -5141.90211237
0.42793

Frequencies
0.7944 7.3564 13.7019
18.5241 22.4587 23.8316
26.0179 30.6178 31.9679
35.0411 36.2468 37.7255
39.4765 41.5762 45.4471
48.9250 49.8220 53.1282
55.8873 57.4841 63.5432
66.0379 68.9709 74.8325
77.8000 79.4887 82.5179
85.5212 87.4563 91.2285
103.5916 106.4142 114.6596
117.2913 123.4816 128.0472
144.8285 171.3034 177.2513
182.1477 188.3066 197.4829
207.7294 220.4873 226.8051
237.1387 239.3938 253.1256
276.0743 277.2882 280.0013
285.3419 287.1035 288.6785
294.1043 297.6303 315.7938
317.3900 322.3980 326.9882
331.2629 334.3510 334.6437
352.5830 396.1721 400.6017
409.2525 424.0230 427.3158
438.1106 490.5003 496.0346
505.3677 520.4344 539.0257
540.4256 548.6005 550.3524
553.8778 560.7081 562.8120
562.9502 578.8968 584.0115
586.3645 587.4698 591.0456
618.0957 632.9066 647.9536
649.0955 661.9679 681.0336
717.8212 725.4048 761.1082
763.9740 779.1428 781.2715
787.0153 804.2302 815.2839
815.6122 836.9911 841.4423
842.5405 869.3104 882.8880
890.8484 906.2011 908.5663
908.7255 918.8005 923.1077
934.5319 953.2364 972.1940
978.7412 979.9108 1003.7885
1008.9251 1015.2996 1022.4978
1029.1483 1031.9361 1048.7443
1053.2006 1058.7406 1064.4255
1065.1795 1075.6096 1077.7880
1084.3962 1091.3170 1104.9316
1156.0736 1171.6910 1194.6378
1200.9520 1202.4704 1206.3544
1207.7120 1209.4636 1210.7867
1213.3678 1214.6787 1226.9558
1229.6193 1238.8022 1242.0103
1243.0116 1245.5157 1260.4473
1262.9640 1264.2685 1266.0115
1269.6727 1270.3332 1274.4017
1278.2847 1284.3441 1287.0098
1287.8203 1300.4916 1312.1874
1328.5162 1330.5797 1333.5741
1334.5890 1338.8546 1345.3206
1347.6665 1355.2035 1355.6293
1357.4516 1362.4068 1363.6759
1378.2350 1380.5472 1382.3124
1406.5076 1409.4623 1412.7502
1495.1112 1498.6837 1501.6381
1505.3693 1505.6012 1508.3896
1512.9175 1513.2602 1519.5179
1520.8453 1525.3085 1532.8654
1540.3816 1547.4221 3009.9700
3021.5869 3047.2715 3049.3273
3054.0366 3054.0985 3057.4706
3064.9096 3066.3601 3068.9831
3073.9796 3082.9387 3088.6224
3090.8296 3091.5355 3093.7335
3100.1441 3116.0760 3120.0508
3123.5001 3126.4459 3127.9476
3137.4150 3145.7404 3145.7672
3146.2979 3158.7140 3161.1803

$Ca(NTf_2)_2(DABCO)(THF)_3$ 6c

Gaussian 16: ES64L-G16RevB.01 20-Dec-2017

B3LYP/6-31G(d,p) gfprint gfinput scf=(direct,tight,maxcycle=300,xqc) opt=(maxcycle=250) freq=noraman AN.EDU\23-Mar-2021\0\\# B3LYP/6-31G(d,p) gfprint gfinput scf=(direct,t ight,maxcycle=300,xqc) opt=(maxcycle=250) freq=noraman\\010b_ca_2ntf2_

Full point group C1 NOp 1

Stoichiometry C22H36CaF12N4O11S4
Framework group
C1[X(C22H36CaF12N4O11S4)]

Num atoms: 90
Charge = 0 Multiplicity = 1

SCF = -5374.80097403 l Predicted change in Energy=-4.636954D-08

Optimization completed.
Maximum Force 0.000033 0.000450 YES
RMS Force 0.000004 0.000300 YES
Maximum Displacement 0.001642 0.001800 YES
RMS Displacement 0.000322 0.001200 YES

Atom Type	Coordinates (in Angstroms) X	Y	Z
Ca	-4.117046	-0.742937	1.613245
S	-7.040302	0.481866	3.560007
O	-7.389822	1.864374	3.246853
O	-5.609577	0.066829	3.460947
N	-8.057686	-0.538046	2.853562
S	-7.575295	-2.030719	2.444194
O	-7.692013	-3.047579	3.491094
O	-6.319954	-2.043144	1.638145
C	-8.924551	-2.415540	1.207637
F	-10.103149	-2.486937	1.815360
F	-8.974876	-1.495640	0.240918
F	-8.630854	-3.600120	0.658410
C	-7.356319	0.296964	5.395178
F	-8.588844	0.691141	5.698533
F	-7.192605	-0.978628	5.760825
F	-6.470556	1.054035	6.057842
S	-1.961555	0.280392	-1.360740
O	-1.136432	1.485121	-1.230313
O	-3.236349	0.265626	-0.592096
N	-1.154093	-1.121503	-1.318839
S	-0.900278	-1.885053	0.068729
O	-0.811986	-3.327649	-0.142169
O	-1.734392	-1.384588	1.199546
C	0.818004	-1.363783	0.595004
F	0.872447	-0.033507	0.722420
F	1.092102	-1.923943	1.780525
F	1.717668	-1.760633	-0.299427
C	-2.503520	0.222765	-3.147702
F	-3.216562	1.331517	-3.385576
F	-1.453377	0.188949	-3.958808
F	-3.281170	-0.840967	-3.376573
C	-4.471378	-3.797479	-2.099603
C	-5.028161	-2.673137	-1.221969
C	-3.983045	-4.064674	0.276016
C	-3.894800	-4.807540	-1.071847
H	-5.243733	-4.234237	-2.738265
H	-3.677674	-3.408640	-2.741219
H	-6.061853	-2.873434	-0.908829
H	-4.964674	-1.683270	-1.674046
H	-3.065084	-4.131053	0.858980
H	-4.837178	-4.404975	0.875829
H	-4.466484	-5.738909	-1.042010
H	-2.857334	-5.050946	-1.307729
O	-4.178228	-2.672609	-0.058182
C	-6.420405	1.559174	-1.614175
C	-5.718555	2.780762	-0.972760
C	-5.396598	2.320540	0.467044
O	-5.724847	0.911482	0.529973
C	-6.798659	0.702400	-0.406989
H	-7.292020	1.834776	-2.213890
H	-5.720125	1.019263	-2.256560
H	-6.371314	3.657843	-0.957423
H	-4.813256	3.046484	-1.522711
H	-4.341327	2.411567	0.724996
H	-6.005890	2.846199	1.210275
H	-7.742053	1.029215	0.048901
H	-6.860106	-0.366293	-0.608080
C	-2.188264	2.950646	4.171511
C	-1.274673	3.001947	2.940358
C	-1.522362	1.639906	2.294613
O	-2.895869	1.280136	2.637934
C	-3.410453	2.217208	3.627687
H	-1.718205	2.371927	4.974859
H	-2.442147	3.937091	4.568305
H	-0.220328	3.155645	3.183868
H	-1.586278	3.807520	2.266509
H	-1.428090	1.655322	1.209319
H	-0.857430	0.870064	2.698937
H	-3.965747	1.652586	4.374928
H	-4.103986	2.905125	3.130378
N	-3.307328	-2.219804	3.929318
C	-2.982750	-1.227030	4.992181
C	-2.594522	-1.955836	6.317527
C	-1.742514	-3.808378	5.073832

C -2.115290 -3.094133 3.737640
C -4.432674 -3.065942 4.420751
C -4.034570 -3.781183 5.749468
H -2.167353 -0.597814 4.621327
H -3.861717 -0.592854 5.128360
H -1.576210 -1.697163 6.628577
H -3.267959 -1.671963 7.133611
H -0.723732 -3.552848 5.385564
H -1.786560 -4.897627 4.966605
H -1.298707 -2.473809 3.364678
H -2.353446 -3.818075 2.953255
H -5.300574 -2.422990 4.564619
H -4.685885 -3.783604 3.635292
H -4.715480 -3.504955 6.561523
H -4.079210 -4.870307 5.643131
N -2.665965 -3.414582 6.148176

Statistical Thermodynamic Analysis
Temperature 298.150 Kelvin. Pressure 1.00000 Atm.

SCF = -5374.80097403 l Predicted change in Energy=-4.636954D-08
Zero-point correction (ZPE) = -5374.14855803 0.652416
Internal Energy (U) = -5374.08811203 0.712862
Enthalpy (H) = -5374.08716703 0.713807
Gibbs Free Energy (G) = -5374.25887803 0.542096

Frequencies
0.6885 16.6197 23.3515
24.3104 24.8642 28.9613
30.4616 31.8693 34.2946
37.6440 38.9311 40.6121
42.6517 43.0536 47.1920
50.1829 51.4314 54.5666
58.4191 60.9859 63.8032
65.3625 66.1988 68.9772
71.2264 72.6721 75.4873
78.6872 80.6610 85.7698
86.7826 90.9969 95.6447
98.1587 100.1889 104.4121
107.0212 110.9065 113.9117
121.8784 127.9109 133.9291
145.2331 159.3927 174.4400
178.0480 182.8416 185.1306
188.9395 199.2941 206.1226
218.5567 222.7544 232.2187
235.6398 264.5828 277.9427
278.2068 284.3921 284.8488
295.0132 295.3157 298.7232
310.0009 319.0967 319.7706
327.9901 329.9595 333.2121
336.9677 341.6498 343.7551
396.7257 398.4386 426.2879
427.8019 437.2783 441.9873
487.3698 488.7945 505.8436
506.8850 538.4485 538.5836
549.6285 549.8793 552.8400
553.3175 559.4408 560.9708
579.2694 587.8232 588.2285
589.5176 591.2108 608.5786
612.5754 636.3750 647.2610
648.9550 675.6591 677.6188
688.6416 723.7868 724.3633
761.6939 761.8219 773.1456
783.9150 784.2012 806.3290
807.0755 811.0988 818.2176
840.5790 842.3516 850.0299
874.5733 876.2076 877.6422
884.6797 906.4232 911.5216
913.4567 915.3134 918.8463
923.1966 926.8762 936.0853
946.6612 954.4466 972.3836
975.3230 979.3166 980.6075
1001.3473 1002.4570 1006.0191
1028.0719 1029.3650 1031.7108
1048.7972 1051.4547 1056.2007
1059.0541 1061.3790 1066.2055
1073.6618 1073.7450 1078.2026
1079.4319 1090.0912 1098.4350
1155.9947 1157.8245 1171.6807
1195.5403 1199.4002 1200.2099
1201.2203 1204.8450 1207.4772
1209.2614 1211.2688 1213.3514
1222.6862 1226.4293 1228.1184
1230.5664 1232.0120 1238.6198
1240.5625 1242.4482 1257.8904
1259.1985 1260.2335 1262.3995
1263.8889 1265.8432 1269.7341
1272.4817 1273.0809 1281.6682
1284.7198 1286.8809 1291.1211
1294.4481 1298.7701 1322.4190
1323.9192 1330.7222 1331.5970
1333.7539 1336.2671 1336.9014
1342.5855 1344.8186 1352.7791

1354.6230 1356.9508 1359.2728
1361.6203 1372.6200 1377.8581
1380.0489 1383.2227 1402.4083
1405.4845 1410.7177 1413.7066
1494.2389 1497.5474 1499.6329
1499.9631 1501.8279 1505.0972
1506.9685 1510.9688 1511.9450
1515.3431 1517.1037 1519.7398
1521.8296 1525.5431 1535.9607
1539.4272 1543.7685 1547.2809
3031.8123 3037.3562 3042.9273
3045.2946 3045.7197 3050.9867
3054.2754 3059.6378 3063.1977
3064.3241 3065.4950 3069.6529
3077.5028 3078.2102 3080.8725
3082.2851 3084.6923 3087.1066
3088.8836 3090.9175 3097.0023
3119.5191 3119.7091 3126.1619
3126.3518 3127.8045 3134.9815
3141.4758 3142.7303 3151.9910
3154.2405 3159.8458 3160.4884
3162.8321 3163.9337 3165.1191

Ca(NTf₂)₂(Pip)(THF) 7a

Gaussian 16: ES64L-G16RevB.01 20-Dec-2017

B3LYP/6-31G(d,p) gfprint gfinput scf=(direct,tight,maxcycle=300,xqc) opt=(maxcycle=250) freq=noraman
N.EDU\31-Mar-2021\0\\# B3LYP/6-31G(d,p) gfprint gfinput scf=(direct,ti ght,maxcycle=300,xqc) opt=(maxcycle=250) freq=noraman\\010c_ca_2ntf2_p

----

Full point group C1 NOp 1
Stoichiometry C13H19CaF12N3O9S4
Framework group C1[X(C13H19CaF12N3O9S4)]

Num atoms: 61
Charge = 0 Multiplicity = 1

SCF = -4816.43036646 l Predicted change in Energy=-1.530634D-08

Optimization completed.

Maximum Force 0.000029 0.000450 YES
RMS Force 0.000003 0.000300 YES
Maximum Displacement 0.001558 0.001800 YES
RMS Displacement 0.000312 0.001200 YES

Atom Type	Coordinates (in Angstroms) X	Y	Z
C	-7.374628	1.316358	-0.977757
F	-7.846529	1.825670	-2.117025
F	-6.431285	2.126827	-0.491681
F	-8.361242	1.196697	-0.097884
S	-6.640587	-0.358485	-1.362384
O	-5.517939	-0.027570	-2.298811
O	-7.710610	-1.184370	-1.914082
N	-6.168107	-0.763621	0.124180
S	-4.868010	-1.615957	0.505202
O	-4.560081	-1.452808	1.916458
O	-3.734161	-1.474315	-0.462027
C	-5.362793	-3.403444	0.257557
F	-6.388042	-3.715054	1.040364
F	-5.710980	-3.581942	-1.027575
F	-4.324362	-4.191537	0.538801
C	1.377550	-1.458101	-3.970052
F	1.402457	-0.186033	-3.561595
F	1.783792	-2.249625	-2.977731
F	2.177405	-1.606381	-5.020565
S	-0.374916	-1.927137	-4.434536
O	-1.137181	-1.606111	-3.185475
O	-0.326677	-3.321885	-4.855898
N	-0.753425	-0.971973	-5.670313
S	-1.586448	0.394696	-5.460161
O	-0.841925	1.538394	-4.917746
O	-2.935565	0.169884	-4.848728
C	-1.958580	0.749040	-7.257440
F	-2.672284	1.874272	-7.298961
F	-0.827893	0.908766	-7.934022
F	-2.667902	-0.247598	-7.782031
Ca	-3.299666	-0.737984	-2.654184
C	-5.120192	-2.614124	-4.863176
O	-4.157198	-2.690142	-3.785298
C	-3.666887	-4.055926	-3.760737
C	-3.723439	-4.553474	-5.221668
C	-4.480358	-3.433679	-5.982047

H -6.072422 -3.040069 -4.522247
H -5.268271 -1.559380 -5.102451
H -2.655949 -4.028537 -3.351348
H -4.312670 -4.642283 -3.097771
H -2.719374 -4.695494 -5.625306
H -4.251972 -5.508500 -5.280531
H -5.223567 -3.823991 -6.681847
H -3.780190 -2.808537 -6.543103
C -0.974012 1.121303 -0.938445
C -0.242594 2.387663 -0.476682
C -1.214541 3.386117 0.168340
C -2.392650 3.677384 -0.772500
C -3.070998 2.377510 -1.220529
N -2.129582 1.402840 -1.834216
H -1.361716 0.572455 -0.070531
H -0.293220 0.453395 -1.473680
H 0.245574 2.853909 -1.343721
H 0.554621 2.111589 0.223278
H -0.693868 4.313958 0.429308
H -1.597270 2.965273 1.108472
H -3.131510 4.323801 -0.284795
H -2.033509 4.219443 -1.658497
H -3.534758 1.883767 -0.357801
H -3.870515 2.580167 -1.940186
H -1.753453 1.822884 -2.688581

Statistical Thermodynamic Analysis
Temperature 298.150 Kelvin. Pressure 1.00000 Atm.

SCF = -4816.43036646 l Predicted change in Energy=-1.530634D-08
Zero-point correction (ZPE) = -4816.04018646 0.39018
Internal Energy (U) = -4815.99354246 0.436824
Enthalpy (H) = -4815.99259746 0.437769
Gibbs Free Energy (G) = -4816.13301746 0.297349

Frequencies
10.8275 12.0357 14.4735
19.5865 21.4938 23.4366
26.2005 27.0283 31.5091
33.9863 35.7297 40.8084
41.3219 44.6221 47.3321
49.1257 52.5220 60.9834
63.2060 72.0275 77.1762
80.8849 88.3899 93.6251
97.4927 105.2325 109.2601
120.7669 127.6897 159.8320
165.3405 175.2214 182.1298
190.8404 204.6893 214.4196
228.8882 234.6359 239.5955
243.9899 260.8225 273.2314
281.5996 283.3552 285.8938
286.6167 293.0275 298.4647
303.8929 314.5494 318.9076
322.4760 333.1965 351.5639
362.5654 396.7782 405.6111
411.1771 423.5543 450.5255
458.3645 459.5114 489.7186
500.4166 502.4740 517.4513
537.3281 538.1973 550.5128
551.2255 553.8484 557.6922
562.3410 566.2426 586.3655
589.2182 591.1639 615.5923
627.4378 648.6088 692.2790
716.1218 721.5953 763.6144
765.8612 784.0391 789.6536
808.0574 811.2847 815.3964
858.2000 871.9145 876.2972
885.2412 909.4076 919.7356
947.9085 956.4474 970.0084
1004.7230 1007.4425 1019.6027
1031.3673 1051.2253 1055.5076
1059.5561 1060.3772 1067.5368
1074.6876 1083.5237 1095.8992
1100.2805 1136.8231 1156.4105
1198.3206 1201.8206 1203.6595
1207.9715 1208.9755 1216.4088
1223.7651 1233.2858 1238.8657
1243.1132 1249.0632 1254.9170
1262.0107 1265.8025 1270.2399
1271.2033 1273.3854 1283.2559
1290.5894 1292.7003 1298.4197
1298.7681 1311.3311 1313.6826
1324.2453 1336.8452 1351.0222
1362.3748 1378.5852 1387.2342
1394.3177 1399.9719 1408.9492
1416.8119 1483.4106 1495.7873
1499.9273 1502.6946 1503.5529
1506.5826 1518.2449 1518.7130
1521.0364 1541.2245 3025.3685
3026.7623 3032.1295 3045.0105
3045.3642 3049.2922 3059.7160
3079.9829 3080.3153 3080.3889
3086.1823 3087.3394 3100.6443

3113.5693 3124.8761 3135.3868
3139.7070 3150.4830 3445.6959

Ca(NTf$_2$)$_2$(Pip)(THF)$_2$ 7b

Gaussian 16: ES64L-G16RevB.01 20-Dec-2017

B3LYP/6-31G(d,p) gfprint gfinput scf=(direct,tight,maxcycle=300,xqc) opt=(maxcycle=250) freq=noraman
AN.EDU\29-Mar-2021\0\\# B3LYP/6-31G(d,p) gfprint gfinput scf=(direct,t
ight,maxcycle=300,xqc) opt=(maxcycle=250) freq=noraman\\010c_ca_2ntf2_

Full point group C1 NOp 1
Stoichiometry C17H27CaF12N3O10S4
Framework group C1[X(C17H27CaF12N3O10S4)]

Num atoms: 74
Charge = 0 Multiplicity = 1

SCF = -5048.91188421 l Predicted change in Energy=-1.032435D-08

Optimization completed.
Maximum Force 0.000012 0.000450 YES
RMS Force 0.000002 0.000300 YES
Maximum Displacement 0.001376 0.001800 YES
RMS Displacement 0.000300 0.001200 YES

Atom Coordinates (in Angstroms)
Type X Y Z

C -5.118180 -4.801093 -3.525570
F -6.086873 -4.500271 -4.388566
F -5.633015 -4.919306 -2.301741
F -4.534313 -5.940696 -3.878423
S -3.860982 -3.419576 -3.472163
O -2.844959 -3.780940 -2.474352
O -4.670577 -2.204583 -3.157078
N -3.383529 -3.488356 -5.016905
S -2.551785 -2.282887 -5.676027
O -2.650721 -1.005114 -4.909651
O -2.775237 -2.234175 -7.115858
C -0.750225 -2.739641 -5.449768
F -0.472536 -2.831840 -4.142427
F -0.488140 -3.899624 -6.041537
F 0.008585 -1.780025 -5.987435
C -5.332385 4.203376 -1.668227
F -5.970385 3.795137 -2.779123
F -6.113867 5.035331 -0.993181
F -4.212607 4.846207 -2.033424
S -4.882580 2.713219 -0.632672
O -4.242796 3.230106 0.568436
O -4.026757 1.927115 -1.570424
N -6.303395 2.065739 -0.287335
S -7.035979 0.895465 -1.119548
O -6.240368 0.335513 -2.247496
O -8.429431 1.219521 -1.396893
C -7.043470 -0.495477 0.128153
F -7.595855 -1.571320 -0.433678
F -5.782203 -0.781615 0.479016
F -7.735550 -0.149489 1.207864
Ca -4.052100 0.160566 -3.252068
C -2.065294 3.077559 -3.928768
O -2.882145 2.001217 -4.434130
C -2.732679 2.042618 -5.872618
C -2.671149 3.540720 -6.232071
C -2.334886 4.242263 -4.887217
H -2.362606 3.261280 -2.897939
H -1.010663 2.768228 -3.957348
H -1.807703 1.520899 -6.145992
H -3.578874 1.504256 -6.298342
H -1.912791 3.726056 -6.997047
H -3.627266 3.893697 -6.627159
H -3.185816 4.826687 -4.529187
H -1.475638 4.913433 -4.962007
C -6.038217 -0.726477 -5.988330
O -5.594575 0.417923 -5.193663
C -6.580291 1.481588 -5.278478
C -7.364376 1.189359 -6.552673
C -7.416386 -0.345886 -6.537131
H -5.301356 -0.884840 -6.781828
H -6.061588 -1.608018 -5.343919
H -7.221029 1.444380 -4.391110
H -6.039588 2.431830 -5.291402
H -8.353343 1.654401 -6.546766
H -6.819132 1.549635 -7.432206
H -7.599217 -0.782517 -7.522010
H -8.205922 -0.689172 -5.860902

C -2.365199 -0.725591 -0.281171
C -1.228682 -1.365517 0.525788
C 0.112467 -0.677243 0.231248
C 0.383453 -0.639103 -1.280145
C -0.802753 -0.026537 -2.036036
N -2.094102 -0.697262 -1.742991
H -2.524859 0.309658 0.041001
H -3.303286 -1.265694 -0.123675
H -1.163812 -2.431188 0.266854
H -1.465025 -1.312875 1.595001
H 0.929484 -1.187130 0.753812
H 0.084333 0.350590 0.619436
H 1.293533 -0.067420 -1.499029
H 0.552622 -1.657953 -1.654638
H -0.915924 1.029382 -1.758300
H -0.638433 -0.068808 -3.117473
H -2.005370 -1.672197 -2.039034

Statistical Thermodynamic Analysis
Temperature 298.150 Kelvin. Pressure 1.00000 Atm.

SCF = -5048.91188421 l Predicted change in Energy=-1.032435D-08
Zero-point correction (ZPE) = -5048.40270521 0.509179
Internal Energy (U) = -5048.34955821 0.562326
Enthalpy (H) = -5048.34861421 0.56327
Gibbs Free Energy (G) = -5048.50370821 0.408176

Frequencies
8.0365 13.1421 14.9818
17.8969 21.9862 26.9930
27.5606 29.9954 30.4274
34.0623 35.2111 36.7603
39.5957 39.9314 43.2156
44.3757 45.5015 50.3895
56.8669 60.4945 65.2088
67.6343 72.5175 76.4233
85.0235 86.5593 92.0444
97.9093 104.8599 107.4688
107.9403 111.4016 114.6140
122.3450 141.1784 163.5306
166.2865 181.5989 188.8716
198.8050 202.5367 205.0742
215.7172 221.2988 230.3690
239.6240 243.5030 249.9181
265.3196 270.9239 275.0090
282.6810 285.9979 287.5645
293.6544 294.1962 302.8155
311.6232 317.1550 322.4211
327.0213 327.2031 355.5883
394.3669 397.4821 400.3547
416.0059 447.1368 456.7300
461.3909 490.4549 498.2530
504.5495 521.7065 537.8735
538.4180 548.9809 549.9036
553.9992 561.3503 562.5746
569.0919 582.2311 586.6690
588.7615 591.0214 620.1133
636.6960 648.5109 670.8891
683.1997 720.7675 723.7208
759.8178 763.9204 781.2082
788.0278 808.9867 815.0930
816.7152 849.3905 860.2726
870.6128 878.0179 881.6111
885.3623 887.4604 910.8091
921.7378 927.6940 932.8462
949.1598 957.7273 969.5689
977.1326 1003.3254 1016.1489
1022.4989 1030.2797 1048.1828
1055.6513 1056.3807 1058.0820
1062.2612 1068.5689 1078.7532
1085.9544 1093.1541 1100.3904
1101.6012 1134.2472 1154.5723
1171.9646 1195.1682 1199.9934
1203.9945 1204.6014 1208.3387
1211.0637 1215.3038 1216.3528
1216.8202 1226.9666 1230.6514
1239.7792 1240.5527 1250.2230
1262.8268 1263.9140 1266.1044
1267.2429 1268.1480 1270.3241
1272.8651 1281.5802 1287.1091
1298.6848 1299.7328 1301.8261
1309.7445 1315.3479 1322.2341
1329.8430 1331.3931 1351.4641
1357.8212 1360.7557 1374.7829
1383.2935 1384.7799 1392.2225
1398.5669 1407.4240 1412.7748
1415.2372 1481.0659 1499.2486
1501.3303 1501.4724 1503.9056
1504.4079 1506.2048 1511.4725
1520.0814 1520.5414 1523.6151
1536.4657 1542.5692 1549.0422
3019.4640 3024.7319 3028.9792
3032.2474 3039.4812 3051.9617

3060.2978 3060.8760 3064.0011
3071.8371 3072.4613 3074.9794
3077.3975 3079.3437 3084.7295
3088.3777 3097.1688 3114.7147
3116.5213 3116.9551 3122.7796
3129.5701 3137.4813 3138.9991
3160.0790 3176.2756 3457.6111

$Ca(NTf_2)_2(Pip)(THF)_3$ 7c

Gaussian 16: ES64L-G16RevB.01 20-Dec-2017

B3LYP/6-31G(d,p) gfprint gfinput scf=(direct,tight,maxcycle=300,xqc) opt=(maxcycle=250) freq=noraman
AN.EDU\26-Mar-2021\0\\# B3LYP/6-31G(d,p) gfprint gfinput scf=(direct,t
ight,maxcycle=300,xqc) opt=(maxcycle=250) freq=noraman\\010c_ca_2ntf2_

Full point group C1 NOp 1
Stoichiometry C21H35CaF12N3O11S4
Framework group C1[X(C21H35CaF12N3O11S4)]

Num atoms: 87
Charge = 0 Multiplicity = 1

SCF = -5281.38218758 l Predicted change in Energy=-8.731104D-09

Optimization completed.
Maximum Force 0.000028 0.000450 YES
RMS Force 0.000005 0.000300 YES
Maximum Displacement 0.000260 0.001800 YES
RMS Displacement 0.000040 0.001200 YES

Atom Coordinates (in Angstroms)
Type X Y Z

Ca -4.318394 -0.387206 -0.901155
S -7.599076 -2.332393 -1.181823
O -8.770221 -1.845017 -1.903027
O -6.656643 -1.314426 -0.638760
N -6.896083 -3.512654 -2.006695
S -5.342013 -3.920160 -1.836125
O -5.098775 -5.069814 -0.964908
O -4.414746 -2.765715 -1.684132
C -5.023837 -4.511969 -3.579731
F -5.744297 -5.593789 -3.850028
F -5.310005 -3.552152 -4.467872
F -3.721061 -4.806397 -3.673906
C -8.253909 -3.151859 0.367008
F -9.159911 -4.073222 0.056377
F -7.243839 -3.728612 1.031738
F -8.812933 -2.222542 1.148945
S -1.999860 2.015190 0.901515
O -1.096099 1.594507 1.967635
O -3.237121 1.215720 0.699718
N -1.169711 2.325074 -0.439695
S -1.652980 2.003698 -1.944123
O -3.021344 1.434350 -2.070271
O -0.582886 1.353455 -2.698651
C -1.828279 3.698266 -2.717641
F -2.748129 4.416795 -2.065310
F -0.665286 4.341888 -2.691043
F -2.221066 3.541205 -3.987164
C -2.657250 3.674942 1.463856
F -3.457212 4.206910 0.528727
F -3.372896 3.490229 2.579832
F -1.656411 4.513298 1.711748
C -0.157669 -2.637936 -0.205477
C -0.885579 -1.289139 -0.066754
C -1.393052 -1.989002 -2.198699
C -0.542416 -3.132078 -1.627156
H 0.921192 -2.512990 -0.083887
H -0.494356 -3.348275 0.554647
H -0.220780 -0.450044 -0.301785
H -1.340908 -1.117291 0.908255
H -2.224247 -2.333503 -2.813863
H -0.786275 -1.264165 -2.754618
H 0.330865 -3.331447 -2.253462
H -1.130950 -4.051333 -1.570413
O -1.953302 -1.329676 -1.041334
C -4.679431 0.717958 -5.464111
C -6.213087 0.613148 -5.248573
C -6.355127 -0.232524 -3.964041
O -5.047169 -0.229158 -3.350428
C -4.111864 -0.275532 -4.447954
H -4.376496 0.478216 -6.486557
H -4.320402 1.724219 -5.231974
H -6.714906 0.125446 -6.088255

H -6.662058 1.602370 -5.128332
H -7.066244 0.168081 -3.241084
H -6.640999 -1.265654 -4.190613
H -4.085616 -1.296665 -4.851267
H -3.130673 -0.004311 -4.060798
C -3.385096 -1.559060 3.547513
C -3.094352 -2.951905 2.928451
C -3.814235 -2.920420 1.561788
O -4.165597 -1.534666 1.336919
C -4.471217 -0.987914 2.633603
H -3.711622 -1.617172 4.589087
H -2.498383 -0.920489 3.506358
H -3.483120 -3.764003 3.548460
H -2.019451 -3.114775 2.814268
H -3.203130 -3.237403 0.717736
H -4.728771 -3.522540 1.573696
H -5.473972 -1.320779 2.939435
H -4.454192 0.097861 2.546278
C -6.003640 2.723803 -1.453194
C -7.194458 3.689025 -1.388597
C -7.497537 4.084530 0.063989
C -7.688987 2.832125 0.931508
C -6.487320 1.886172 0.801076
H -5.093229 3.231495 -1.122666
H -5.820001 2.386236 -2.477319
H -8.078771 3.207357 -1.830329
H -6.981278 4.575882 -1.997488
H -8.383655 4.727145 0.113275
H -6.657491 4.672821 0.459067
H -7.827717 3.104675 1.984696
H -8.600776 2.303283 0.619828
H -5.593970 2.355198 1.226558
H -6.666865 0.958646 1.354794
N -6.169671 1.521747 -0.601406
H -6.963476 0.984108 -0.951995

Statistical Thermodynamic Analysis
Temperature 298.150 Kelvin. Pressure 1.00000 Atm.

SCF = -5281.38218758 l Predicted change in Energy=-8.731104D-09
Zero-point correction (ZPE) = -5280.75405458 0.628133
Internal Energy (U) = -5280.69420758 0.68798
Enthalpy (H) = -5280.69326358 0.688924
Gibbs Free Energy (G) = -5280.86394058 0.518247

Frequencies
3.4002 14.0941 18.0975
22.6632 25.5184 26.5019
28.8243 31.2671 32.6333
35.3086 36.2679 37.6267
39.6790 40.4491 41.1060
43.8433 45.6225 47.5792
51.4311 53.1217 55.8160
61.6951 63.7680 65.8091
67.7301 72.6100 74.5945
76.7892 80.7532 85.4449
89.1304 94.9652 96.8469
97.8177 104.3726 105.7286
110.1532 115.9223 120.5432
122.6259 126.7933 135.7655
138.6051 175.2301 183.1391
186.5261 195.7302 199.7960
203.6227 210.9187 215.7569
219.9862 227.3004 238.2247
248.8044 274.3836 276.3654
278.2861 283.0018 287.0055
288.5640 292.7907 294.5954
298.7137 312.8347 315.9685
316.1514 321.7608 322.5866
331.6491 343.3667 393.4298
394.8362 404.7688 415.1862
425.2696 453.1036 461.3285
492.5160 493.6269 512.6515
520.8533 537.8531 539.7129
548.2814 550.0106 554.2562
560.1059 560.2438 562.8262
578.2435 581.1746 590.5461
624.5676 626.0598 634.5919
638.5748 645.1447 659.0824
672.8876 681.2901 716.1891
720.2680 760.1948 761.9438
779.9867 782.2365 805.2486
806.0595 809.9342 813.1386
816.9309 854.3786 868.4026
873.0962 874.9014 875.2121
885.3114 911.0787 911.4579
912.0067 920.8957 924.6425
928.9240 946.9611 947.7171
951.8947 956.9382 967.8635
971.1889 972.1971 980.9654
1012.2798 1016.1912 1027.5490
1056.3902 1056.9380 1059.2461
1063.1932 1063.4811 1064.1146

1066.0387 1070.2314 1078.9803
1086.8051 1098.3316 1104.3805
1105.6676 1133.6368 1150.7085
1152.2819 1152.4954 1202.8922
1203.1757 1204.5609 1215.0321
1215.2876 1216.4306 1221.4404
1224.1988 1224.2775 1228.0673
1229.2235 1232.4873 1241.4041
1243.1422 1243.9036 1245.2425
1255.9014 1257.9622 1258.3464
1262.8073 1263.6218 1264.2747
1266.6574 1281.9873 1283.9508
1287.3571 1288.7457 1291.8949
1294.5747 1300.2002 1306.7975
1308.4904 1319.1038 1321.1472
1325.3899 1333.5807 1337.1519
1339.2652 1349.1767 1359.5967
1376.9544 1377.5186 1381.0217
1383.9641 1391.7862 1396.9092
1410.7539 1412.9753 1413.7716
1416.9637 1482.8024 1498.2027
1500.2334 1501.2404 1501.4834
1503.2378 1503.8144 1504.9733
1515.0313 1516.1423 1517.4673
1518.2932 1520.0431 1520.8764
1522.0126 1536.6662 1538.4027
1541.1063 3013.3079 3018.2789
3022.6692 3028.1349 3038.1178
3054.6066 3059.6314 3061.2970
3065.5315 3069.6668 3072.9144
3073.1366 3074.5955 3075.1065
3075.9087 3080.7577 3083.3207
3083.5116 3083.7119 3084.8018
3111.4574 3113.8549 3114.3584
3115.2827 3128.1692 3132.8208
3132.9710 3133.8190 3149.5197
3156.9253 3159.7102 3161.5150
3165.3908 3167.6383 3480.0582

$Ca(NTf_2)_2(PhSO_2F)(DABCO)$ 8a

Gaussian 16: ES64L-G16RevB.01 20-Dec-2017

B3LYP/6-31G(d,p) gfprint gfinput scf=(direct,tight,maxcycle=300,xqc) opt=(maxcycle=250) freq=noraman
AN.EDU\05-Apr-2021\0\\# B3LYP/6-31G(d,p) gfprint gfinput scf=(direct,tight,maxcycle=300,xqc) opt=(maxcycle=250) freq=noraman\\020b_ca_2ntf2_

Full point group C1 NOp 1
Stoichiometry C16H17CaF13N4O10S5
Framework group C1[X(C16H17CaF13N4O10S5)]

Num atoms: 66
Charge = 0 Multiplicity = 1

SCF = -5557.45830483 l Predicted change in Energy=-1.385352D-08

Optimization completed.
Maximum Force 0.000025 0.000450 YES
RMS Force 0.000003 0.000300 YES
Maximum Displacement 0.001503 0.001800 YES
RMS Displacement 0.000278 0.001200 YES

Atom Coordinates (in Angstroms)
Type X Y Z

C -4.990480 5.427593 -2.695056
F -4.980895 5.705984 -1.397393
F -6.249366 5.304499 -3.120040
F -4.388623 6.410395 -3.373686
S -4.086116 3.825216 -3.025420
O -4.223112 3.670436 -4.506206
O -4.724609 2.793652 -2.216737
N -2.602631 4.150438 -2.506560
S -1.306797 4.425738 -3.412980
O -0.385166 5.328054 -2.743957
O -1.585743 4.687183 -4.860372
C -0.480297 2.749887 -3.476190
F -1.345091 1.858224 -3.986528
F 0.589145 2.819756 -4.277029
F -0.104540 2.361839 -2.265576
C -7.330498 4.432309 -7.028674
F -8.427757 5.123774 -7.319583
F -7.665876 3.190027 -6.672094
F -6.690031 5.021780 -6.009891
S -6.198627 4.355350 -8.520567
O -6.951809 3.674464 -9.560300

O -4.990970 3.655328 -7.978001
N -5.919237 5.877456 -8.941057
S -4.762402 6.816608 -8.328061
O -3.901337 7.371274 -9.369459
O -4.066786 6.258655 -7.124499
C -5.752090 8.242294 -7.629193
F -6.623327 7.783984 -6.728651
F -4.908181 9.089523 -7.041287
F -6.401970 8.860887 -8.608710
Ca -3.152605 4.151112 -6.550448
N 0.758007 4.714501 -9.867545
C -0.452223 4.332136 -10.610709
C -1.665933 4.177813 -9.640947
C -0.673340 5.808668 -8.145015
C 0.506086 5.975597 -9.152949
C 1.070924 3.664694 -8.888085
C -0.126702 3.456294 -7.909963
H -0.651409 5.099356 -11.365527
H -0.248706 3.395046 -11.139219
H -2.453088 4.901527 -9.872517
H -2.102853 3.174917 -9.691816
H -1.488405 6.504673 -8.360580
H -0.352855 5.971219 -7.110846
H 0.281044 6.750225 -9.892842
H 1.426357 6.269649 -8.637674
H 1.299661 2.741589 -9.430939
H 1.974974 3.959484 -8.345281
H -0.539697 2.445739 -7.986828
H 0.165016 3.617654 -6.867639
N -1.222115 4.419197 -8.234055
C -6.181464 -0.214773 -7.448989
C -5.174513 0.318132 -6.639902
C -5.384702 0.672878 -5.303937
C -6.654601 0.482427 -4.765967
C -7.677537 -0.049745 -5.555374
C -7.444595 -0.394269 -6.889219
H -5.981161 -0.461761 -8.484967
H -4.594980 1.107409 -4.704522
H -6.837236 0.763858 -3.734413
H -8.666323 -0.190301 -5.129933
H -8.246985 -0.796408 -7.498537
S -3.575746 0.553257 -7.348119
O -2.921451 1.734731 -6.764733
F -2.753405 -0.669029 -6.693579
O -3.565119 0.349295 -8.779729

Statistical Thermodynamic Analysis
Temperature 298.150 Kelvin. Pressure
1.00000 Atm.

SCF = -5557.45830483 l Predicted change in
Energy=-1.385352D-08
Zero-point correction (ZPE) = -5557.05848883
0.399816
Internal Energy (U) = -5557.00737083
0.450934
Enthalpy (H) = -5557.00642683 0.451878
Gibbs Free Energy (G) = -5557.15723983
0.301065

Frequencies
7.9000 8.5721 12.8448
16.7579 17.5543 20.8403
23.4488 26.7003 28.7233
30.5088 33.5880 35.5810
37.3380 38.7160 40.1009
48.2127 48.4325 52.4161
56.9781 61.3460 63.8784
74.1985 85.0963 89.5372
93.1023 103.7106 105.5867
110.3115 118.9792 126.6007
129.1272 176.9155 183.4562
186.4209 192.0297 197.2166
201.8484 207.5488 210.5999
214.3221 238.2127 246.2331
268.9253 269.1610 275.8141
280.1692 282.4942 289.0151
295.2578 297.4214 310.3529
312.3253 314.2743 323.5809
325.7664 329.2924 333.3793
335.1473 342.1010 346.6424
391.6816 397.3946 400.8967
408.8434 420.5237 424.7367
426.0396 433.3287 485.3889
498.1637 502.5116 515.5079
517.2400 522.9127 537.1329
538.7222 548.9484 549.3664
560.7277 564.2821 566.5531
573.3339 578.3823 584.4323
586.4204 587.4635 591.2494
612.2577 621.6322 622.8384
631.0812 685.5813 718.5387
719.3555 720.5538 761.6181
762.2283 762.6595 782.0245
782.6748 784.3876 803.3994
810.8121 820.5138 842.7895
843.9345 854.9927 906.1581
908.7964 947.1431 978.1772

991.6208 1004.8591 1010.4615
1011.5141 1020.4169 1021.4159
1023.0785 1026.0144 1032.9690
1043.7585 1064.4375 1074.0094
1077.8591 1079.5827 1081.8213
1088.2024 1094.9293 1114.7281
1162.0669 1194.3287 1200.9308
1204.4013 1207.1153 1208.1860
1208.7761 1209.5651 1212.2437
1212.5739 1227.0717 1231.5660
1244.7134 1255.0626 1265.4872
1270.5357 1272.0523 1274.0582
1278.2875 1287.2433 1295.6615
1312.1462 1320.1351 1337.7817
1340.5832 1343.8242 1347.4496
1348.8655 1353.1662 1357.5910
1360.4602 1364.3204 1365.6560
1370.2261 1383.5946 1409.6678
1490.1725 1497.7543 1499.9041
1508.5837 1512.9293 1513.7953
1515.4402 1528.5065 1635.3606
1639.8720 3051.3794 3053.4354
3055.8887 3060.7130 3064.6609
3067.9800 3094.0113 3097.0351
3100.0969 3117.4229 3121.4767
3127.6613 3199.4690 3213.9678
3220.9818 3233.8686 3248.7575

$Ca(NTf_2)_2(PhSO_2F)(DABCO)(THF)$ 8b

Gaussian 16: ES64L-G16RevB.01 20-Dec-2017

B3LYP/6-31G(d,p) gfprint gfinput scf=(direct,tight,maxcycle=300,xqc) opt=(maxcycle=250) freq=noraman AN.EDU\04-Apr-2021\0\\# B3LYP/6-31G(d,p) gfprint gfinput scf=(direct,t ight,maxcycle=300,xqc) opt=(maxcycle=250) freq=noraman\\020b_ca_2ntf2_

Full point group C1 NOp 1
Stoichiometry C20H25CaF13N4O11S5
Framework group
C1[X(C20H25CaF13N4O11S5)]

Num atoms: 79
Charge = 0 Multiplicity = 1

SCF = -5789.94169011 l Predicted change in Energy=-2.740762D-08

Optimization completed.
Maximum Force 0.000024 0.000450 YES
RMS Force 0.000003 0.000300 YES
Maximum Displacement 0.001693 0.001800 YES
RMS Displacement 0.000290 0.001200 YES

Atom Type	Coordinates (in Angstroms) X	Y	Z
C	-3.482190	5.127462	-3.258729
F	-3.743841	3.995311	-2.585592
F	-4.641668	5.687535	-3.627562
F	-2.818758	5.965289	-2.473085
S	-2.483549	4.711513	-4.784319
O	-2.140913	5.977673	-5.414147
O	-3.406419	3.807291	-5.532337
N	-1.172891	4.010622	-4.185182
S	-0.982576	2.441170	-3.896060
O	-2.089914	1.566117	-4.379846
O	-0.490195	2.181065	-2.549194
C	0.429373	2.032633	-5.051811
F	0.633712	0.708919	-5.025720
F	0.118150	2.393932	-6.303744
F	1.536113	2.658366	-4.672224
C	-6.518667	2.648957	-9.926672
F	-5.294728	2.611660	-10.456206
F	-7.395586	2.146765	-10.788800
F	-6.838384	3.917395	-9.652846
S	-6.569104	1.696987	-8.319561
O	-7.932665	1.810152	-7.796742
O	-5.496156	2.329953	-7.495014
N	-6.147326	0.252143	-8.911407
S	-5.626649	-0.925812	-7.954308
O	-5.218511	-0.456796	-6.594849
O	-4.713176	-1.807833	-8.672800
C	-7.144532	-1.952062	-7.572036
F	-8.076248	-1.182437	-7.008006
F	-7.623897	-2.495665	-8.686397
F	-6.798413	-2.919874	-6.714653
Ca	-4.186552	1.510040	-5.600264
N	-4.938949	-1.766514	-1.526975

C -4.550557 -0.437239 -1.031182
C -4.358475 0.557642 -2.217938
C -6.021208 -0.663591 -3.484517
C -6.195759 -1.641153 -2.280935
C -3.889703 -2.263936 -2.429451
C -3.690119 -1.287886 -3.631823
H -5.327981 -0.087776 -0.343391
H -3.625499 -0.541731 -0.454539
H -5.043240 1.408211 -2.147138
H -3.339009 0.948030 -2.251671
H -6.703590 0.187737 -3.415350
H -6.206965 -1.162037 -4.438162
H -6.973086 -1.288237 -1.594860
H -6.491047 -2.638508 -2.624182
H -2.964549 -2.372103 -1.853455
H -4.179698 -3.261732 -2.775788
H -2.672341 -0.887841 -3.660660
H -3.891995 -1.779793 -4.588628
N -4.626288 -0.131271 -3.515142
C -6.771401 5.106744 -6.110213
C -7.808263 4.409517 -5.484833
C -9.155175 4.622446 -5.787904
C -9.468691 5.576847 -6.752312
C -8.450354 6.290971 -7.388052
C -7.110143 6.056836 -7.070827
H -5.735768 4.902137 -5.869294
H -9.926518 4.044353 -5.292926
H -10.506835 5.755876 -7.011521
H -8.702747 7.031421 -8.140804
H -6.323270 6.608325 -7.574357
S -7.420229 3.206374 -4.252971
O -6.095960 2.624911 -4.483770
F -7.189549 4.144474 -2.956193
O -8.548842 2.354321 -3.945827
C -1.558470 0.891930 -9.521235
C -2.292491 1.808082 -8.546366
O -2.583604 0.973835 -7.390817
C -1.767962 -0.233992 -7.428428
C -0.772877 -0.023044 -8.569855
H -2.276673 0.308593 -10.106555
H -0.919331 1.448833 -10.211320
H -3.238197 2.195012 -8.927118
H -1.663833 2.645085 -8.222073
H -1.288287 -0.350145 -6.453446
H -2.432005 -1.082427 -7.619568
H -0.471035 -0.967215 -9.029794
H 0.124028 0.485760 -8.203020

Statistical Thermodynamic Analysis

Temperature 298.150 Kelvin. Pressure 1.00000 Atm.

SCF = -5789.94169011 l Predicted change in Energy=-2.740762D-08
Zero-point correction (ZPE) = -5789.42276111 0.518929
Internal Energy (U) = -5789.36518611 0.576504
Enthalpy (H) = -5789.36424211 0.577448
Gibbs Free Energy (G) = -5789.53010411 0.411586

Frequencies
0.6988 12.2576 15.3906
18.7529 20.9264 25.8016
27.4174 29.6889 30.5027
33.9236 35.1583 37.1270
38.6091 41.7759 42.5740
46.0730 51.2463 54.5077
55.7130 59.8489 60.6437
64.4807 68.0554 77.0175
79.5944 82.8492 86.8175
92.4080 97.5913 100.3218
102.1559 104.3804 107.5632
108.1680 112.7353 116.8728
125.0226 167.7993 170.4945
176.5573 188.3762 192.7247
198.2445 201.4114 205.4213
212.0561 222.2564 229.5253
232.6482 255.8506 270.2271
271.6632 276.0802 278.8970
285.0021 286.4232 292.5175
297.2583 312.5006 312.6752
319.0304 322.5692 329.4468
331.9651 333.6424 336.7183
340.0304 351.0903 393.7239
399.8276 402.1640 410.8649
427.4424 428.2341 435.0418
444.1961 476.6538 489.6589
498.5582 504.0509 515.9849
521.9317 537.8511 538.5181
548.7541 550.1566 553.3543
561.1481 563.1509 568.0552
576.2719 580.9695 586.9424
587.6824 588.7025 591.6518
615.0773 618.3504 623.4332
647.0577 686.3163 687.8381
719.1977 720.0520 722.8005

759.9571 760.4875 763.9316
777.4357 780.1405 786.5458
802.2163 804.8698 821.9235
843.6712 844.6491 853.5689
858.1818 881.8766 892.5548
906.5273 911.0110 921.9146
931.6842 946.6296 980.5779
980.7912 986.3673 1003.1436
1006.8198 1011.5686 1016.3813
1022.6371 1026.6579 1027.3137
1033.7677 1046.5519 1046.9003
1052.5247 1061.5514 1076.3037
1077.6000 1084.1992 1089.1203
1091.4471 1097.4562 1116.2153
1174.6103 1178.5662 1194.8015
1198.1308 1200.0493 1202.6596
1203.6140 1208.7975 1209.4163
1210.4618 1211.0615 1212.3861
1213.8283 1223.0777 1226.4633
1239.6388 1246.2276 1260.3351
1265.9308 1266.2363 1269.0466
1272.7271 1274.8458 1275.3386
1282.9773 1296.8175 1297.5116
1314.1134 1331.0249 1336.4866
1338.7106 1344.2807 1345.7376
1347.3895 1354.8903 1355.4353
1357.7015 1361.0096 1361.7201
1363.0011 1372.4449 1381.5652
1385.1670 1405.1837 1415.4562
1491.3791 1499.5972 1500.3851
1501.1459 1510.5186 1513.2931
1514.5712 1515.4425 1519.3606
1530.3996 1536.6109 1549.3620
1637.7651 1641.6238 3047.5405
3048.2966 3052.0882 3058.8547
3062.8919 3071.1009 3071.8649
3072.4194 3074.6352 3085.5316
3090.3630 3093.6983 3095.7401
3119.0925 3121.6608 3126.2725
3134.9260 3137.3298 3142.0328
3152.8023 3196.9361 3210.9764
3217.9980 3233.7105 3243.4361

$Ca(NTf_2)_2(PhSO_2F)(DABCO)(THF)_2$ 8c

Gaussian 16: ES64L-G16RevB.01 20-Dec-2017

# B3LYP/6-31G(d,p) gfprint gfinput
scf=(direct,tight,maxcycle=300,xqc)
opt=(maxcycle=250) freq=noraman
AN.EDU\16-Mar-2021\0\\# B3LYP/6-31G(d,p)
gfprint gfinput scf=(direct,t
ight,maxcycle=300,xqc) opt=(maxcycle=250)
freq=noraman\\020b_ca_2ntf2_

Full point group C1 NOp 1
Stoichiometry C24H33CaF13N4O12S5
Framework group
C1[X(C24H33CaF13N4O12S5)]

Num atoms: 92
Charge = 0 Multiplicity = 1

SCF = -6022.41126729 l Predicted change in Energy=-4.072436D-09

Optimization completed.
Maximum Force 0.000007 0.000450 YES
RMS Force 0.000001 0.000300 YES
Maximum Displacement 0.001005 0.001800 YES
RMS Displacement 0.000188 0.001200 YES

Atom Coordinates (in Angstroms)
Type X Y Z

Ca -1.884383 1.740300 0.800862
S -2.231403 -0.212955 -2.358741
O -1.806038 0.983711 -1.573474
O -3.589711 -0.196017 -2.903522
N -1.744949 -1.613589 -1.715249
S -1.949935 -1.965488 -0.162076
O -2.154283 -0.781369 0.712702
O -0.955346 -2.948067 0.266343
C -3.596694 -2.852496 -0.083506
F -4.562626 -2.049776 -0.543131
F -3.555543 -3.960983 -0.816871
F -3.857571 -3.167281 1.188499
C -1.076236 -0.188699 -3.827216
F -1.350212 0.914304 -4.532201
F 0.196013 -0.133518 -3.419651
F -1.256334 -1.260050 -4.589618

S	-3.365957	4.547002	2.839726
O	-3.294837	3.610154	1.683217
O	-3.273968	5.973514	2.526582
N	-2.513147	4.079539	4.130558
S	-1.084812	3.366740	4.030693
O	-0.885275	2.435374	5.139527
O	-0.719827	2.908922	2.659554
C	0.145322	4.745144	4.319170
F	1.383949	4.253851	4.178049
F	-0.040817	5.715158	3.417589
F	0.006525	5.244955	5.543274
C	-5.081269	4.272619	3.531667
F	-5.238591	3.004667	3.915083
F	-5.304421	5.082649	4.560579
F	-5.959505	4.541313	2.555056
C	2.060290	-0.902472	0.596316
C	1.260333	0.152871	-0.155041
C	1.325112	0.824782	2.110488
C	2.507197	-0.119779	1.840941
H	1.407630	-1.739776	0.861182
H	2.896301	-1.290498	0.008263
H	0.528868	-0.253025	-0.851236
H	1.920512	0.842205	-0.700175
H	1.633727	1.836569	2.381290
H	0.662501	0.450260	2.893939
H	2.716493	-0.761641	2.700666
H	3.415464	0.452419	1.624150
O	0.550836	0.883079	0.873672
C	-6.387212	1.637795	-0.961591
C	-6.666500	1.405369	0.530212
C	-5.310960	0.919663	1.057155
O	-4.309631	1.420951	0.126860
C	-4.961773	2.174638	-0.923674
H	-6.413689	0.694930	-1.517359
H	-7.086035	2.336986	-1.428592
H	-7.463436	0.680549	0.715435
H	-6.943310	2.346853	1.016153
H	-5.089036	1.307214	2.052272
H	-5.231735	-0.170133	1.068126
H	-4.404335	2.005309	-1.845138
H	-4.943912	3.241412	-0.663116
C	0.109759	-2.575068	4.368541
C	-0.961767	-1.846951	3.855755
C	-1.718063	-1.077012	4.744602
C	-1.433767	-1.002274	6.108343
C	-0.349406	-1.730406	6.596592
C	0.415255	-2.514926	5.731578
H	0.699689	-3.193248	3.699664
H	-1.201973	-1.884124	2.799563

H	-2.042771	-0.386787	6.759548
H	-0.105697	-1.683570	7.652812
H	1.253448	-3.084820	6.120781
S	-3.084793	-0.127420	4.135081
O	-2.675801	0.752721	3.043164
F	-3.924024	-1.301951	3.400569
O	-3.912972	0.369591	5.210923
N	-0.896019	3.856865	-0.681207
C	-0.555140	5.039674	0.161196
C	0.015558	6.187960	-0.727768
C	-1.368329	5.466364	-2.540777
C	-1.915689	4.283250	-1.681430
C	0.334393	3.439836	-1.410527
C	0.851230	4.604754	-2.311450
H	-1.462692	5.354703	0.678848
H	0.162120	4.716274	0.918675
H	-0.579258	7.100817	-0.618010
H	1.045490	6.433757	-0.446678
H	-1.984355	6.363211	-2.415937
H	-1.370220	5.212864	-3.606605
H	-2.815912	4.571399	-1.129835
H	-2.157616	3.413931	-2.298499
H	1.074333	3.151060	-0.657792
H	0.092119	2.551372	-1.998519
H	1.882584	4.873737	-2.057675
H	0.838487	4.320010	-3.369321
N	0.009674	5.799148	-2.147477

Statistical Thermodynamic Analysis
Temperature 298.150 Kelvin. Pressure 1.00000 Atm.

SCF = -6022.41126729 l Predicted change in Energy=-4.072436D-09
Zero-point correction (ZPE) = -6021.77368829 0.637579
Internal Energy (U) = -6021.70943029 0.701837
Enthalpy (H) = -6021.70848629 0.702781
Gibbs Free Energy (G) = -6021.88737529 0.523892

Frequencies
8.3483 11.2928 17.8727
20.5399 23.3796 26.5265
27.1549 28.7745 29.7568
31.1904 32.8868 35.9886
36.9639 38.9746 41.5750
45.6097 47.2576 48.6780

50.0676 51.7355 52.9745
55.4931 60.0079 63.6476
67.6108 71.6832 76.7488
78.7292 82.6060 85.1908
89.7436 90.4018 94.5702
98.5507 102.3671 103.2604
105.4396 110.9129 112.1045
114.6779 119.7409 121.3831
129.0569 135.2274 171.6542
173.2054 176.2221 179.5921
181.8405 185.1236 186.4083
198.2802 209.1603 214.9074
222.8495 230.4790 239.9659
272.2807 276.2085 276.7902
278.5443 280.2546 283.4344
286.9840 293.1236 296.0224
305.5726 316.2726 317.9019
324.6770 328.8409 338.5044
338.9913 339.3318 340.1508
348.4401 392.5597 398.8781
408.2511 423.5945 427.1100
427.2846 429.6333 436.2691
472.2461 490.2612 492.6700
507.5735 511.3037 520.6026
537.9260 539.4646 549.6262
549.9422 553.9573 553.9968
560.0544 561.9258 571.3822
584.8232 586.1850 587.6226
588.9602 590.5780 592.0242
611.9028 624.1114 640.5751
641.1601 683.1540 686.6951
691.3490 717.1032 720.2528
721.7057 761.3292 761.6434
763.0230 776.2816 783.0464
783.6997 792.9628 806.9741
823.9436 834.0725 844.9163
846.8138 853.2627 857.4757
881.3498 885.0388 892.1593
899.3018 906.0667 910.7146
921.7062 924.8012 933.7148
934.9131 942.7554 973.7413
980.8835 986.0545 987.2439
1002.7132 1009.6391 1012.9614
1015.5818 1015.7926 1028.5652
1031.6733 1035.1430 1045.6100
1049.7153 1050.0762 1059.4612
1059.5052 1069.1900 1073.4019
1078.9041 1080.5620 1094.3502
1095.2267 1101.7214 1113.0787
1167.8257 1174.4973 1179.4706
1192.6592 1195.5852 1197.6341
1201.6444 1202.2630 1206.7926
1209.5043 1209.8114 1209.9819
1210.5054 1213.1665 1213.7418
1218.4145 1232.6199 1235.6960
1237.6445 1239.7645 1258.6299
1258.9534 1260.7250 1262.3604
1264.3174 1267.4722 1273.1848
1275.5029 1276.4899 1285.4177
1286.9362 1291.0446 1295.0332
1329.1492 1331.0394 1334.0304
1337.2190 1343.2532 1344.6715
1346.4686 1353.8922 1354.4353
1355.0748 1357.9358 1359.8219
1361.1982 1362.1826 1375.3265
1380.0664 1381.1008 1382.3781
1403.3499 1410.9813 1413.3452
1489.5159 1499.0684 1500.0226
1501.3798 1504.4026 1510.4985
1513.0906 1513.4944 1514.4516
1515.1436 1518.7474 1527.0456
1530.9146 1531.5643 1545.5387
1546.2971 1638.1600 1641.1999
3019.9445 3029.5140 3043.1923
3046.3914 3050.0796 3063.9447
3065.9983 3068.2405 3069.1627
3071.4237 3077.7551 3086.9821
3087.9109 3090.8041 3091.9227
3092.0518 3094.9356 3113.9450
3119.2281 3123.7293 3128.2268
3129.7004 3130.2164 3139.9811
3150.7741 3150.8765 3152.6036
3175.6723 3194.6298 3208.5946
3215.8225 3234.8387 3236.8683

$Ca(NTf_2)_2(PhSO_2F)(Pip)$ 9a

Gaussian 16: ES64L-G16RevB.01 20-Dec-2017

B3LYP/6-31G(d,p) gfprint gfinput scf=(direct,tight,maxcycle=300,xqc) opt=(maxcycle=250) freq=noraman
AN.EDU\09-Apr-2021\0\\# B3LYP/6-31G(d,p) gfprint gfinput scf=(direct,t ight,maxcycle=300,xqc) opt=(maxcycle=250) freq=noraman\\020d_ca_2ntf2_

Full point group C1 NOp 1
Stoichiometry C15H16CaF13N3O10S5
Framework group
C1[X(C15H16CaF13N3O10S5)]

Num atoms: 63
Charge = 0 Multiplicity = 1

SCF = -5464.03444317 l Predicted change in Energy=-9.612137D-09

Optimization completed.
Maximum Force 0.000012 0.000450 YES
RMS Force 0.000003 0.000300 YES
Maximum Displacement 0.001412 0.001800 YES
RMS Displacement 0.000274 0.001200 YES

Atom Coordinates (in Angstroms)
Type X Y Z

C	-0.375840	-3.601171	-6.922815
F	-0.321669	-4.913067	-7.120023
F	-0.525208	-3.347307	-5.621772
F	0.746624	-3.029040	-7.360982
S	-1.836658	-2.872403	-7.834231
O	-1.743268	-1.413478	-7.524421
O	-3.032310	-3.551548	-7.341130
N	-1.474771	-3.270661	-9.350243
S	-1.336186	-2.268647	-10.590403
O	-0.560752	-2.879212	-11.656625
O	-0.996064	-0.856237	-10.225046
C	-3.086913	-2.088419	-11.224956
F	-3.550346	-3.250342	-11.664313
F	-3.868252	-1.645639	-10.222796
F	-3.107862	-1.191418	-12.213773
C	1.351090	-0.161344	-5.104170
F	2.075544	-1.077170	-5.746186
F	1.836440	0.020945	-3.882442
F	0.079263	-0.581441	-5.024163
S	1.388384	1.443404	-6.066966
O	0.820443	1.030836	-7.389477
O	2.760685	1.921425	-6.056788
N	0.453208	2.441390	-5.228760
S	-1.150913	2.522320	-5.278950
O	-1.811102	2.106800	-4.046704
O	-1.761799	2.025616	-6.555008
C	-1.380030	4.372361	-5.404343
F	-2.690445	4.624401	-5.439729
F	-0.835814	4.979176	-4.356868
F	-0.817211	4.826948	-6.529726
Ca	-1.258946	0.703981	-8.448110
C	-5.412748	1.417857	-5.398817
C	-5.053330	0.736227	-6.564216
C	-4.872521	-0.649648	-6.605789
C	-5.060872	-1.372576	-5.431423
C	-5.420955	-0.712153	-4.253583
C	-5.595610	0.673671	-4.235611
H	-5.523039	2.495711	-5.404736
H	-4.578299	-1.154192	-7.517298
H	-4.903571	-2.445363	-5.444745
H	-5.556924	-1.281467	-3.339407
H	-5.861409	1.179606	-3.313720
S	-4.773090	1.680186	-8.026617
O	-3.634364	1.131387	-8.783333
F	-6.036288	1.256386	-8.933305
O	-4.874030	3.104733	-7.798600
C	-0.400052	5.045537	-9.889812
C	0.693791	4.036105	-9.511527
C	0.688822	2.830836	-10.453076
N	-0.655429	2.190100	-10.474309
C	-1.695760	3.147372	-10.937467
C	-1.766358	4.352443	-9.997269
H	-0.151675	5.508389	-10.854928
H	-0.440892	5.854201	-9.152935
H	0.537417	3.691484	-8.483284
H	1.683209	4.505043	-9.537056
H	1.420157	2.084393	-10.128362
H	0.968916	3.154211	-11.468375
H	-1.468513	3.492056	-11.959149
H	-2.654895	2.621511	-10.971077
H	-2.093511	4.022318	-9.003149
H	-2.529577	5.048216	-10.362363
H	-0.617823	1.423161	-11.147543

Statistical Thermodynamic Analysis
Temperature 298.150 Kelvin. Pressure 1.00000 Atm.

SCF = -5464.03444317 l Predicted change in Energy=-9.612137D-09
Zero-point correction (ZPE) = -5463.65858517 0.375858

Internal Energy (U) = -5463.60853817
0.425905
Enthalpy (H) = -5463.60759417   0.426849
Gibbs Free Energy (G) = -5463.75434817
0.280095

Frequencies
9.4565 12.2281 15.8526
16.5729 20.3051 21.1110
26.2874 28.4088 31.8596
35.6336 37.0218 38.6864
41.5608 43.2213 45.1337
48.3784 50.6156 52.8029
59.2887 61.4240 68.1570
76.2509 81.8186 94.3395
106.5826 111.1257 114.6568
119.4645 132.3656 136.8454
178.3994 184.4937 186.1574
193.8645 197.7855 200.7851
201.3844 211.0063 219.4429
234.0097 242.7445 261.9370
265.7384 272.0880 275.6176
279.9276 285.1655 287.4407
291.7794 297.4497 306.8614
312.6502 314.3587 314.9185
320.9000 322.2928 333.8042
339.7933 348.3111 395.6911
398.9912 406.3429 408.8791
412.2275 421.6433 431.8172
449.6703 453.9145 486.8183
500.0579 500.5353 515.6430
521.1667 522.7538 537.4401
538.4907 549.8641 550.5936
561.6262 564.3514 566.2489
567.5174 579.7763 581.1825
586.1666 592.2407 619.9085
622.9065 623.4401 686.6611
716.7688 719.1969 720.3320
760.4112 762.3121 763.4652
782.9906 783.9693 811.0856
814.3829 818.4432 852.9474
854.1176 876.7037 879.4262
945.4054 978.4491 978.9854
991.1363 1011.1182 1016.4988
1022.4287 1022.9109 1044.0723
1049.7411 1060.2489 1066.7811
1074.0340 1080.6902 1087.0922
1091.3827 1103.3397 1111.6328
1114.7216 1155.6269 1173.9432
1187.5942 1192.0688 1194.4217
1199.8959 1200.9496 1207.0734
1209.1450 1212.2529 1228.8438
1233.9580 1239.5341 1255.8064
1267.6628 1270.6904 1273.0892
1276.3695 1289.8029 1294.2535
1300.7779 1304.1385 1311.6157
1319.9566 1328.5994 1343.4534
1350.8439 1353.3618 1369.8131
1370.4107 1389.7220 1396.1880
1433.4886 1482.2250 1490.3465
1495.5497 1498.9221 1509.4358
1511.6508 1514.7750 1528.1353
1635.1783 1640.0371 2990.4668
2993.6440 3029.3761 3047.0822
3060.2766 3090.5675 3093.0984
3095.5220 3095.5764 3105.4163
3200.8821 3216.1483 3225.3235
3235.2047 3246.5943 3475.6273

$Ca(NTf_2)_2(PhSO_2F)(Pip)(THF)$ 9b

Gaussian 16: ES64L-G16RevB.01 20-Dec-2017

B3LYP/6-31G(d,p) gfprint gfinput scf=(direct,tight,maxcycle=300,xqc) opt=(maxcycle=250) freq=noraman
AN.EDU\08-Apr-2021\0\\# B3LYP/6-31G(d,p) gfprint gfinput scf=(direct,t
ight,maxcycle=300,xqc) opt=(maxcycle=250) freq=noraman\\020d_ca_2ntf2_

Full point group C1 NOp 1
Stoichiometry C19H24CaF13N3O11S5
Framework group C1[X(C19H24CaF13N3O11S5)]

Num atoms: 76
Charge = 0 Multiplicity = 1

SCF = -5696.51833232 l Predicted change in Energy=-2.271682D-08

Optimization completed.
Maximum Force   0.000017   0.000450   YES
RMS   Force   0.000003   0.000300   YES

Maximum Displacement 0.001474 0.001800 YES
RMS Displacement 0.000325 0.001200 YES

Atom Coordinates (in Angstroms)

Type	X	Y	Z
C	-6.362953	6.618500	-3.045961
F	-6.005713	7.764813	-2.477284
F	-5.261310	5.950132	-3.429012
F	-7.040383	5.876438	-2.168657
S	-7.424064	6.931776	-4.553607
O	-8.532264	7.770700	-4.120683
O	-7.752294	5.549527	-5.010234
N	-6.439959	7.747984	-5.522751
S	-5.502157	7.099761	-6.656654
O	-5.728401	5.648045	-6.916013
O	-4.113552	7.534124	-6.544944
C	-6.182839	7.917369	-8.192553
F	-5.972761	9.227692	-8.154811
F	-5.571914	7.395469	-9.261546
F	-7.498045	7.678383	-8.287542
C	-4.661861	1.759527	-1.921180
F	-3.741249	1.511919	-2.862550
F	-4.360124	2.905931	-1.308248
F	-4.669090	0.768081	-1.036501
S	-6.339830	1.922958	-2.731281
O	-6.109954	3.029323	-3.703346
O	-7.289044	2.158479	-1.646711
N	-6.617794	0.497838	-3.404570
S	-6.230232	0.124855	-4.925632
O	-4.912122	-0.487057	-5.105980
O	-6.607758	1.168677	-5.925780
C	-7.499280	-1.219236	-5.199446
F	-8.729853	-0.723716	-5.087560
F	-7.324481	-1.685075	-6.440503
F	-7.326376	-2.205349	-4.326331
Ca	-6.651231	3.596266	-5.978116
C	-11.490779	5.535151	-3.726466
C	-10.602465	4.457925	-3.672806
C	-9.930444	4.084495	-2.505920
C	-10.172566	4.824625	-1.350932
C	-11.059284	5.903587	-1.377319
C	-11.714022	6.259654	-2.558715
H	-11.972136	5.805218	-4.658922
H	-9.227129	3.259648	-2.491354
H	-9.654924	4.558658	-0.435567
H	-11.234230	6.478079	-0.472940
H	-12.389005	7.108637	-2.575970
S	-10.312983	3.545944	-5.154055
O	-8.987359	2.922472	-5.151860
F	-11.321163	2.298242	-4.964186
O	-10.770804	4.256049	-6.336615
C	-1.887412	3.271905	-5.885020
C	-3.246293	3.893993	-5.548285
O	-4.234441	3.137763	-6.313472
C	-3.575371	2.072072	-7.044461
C	-2.263436	1.842967	-6.304142
H	-1.418396	3.804635	-6.719074
H	-1.201364	3.306641	-5.035002
H	-3.321618	4.946723	-5.829083
H	-3.495678	3.778154	-4.489024
H	-4.235628	1.204675	-7.040596
H	-3.403287	2.403113	-8.077948
H	-1.508095	1.362938	-6.931826
H	-2.440896	1.210425	-5.428943
C	-6.555137	2.390401	-10.487566
C	-7.025760	1.467696	-9.352922
C	-8.287524	2.009325	-8.676755
N	-8.065923	3.398846	-8.192757
C	-7.725454	4.297892	-9.326092
C	-6.429950	3.840631	-9.997242
H	-7.282149	2.351797	-11.310640
H	-5.601798	2.037231	-10.896297
H	-6.235907	1.364605	-8.599495
H	-7.228236	0.459516	-9.731444
H	-8.560997	1.387422	-7.820248
H	-9.125593	1.987417	-9.392443
H	-8.540300	4.297029	-10.068941
H	-7.628895	5.314156	-8.942443
H	-5.604318	3.929979	-9.279174
H	-6.196845	4.514695	-10.828835
H	-8.954420	3.724636	-7.808317

Statistical Thermodynamic Analysis
Temperature 298.150 Kelvin. Pressure 1.00000 Atm.

SCF = -5696.51833232 l Predicted change in Energy=-2.271682D-08
Zero-point correction (ZPE) = -5696.02366132 0.494671
Internal Energy (U) = -5695.96693832 0.551394
Enthalpy (H) = -5695.96599432 0.552338

Gibbs Free Energy (G) = -5696.12883632
0.389496

Frequencies
8.9378 10.4592 10.7229
16.1808 19.3350 23.3590
26.9962 28.8418 29.7545
31.9981 35.0148 36.2672
38.9977 41.5128 42.7900
44.1849 49.2355 51.0114
54.2087 57.7280 57.9953
60.1716 64.3802 70.6339
75.4342 77.9784 85.7598
96.8899 97.5775 101.8321
107.4040 110.6362 113.0268
118.2118 129.2714 133.2684
150.9921 172.4716 178.5844
187.1419 190.7759 196.9290
203.8031 206.0535 213.5833
218.6854 227.0354 233.4410
251.3745 270.9756 273.0866
274.7457 276.8542 277.5045
285.8218 287.2144 292.1928
294.2438 298.3508 314.3414
315.4711 317.5564 323.2106
328.5221 330.6980 340.7187
347.6307 395.0792 400.6285
403.6996 409.1340 425.2880
432.1434 440.9046 452.4541
453.2917 482.3050 492.0447
497.5978 508.7966 518.1193
521.2052 538.3087 538.7064
548.9027 550.3958 554.2047
560.8228 563.2058 566.8762
575.1797 582.0449 582.7070
588.2396 591.9526 622.7105
623.3066 638.0787 684.5539
687.8959 719.3976 719.9118
721.1446 760.6834 761.8426
763.9372 781.5922 786.4436
805.4139 815.0341 819.7858
851.8892 856.4270 859.9756
876.8577 880.4722 884.1481
891.4000 922.0049 932.4830
953.8389 976.6827 983.1006
991.2435 992.1963 1010.4856
1017.3444 1018.8505 1020.4115
1045.0602 1049.0763 1050.4260
1053.6867 1059.3997 1070.8039
1079.0501 1087.8649 1092.2681
1100.3065 1113.3411 1113.7762
1116.6623 1166.0284 1174.1359
1174.8124 1191.8925 1192.9358
1194.0020 1195.1827 1201.3225
1203.6703 1207.7367 1210.3811
1211.9048 1212.9199 1231.0049
1232.9185 1235.8743 1252.2193
1261.9396 1264.6548 1266.4276
1268.3866 1268.7009 1275.1247
1285.5956 1292.5079 1295.7125
1297.3664 1305.1999 1312.8039
1329.5000 1330.5626 1338.4523
1344.5134 1354.7408 1357.0981
1368.5483 1369.7890 1381.9476
1388.0042 1394.2710 1413.8353
1437.2279 1485.6423 1490.4174
1496.1930 1499.7779 1502.8383
1510.0245 1512.1644 1512.2594
1516.7482 1528.5721 1531.7431
1547.5894 1636.1564 1641.2281
2983.0107 2987.2312 3021.9803
3027.2604 3040.9332 3051.9564
3066.3883 3071.5949 3077.8884
3080.0872 3089.5162 3095.9227
3108.5498 3120.1552 3130.6918
3139.0461 3146.9624 3153.0944
3197.6428 3212.5825 3218.7955
3230.1908 3233.5014 3471.7517

$Ca(NTf_2)_2(PhSO_2F)(Pip)(THF)_2$ 9c

Gaussian 16: ES64L-G16RevB.01 20-Dec-2017

B3LYP/6-31G(d,p) gfprint gfinput scf=(direct,tight,maxcycle=300,xqc) opt=(maxcycle=250) freq=noraman
AN.EDU\23-Mar-2021\0\\# B3LYP/6-31G(d,p) gfprint gfinput scf=(direct,t ight,maxcycle=300,xqc) opt=(maxcycle=250) freq=noraman\\020d_ca_2ntf2_

Full point group C1 NOp 1
Stoichiometry C23H32CaF13N3O12S5
Framework group C1[X(C23H32CaF13N3O12S5)]

Num atoms: 89

Charge = 0 Multiplicity = 1

SCF = -5928.99232254 l Predicted change in Energy=-2.592159D-08

Optimization completed.
Maximum Force 0.000033 0.000450 YES
RMS Force 0.000004 0.000300 YES
Maximum Displacement 0.001651 0.001800 YES
RMS Displacement 0.000245 0.001200 YES

Atom Coordinates (in Angstroms)
Type X Y Z

Ca	-5.788766	-1.139273	0.096352
S	-4.712397	-4.457380	-1.043534
O	-4.628024	-4.377720	-2.508656
O	-5.754903	-3.597659	-0.411722
N	-3.294281	-4.445410	-0.260940
S	-2.524869	-3.054742	-0.038251
O	-1.707829	-3.090932	1.169712
O	-3.390171	-1.860762	-0.265642
C	-1.311143	-2.927055	-1.458357
F	-0.486825	-3.970292	-1.463764
F	-1.974817	-2.877637	-2.620650
F	-0.601439	-1.800553	-1.317876
C	-5.232387	-6.198647	-0.611055
F	-5.368315	-6.329930	0.705310
F	-6.412930	-6.415717	-1.200780
F	-4.342691	-7.074872	-1.065559
S	-8.350073	0.770210	2.131460
O	-7.004541	0.163798	1.936945
O	-9.296000	0.049104	2.978693
N	-9.072503	1.310422	0.804574
S	-8.489265	1.276749	-0.692342
O	-9.493143	0.785633	-1.632949
O	-7.107754	0.738356	-0.831151
C	-8.274012	3.087228	-1.108527
F	-7.842599	3.184335	-2.369525
F	-9.427989	3.734770	-0.982178
F	-7.363391	3.641247	-0.296947
C	-7.964370	2.336988	3.079072
F	-7.362095	2.002565	4.233229
F	-7.134843	3.120231	2.378142
F	-9.077784	3.009315	3.349060
C	-2.930119	2.665373	0.256277
C	-4.369524	2.149588	0.456488
C	-3.063112	0.630841	1.568955
C	-2.044641	1.574651	0.920247
H	-2.692010	2.767609	-0.805592
H	-2.798578	3.647344	0.718581
H	-5.016079	2.254529	-0.413413
H	-4.855260	2.633956	1.312372
H	-3.321834	0.957593	2.586872
H	-2.761720	-0.414348	1.586611
H	-1.465350	1.033464	0.167577
H	-1.343814	1.983691	1.652610
O	-4.232783	0.741508	0.733353
C	-10.327468	-2.701854	-0.422675
C	-10.125815	-2.980843	1.092598
C	-8.606801	-2.870202	1.294463
O	-8.140241	-2.058294	0.190483
C	-8.902332	-2.502226	-0.955286
H	-10.834339	-3.520919	-0.939761
H	-10.912549	-1.791946	-0.573144
H	-10.491992	-3.966994	1.390310
H	-10.641001	-2.230624	1.695947
H	-8.322968	-2.365720	2.216409
H	-8.111901	-3.848569	1.241775
H	-8.465499	-3.439513	-1.324741
H	-8.827223	-1.729886	-1.720499
C	-4.468594	0.131191	-2.862880
C	-3.895210	0.084584	-4.285224
C	-5.002975	-0.195763	-5.311574
C	-5.794137	-1.453995	-4.924300
C	-6.302785	-1.368804	-3.478807
H	-5.158026	0.978945	-2.763688
H	-3.671698	0.264240	-2.124259
H	-3.133426	-0.705916	-4.334320
H	-3.385685	1.029809	-4.509878
H	-4.578442	-0.302723	-6.316249
H	-5.685493	0.665122	-5.349017
H	-6.642205	-1.603230	-5.603358
H	-5.152091	-2.340029	-5.019176
H	-7.048780	-0.570706	-3.382502
H	-6.781028	-2.309031	-3.190264
N	-5.226544	-1.089650	-2.497129
H	-4.576293	-1.875408	-2.543098
C	-3.621008	-1.347424	6.998619
C	-3.894768	-2.226511	5.951901
C	-4.679560	-1.766529	4.892745
C	-5.200010	-0.470074	4.844396
C	-4.920321	0.388075	5.905788

C -4.134166 -0.048614 6.975683
H -3.007590 -1.678502 7.830105
H -3.506633 -3.238518 5.944622
H -5.802844 -0.144284 4.003911
H -5.322279 1.395474 5.892988
H -3.919960 0.628178 7.797154
S -5.036017 -2.882396 3.566132
O -4.284784 -4.110816 3.681847
F -6.579912 -3.250936 3.892000
O -5.104036 -2.143136 2.307772

Statistical Thermodynamic Analysis
Temperature 298.150 Kelvin. Pressure 1.00000 Atm.

SCF = -5928.99232254 | Predicted change in Energy=-2.592159D-08
Zero-point correction (ZPE) = -5928.37881854 0.613504
Internal Energy (U) = -5928.31524554 0.677077
Enthalpy (H) = -5928.31430154 0.678021
Gibbs Free Energy (G) = -5928.49438254 0.49794

Frequencies
3.3485 8.5942 14.5187
16.2189 20.3846 23.3249
24.1300 29.7544 30.8108
31.7535 35.8296 36.6575
38.1427 40.4570 41.9678
43.6606 46.1848 46.8006
48.9282 50.8508 53.7432
55.3256 57.7782 58.8261
61.5212 66.0594 68.4206
71.5039 73.9102 75.5863
80.8514 82.3665 85.4413
87.1356 94.3503 99.4182
109.0665 115.3058 119.4274
121.0793 121.7728 123.2191
132.3152 142.8597 166.2943
172.2701 184.8559 186.5912
196.5033 199.6298 203.8550
207.7115 218.2801 224.8232
230.3312 240.0090 245.3870
273.7007 274.8480 276.9522
277.9513 282.9134 288.1090
290.6385 293.2125 310.2935
312.0596 313.8174 317.3648
320.9865 322.6041 327.0303
332.4456 339.9443 348.9064
395.4020 395.8499 402.8739
413.1647 418.1239 433.2095
449.7385 454.1617 461.8164
470.5252 489.1800 493.1712
503.8080 519.1062 520.9373
537.8597 538.2259 548.1050
549.6531 553.1915 559.1481
561.2112 561.4715 569.1711
578.4560 584.8011 592.4622
623.9138 631.7304 634.7557
648.2740 651.8658 661.8296
666.2442 693.3688 716.1362
718.5528 726.6467 759.1809
762.4532 766.5018 778.8082
787.5184 795.2891 809.0726
814.5836 816.9195 817.7641
859.6366 869.0408 870.1642
871.2928 877.7837 885.7980
911.3753 913.0142 919.8732
931.7792 949.3561 956.1711
957.2936 958.3118 967.3290
973.0132 997.5813 1000.0359
1007.6836 1011.7835 1018.0238
1021.6377 1029.7503 1047.0282
1058.1827 1058.6943 1060.9159
1063.4401 1067.7219 1071.8736
1072.5776 1088.9021 1094.0156
1095.9522 1099.9106 1103.1588
1114.5359 1132.0785 1152.3918
1154.9559 1180.2773 1192.7243
1199.6057 1200.0027 1210.1857
1210.4540 1212.7601 1215.2551
1217.4365 1222.3893 1224.8956
1226.9059 1228.6039 1238.4426
1239.3721 1242.1606 1251.0910
1255.0525 1258.6341 1261.3599
1263.8888 1265.0758 1269.2072
1282.0907 1282.5588 1283.9463
1285.8849 1298.2149 1299.8459
1303.7351 1307.7776 1323.3043
1326.1932 1332.5963 1334.3396
1346.1844 1350.1975 1359.7323
1363.8195 1375.1709 1376.8495
1382.9216 1383.1125 1391.0353
1396.9553 1408.2342 1410.9694
1412.7417 1472.8946 1490.3544
1499.3095 1499.7487 1501.3178

1501.6527 1503.1797 1505.7609
1517.5374 1517.8275 1518.4964
1519.1468 1520.2246 1523.5009
1538.5996 1540.4924 1638.1767
1641.1375 3012.7847 3022.2216
3026.2061 3032.5775 3033.5850
3040.2122 3043.5276 3052.0587
3052.7302 3071.7733 3074.4888
3075.7956 3079.5802 3082.3016
3083.9647 3088.2211 3099.2275
3111.2286 3114.0826 3123.7540
3133.4039 3141.2154 3158.1708
3167.3987 3173.2943 3183.0304
3196.2724 3210.7188 3216.9564
3225.4334 3231.4768 3485.5521

$Ca(NTf_2)_2$(DABCO)(Pip) 10a

Gaussian 16: ES64L-G16RevB.01 20-Dec-2017

B3LYP/6-31G(d,p) gfprint gfinput scf=(direct,tight,maxcycle=300,xqc) opt=(maxcycle=250) freq=noraman N.EDU\07-Apr-2021\0\\# B3LYP/6-31G(d,p) gfprint gfinput scf=(direct,ti ght,maxcycle=300,xqc) opt=(maxcycle=250) freq=noraman\\020c_ca_2ntf2_d

----

Full point group C1 NOp 1
Stoichiometry C15H23CaF12N5O8S4
Framework group C1[X(C15H23CaF12N5O8S4)]

Num atoms: 68
Charge = 0 Multiplicity = 1

SCF = -4929.31487584 l Predicted change in Energy=-6.557623D-09

Optimization completed.
Maximum Force 0.000013 0.000450 YES
RMS Force 0.000002 0.000300 YES
Maximum Displacement 0.001458 0.001800 YES
RMS Displacement 0.000267 0.001200 YES

Atom Type	Coordinates (in Angstroms) X	Y	Z
C	-0.340156	6.466301	-3.550941
F	-0.684974	7.090823	-4.672999
F	0.447083	7.244447	-2.818618
F	-1.435589	6.152587	-2.859085
S	0.538566	4.864695	-3.949741
O	-0.424310	4.127186	-4.831329
O	0.852416	4.201137	-2.677235
N	1.792060	5.480920	-4.758310
S	2.785274	4.546588	-5.611020
O	3.370672	5.298820	-6.711502
O	2.205412	3.201151	-5.921073
C	4.190646	4.115694	-4.449778
F	4.750682	5.225567	-3.982386
F	3.715777	3.388449	-3.432082
F	5.094598	3.399728	-5.117934
C	-4.112127	-0.261858	-7.145038
F	-4.555551	-1.441733	-7.560632
F	-4.896703	0.197250	-6.169654
F	-4.112493	0.605753	-8.157529
S	-2.387896	-0.431419	-6.441809
O	-2.459740	-1.359867	-5.313967
O	-2.007923	0.979784	-6.105521
N	-1.658416	-0.986026	-7.773282
S	-0.069050	-0.947933	-7.989020
O	0.621171	0.080992	-7.147987
O	0.243599	-0.970221	-9.411418
C	0.584347	-2.549013	-7.270374
F	0.335313	-2.575875	-5.956100
F	-0.005477	-3.584157	-7.857500
F	1.900805	-2.605746	-7.470942
Ca	0.079741	2.117995	-6.050486
C	1.887501	0.202821	-3.823946
C	2.373503	-0.271148	-2.448777
C	1.217809	-0.864762	-1.629981
C	0.044836	0.123609	-1.553223
C	-0.381864	0.582283	-2.952859
N	0.743331	1.150419	-3.746542
H	1.561299	-0.654440	-4.424503
H	2.696483	0.697619	-4.371503
H	2.809088	0.582427	-1.910803
H	3.177497	-1.005171	-2.579072
H	1.559797	-1.133684	-0.624422
H	0.877186	-1.794924	-2.105606
H	-0.812647	-0.329425	-1.042259

H 0.338917 1.001955 -0.962065
H -0.797718 -0.259741 -3.517410
H -1.164817 1.344834 -2.888725
H 1.065781 1.979745 -3.240974
N -0.661092 4.427396 -10.658928
C 0.607634 3.682346 -10.647599
C 0.818803 2.978234 -9.270828
C -1.585523 2.809430 -9.004997
C -1.766529 3.495423 -10.394924
C -0.629747 5.442907 -9.596870
C -0.411809 4.772699 -8.204572
H 0.584794 2.950742 -11.461613
H 1.420159 4.385163 -10.858205
H 0.875608 1.891249 -9.379432
H 1.735954 3.317263 -8.779350
H -1.526482 1.722040 -9.102974
H -2.416891 3.033527 -8.329164
H -1.788890 2.751300 -11.197540
H -2.707032 4.054777 -10.438535
H 0.174651 6.151442 -9.819094
H -1.573525 5.997753 -9.623892
H 0.516616 5.117427 -7.740786
H -1.229923 4.993915 -7.511849
N -0.326614 3.288928 -8.358986

Statistical Thermodynamic Analysis
Temperature 298.150 Kelvin. Pressure 1.00000 Atm.

SCF = -4929.31487584 | Predicted change in Energy=-6.557623D-09
Zero-point correction (ZPE) = -4928.85797184 0.456904
Internal Energy (U) = -4928.80976584 0.50511
Enthalpy (H) = -4928.80882184 0.506054
Gibbs Free Energy (G) = -4928.95155684 0.363319

Frequencies
9.1649 10.9247 14.4889
17.9750 22.3192 26.3896
31.3714 33.2630 33.5710
34.8118 37.2315 38.5970
38.9373 40.2413 47.5232
54.8204 60.0412 66.0857
69.4138 74.4090 79.0569
83.7440 86.3807 90.4280
93.2362 96.3095 105.4231
106.4920 110.9458 159.1266
162.7450 170.9632 177.1520
180.0732 208.3007 215.2766
220.6956 229.4664 235.0635
240.9914 256.6347 274.2057
281.0831 283.9925 286.1958
291.2564 295.4625 303.0957
316.1580 317.7080 326.4988
327.9399 330.4356 333.4138
357.8848 363.6210 398.0538
406.5233 412.3721 423.8636
425.4268 441.9815 450.5739
458.4671 460.1254 490.7113
496.0095 502.1456 506.4393
537.9148 538.0529 550.3379
550.6662 554.1268 554.3388
561.6849 564.9540 585.6761
586.6056 587.6083 589.4709
592.1628 618.5364 639.9028
647.1454 718.1083 721.4340
765.1976 765.6694 779.3182
786.9708 789.6035 803.8817
812.7541 815.0504 816.6258
838.6953 842.0672 859.8160
876.7491 885.7606 906.3266
909.4034 955.3496 978.2928
1004.2782 1004.3663 1009.8235
1017.0419 1021.3393 1026.4713
1030.3182 1031.3262 1046.0355
1057.0163 1061.5417 1076.7168
1077.4484 1078.1184 1084.1827
1094.6779 1099.3623 1137.2743
1197.3283 1200.1410 1202.2511
1204.7356 1207.2483 1208.6528
1211.9857 1213.9204 1216.0992
1236.6253 1241.1902 1253.1143
1254.4670 1266.5353 1270.4459
1270.5959 1272.2435 1277.1671
1284.3740 1287.8031 1299.2282
1300.2250 1306.3532 1312.3528
1336.3040 1339.4846 1347.4077
1348.0656 1353.4019 1356.5457
1358.7492 1362.6575 1363.6097
1364.1680 1383.3265 1387.8948
1393.7203 1400.4676 1407.2292
1418.8337 1483.6705 1493.3854
1497.4941 1499.4280 1501.0553
1503.8225 1504.8858 1505.1562
1506.7546 1513.4381 1518.9822

1524.6847 3025.3219 3026.6637
3031.6642 3051.1603 3052.8832
3053.2093 3056.3856 3058.0140
3063.3295 3065.3769 3068.8814
3077.5293 3079.8924 3084.5116
3094.5483 3096.8295 3099.5284
3102.5235 3107.0873 3119.2114
3123.6287 3125.9480 3448.2924

Ca(NTf$_2$)$_2$(DABCO)(Pip)(THF) 10b

Gaussian 16: ES64L-G16RevB.01 20-Dec-2017

B3LYP/6-31G(d,p) gfprint gfinput scf=(direct,tight,maxcycle=300,xqc) opt=(maxcycle=250) freq=noraman N.EDU\07-Apr-2021\0\\# B3LYP/6-31G(d,p) gfprint gfinput scf=(direct,ti ght,maxcycle=300,xqc) opt=(maxcycle=250) freq=noraman\\020c_ca_2ntf2_d
----
Full point group C1 NOp 1
Stoichiometry C19H31CaF12N5O9S4
Framework group C1[X(C19H31CaF12N5O9S4)]

Num atoms: 81
Charge = 0 Multiplicity = 1

SCF = -5161.79297919 l Predicted change in Energy=-3.928565D-08

Optimization completed.
Maximum Force 0.000066 0.000450 YES
RMS Force 0.000010 0.000300 YES
Maximum Displacement 0.000567 0.001800 YES
RMS Displacement 0.000106 0.001200 YES

Atom Coordinates (in Angstroms)
Type X Y Z

C 2.761937 -1.644262 -5.850203
F 3.223637 -1.021421 -6.930446
F 3.104904 -2.927519 -5.883558
F 3.264107 -1.072728 -4.755345
S 0.902063 -1.480188 -5.757828
O 0.648492 -0.012466 -5.805965
O 0.465492 -2.162102 -4.530149
N 0.533937 -2.239438 -7.143101
S -0.924065 -2.073025 -7.786314
O -0.875933 -2.197060 -9.237816
O -1.696404 -0.927630 -7.214592
C -1.911799 -3.542510 -7.178407
F -1.972112 -3.524101 -5.841843
F -3.155809 -3.453634 -7.667540
F -1.355364 -4.677505 -7.584630
C 0.964555 3.994601 -2.985880
F 2.132152 3.354766 -3.036709
F 1.126281 5.176093 -2.397147
F 0.094194 3.263190 -2.275720
S 0.324892 4.225576 -4.728519
O 0.132167 2.832530 -5.213641
O 1.322191 5.039589 -5.410212
N -1.041642 5.062771 -4.553293
S -2.527191 4.468918 -4.656872
O -2.614099 2.985871 -4.812024
O -3.358399 5.262648 -5.559922
C -3.229256 4.753115 -2.947358
F -3.201522 6.046935 -2.647070
F -4.493002 4.317226 -2.933300
F -2.523180 4.068494 -2.040959
Ca -1.495636 1.209419 -6.046007
C -4.531952 0.056743 -7.644781
O -3.910542 1.141103 -6.894316
C -4.903695 2.177062 -6.626414
C -6.086104 1.854965 -7.534940
C -6.034480 0.322741 -7.591274
H -4.152777 0.086729 -8.672456
H -4.229998 -0.891088 -7.199590
H -5.180520 2.125101 -5.567772
H -4.449090 3.150686 -6.811587
H -7.027053 2.246628 -7.140388
H -5.933554 2.280818 -8.533035
H -6.558661 -0.101585 -8.451422
H -6.469771 -0.107331 -6.682318
C -3.388173 -0.816947 -3.910322
C -3.643950 -1.728979 -2.703176
C -3.344974 -0.998718 -1.385912
C -1.930799 -0.400953 -1.410433
C -1.727846 0.480894 -2.648554
N -2.029207 -0.221793 -3.923414
H -4.109507 0.010660 -3.914272

H -3.518953 -1.371509 -4.844515
H -3.002104 -2.616659 -2.786982
H -4.680827 -2.085744 -2.725933
H -3.461005 -1.679827 -0.535585
H -4.077700 -0.191559 -1.244018
H -1.742054 0.189771 -0.506289
H -1.188027 -1.210301 -1.421271
H -2.376170 1.362593 -2.595973
H -0.698057 0.844361 -2.701428
H -1.358610 -0.991119 -4.000489
N -0.060478 3.299689 -10.642307
C 1.084396 2.673807 -9.961002
C 0.636995 2.026918 -8.613120
C -1.599181 1.715400 -9.491461
C -1.101578 2.284389 -10.856204
C -0.604285 4.362402 -9.783920
C -1.004291 3.787086 -8.389300
H 1.517535 1.924112 -10.631346
H 1.844041 3.444245 -9.793796
H 0.770634 0.941140 -8.625680
H 1.199819 2.420744 -7.762595
H -1.491960 0.628364 -9.446144
H -2.652187 1.954781 -9.310889
H -0.682516 1.489392 -11.481650
H -1.923436 2.744135 -11.415910
H 0.152162 5.146494 -9.677946
H -1.467193 4.805223 -10.292497
H -0.382291 4.215621 -7.600516
H -2.050340 4.003248 -8.146750
N -0.811471 2.306309 -8.370039

Statistical Thermodynamic Analysis
Temperature 298.150 Kelvin. Pressure
1.00000 Atm.

SCF = -5161.79297919 | Predicted change in
Energy=-3.928565D-08
Zero-point correction (ZPE) = -5161.21741319
0.575566
Internal Energy (U) = -5161.16257519
0.630404
Enthalpy (H) = -5161.16163119 0.631348
Gibbs Free Energy (G) = -5161.32030619
0.472673

Frequencies
5.5327 9.0777 15.0767
16.9682 18.4277 25.2659
27.2677 28.8531 30.7043
32.5235 35.1717 37.6807
40.7413 42.5753 44.6383
50.2777 52.6265 54.7759
57.4260 62.5187 68.3374
72.6931 74.3563 77.8195
79.0401 82.3322 85.6662
90.1883 92.4609 94.2806
103.9088 111.5346 114.7246
125.8707 130.5325 156.5037
163.5628 179.0180 182.5801
184.6389 191.3908 203.6858
215.6735 218.6054 227.2771
235.4800 239.1684 250.8216
265.9548 274.5217 277.6034
278.3877 282.9208 285.6862
290.2386 293.7441 313.6004
316.7918 321.5463 327.2864
331.6018 332.5543 333.8214
351.1634 395.1398 397.6663
409.1835 410.7094 425.0110
425.9145 445.8877 455.9321
459.7036 490.3307 496.3527
504.7118 521.7338 538.9745
539.5728 549.0181 549.5955
553.8881 560.7376 562.1867
563.4004 577.8002 578.2323
586.1011 586.3616 587.9298
591.0418 616.0038 634.3595
651.0209 673.6981 718.8513
726.2324 762.2538 763.5026
777.5852 782.5639 789.1323
804.7153 812.8061 817.0569
817.9314 839.7929 843.3484
849.6772 859.5824 877.3194
878.0934 883.3861 885.6182
906.4214 909.9879 918.8496
936.4930 957.1191 973.7253
978.8210 1002.8363 1003.8182
1015.7403 1017.4673 1023.3207
1027.6533 1030.7599 1031.6805
1048.3361 1049.4978 1059.9775
1063.0974 1074.8524 1076.3738
1077.9363 1085.7206 1092.5083
1102.8531 1104.4592 1136.6639
1170.0223 1195.1855 1199.1634
1199.9595 1204.5596 1206.6296
1207.3445 1210.6025 1210.9898
1213.6270 1215.0129 1217.1925
1226.8911 1231.2189 1247.0204

1250.9080 1262.2845 1264.8543
1266.8071 1268.8021 1269.0904
1272.0456 1274.1156 1282.5903
1283.8834 1299.4396 1300.8259
1308.7778 1311.5397 1330.1624
1332.2378 1336.1006 1344.0699
1347.2337 1350.1683 1353.4198
1355.9285 1357.2723 1360.7466
1360.8612 1362.8998 1381.4334
1382.3614 1385.0800 1392.3010
1398.0453 1403.6914 1412.9604
1413.8792 1486.4663 1496.9695
1497.1480 1498.7608 1499.2894
1501.5735 1503.8290 1507.3238
1509.7501 1509.9280 1510.9887
1513.2188 1517.3369 1524.6874
1533.4241 1545.0035 3021.8382
3028.3945 3032.0125 3038.0793
3048.0528 3051.8419 3054.4671
3055.5660 3058.3690 3060.6905
3062.0857 3064.4953 3064.7303
3066.7412 3071.0054 3075.3311
3079.3401 3084.5355 3093.0873
3097.3328 3099.3737 3101.0301
3119.6489 3121.1632 3122.5783
3126.6845 3127.2936 3132.9316
3148.2344 3151.9294 3445.1996

Ca(NTf$_2$)$_2$(DABCO)(Pip)(THF)$_2$ 10c

Gaussian 16: ES64L-G16RevB.01 20-Dec-2017

B3LYP/6-31G(d,p) gfprint gfinput scf=(direct,tight,maxcycle=300,xqc) opt=(maxcycle=250) freq=noraman AN.EDU\22-Mar-2021\0\\# B3LYP/6-31G(d,p) gfprint gfinput scf=(direct,t ight,maxcycle=300,xqc) opt=(maxcycle=250) freq=noraman\\020c_ca_2ntf2_

Full point group C1 NOp 1
Stoichiometry C23H39CaF12N5O10S4
Framework group C1[X(C23H39CaF12N5O10S4)]

Num atoms: 94
Charge = 0 Multiplicity = 1

SCF = -5394.26684669 l Predicted change in Energy=-2.596658D-08

Optimization completed.
Maximum Force 0.000412 0.000450 YES
RMS Force 0.000069 0.000300 YES
Maximum Displacement 0.000781 0.001800 YES
RMS Displacement 0.000088 0.001200 YES

Atom Coordinates (in Angstroms)
Type X Y Z

Ca -2.741575 2.441130 1.027245
S -3.180395 4.133425 4.402385
O -3.133545 3.996769 2.921943
O -2.353752 5.162875 5.021712
N -3.021525 2.745970 5.198771
S -3.277040 1.313679 4.505845
O -2.731851 1.206897 3.118678
O -4.602351 0.731228 4.720530
C -2.076166 0.296334 5.511088
F -2.410375 0.312252 6.795936
F -2.129526 -0.959615 5.050621
F -0.833413 0.759970 5.362887
C -4.953919 4.643635 4.714346
F -5.221504 5.749138 4.008534
F -5.775924 3.664700 4.311314
F -5.150106 4.883169 6.005659
S -3.254351 3.352395 -2.515814
O -4.578390 3.089699 -3.072788
O -2.772531 2.444543 -1.430563
N -3.082725 4.909379 -2.183877
S -2.049250 5.420084 -1.040832
O -0.675505 5.659215 -1.488378
O -2.208777 4.702775 0.254204
C -2.810795 7.099086 -0.731440
F -2.807108 7.822992 -1.845537
F -4.061089 6.969608 -0.283849
F -2.072110 7.706321 0.200252
C -2.036845 2.998764 -3.893123
F -2.302715 3.763179 -4.947629
F -0.794642 3.241131 -3.464680
F -2.138230 1.710942 -4.238539
C -4.459524 -1.732382 -0.755017

C -3.500960 -0.555176 -0.881410
C -3.786479 -1.009065 1.433837
C -4.097897 -2.278895 0.633005
H -5.498493 -1.385545 -0.779904
H -4.327345 -2.464065 -1.556522
H -3.827600 0.230219 -1.561316
H -2.498808 -0.888212 -1.182837
H -2.954790 -1.131477 2.129269
H -4.648763 -0.643400 1.997890
H -4.902536 -2.860847 1.090020
H -3.214404 -2.922420 0.569949
O -3.430991 0.016346 0.451981
C -7.172650 4.054297 1.154313
C -7.479152 2.627214 0.620698
C -6.128290 2.133153 0.071312
O -5.130732 2.963108 0.708347
C -5.708764 4.289663 0.764088
H -7.827959 4.814812 0.722515
H -7.282731 4.089771 2.239910
H -8.240056 2.630699 -0.163875
H -7.835799 1.981511 1.427021
H -5.893468 1.097783 0.321687
H -6.057160 2.269527 -1.014674
H -5.620249 4.754595 -0.225390
H -5.135469 4.867668 1.487272
N 1.026630 4.668458 2.639635
C 1.120322 4.415187 4.097237
C 1.846576 5.605976 4.801688
C 0.971576 7.107978 3.153324
C 0.264568 5.923328 2.420328
C 2.392965 4.848635 2.099204
C 3.095063 6.053034 2.807506
H 0.106535 4.287629 4.486484
H 1.659120 3.471722 4.243291
H 1.204823 6.061625 5.562962
H 2.765986 5.276891 5.299427
H 0.318821 7.545723 3.915903
H 1.242490 7.906374 2.453336
H -0.751985 5.770022 2.790023
H 0.206055 6.092058 1.341178
H 2.946554 3.914987 2.253899
H 2.310054 5.009582 1.018914
H 4.018516 5.737842 3.306540
H 3.362030 6.835355 2.088376
N 2.202757 6.642533 3.818239
C 0.576404 2.322660 -0.203603
C 0.423656 1.075102 1.881021
C 0.244412 -0.244863 1.126557
C 0.908492 -0.181840 -0.257003
C 0.417413 1.047696 -1.034796
H 1.650334 2.507333 -0.033264
H 0.183187 3.189353 -0.739430
H -0.083481 1.038164 2.847739
H 1.497893 1.234492 2.075705
H 0.662263 -1.064832 1.722154
H -0.828845 -0.444240 1.010874
H 1.998260 -0.118800 -0.130462
H 0.713015 -1.101104 -0.821683
H -0.638159 0.923938 -1.304927
H 0.969984 1.160367 -1.974361
N -0.129500 2.209725 1.100741
H 0.110814 3.068358 1.626174

Statistical Thermodynamic Analysis
Temperature 298.150 Kelvin. Pressure 1.00000 Atm.

SCF = -5394.26684669 l Predicted change in Energy=-2.596658D-08
Zero-point correction (ZPE) = -5393.57247869 0.694368
Internal Energy (U) = -5393.51104469 0.755802
Enthalpy (H) = -5393.51010069 0.756746
Gibbs Free Energy (G) = -5393.68464069 0.582206

Frequencies
7.9259 12.1382 15.3374
15.6385 18.6233 23.7125
25.8294 28.4269 29.9938
30.9823 33.6311 34.9221
38.4087 38.4835 39.7912
41.8383 45.5001 47.6518
49.2159 52.2714 53.2606
55.3517 58.9013 61.9849
63.9084 67.2814 70.2291
75.1725 78.8508 81.5561
85.9912 89.2588 91.6116
93.3584 98.6789 104.4513
106.7376 110.4338 115.1230
120.7237 130.6139 142.5942
151.7799 169.1460 178.2094
181.6360 184.3306 188.9495
204.8870 216.6741 221.3523
232.2923 233.5800 248.2516
267.8627 271.6177 276.6954
277.5814 284.3717 285.7460

293.8621 295.1881 298.3119
302.1002 315.1310 318.0058
323.8643 327.3985 334.8336
337.7923 345.9066 348.9351
396.7591 399.8909 426.4244
427.7846 428.6335 430.8305
438.4998 454.2233 455.7453
489.7457 494.1633 507.7411
510.8479 538.4749 539.3243
549.4310 549.9860 553.2390
554.7261 560.5976 562.6578
582.4299 585.3167 587.2344
589.1093 589.2967 592.1345
613.4834 642.2885 644.4860
645.3375 667.6510 678.6703
722.5598 723.0279 761.9426
763.6326 769.7379 783.8758
785.9316 807.5761 811.6342
815.3338 816.2048 821.4893
838.3599 839.4894 840.4891
863.9575 870.4047 878.0877
879.2391 881.9694 884.0694
900.9480 902.7004 909.8917
920.7260 924.2170 935.7496
949.7198 971.4933 972.7912
973.5658 979.2289 997.7321
1004.9509 1012.8448 1016.1173
1032.0135 1033.4710 1038.4641
1049.9445 1051.0030 1051.3034
1059.8750 1060.6836 1062.4737
1064.5287 1073.4086 1076.5861
1077.7422 1092.6676 1102.7371
1114.5979 1154.1333 1156.7323
1169.0146 1179.1629 1194.7914
1198.0047 1201.0946 1202.1491
1204.5480 1209.0511 1209.9595
1210.8887 1212.6928 1213.3729
1225.3812 1228.2997 1233.3891
1239.8645 1240.8606 1242.3433
1248.3050 1261.3036 1262.1119
1263.4846 1264.7954 1266.1658
1267.4609 1271.8582 1273.3065
1284.7548 1285.3241 1285.7443
1292.6835 1304.4291 1305.0802
1312.4412 1325.1197 1328.4666
1329.2234 1332.7248 1335.8290
1336.4155 1339.1621 1343.4499
1351.8194 1354.0296 1357.1898
1357.6881 1357.9943 1361.1759
1375.9556 1378.3776 1381.1555
1382.3015 1388.9835 1394.9420
1397.6235 1411.1690 1415.0104
1442.8763 1494.3881 1494.6372
1495.4158 1499.1754 1501.5483
1503.6351 1505.3790 1505.6924
1509.2583 1509.5685 1513.0464
1513.1946 1519.9116 1521.5270
1526.5604 1530.0365 1534.7520
1546.3240 1546.8217 1549.1134
2973.0917 2979.4228 3025.3180
3031.6932 3035.1553 3038.9337
3040.7192 3043.2099 3045.6673
3049.1002 3051.3052 3051.3965
3054.3166 3063.0625 3068.8058
3072.0279 3074.8531 3077.6435
3079.4462 3082.4505 3084.5228
3089.1004 3092.2056 3092.6070
3093.6247 3099.6562 3117.0791
3117.7010 3119.3596 3121.5409
3122.7634 3124.7995 3133.7278
3140.5282 3143.0891 3147.2731
3166.0340 3174.0347 3238.4585

$Ca(NTf_2)_2(PhSO_2F)(DABCO)(Pip)$ 11a

Gaussian 16: ES64L-G16RevB.01 20-Dec-2017

B3LYP/6-31G(d,p) gfprint gfinput scf=(direct,tight,maxcycle=300,xqc) opt=(maxcycle=250) freq=noraman
AN.EDU\07-Jan-2021\0\\# B3LYP/6-31G(d,p) gfprint gfinput scf=(direct,t ight,maxcycle=300,xqc) opt=(maxcycle=250) freq=noraman\\020a-Ca-2NTf2-
----
Full point group C1 NOp 1
Stoichiometry C21H28CaF13N5O10S5
Framework group C1[X(C21H28CaF13N5O10S5)]

Num atoms: 83
Charge = 0 Multiplicity = 1

SCF = -5809.39388321 l Predicted change in Energy=-2.789487D-08

Optimization completed.

Maximum Force 0.000027 0.000450 YES
RMS Force 0.000004 0.000300 YES
Maximum Displacement 0.001621 0.001800 YES
RMS Displacement 0.000302 0.001200 YES

Atom Type	Coordinates (in Angstroms) X	Y	Z
Ca	4.081103	1.236644	-5.161397
S	6.123446	4.020929	-6.259513
O	7.367610	3.412169	-6.737509
O	4.888930	3.198587	-6.420455
C	5.810718	5.498721	-7.360248
F	4.687615	6.115240	-6.979733
F	6.830551	6.347185	-7.291231
F	5.669746	5.065927	-8.614495
N	6.320046	4.695936	-4.813772
S	5.464117	4.319046	-3.502925
O	6.281268	4.464921	-2.308421
O	4.655456	3.076279	-3.642245
C	4.176407	5.672100	-3.394473
F	4.762025	6.852286	-3.223581
F	3.448888	5.703198	-4.525328
F	3.359085	5.423921	-2.368658
S	2.222297	-2.034268	-4.909849
O	3.225377	-1.080388	-5.474562
O	1.037996	-2.315763	-5.716547
C	3.164421	-3.651115	-4.849923
F	3.597297	-3.944514	-6.083651
F	4.213609	-3.538194	-4.032526
F	2.367004	-4.624035	-4.420086
N	1.808261	-1.720601	-3.398203
S	2.750543	-0.852047	-2.401676
O	3.274961	0.398195	-3.024816
O	3.689657	-1.629663	-1.597078
C	1.409686	-0.249925	-1.246520
F	0.817812	-1.278980	-0.651441
F	1.981383	0.532677	-0.332836
F	0.498584	0.458888	-1.924117
S	4.046285	-0.429913	-8.614318
O	4.854122	-1.596676	-8.333460
F	4.633881	0.255767	-9.952492
O	3.977929	0.683214	-7.663461
C	-0.114898	-1.557565	-9.995830
C	0.665444	-2.482555	-9.302806
C	1.957847	-2.148464	-8.897968
C	2.433597	-0.872539	-9.199797
C	1.666343	0.074591	-9.886899
C	0.383314	-0.283843	-10.290001
H	-1.118791	-1.826908	-10.308701
H	0.270861	-3.464546	-9.066109
H	2.573116	-2.851265	-8.350088
H	2.068533	1.055278	-10.115790
H	-0.226421	0.428820	-10.835709
C	6.988246	-0.641182	-2.973952
C	7.297699	0.657646	-3.719774
C	7.207820	-0.485929	-5.878595
C	6.887670	-1.819828	-5.207033
H	5.913009	-0.706090	-2.774394
H	7.481577	-0.613787	-1.996057
H	8.389175	0.778904	-3.809018
H	6.916606	1.519887	-3.165949
H	6.766834	-0.441496	-6.877463
H	8.299310	-0.385492	-5.993863
H	5.802289	-1.957996	-5.188423
H	7.299799	-2.633588	-5.814315
N	6.679596	0.654213	-5.078077
H	7.019062	1.493626	-5.552339
N	-0.708964	3.298799	-5.883841
C	-0.753631	1.843420	-6.089913
C	0.643223	1.207109	-5.812234
C	1.191792	3.001801	-4.294021
C	-0.245171	3.570083	-4.513403
C	0.250699	3.881347	-6.833578
C	1.660679	3.238283	-6.656142
H	-1.515003	1.420757	-5.426239
H	-1.075567	1.653761	-7.119643
H	0.604614	0.538013	-4.949263
H	1.000338	0.625809	-6.666340
H	1.226909	2.303844	-3.455082
H	1.910881	3.797032	-4.094674
H	-0.958724	3.117549	-3.816837
H	-0.268832	4.652637	-4.349229
H	-0.130611	3.725138	-7.848847
H	0.287529	4.962356	-6.661887
H	1.973515	2.688328	-7.549114
H	2.428262	3.987487	-6.451093
N	1.644664	2.273408	-5.517047
C	7.445227	-1.867620	-3.777256
H	8.543396	-1.887855	-3.817914
H	7.130120	-2.788927	-3.275605

Statistical Thermodynamic Analysis

Temperature 298.150 Kelvin. Pressure 1.00000 Atm.

SCF = -5809.39388321 l Predicted change in Energy=-2.789487D-08
Zero-point correction (ZPE) = -5808.83239521 0.561488
Internal Energy (U) = -5808.77388821 0.619995
Enthalpy (H) = -5808.77294321   0.62094
Gibbs Free Energy (G) = -5808.93826821 0.455615

Frequencies
7.2633 12.0473 16.0897
18.1645 21.7472 24.7650
29.3286 30.2990 31.7147
33.6742 36.7419 38.5690
39.9420 42.1616 45.0622
47.1476 47.5420 52.0759
53.3078 57.8419 60.7887
63.5866 66.2485 70.9458
74.3298 78.7807 82.0959
83.9840 90.4243 94.9920
97.1394 99.5575 105.6633
109.1781 118.4355 126.0600
129.9452 169.3056 175.1112
175.3249 179.2865 185.5696
190.4636 201.4402 206.4195
215.4399 219.8958 233.7970
244.8560 260.8297 273.8406
276.0057 278.2574 283.4928
284.0558 285.2592 289.6176
294.8532 304.5117 313.6427
316.6652 322.8954 326.3088
333.5087 336.7280 337.4657
341.4869 348.1899 392.8070
395.7388 408.7047 412.5947
418.3198 420.4017 425.0172
428.5704 433.8098 449.3194
452.5931 486.0606 491.4483
495.4209 509.8819 518.9494
520.2255 538.2159 539.2963
549.0720 549.4768 553.9303
559.5989 560.8433 564.0670
572.7563 577.9095 578.3955
586.6545 587.3386 590.7192
615.4735 624.1432 637.6220
641.8775 690.4800 717.1764
719.8244 720.9788 759.5241
761.2509 762.8521 778.5378
783.1942 784.4224 802.8016
805.3151 812.1778 815.4897
815.9884 838.7171 843.0938
853.9315 858.7690 873.3857
881.2290 907.5057 910.7636
942.8939 977.6747 980.1227
988.1529 1003.2715 1004.1309
1008.2227 1011.9916 1013.3600
1017.2205 1025.1449 1028.5078
1030.4249 1046.9986 1050.1033
1058.7969 1059.5358 1074.8511
1077.2281 1078.8333 1090.0516
1095.9115 1101.0815 1112.1607
1114.7443 1129.4610 1168.9450
1176.8270 1191.3995 1195.4779
1199.7963 1200.5208 1203.8394
1206.2700 1207.1568 1209.3299
1211.2776 1211.7335 1212.6904
1225.3733 1234.6211 1242.4983
1246.6542 1258.7525 1262.6951
1265.9571 1271.8771 1274.7306
1279.8852 1289.3340 1292.9084
1305.2611 1306.9869 1316.2073
1328.3500 1332.6609 1336.5566
1346.3047 1346.9479 1348.3212
1354.8897 1355.0994 1357.3139
1358.3085 1361.3611 1363.1625
1369.2481 1372.4716 1381.7981
1391.5305 1398.9389 1407.1079
1439.2721 1489.3875 1489.9874
1496.4213 1497.0528 1498.1839
1499.1448 1507.6083 1509.2439
1509.7367 1512.2678 1512.4262
1519.8374 1525.5857 1529.0889
1638.2770 1641.5900 2988.3283
2993.0974 3024.8837 3045.3353
3049.1580 3052.7331 3060.0351
3061.1100 3071.7693 3078.3836
3084.1719 3090.4364 3094.3119
3097.0470 3102.4999 3106.1268
3109.0961 3111.0688 3116.7774
3132.8009 3136.0009 3162.5614
3198.0349 3209.7471 3219.6330
3223.7596 3244.7554 3459.0539

$Ca(NTf_2)_2(PhSO_2F)$(DABCO)(Pip)(THF) 11b

Gaussian 16: ES64L-G16RevB.01 20-Dec-2017

B3LYP/6-31G(d,p) gfprint gfinput scf=(direct,tight,maxcycle=300,xqc) opt=(maxcycle=250) freq=noraman
AN.EDU\08-Jan-2021\0\\# B3LYP/6-31G(d,p) gfprint gfinput scf=(direct,t ight,maxcycle=300,xqc) opt=(maxcycle=250) freq=noraman\\020a-Ca-2NTf2-

----

Full point group C1 NOp 1
Stoichiometry C25H36CaF13N5O11S5
Framework group C1[X(C25H36CaF13N5O11S5)]

Num atoms: 96
Charge = 0 Multiplicity = 1

SCF = -6041.87340424 l Predicted change in Energy=-2.583419D-08

Optimization completed.
Maximum Force 0.000015 0.000450 YES
RMS Force 0.000002 0.000300 YES
Maximum Displacement 0.001444 0.001800 YES
RMS Displacement 0.000276 0.001200 YES

Atom Coordinates (in Angstroms)
Type X Y Z

Ca 5.038113 2.594757 -4.081204
S 7.461419 2.402707 -6.991479
O 8.266358 1.206899 -7.239353
O 6.156463 2.201269 -6.291065
C 6.935859 3.001208 -8.686122
F 6.175055 2.063993 -9.261737
F 6.223892 4.127709 -8.565148
F 8.004593 3.230827 -9.441945
N 8.357762 3.575805 -6.384318
S 7.702864 4.755996 -5.477860
O 6.822344 4.241377 -4.392470
O 7.216057 5.915330 -6.225723
C 9.257702 5.287095 -4.586220
F 9.754961 4.272621 -3.875898
F 10.166324 5.710741 -5.458423
F 8.927571 6.281322 -3.761940
S 3.876014 4.414778 -1.106073
O 2.798261 5.374124 -1.357091
O 4.809961 4.166906 -2.243123
C 4.971964 5.146967 0.218724
F 5.996227 4.331557 0.473401
F 5.438052 6.309343 -0.240850
F 4.275431 5.354049 1.331741
N 3.444219 3.066640 -0.319987
S 2.939181 1.760166 -1.100457
O 3.238125 0.560766 -0.322958
O 3.291659 1.749117 -2.550299
C 1.072059 1.893050 -1.119996
F 0.705209 3.029670 -1.721462
F 0.569813 0.857195 -1.796694
F 0.603769 1.880244 0.124762
S 3.482138 -0.720262 -5.581992
O 4.143395 0.471689 -5.064482
F 2.624692 -0.160499 -6.843581
O 2.550356 -1.487284 -4.783061
C 6.448624 -3.363316 -7.746830
C 6.766334 -2.025652 -7.500639
C 5.860905 -1.204486 -6.830034
C 4.647919 -1.756264 -6.416206
C 4.308340 -3.091763 -6.649763
C 5.226111 -3.894967 -7.323585
H 7.156846 -3.997801 -8.270890
H 7.712931 -1.605838 -7.823856
H 6.097003 -0.167470 -6.629558
H 3.358791 -3.486205 -6.306255
H 4.987030 -4.935671 -7.516954
C 7.861239 0.573123 -0.602594
C 6.884411 1.456965 -1.388927
C 8.066888 0.980972 -3.485131
C 9.074306 0.083941 -2.753567
H 7.442755 -0.440140 -0.525021
H 7.950398 0.955429 0.420991
H 7.222412 2.500027 -1.365204
H 5.896952 1.427301 -0.922253
H 7.914632 0.624702 -4.507836
H 8.467236 1.998613 -3.553937
H 8.727563 -0.957985 -2.795904
H 10.035940 0.118862 -3.279090
N 6.741042 1.064050 -2.817137
H 6.328995 0.124353 -2.841748
N 3.211849 3.999492 -5.384359

C	1.869807	3.843643	-4.748606
C	0.812102	4.696548	-5.515637
C	2.050023	4.477097	-7.548469
C	3.102948	3.589319	-6.814760
C	3.584619	5.447656	-5.333491
C	2.502280	6.300729	-6.064950
H	1.615602	2.779070	-4.754065
H	1.953223	4.161446	-3.707851
H	0.023421	4.063848	-5.937194
H	0.332116	5.416625	-4.844899
H	1.250674	3.865000	-7.980342
H	2.512810	5.038011	-8.367451
H	2.825622	2.530901	-6.838926
H	4.092060	3.683664	-7.267348
H	3.678443	5.728600	-4.281186
H	4.568339	5.562454	-5.796768
H	2.036908	7.016361	-5.379351
H	2.943442	6.872902	-6.887870
N	1.443917	5.438783	-6.616789
C	9.232232	0.512815	-1.288750
H	9.701405	1.505937	-1.250761
H	9.902494	-0.171001	-0.755458
C	4.111975	-3.559215	-2.277609
C	5.373973	-4.208901	-2.900875
C	6.289721	-3.001929	-3.225305
O	5.493032	-1.824604	-3.034005
C	4.572012	-2.132155	-1.971883
H	3.293339	-3.530301	-3.001232
H	3.759713	-4.084082	-1.385846
H	5.128294	-4.780795	-3.799432
H	5.866685	-4.891892	-2.203195
H	7.157148	-2.977897	-2.549107
H	6.655395	-2.988082	-4.255023
H	3.781464	-1.383128	-1.985776
H	5.089246	-2.077072	-1.001719

Statistical Thermodynamic Analysis
Temperature 298.150 Kelvin. Pressure 1.00000 Atm.

SCF = -6041.87340424 l Predicted change in Energy=-2.583419D-08
Zero-point correction (ZPE) = -6041.19375824 0.679646
Internal Energy (U) = -6041.12814224 0.745262
Enthalpy (H) = -6041.12719824 0.746206
Gibbs Free Energy (G) = -6041.31081324 0.562591

Frequencies
10.7166 14.2838 14.7190
20.5225 22.8006 25.3031
28.3227 29.3832 30.8858
32.9679 33.7950 35.0142
36.6710 37.8809 38.2237
39.3227 42.1066 43.0069
45.1746 47.0831 50.1959
51.8913 54.5566 54.8650
56.9395 57.4669 61.0490
64.7637 67.0108 72.3718
75.1734 78.1586 78.5804
79.8655 84.7208 86.9039
92.0040 93.6095 95.7246
96.7299 105.2202 107.3609
117.9573 120.7303 168.5133
169.9927 176.1788 179.2784
187.3982 192.1456 204.7409
213.9379 215.2890 222.6578
234.4482 240.2161 242.0606
274.2712 275.2201 276.6802
278.4819 280.6125 285.2880
289.9252 292.7173 295.6405
313.2150 316.6401 317.2582
323.5493 325.5671 333.0408
334.7744 340.0469 347.1909
350.0026 395.4284 398.7200
409.4011 415.4360 425.2077
426.8051 434.0336 436.6403
437.9195 457.0487 459.1367
475.4851 489.7129 493.8262
507.1885 508.3273 516.5848
538.4984 538.6417 549.9301
550.5154 553.6931 553.9498
560.0198 562.6131 571.2181
585.1113 587.3396 587.8651
589.5020 592.5408 616.5043
617.1907 624.0562 643.6556
645.7112 686.2946 694.5710
717.9556 722.7127 723.5166
763.1267 764.0261 767.9712
779.2904 786.2191 786.6191
788.7947 801.1438 804.8438
810.8922 815.5396 820.2850
842.6540 843.6553 861.1008
871.6140 872.1208 878.6116
887.9397 907.9782 910.1162
910.7546 926.8563 942.9342

957.2810 958.4149 966.0986
980.1657 1000.4592 1004.4051
1006.3332 1009.6367 1012.1815
1023.6285 1024.1972 1028.7963
1028.9000 1032.4081 1034.4468
1043.3009 1047.0192 1056.4904
1060.7074 1069.4004 1077.4479
1079.0314 1084.8730 1089.3215
1091.3276 1093.8406 1101.0733
1101.8047 1111.4741 1141.3558
1152.4932 1182.1401 1190.9398
1200.9296 1202.9105 1207.4529
1208.0157 1208.6574 1211.7356
1212.8991 1213.7754 1215.4578
1217.7230 1221.7967 1233.0423
1238.8782 1239.7442 1249.9058
1251.7935 1252.9689 1260.9545
1262.1690 1264.0273 1269.0569
1277.6502 1281.4158 1284.6847
1287.0541 1291.1107 1295.0664
1299.1539 1313.4835 1315.9189
1333.0049 1336.5833 1339.8160
1342.9796 1347.7499 1348.2278
1351.1597 1358.0678 1360.2732
1361.7449 1362.7176 1364.5358
1366.2224 1374.0101 1379.1758
1383.8059 1386.4129 1394.4509
1399.4440 1409.7714 1410.2174
1418.0933 1488.2448 1489.1147
1495.7082 1496.4317 1498.8881
1499.3411 1502.0751 1505.5480
1506.9282 1512.2719 1513.1097
1514.9748 1515.7909 1518.7368
1519.9197 1526.6143 1532.0511
1539.7206 1635.8443 1640.3442
3002.2787 3007.2671 3023.3727
3024.7619 3029.4064 3046.3246
3049.3198 3053.6764 3054.3747
3063.1882 3069.1876 3073.5867
3075.2150 3075.8511 3078.2257
3078.8705 3083.2206 3083.7064
3091.5796 3095.7696 3097.8043
3104.3725 3116.5254 3119.1129
3124.1308 3132.1126 3135.0289
3139.7743 3141.1726 3169.6520
3196.1854 3209.8365 3221.0240
3227.3343 3251.5242 3399.9298

$Ca(NTf2)_2(DABCO)_2(THF)$ 12

Gaussian 16: ES64L-G16RevB.01 20-Dec-2017

# B3LYP/6-31G(d,p) gfprint gfinput
scf=(direct,tight,maxcycle=300,xqc)
opt=(maxcycle=250) freq=noraman
N.EDU\07-Jan-2022\0\\# B3LYP/6-31G(d,p)
gfprint gfinput scf=(direct,ti
ght,maxcycle=300,xqc) opt=(maxcycle=250)
freq=noraman\\010d_1T_059.xyz

Full point group C1 NOp 1
Stoichiometry C20H32CaF12N6O9S4
Framework group
C1[X(C20H32CaF12N6O9S4)]

Num atoms: 84
Charge = 0 Multiplicity = 1

SCF = -5255.21388273 l Predicted change in Energy=-8.841015D-08

Optimization completed.
Maximum Force 0.000025 0.000450 YES
RMS Force 0.000005 0.000300 YES
Maximum Displacement 0.001065 0.001800 YES
RMS Displacement 0.000188 0.001200 YES

Atom Coordinates (in Angstroms)
Type X Y Z

Ca 1.522487 -0.283158 0.363231
S -2.043358 -1.261185 -0.185463
O -2.745181 -0.840407 -1.393275
O -0.557538 -1.402193 -0.272248
C -2.584418 -3.024677 0.131496
F -2.081938 -3.449162 1.295415
F -3.909600 -3.102346 0.162451
F -2.114260 -3.802209 -0.853291
N -2.542027 -0.420397 1.078980
S -1.606326 -0.192433 2.387188
O -0.200400 0.171264 2.053760

O	-1.787541	-1.172727	3.458367
C	-2.364547	1.412568	2.975000
F	-3.653186	1.239454	3.248918
F	-1.715651	1.782118	4.079529
F	-2.223978	2.355991	2.043130
S	3.361712	2.616281	1.875001
O	2.942829	3.983595	1.590555
O	2.333253	1.541431	1.800714
C	3.873792	2.587291	3.674510
F	4.382983	1.385770	3.972105
F	4.789320	3.523955	3.901612
F	2.801947	2.812410	4.434296
N	4.711849	2.270269	1.066375
S	5.049016	0.773765	0.579909
O	3.895848	0.078111	-0.072625
O	5.841018	-0.043572	1.501948
C	6.158661	1.196757	-0.861905
F	6.534677	0.043431	-1.428167
F	5.493922	1.922451	-1.763863
F	7.230963	1.861100	-0.450661
N	0.833014	1.624456	-1.366171
C	1.989276	2.482655	-1.761515
C	1.528999	3.565151	-2.786768
C	-0.612678	3.636794	-1.719872
C	-0.197242	2.502521	-0.731481
C	0.248610	1.029152	-2.604481
C	-0.238455	2.157483	-3.565355
H	2.388032	2.949620	-0.858703
H	2.769190	1.835461	-2.173500
H	1.767246	4.570086	-2.423697
H	2.031255	3.436928	-3.751808
H	-0.360836	4.622167	-1.314734
H	-1.692038	3.621003	-1.902741
H	0.229728	2.903932	0.191832
H	-1.053774	1.878949	-0.460975
H	1.024575	0.408172	-3.065148
H	-0.573994	0.372745	-2.312534
H	0.236485	2.072992	-4.549028
H	-1.321138	2.098574	-3.716783
N	0.078487	3.481069	-3.010049
N	2.235198	-2.206615	2.043044
C	1.146226	-3.217042	2.183395
C	1.574917	-4.331734	3.187980
C	2.893240	-2.741287	4.396854
C	2.495492	-1.616095	3.391276
C	3.473087	-2.905257	1.588920
C	3.874283	-4.007993	2.617412
H	0.248963	-2.699130	2.527687
H	0.937103	-3.627092	1.189783
H	0.870341	-4.394037	4.023625
H	1.598409	-5.314399	2.704301
H	2.184704	-2.792587	5.230026
H	3.886286	-2.556989	4.819776
H	1.588151	-1.092076	3.703157
H	3.290357	-0.873764	3.286246
H	3.266908	-3.326547	0.599030
H	4.260274	-2.156119	1.476644
H	3.902959	-4.997577	2.148009
H	4.869083	-3.811807	3.030652
N	2.913095	-4.052992	3.728986
C	1.235874	-2.789253	-2.238876
C	3.429158	-1.866901	-2.272092
C	3.243481	-2.752723	-3.504004
C	2.136573	-3.713750	-3.050516
H	0.658219	-3.288450	-1.461175
H	0.538927	-2.238823	-2.881361
H	3.723012	-0.842400	-2.505739
H	4.164548	-2.281601	-1.575684
H	2.903357	-2.159032	-4.359741
H	4.169759	-3.257069	-3.790608
H	2.550388	-4.504482	-2.414588
H	1.604405	-4.187603	-3.879273
O	2.134152	-1.836630	-1.600727

Statistical Thermodynamic Analysis
Temperature 298.150 Kelvin. Pressure
1.00000 Atm.

SCF = -5255.21388273 | Predicted change in Energy=-8.841015D-08
Zero-point correction (ZPE) = -5254.61427873 0.599604
Internal Energy (U) = -5254.55844473 0.655438
Enthalpy (H) = -5254.557500729999 0.656382
Gibbs Free Energy (G) = -5254.717538729999 0.496344

Frequencies
8.5405 15.6376 17.4646
18.5004 23.2447 28.4951
29.4221 30.5856 32.6186
34.1100 35.8651 37.4591
39.0339 42.5507 45.3274
46.1547 47.5633 47.6808
55.2799 58.0155 64.2782
68.0311 70.2634 72.2823

72.9135 76.2351 84.2010
87.2071 89.1731 90.9310
93.1281 94.0139 102.9039
105.5457 114.2189 128.2982
145.5432 170.8506 173.9459
177.4672 181.6879 187.8077
204.3808 211.9172 220.4994
224.5029 233.8247 249.2770
264.0960 276.3518 278.6691
285.3965 285.9757 292.9220
295.8673 315.9354 316.8214
323.6910 326.0681 330.8600
331.9935 332.5659 333.0381
345.2852 349.5642 397.4891
400.4719 424.4276 425.1467
426.4663 427.0382 431.4345
435.2553 490.3873 493.9715
507.4795 510.9564 538.6506
539.1287 549.7251 550.4262
553.6694 554.1248 561.3022
562.0502 578.3053 585.0504
586.7869 588.1235 588.7342
591.0345 591.3294 615.4771
617.1730 642.5062 646.2024
674.3370 722.1439 724.2459
763.2744 763.9390 776.5159
779.6099 785.6920 786.1927
804.9529 805.3519 818.2926
819.9892 840.5147 841.6250
842.0772 843.5317 846.9954
877.7236 884.7157 906.1302
907.0541 910.3886 911.3465
919.1175 936.0031 971.7554
978.8822 979.7653 1003.8633
1004.0335 1008.1968 1010.7081
1023.3678 1024.6711 1026.7081
1029.2930 1032.0618 1032.8142
1048.2696 1050.0101 1062.0693
1066.4450 1076.8419 1077.2119
1078.3140 1079.5985 1091.4634
1100.5827 1170.3315 1193.9894
1200.1502 1201.4274 1202.4324
1207.0504 1207.4462 1207.9834
1208.3615 1210.1978 1211.5273
1211.7998 1212.8840 1232.0856
1238.8850 1242.5545 1253.1292
1262.7767 1263.8966 1265.7628
1266.4464 1269.8978 1271.1989
1275.4977 1275.6635 1283.6686
1288.2237 1296.1385 1304.8530
1329.0273 1332.7324 1334.2768
1337.5788 1339.6735 1345.3011
1345.8008 1347.2234 1348.7827
1353.7784 1353.8143 1356.9888
1358.9486 1359.4598 1362.4247
1363.1430 1364.2813 1364.8239
1381.0695 1382.3915 1382.9343
1405.4761 1406.7242 1410.3590
1495.3558 1495.7294 1498.3561
1498.6204 1501.6174 1506.2980
1506.4269 1509.0007 1510.3747
1511.0495 1512.7050 1512.9399
1523.0449 1525.1563 1529.8929
1541.3477 3045.9910 3047.1739
3049.0826 3050.5735 3053.6092
3054.3385 3055.1374 3058.1390
3059.6068 3060.0674 3064.0562
3065.4656 3071.5984 3072.6787
3075.3719 3082.0152 3092.6015
3094.3073 3096.2475 3097.2880
3098.6722 3099.5046 3120.7861
3125.4995 3127.7200 3129.2565
3132.8871 3135.3904 3137.1955
3138.5260 3138.7712 3152.0398

$Ca(NTf_2)_2(Pip)_2(THF)$ 13

Gaussian 16: ES64L-G16RevB.01 20-Dec-2017

B3LYP/6-31G(d,p) gfprint gfinput scf=(direct,tight,maxcycle=300,xqc) opt=(maxcycle=250) freq=noraman
N.EDU\09-Jan-2022\0\\# B3LYP/6-31G(d,p) gfprint gfinput scf=(direct,ti ght,maxcycle=300,xqc) opt=(maxcycle=250) freq=noraman\\010e_1T_052.xyz
----
Full point group C1 NOp 1
Stoichiometry C18H30CaF12N4O9S4
Framework group C1[X(C18H30CaF12N4O9S4)]

Num atoms: 78
Charge = 0 Multiplicity = 1

SCF = -5068.37299229 I Predicted change in Energy=-2.484542D-08

Optimization completed.
Maximum Force 0.000033 0.000450 YES
RMS Force 0.000003 0.000300 YES
Maximum Displacement 0.001529 0.001800 YES
RMS Displacement 0.000293 0.001200 YES

Atom Coordinates (in Angstroms)
Type X Y Z

Ca	2.702184	2.487435	-0.069737
S	3.144150	-0.748382	-1.891262
O	4.416657	-1.165321	-2.469368
O	3.203715	0.183305	-0.728940
C	2.416923	-2.303088	-1.144427
F	1.173322	-2.072340	-0.703615
F	2.389798	-3.279004	-2.045074
F	3.178483	-2.670749	-0.107703
N	2.106092	-0.328939	-3.041849
S	1.100591	0.928320	-3.020044
O	1.118446	1.621328	-4.303425
O	1.177193	1.774705	-1.794968
C	-0.598146	0.154226	-2.891176
F	-0.776057	-0.724452	-3.873441
F	-0.735167	-0.462158	-1.712379
F	-1.509813	1.123140	-2.985686
S	0.596569	4.708935	1.842448
O	0.840884	3.965156	0.572447
O	0.046541	3.916924	2.949300
C	-0.674576	5.996181	1.373603
F	-0.238359	6.709437	0.334926
F	-1.788888	5.349667	1.029058
F	-0.921922	6.798839	2.401886
N	1.797795	5.722883	2.236376
S	3.299516	5.193481	2.426237
O	4.255486	6.262814	2.160214
O	3.551407	3.869771	1.777911
C	3.470745	4.785716	4.245686
F	3.209598	5.859314	4.983480
F	2.620093	3.804152	4.565210
F	4.720042	4.374397	4.477738
C	2.564060	0.165589	2.594829
C	1.915250	-0.543774	3.790664
C	0.562456	-1.154772	3.397006
C	-0.342612	-0.090624	2.759715
C	0.365725	0.601852	1.589457
H	2.826050	-0.565029	1.821194
H	3.491826	0.663501	2.898310
H	1.769642	0.180667	4.604210
H	2.596247	-1.314171	4.171902
H	0.075615	-1.604085	4.269726
H	0.729168	-1.966958	2.675355
H	-1.279343	-0.537171	2.405722
H	-0.614888	0.665354	3.508712
H	0.541563	-0.115095	0.781129
H	-0.250497	1.410089	1.186270
N	1.684971	1.180940	1.961466
H	1.498391	1.907313	2.655941
C	5.882197	2.197100	-1.588534
C	7.410564	2.310440	-1.650030
C	8.066777	1.397216	-0.603872
C	7.491573	1.673437	0.793216
C	5.959217	1.591585	0.787248
H	5.575915	1.184070	-1.870398
H	5.405584	2.889001	-2.291002
H	7.706936	3.353897	-1.468533
H	7.754772	2.055499	-2.658983
H	9.154194	1.531329	-0.603647
H	7.877957	0.349230	-0.873231
H	7.892368	0.963945	1.526895
H	7.795889	2.675827	1.126846
H	5.635842	0.565588	0.576762
H	5.554160	1.876069	1.764150
N	5.327047	2.464248	-0.235899
H	5.559691	3.426994	0.014459
C	2.304277	6.189169	-3.040457
C	1.946360	4.789949	-2.539527
C	3.944484	5.518724	-1.464194
C	3.147165	6.748417	-1.885764
H	2.900713	6.131635	-3.957521
H	1.413643	6.785497	-3.253829
H	1.859330	4.042374	-3.329369
H	1.027772	4.792598	-1.945628
H	4.261022	5.544579	-0.419484
H	4.825525	5.375118	-2.104272
H	3.791759	7.581282	-2.177802
H	2.505471	7.080541	-1.062960
O	3.043775	4.394222	-1.650371

Statistical Thermodynamic Analysis
Temperature 298.150 Kelvin. Pressure 1.00000 Atm.

SCF = -5068.37299229 | Predicted change in Energy=-2.484542D-08
Zero-point correction (ZPE) = -5067.82133829 0.551654
Internal Energy (U) = -5067.76752429 0.605468
Enthalpy (H) = -5067.76658029 0.606412
Gibbs Free Energy (G) = -5067.92257829 0.450414

Frequencies
7.1677 11.2929 13.6896
15.7317 18.7814 25.3523
25.8605 28.5278 30.8340
32.7334 35.8620 37.2100
38.3212 39.6751 43.8725
46.0667 49.3885 54.1600
59.2339 66.4048 72.2707
75.0752 79.4820 83.4473
85.0843 90.6743 94.7576
98.9692 104.6432 108.3213
112.4213 117.1827 122.1976
125.3317 160.5506 174.5561
178.2964 179.4045 189.5794
196.3421 205.9580 214.9171
221.7645 230.5382 236.0419
241.7070 245.3439 249.8556
274.2912 275.7173 276.1403
279.6842 282.7776 286.2840
287.2922 290.5872 295.5759
315.3696 317.6101 319.1659
326.6861 331.4274 353.4509
395.6449 396.6347 408.6252
412.9399 417.0762 448.0169
454.3043 455.5165 459.4974
460.5576 490.3536 496.5110
503.9920 522.1119 537.8588
540.0993 548.7424 550.1599
553.5662 561.0113 561.8396
563.1768 577.7341 579.7379
580.2648 583.3468 591.8350
632.9012 647.2487 677.8506
716.9676 722.7605 761.6332
764.5137 782.0108 787.7365
813.4849 814.7183 815.2397
816.7208 849.3171 855.9392
859.1880 873.3629 875.9837
882.4304 883.8858 885.3063
886.6918 921.6312 933.5781
955.9053 957.9054 978.6328
988.0682 1004.6315 1008.6596
1016.1252 1028.2101 1030.3777
1045.9227 1048.7536 1058.3445
1060.7381 1061.3946 1064.7935
1070.5030 1083.4978 1091.3305
1100.2208 1100.6341 1102.2528
1131.7249 1134.6416 1175.7100
1197.2143 1199.8403 1204.4774
1204.5652 1206.4417 1209.6279
1211.5191 1214.2983 1215.4454
1215.7764 1226.3971 1235.3926
1248.9880 1251.0103 1262.3389
1263.8315 1266.5440 1267.9372
1272.1581 1275.3181 1281.9498
1288.2864 1295.6154 1297.0615
1297.4855 1308.5860 1309.4965
1311.2291 1331.0612 1349.6037
1352.9521 1359.0863 1360.3629
1361.5318 1383.5572 1384.2503
1385.9837 1391.4366 1392.3564
1398.0711 1399.3279 1412.6545
1415.8675 1416.5629 1473.1570
1476.8328 1497.5277 1498.4873
1498.9030 1499.6231 1500.8514
1502.0070 1503.3638 1506.0034
1509.0959 1512.0724 1519.5065
1523.3291 1536.8998 1550.2419
3016.9767 3021.7304 3022.5079
3024.8960 3026.0911 3032.7073
3033.7364 3049.1880 3052.4275
3059.7236 3064.1616 3071.1405
3072.5223 3074.4170 3075.8176
3079.3711 3079.5426 3079.8458
3084.4571 3085.0184 3099.4897
3101.9581 3110.0665 3122.4414
3125.1857 3126.1246 3133.8301
3148.9517 3466.4604 3467.9512

$Ca(NTf_2)_2(Pip)_3$ 14

Gaussian 16: ES64L-G16RevB.01 20-Dec-2017

B3LYP/6-31G(d,p) gfprint gfinput scf=(direct,tight,maxcycle=300,xqc) opt=(maxcycle=250) freq=noraman
N.EDU\10-Jan-2022\0\\# B3LYP/6-31G(d,p) gfprint gfinput scf=(direct,ti

ght,maxcycle=300,xqc) opt=(maxcycle=250)
freq=noraman\\020h_0T_015.xyz

Full point group C1 NOp 1
Stoichiometry C19H33CaF12N5O8S4
Framework group
C1[X(C19H33CaF12N5O8S4)]

Num atoms: 82
Charge = 0 Multiplicity = 1

SCF = -5087.83570070 l Predicted change in
Energy=-1.314929D-08

Optimization completed.
Maximum Force 0.000014
0.000450 YES
RMS Force 0.000002 0.000300
YES
Maximum Displacement 0.001701
0.001800 YES
RMS Displacement 0.000296
0.001200 YES

Atom Coordinates (in Angstroms)
Type X Y Z

Ca 0.124638 2.831289 3.960009
S -3.301146 2.905267 2.796882
O -2.357819 2.552353 3.905034
O -3.519949 4.344757 2.596577
C -4.931668 2.167759 3.334497
F -5.300925 2.796756 4.453667
F -5.852833 2.348074 2.396258
F -4.787153 0.868380 3.587940
N -3.076681 2.019567 1.468647
S -1.630997 1.982625 0.756962
O -1.372251 0.666343 0.182800
O -0.555506 2.599646 1.587044
C -1.781522 3.166539 -0.684841
F -2.018521 4.397866 -0.222845
F -0.634362 3.162704 -1.364052
F -2.776361 2.788662 -1.481592
N 3.885814 4.400195 4.299390
S 3.023406 4.439241 5.647654
O 1.726541 3.701405 5.564476
O 2.994538 5.789878 6.194564
S 3.694994 3.266684 3.160188
O 2.266944 2.986554 2.826952
O 4.567981 2.098916 3.284580
C 4.313866 4.253323 1.698149
F 4.211889 3.476689 0.618036
F 3.570308 5.347065 1.525147
F 5.583652 4.598738 1.882228
C 3.978470 3.397410 6.873669
F 5.170481 3.932853 7.111132
F 3.284084 3.329823 8.017716
F 4.129335 2.161678 6.385990
C -0.481306 -1.983296 2.896088
C -0.629674 -0.634942 3.611012
C 1.805419 -0.365509 3.746416
C 2.026328 -1.707388 3.036721
C 0.845518 -2.661773 3.266982
H -0.526752 -1.810159 1.812845
H -1.332989 -2.626313 3.148211
H -0.704439 -0.794164 4.695755
H -1.546274 -0.129013 3.293962
H 2.616267 0.331240 3.521029
H 1.797648 -0.518544 4.833925
H 2.148156 -1.523578 1.960221
H 2.964717 -2.153651 3.385952
H 0.819215 -2.956314 4.326076
H 0.978576 -3.583945 2.690556
N 0.517138 0.282499 3.381458
H 0.552734 0.463507 2.375151
C -2.066734 2.797270 8.369168
C -1.377725 3.202444 7.059336
C 0.144976 1.289307 7.200200
C -0.502417 0.821472 8.510325
C -1.111387 2.006247 9.274241
H -2.948513 2.183413 8.136368
H -2.435320 3.694351 8.880431
H -0.566843 3.911766 7.264752
H -2.088338 3.691898 6.385969
H 0.522731 0.435292 6.628043
H 1.000330 1.938717 7.414179
H -1.287385 0.085301 8.284491
H 0.245393 0.305265 9.123795
H -0.304189 2.668808 9.615640
H -1.632623 1.657964 10.172741
N -0.776004 2.058272 6.327130
H -1.543071 1.442793 6.050952
C 0.183158 5.892475 2.327725
C -0.308662 7.285869 1.916827
C -0.271290 8.258439 3.104182
C -1.033223 7.674002 4.302313
C -0.518649 6.270947 4.649011

H 1.245545 5.934082 2.593109
H 0.081201 5.186404 1.499571
H -1.336411 7.203741 1.536548
H 0.305544 7.658299 1.088364
H -0.690686 9.229880 2.819358
H 0.773376 8.438342 3.393218
H -0.936577 8.324056 5.179978
H -2.104984 7.616319 4.065613
H 0.515770 6.328057 5.005722
H -1.113043 5.833708 5.458245
N -0.550160 5.332034 3.496020
H -1.529418 5.238169 3.212814

Statistical Thermodynamic Analysis
Temperature 298.150 Kelvin. Pressure 1.00000 Atm.

SCF = -5087.83570070 l Predicted change in Energy=-1.314929D-08
Zero-point correction (ZPE) = -5087.2416137 0.594087
Internal Energy (U) = -5087.1869237 0.648777
Enthalpy (H) = -5087.185979700001 0.649721
Gibbs Free Energy (G) = -5087.342501700001 0.493199

Frequencies
7.8199 16.9212 17.1690
19.9486 25.7748 29.8300
30.8498 32.2038 33.0556
33.7568 35.3068 36.7456
39.3716 40.4227 42.5995
45.5660 48.5330 50.8308
53.2375 56.6298 61.3963
66.1818 73.4154 81.8542
85.6303 89.1504 92.3294
95.2303 98.3888 103.0667
104.7897 109.7670 112.3705
115.4511 164.4898 168.9682
177.6790 179.3669 189.7696
205.5808 213.2764 216.3833
226.0537 232.0209 239.3674
242.9621 243.8843 245.1418
274.0141 277.0962 280.1961
282.3996 285.4956 286.7213
289.1982 291.0287 296.2448
315.8510 319.6166 324.7075
329.4333 346.7573 349.1816
396.6750 400.1746 411.6636
413.6001 418.3668 431.7865
449.1881 453.8734 454.1110
455.2204 459.4292 459.9233
460.7577 487.3207 495.1018
502.6336 511.3308 538.6596
538.7597 550.1253 550.5828
553.6341 554.5028 560.4278
563.5132 579.4159 582.5350
584.2057 589.7188 592.1559
640.1518 653.2413 720.8950
725.4906 763.2424 765.1864
785.4972 789.0169 812.2949
812.6953 813.8750 814.9323
815.2369 817.2375 854.1331
857.1600 860.0203 873.8160
875.1826 876.9952 884.6892
885.7394 886.2067 954.8736
956.4829 957.5417 983.2166
998.7842 1002.3124 1012.9845
1016.3162 1028.5624 1030.9307
1031.2352 1051.4461 1059.5394
1060.4315 1061.0377 1063.2495
1064.7969 1070.9415 1081.7310
1090.1430 1099.1057 1099.3767
1100.4222 1102.2115 1131.4996
1131.8063 1138.6264 1198.4184
1202.0104 1204.3987 1206.5245
1208.7002 1212.0556 1214.8695
1215.3315 1216.3884 1216.5480
1231.3967 1237.4519 1249.8649
1252.8598 1259.4148 1263.7782
1266.5042 1268.7729 1282.7044
1284.5147 1295.2479 1296.0799
1299.6258 1301.0928 1303.5717
1307.7595 1308.4035 1312.3786
1346.5400 1348.9439 1352.1154
1359.4511 1360.7122 1362.7524
1382.3804 1384.3872 1387.5712
1390.8635 1392.3120 1393.8915
1397.1685 1398.3039 1400.5737
1410.3762 1412.5703 1416.9739
1473.6161 1476.7436 1487.5230
1497.6764 1498.5753 1498.9030
1499.2637 1499.9976 1501.3835
1502.5152 1504.2817 1504.7605
1504.8767 1507.6726 1510.1118
1518.9889 1519.1447 1521.5774

3018.7729 3018.9198 3022.2770
3023.9134 3025.3649 3026.1237
3028.8632 3030.8258 3031.4513
3037.4376 3038.3670 3049.1522
3055.9127 3059.7438 3062.9079
3076.4558 3076.7385 3077.9792
3078.1315 3078.2915 3080.6537
3083.8637 3084.2039 3085.6901
3103.3738 3103.6452 3104.8310
3110.7836 3122.5126 3124.9939
3442.4657 3455.9405 3471.0219

Preorganized Ca^{2+} Complex 15

Gaussian 16: ES64L-G16RevB.01 20-Dec-2017

B3LYP/6-31G(d,p) gfprint gfinput scf=(direct,tight,maxcycle=300,xqc) opt=(maxcycle=250) freq=noraman
AN.EDU\23-Apr-2021\0\\# B3LYP/6-31G(d,p) gfprint gfinput scf=(direct,t ight,maxcycle=300,xqc) opt=(maxcycle=250) freq=noraman\\023_2T_032.xyz
----
Full point group C1 NOp 1
Stoichiometry C29H44CaF13N5O12S5
Framework group C1[X(C29H44CaF13N5O12S5)]

Num atoms: 109
Charge = 0 Multiplicity = 1

SCF = -6274.33383497 l Predicted change in Energy=-1.137746D-08

Optimization completed.
Maximum Force 0.000012 0.000450 YES
RMS Force 0.000001 0.000300 YES
Maximum Displacement 0.001142 0.001800 YES
RMS Displacement 0.000173 0.001200 YES

Atom Type Coordinates (in Angstroms) X Y Z

Ca 0.486292 -0.997888 -0.514592
S 3.738277 0.021107 0.749929
O 3.924919 0.011202 2.197739
O 2.796638 -0.990590 0.181570
C 5.377328 -0.457811 -0.008468
F 5.291922 -0.478042 -1.343776
F 6.331657 0.386555 0.363379
F 5.673282 -1.688181 0.421077
N 3.593959 1.479382 0.064852
S 2.191535 2.238154 -0.104167
O 0.998945 1.342014 -0.095751
O 2.288237 3.216548 -1.185369
C 1.975885 3.230799 1.468844
F 0.857961 3.963918 1.365263
F 1.868868 2.407185 2.508347
F 3.016129 4.041234 1.639677
S -0.376813 -1.761787 3.028695
O -1.057314 -1.089119 4.125028
O 0.023343 -0.948587 1.847487
C 1.263200 -2.358076 3.704351
F 1.976535 -1.314569 4.109728
F 1.063972 -3.192065 4.721756
F 1.923860 -3.001805 2.728650
N -1.146327 -3.127371 2.650804
S -1.155076 -3.794223 1.196034
O -0.295507 -4.972031 1.044789
O -1.137732 -2.822173 0.058250
C -2.907492 -4.441984 1.188961
F -3.145800 -4.971748 -0.024297
F -3.065617 -5.378810 2.114934
F -3.780167 -3.452559 1.392894
S -2.640069 0.044279 -2.510085
O -1.674049 -0.251629 -1.449958
F -2.037119 -0.810682 -3.770202
O -2.833434 1.380467 -3.028578
C -6.476166 -2.312610 -2.123959
C -5.312537 -2.784561 -1.512149
C -4.129201 -2.053075 -1.596810
C -4.150803 -0.848718 -2.300354
C -5.298777 -0.356458 -2.923267
C -6.470628 -1.103657 -2.824929
H -7.393756 -2.888254 -2.051947
H -5.318116 -3.718275 -0.961765
H -3.219519 -2.397705 -1.119797
H -5.271713 0.587010 -3.455660
H -7.379107 -0.739505 -3.293400
C -6.045043 0.736871 0.820991
C -5.326254 1.909975 0.145594

C	-3.227480	1.345429	1.228622
C	-3.858131	0.142811	1.935264
H	-6.027047	-0.128259	0.147305
H	-7.098076	0.996900	0.985438
H	-5.472271	2.815940	0.766063
H	-5.774640	2.121372	-0.832772
H	-2.166987	1.159354	1.031382
H	-3.280678	2.219697	1.905830
H	-3.705294	-0.751971	1.319750
H	-3.351138	-0.032572	2.889835
N	-3.901794	1.612926	-0.051403
H	-3.467472	2.439584	-0.473001
N	-2.588883	4.461314	-0.823549
C	-1.326025	4.199667	-1.549645
C	-0.519305	5.526547	-1.719960
C	-1.457040	6.367825	0.318360
C	-2.269102	5.043586	0.498104
C	-3.382058	5.441299	-1.592793
C	-2.570248	6.767855	-1.765035
H	-0.758966	3.456242	-0.982013
H	-1.583741	3.749408	-2.513590
H	0.462082	5.452860	-1.241876
H	-0.351842	5.760727	-2.777456
H	-0.473332	6.294571	0.793579
H	-1.977811	7.221574	0.766981
H	-1.702295	4.300861	1.067401
H	-3.212303	5.216900	1.028567
H	-3.629607	4.990610	-2.560318
H	-4.323679	5.612086	-1.058478
H	-2.407147	7.000952	-2.823431
H	-3.098321	7.619283	-1.320513
N	-1.255076	6.651331	-1.113139
C	-5.362301	0.367830	2.146504
H	-5.506302	1.186040	2.866586
H	-5.829000	-0.521233	2.586870
C	2.422320	-3.942369	-0.795449
C	2.195421	-5.347722	-1.351874
C	1.625997	-5.067656	-2.750970
C	0.748290	-3.846867	-2.488958
H	2.263645	-3.877913	0.281827
H	3.417236	-3.549863	-1.030510
H	3.116581	-5.935814	-1.371003
H	1.457859	-5.872637	-0.738675
H	2.427733	-4.819726	-3.455664
H	1.057130	-5.905820	-3.161578
H	-0.238861	-4.133394	-2.109437
H	0.624648	-3.191510	-3.356155
O	1.439672	-3.091992	-1.461639
C	2.530511	-0.716470	-3.373887
C	0.830799	0.887726	-3.501189
C	2.067002	1.485031	-4.207656
C	3.234048	0.580509	-3.758119
H	3.060198	-1.320027	-2.637912
H	2.307557	-1.334489	-4.256420
H	0.061985	0.572649	-4.213328
H	0.387787	1.564376	-2.769732
H	1.938313	1.452485	-5.293546
H	2.230340	2.522990	-3.912887
H	3.732910	1.003394	-2.883108
H	3.982025	0.431283	-4.541564
O	1.291558	-0.283672	-2.769383

Statistical Thermodynamic Analysis
Temperature 298.150 Kelvin. Pressure
1.00000 Atm.

SCF = -6274.33383497 l Predicted change in
Energy=-1.137746D-08
Zero-point correction (ZPE) = -6273.53643597
0.797399
Internal Energy (U) = -6273.464421969999
0.869413
Enthalpy (H) = -6273.46347797   0.870357
Gibbs Free Energy (G) = -6273.66189797
0.671937

Frequencies
7.5245 13.4222 15.0419
18.8718 21.7518 23.2808
25.6493 27.5616 29.4862
30.2366 33.7149 34.6111
35.8553 36.5542 38.4324
40.4792 42.2974 42.9968
45.9094 47.0239 48.4135
49.2173 50.8351 52.4407
54.2379 55.4079 56.4936
59.9849 60.8687 61.9405
64.9056 68.8498 69.7310
71.2494 73.2697 75.1699
77.6946 81.4182 86.3537
92.8965 93.1394 103.8115
105.1630 107.7464 110.4023
113.3619 116.8814 124.0877
134.2191 138.0654 168.0882
173.2446 178.7480 181.9482
183.8987 189.0378 200.0755
202.4359 214.4106 226.0198
230.9434 241.4230 247.4739

256.1671 262.8160 265.8822
272.3713 275.1216 277.7915
279.2596 283.5808 287.2815
293.7517 298.5415 310.4291
314.0602 316.9371 324.5097
327.1489 339.7211 342.6493
342.6835 347.7065 351.7767
396.4667 398.1345 403.5667
412.1174 419.2458 427.9680
429.1059 431.1058 437.7799
449.5354 454.3277 474.3356
493.0834 493.7861 506.9446
508.2154 513.1893 537.2423
538.0588 549.8289 550.7724
554.0122 556.0064 561.6303
561.7983 567.2124 573.9191
580.3900 588.1639 588.8495
589.7008 590.7795 604.5185
611.6810 623.4942 636.7498
639.4940 683.8783 690.1604
698.4888 711.6771 717.6452
719.3763 751.3682 762.5062
763.4799 767.4985 769.0115
782.6500 783.7643 809.2065
814.0464 814.8975 819.1505
823.5425 836.1646 838.3757
849.4009 853.3771 861.0937
878.3126 878.7597 881.2470
883.1779 890.4210 897.7754
900.3868 904.3428 919.8875
923.4603 931.7406 943.8955
947.9933 968.0663 975.1740
975.9067 977.2252 982.0684
988.9160 996.7191 1010.0773
1011.8780 1014.7397 1016.3742
1030.8737 1036.6171 1040.3631
1046.9423 1047.5869 1053.8317
1056.6209 1057.8202 1061.0041
1066.1549 1068.1043 1072.6059
1076.2481 1078.3592 1087.9981
1091.1134 1100.4807 1110.9642
1114.1063 1133.2528 1162.2484
1169.3740 1173.8722 1174.5216
1182.4164 1192.2618 1196.7802
1197.6599 1198.4616 1200.4005
1201.7364 1202.0666 1205.4701
1208.9383 1209.9424 1212.2625
1214.5821 1216.5678 1232.1009
1233.0378 1238.5659 1239.2152
1245.2681 1254.8628 1255.8430
1261.5448 1267.4791 1268.2630
1269.4095 1271.1857 1274.4172
1281.9977 1286.3753 1291.8431
1296.6478 1297.8414 1299.1611
1314.2613 1318.8997 1327.8123
1330.3244 1332.3579 1333.9885
1336.5214 1340.1106 1342.2899
1346.8796 1350.1879 1352.0867
1356.1246 1357.1750 1358.8129
1359.7221 1361.7033 1371.5325
1373.6646 1376.3945 1380.0077
1381.3539 1387.6197 1392.1815
1393.6474 1409.1633 1411.9404
1433.4160 1491.5957 1492.6365
1494.8078 1496.5922 1499.8228
1502.0305 1502.4386 1505.3357
1507.0737 1508.0392 1508.7998
1510.6862 1510.9593 1516.4266
1519.4368 1520.6902 1524.2630
1526.1673 1530.1739 1543.3272
1545.2721 1548.3328 1637.1878
1642.7717 2918.8811 2928.6265
3008.3925 3020.3168 3034.9494
3037.7144 3041.8173 3043.2106
3047.4978 3049.8770 3052.3667
3055.0629 3055.6452 3059.7026
3063.8800 3064.7966 3069.5615
3070.2783 3074.9172 3079.6091
3083.0157 3084.4894 3086.1637
3086.8475 3088.3637 3097.1979
3098.2348 3102.0021 3106.8900
3110.4852 3119.4296 3122.9906
3131.3359 3134.8726 3141.5941
3147.5992 3153.7157 3163.0373
3197.0043 3211.5285 3224.9858
3231.7035 3234.9080 3401.9592

Transition State Complex 16

Gaussian 16: ES64L-G16RevB.01 20-Dec-2017

# B3LYP/6-31G(d,p) gfprint gfinput
scf=(direct,tight,maxcycle=300,xqc)
opt=(maxcycle=250,ts,calcfc,noeigentest)
freq=noraman
N.EDU\12-Feb-2021\0\\# B3LYP/6-31G(d,p)
gfprint gfinput scf=(direct,ti

ght,maxcycle=300,xqc)
opt=(maxcycle=250,ts,calcfc,noeigentest)
freq=no

Full point group C1 NOp 1
Stoichiometry C21H28CaF13N5O10S5
Framework group
C1[X(C21H28CaF13N5O10S5)]

Num atoms: 83
Charge = 0 Multiplicity = 1

SCF = -5809.36770317 | Predicted change in Energy=-5.754536D-11

Optimization completed.
Maximum Force 0.000001 0.000450 YES
RMS Force 0.000000 0.000300 YES
Maximum Displacement 0.000440 0.001800 YES
RMS Displacement 0.000060 0.001200 YES

Atom Coordinates (in Angstroms)
Type X Y Z

Ca	-0.923886	-0.076735	-1.044845
S	-3.579251	-2.543430	-1.219334
O	-2.836484	-1.323050	-1.658549
O	-4.615242	-3.049192	-2.104082
C	-4.428663	-1.956133	0.340168
F	-5.214018	-0.918880	0.044712
F	-5.156653	-2.930778	0.872700
F	-3.494801	-1.562147	1.218844
N	-2.649817	-3.750961	-0.692040
S	-1.102321	-3.638602	-0.291538
O	-0.760204	-4.601285	0.750017
O	-0.582606	-2.249325	-0.107178
C	-0.209410	-4.199010	-1.835469
F	-0.557698	-5.439714	-2.157211
F	-0.510516	-3.371658	-2.841985
F	1.110758	-4.154370	-1.610205
S	-0.839636	2.024570	1.849976
O	0.498096	2.015068	2.445122
O	-1.316216	0.765858	1.209712
C	-2.007811	2.257808	3.294765
F	-1.649913	3.334451	3.990061
F	-3.259114	2.406521	2.856223
F	-1.936875	1.178277	4.074918
N	-1.043123	3.358834	0.967773
S	-1.770652	3.437039	-0.471356
O	-1.653209	2.187733	-1.274446
O	-1.387302	4.688863	-1.111658
C	-3.616080	3.556538	-0.156418
F	-4.075131	2.370086	0.254235
F	-3.861571	4.474015	0.776952
F	-4.224027	3.896716	-1.290732
S	2.387247	0.330325	-1.320533
O	1.364712	0.353242	-0.238273
F	0.641809	0.061422	-2.586554
O	2.952653	-0.857427	-1.959766
C	2.518371	4.083969	-3.889159
C	1.821548	4.104309	-2.679423
C	1.786077	2.966912	-1.873283
C	2.449700	1.820919	-2.310933
C	3.142136	1.773169	-3.520681
C	3.176467	2.924596	-4.306796
H	2.537514	4.970387	-4.515420
H	1.280265	4.989039	-2.361956
H	1.236658	2.973418	-0.940203
H	3.606248	0.850256	-3.848609
H	3.703113	2.907020	-5.255673
C	4.776455	2.079607	1.706173
C	3.635108	1.893498	0.701066
C	5.139146	0.645871	-0.818085
C	6.310095	0.831395	0.153579
H	4.745847	1.281692	2.458653
H	4.599254	3.017095	2.243593
H	3.613608	2.753491	0.022003
H	2.668819	1.826831	1.203155
H	5.216473	-0.296882	-1.361297
H	5.140856	1.461959	-1.551646
H	6.397242	-0.048451	0.801480
H	7.232752	0.876778	-0.435391
N	3.822276	0.651443	-0.114517
H	3.679784	-0.367385	0.642192
N	3.450201	-1.461602	1.463077
C	4.692046	-1.865569	2.187401
C	4.357674	-3.024622	3.178233
C	2.794113	-3.867603	1.578135
C	2.971299	-2.629930	0.648099
C	2.393513	-1.116849	2.473007
C	2.054091	-2.388454	3.308425
H	5.426759	-2.171568	1.436034
H	5.086284	-0.987356	2.707488

H 5.038019 -3.868468 3.025661
H 4.471284 -2.693296 4.215397
H 3.523366 -4.648164 1.337993
H 1.796025 -4.296288 1.456725
H 3.696944 -2.800654 -0.150726
H 2.030829 -2.338368 0.183406
H 2.787574 -0.308658 3.096465
H 1.530546 -0.729188 1.932919
H 2.116265 -2.176618 4.380601
H 1.037778 -2.730138 3.094633
N 2.980125 -3.485669 2.987248
C 6.141490 2.092667 1.009034
H 6.213839 2.981630 0.368259
H 6.950079 2.167159 1.743659

Statistical Thermodynamic Analysis
Temperature 298.150 Kelvin. Pressure 1.00000 Atm.

SCF = -5809.36770317 l Predicted change in Energy=-5.754536D-11
Zero-point correction (ZPE) = -5808.80962217 0.558081
Internal Energy (U) = -5808.75271717 0.614986
Enthalpy (H) = -5808.75177317 0.61593
Gibbs Free Energy (G) = -5808.91181617 0.455887

Frequencies
-1057.6729 5.0190 9.6792
10.8061 17.3842 20.2583
25.3715 26.9139 30.1611
31.9156 34.1390 36.5904
36.9884 39.5045 41.5107
44.1192 47.6928 53.3594
54.4434 64.9001 67.9457
79.8226 84.1101 84.9421
89.8514 98.1623 101.9487
104.8301 107.4441 116.3284
122.1995 125.8788 133.1742
152.4797 163.4081 170.9276
183.7483 185.6921 195.4362
197.7168 200.6864 203.2897
204.6015 209.5948 214.1380
228.4855 237.2629 243.4355
250.6034 266.7966 270.1384
277.1535 279.8700 281.9373
284.4449 287.2059 296.5713
296.8562 305.4929 308.7733
312.9977 325.4611 326.3681
329.5678 330.2420 334.9787
339.0741 349.6955 363.6472
391.0486 394.9368 396.2177
411.3144 416.5362 423.4566
426.6244 454.1052 455.7920
461.0242 466.4830 493.3248
494.4635 498.5590 502.9643
519.0975 522.4473 537.6314
539.6234 548.1134 548.8643
556.4583 561.1888 566.0671
566.8800 569.6428 577.5782
581.4270 582.2936 582.8334
594.5034 607.2336 622.6231
627.0539 633.0183 677.8807
693.2081 713.5995 715.2903
718.7774 761.0053 762.1738
763.1265 781.0039 782.5204
796.8986 804.6571 815.5552
818.4772 834.6834 843.2072
846.5522 862.4311 872.1231
902.8917 907.8514 911.1390
916.6928 945.3465 977.9022
980.4942 990.3519 1004.1936
1005.1417 1015.6445 1015.9029
1017.5041 1023.6469 1027.4098
1031.6980 1035.9539 1041.8494
1045.3655 1055.8032 1068.9428
1070.0745 1073.8960 1077.1973
1079.7207 1084.8645 1098.0945
1098.8814 1108.5105 1113.4718
1178.8167 1185.3001 1191.8901
1199.0555 1201.8853 1206.1662
1206.9442 1208.3342 1209.9535
1210.6505 1211.9103 1229.3778
1234.1925 1237.0464 1242.1450
1253.1423 1260.3472 1263.3348
1269.1190 1271.4171 1274.4604
1283.8686 1288.3215 1291.6049
1293.8019 1307.1922 1316.2721
1321.8402 1322.3570 1332.7204
1341.1792 1345.3952 1348.3240
1352.0189 1357.7986 1359.7214
1362.4262 1363.7299 1364.8889
1368.6621 1389.9183 1391.4496
1394.7561 1399.8656 1416.2474
1424.3058 1488.3380 1493.5045
1499.1051 1499.3006 1500.5371

1508.0653 1509.3429 1511.1926
1515.7120 1516.5455 1519.3348
1529.2358 1537.4394 1634.1689
1641.2347 1670.5117 1692.1702
3035.8991 3045.8415 3047.2346
3053.8055 3056.5982 3060.1505
3061.2960 3063.2510 3065.4719
3080.0542 3090.2192 3096.0279
3096.8094 3097.5059 3102.0948
3116.8576 3119.5940 3123.2530
3143.5505 3150.6292 3169.6492
3175.6493 3195.4493 3208.9429
3220.5674 3228.4257 3237.3095

Post-SuFEx Product-Ca^{2+} Complex 17

Gaussian 16: ES64L-G16RevB.01 20-Dec-2017

B3LYP/6-31G(d,p) gfprint gfinput scf=(direct,tight,maxcycle=300,xqc) opt=(maxcycle=250) freq=noraman AN.EDU\12-Jan-2021\0\\# B3LYP/6-31G(d,p) gfprint gfinput scf=(direct,t ight,maxcycle=300,xqc) opt=(maxcycle=250) freq=noraman\\040-Ca-2NTf2-P

Full point group C1 NOp 1
Stoichiometry C25H36CaF13N5O11S5
Framework group C1[X(C25H36CaF13N5O11S5)]

Num atoms: 96
Charge = 0 Multiplicity = 1

SCF = -6041.91408003 l Predicted change in Energy=-1.110345D-08

Optimization completed.
Maximum Force 0.000014 0.000450 YES
RMS Force 0.000002 0.000300 YES
Maximum Displacement 0.001123 0.001800 YES
RMS Displacement 0.000207 0.001200 YES

Atom Type	Coordinates (in Angstroms) X	Y	Z
Ca	5.004511	2.574679	-3.171714
S	7.008050	2.844777	-6.313296
O	6.539808	3.616260	-7.468674
O	6.539886	3.292580	-4.974309
C	8.851220	3.139227	-6.218630
F	9.082303	4.423987	-5.941792
F	9.363430	2.380056	-5.238463
F	9.432566	2.820604	-7.370904
N	6.859520	1.283599	-6.611612
S	6.741586	0.146969	-5.460731
O	6.117557	0.619148	-4.197539
O	7.928631	-0.694258	-5.338532
C	5.417504	-0.910039	-6.249749
F	5.854265	-1.430748	-7.393891
F	5.111557	-1.894053	-5.400547
F	4.323078	-0.179003	-6.483468
S	5.064461	6.177409	-2.101499
O	4.749193	7.456874	-2.733111
O	5.179278	5.006903	-3.010710
C	6.781397	6.349893	-1.383961
F	6.841286	7.395658	-0.564534
F	7.103456	5.237834	-0.715381
F	7.645143	6.521195	-2.388534
N	4.194062	5.934533	-0.765965
S	3.326012	4.621658	-0.464575
O	3.095707	4.483900	0.967694
O	3.721068	3.400296	-1.214152
C	1.660649	5.037750	-1.209354
F	1.820090	5.369778	-2.504305
F	0.851283	3.970444	-1.145137
F	1.092993	6.053396	-0.568741
S	3.330732	-0.682207	-1.889218
O	3.915415	0.638735	-2.190635
O	4.194672	-1.801276	-1.513629
C	0.792631	-1.946536	-5.465590
C	1.219515	-2.897469	-4.535596
C	2.013834	-2.510594	-3.458177
C	2.369143	-1.163876	-3.325952
C	1.960855	-0.199957	-4.252746
C	1.168688	-0.609839	-5.327006
H	0.177181	-2.251908	-6.306523
H	0.944263	-3.940769	-4.654056
H	2.380553	-3.241056	-2.745828
H	2.287872	0.833431	-4.176995
H	0.854503	0.122209	-6.064533
N	3.051967	4.633471	-6.217417

C 3.129729 4.019333 -7.585303
C 2.808525 5.131106 -8.627839
C 3.787838 6.783135 -7.190563
C 4.082699 5.722651 -6.089904
C 1.684005 5.199008 -5.988782
C 1.440260 6.311913 -7.051916
H 4.136956 3.615150 -7.699641
H 2.414832 3.192914 -7.607833
H 3.636412 5.240888 -9.333525
H 1.912668 4.880576 -9.204290
H 4.630083 6.862337 -7.883363
H 3.631989 7.769110 -6.743738
H 5.059338 5.253328 -6.204035
H 4.013566 6.125458 -5.079057
H 0.975719 4.370442 -6.069559
H 1.653368 5.571098 -4.963455
H 0.544811 6.093654 -7.641749
H 1.288442 7.280633 -6.567432
N 2.586732 6.423577 -7.963070
C 7.308210 0.333249 -0.309469
C 8.417139 0.595371 -1.368831
C 8.163457 2.043275 -1.819986
O 6.776086 2.295384 -1.513249
C 6.545203 1.664773 -0.233349
H 6.642772 -0.467544 -0.640010
H 7.715798 0.053559 0.665733
H 8.313617 -0.089345 -2.213033
H 9.424565 0.479812 -0.960335
H 8.777975 2.760662 -1.259320
H 8.305516 2.214495 -2.887031
H 5.467233 1.567106 -0.103804
H 6.942512 2.320322 0.553449
C 1.253982 -1.221399 1.463701
C 1.708033 -1.597113 0.048910
C 1.373390 0.746376 -0.710258
C 0.908648 1.154177 0.691549
C 0.287715 -0.029810 1.442966
H 2.139523 -0.972629 2.060189
H 0.784809 -2.098222 1.924303
H 0.849579 -1.971495 -0.533870
H 2.471050 -2.375620 0.082811
H 1.908735 1.568402 -1.181753
H 0.504538 0.481231 -1.335511
H 1.768918 1.547232 1.244936
H 0.191783 1.975581 0.591616
H -0.647251 -0.328615 0.947840
H 0.022700 0.262615 2.464631
N 2.292480 -0.407973 -0.603954
F 3.309470 2.783047 -4.595166
H 3.218967 3.827522 -5.468334

Statistical Thermodynamic Analysis
Temperature 298.150 Kelvin. Pressure 1.00000 Atm.

SCF = -6041.91408003 l Predicted change in Energy=-1.110345D-08
Zero-point correction (ZPE) = -6041.23372303 0.680357
Internal Energy (U) = -6041.16911403 0.744966
Enthalpy (H) = -6041.16816903 0.745911
Gibbs Free Energy (G) = -6041.349424030001 0.564656

Frequencies
7.3008 9.3798 10.8451
15.0587 18.8936 20.1301
23.3007 27.4843 30.7157
32.4538 34.0294 34.6905
36.8164 38.5833 40.0741
42.2416 43.6418 44.1279
44.9268 47.0477 48.6632
52.3083 56.3628 57.4900
58.8046 62.9072 63.6339
69.7121 71.2569 74.6058
81.6379 84.4660 86.3446
92.8205 98.5721 108.0650
111.3292 112.9003 118.1455
121.5539 133.8209 177.6317
181.2920 182.4065 188.7375
192.0106 201.1623 204.0008
205.3114 208.0964 213.8847
215.6952 222.6578 231.0370
240.9598 260.6260 268.5011
270.3949 274.3153 282.0493
284.9670 289.2292 291.8565
294.3286 306.5117 311.8370
315.1866 320.0183 322.0236
322.8631 324.4063 331.1802
331.5211 338.0410 343.4997
353.7340 389.2838 392.7445
397.4734 400.5213 402.9569
412.8098 417.2260 421.3444
424.4098 448.1862 457.4973
490.3066 494.1314 499.1526
501.4461 518.7841 522.2395
537.2347 539.3397 548.3848

549.7509 558.4587 561.0993
561.3540 566.0407 569.2647
579.3334 580.4916 582.0524
588.1764 591.9266 614.2424
622.7769 625.9422 633.7779
644.0282 650.9397 688.8155
702.8988 720.6357 721.7216
736.2754 758.7927 762.3030
771.6437 780.2848 782.8851
793.0204 812.2022 816.3741
821.1341 823.0286 841.2227
843.6424 845.3478 870.5239
871.1617 873.6777 902.6933
904.1799 907.6350 920.4385
928.4563 929.5208 947.9960
959.9813 968.0818 977.5631
980.9186 1001.9382 1003.7953
1010.9042 1017.4761 1017.9795
1021.6366 1027.1042 1034.6411
1038.7559 1040.4098 1048.4023
1058.2305 1060.1496 1066.5370
1072.2936 1074.4250 1079.3953
1083.0714 1088.2851 1091.7709
1101.1608 1106.8160 1115.2794
1126.3781 1142.8527 1155.2519
1178.8499 1189.1113 1191.6326
1200.8355 1203.7676 1204.9496
1205.8130 1208.5060 1210.7296
1215.0174 1216.8237 1220.0546
1228.8721 1233.1021 1240.0520
1242.7078 1243.6466 1246.9597
1259.3000 1259.9435 1260.8656
1264.0674 1270.0826 1270.7270
1279.2485 1290.3629 1291.7654
1295.0809 1299.7351 1305.1583
1311.2130 1319.4560 1321.0101
1332.1312 1332.5527 1335.9045
1341.5078 1343.5626 1345.9812
1348.7208 1356.4852 1361.0999
1362.2313 1363.6062 1366.0832
1368.1528 1377.3589 1387.7533
1393.0638 1395.4167 1401.1156
1408.3508 1416.5857 1428.7587
1485.7712 1489.3734 1497.6463
1501.2609 1501.8228 1503.5480
1505.4016 1508.7072 1514.5175
1516.0534 1517.3667 1521.5810
1522.9644 1523.5613 1523.9386
1538.6331 1542.2426 1554.6753
1563.6716 1638.4534 1640.5250
2102.9110 2977.2338 2981.7569
3023.5208 3027.9338 3032.0731
3048.8714 3060.8053 3065.0167
3067.5997 3073.2678 3079.7051
3086.1138 3087.0352 3088.4290
3091.6804 3095.8107 3107.7609
3112.3280 3115.7482 3119.9097
3123.6443 3124.2262 3140.2303
3141.1292 3153.6972 3155.3080
3155.3656 3159.7603 3171.1102
3184.1588 3186.5535 3194.2401
3201.4641 3210.1171 3223.6852

Post-SuFEx Resting State Complex 18

Gaussian 16: ES64L-G16RevB.01 20-Dec-2017

B3LYP/6-31G(d,p) gfprint gfinput scf=(direct,tight,maxcycle=300,xqc) opt=(maxcycle=250) freq=noraman
N.EDU\12-Jan-2022\0\\# B3LYP/6-31G(d,p) gfprint gfinput scf=(direct,ti
ght,maxcycle=300,xqc) opt=(maxcycle=250) freq=noraman\\ca_2ntf2_1dabco
----
Full point group C1 NOp 1
Stoichiometry C21H36CaF13N7O8S4
Framework group C1[X(C21H36CaF13N7O8S4)]

Num atoms: 90
Charge = 0 Multiplicity = 1

SCF = -5375.16457161 | Predicted change in Energy=-2.021715D-08

Optimization completed.
Maximum Force 0.000037 0.000450 YES
RMS Force 0.000003 0.000300 YES
Maximum Displacement 0.001519 0.001800 YES
RMS Displacement 0.000308 0.001200 YES

Atom Type	Coordinates (in Angstroms) X	Y	Z
S	-0.912243	1.232361	-0.529035
O	-0.430209	2.139639	-1.604122
O	-0.978463	-0.195204	-0.870349
C	0.400221	1.377803	0.790913
F	0.082768	0.643079	1.851688
F	1.548917	0.932212	0.268417
F	0.551725	2.651768	1.155701
N	-2.169656	1.785102	0.309146
S	-3.354211	2.656471	-0.333574
O	-3.927394	3.554217	0.660238
O	-3.051133	3.217560	-1.679704
C	-4.672875	1.388387	-0.716816
F	-5.712463	2.002978	-1.297613
F	-4.180033	0.479798	-1.568510
F	-5.081894	0.782372	0.392190
S	0.492480	5.176804	-6.309113
O	0.544303	6.489813	-6.935450
O	-0.615692	4.917181	-5.350361
C	0.162888	3.953772	-7.685104
F	-1.043770	4.185171	-8.213447
F	1.085851	4.057392	-8.634900
F	0.181536	2.706765	-7.183583
N	1.950853	4.749295	-5.792364
S	2.242899	3.706994	-4.604783
O	1.095713	3.410462	-3.709005
O	3.038767	2.556940	-5.043384
C	3.361900	4.742505	-3.526332
F	2.689667	5.825097	-3.100660
F	4.442606	5.129465	-4.192621
F	3.723866	4.016155	-2.466577
Ca	-1.377135	3.478596	-3.473252
F	-1.450020	1.557700	-4.568328
C	-4.442515	2.623709	-4.710034
C	-5.803520	2.719389	-5.464814
H	-3.747509	1.917793	-5.171562
H	-4.586713	2.313311	-3.673250
H	-5.798678	2.105721	-6.372441
H	-6.627937	2.366306	-4.835614
C	-3.621141	4.424529	-6.094237
H	-3.078601	5.373044	-6.079738
H	-2.992335	3.701387	-6.618881
C	-5.021073	4.563643	-6.768990
H	-5.227286	5.604386	-7.041826
H	-5.077241	3.968915	-7.687336
C	-6.065509	4.957380	-4.654278
H	-6.334164	5.978242	-4.947210
H	-6.843472	4.597167	-3.972589
C	-4.660856	4.916678	-3.973109
H	-4.176500	5.899101	-3.999155
H	-4.723740	4.601118	-2.929142
N	-6.083122	4.110441	-5.857469
N	-3.773143	3.957989	-4.687791
C	2.216617	-1.461933	-3.768550
C	1.344853	-0.184930	-3.575853
H	2.335971	-1.993898	-2.820262
H	3.215116	-1.196625	-4.127568
H	0.941631	-0.098512	-2.567570
H	1.878261	0.732702	-3.824432
C	0.257785	-2.749289	-4.279771
H	-0.168076	-3.467402	-4.987116
H	0.352374	-3.254089	-3.314161
C	-0.650610	-1.490602	-4.153496
H	-1.502752	-1.508489	-4.837432
H	-1.022742	-1.329615	-3.139725
C	0.663497	-0.395746	-5.922732
H	-0.215876	-0.387834	-6.571062
H	1.248663	0.502853	-6.126741
C	1.501624	-1.702690	-6.043573
H	1.045154	-2.394464	-6.758059
H	2.511252	-1.478923	-6.399454
N	0.173308	-0.289351	-4.510102
N	1.603544	-2.379408	-4.742853
H	-0.503878	0.597911	-4.458757
C	-1.381899	6.931465	-2.665139
C	-0.747618	8.204096	-2.088491
C	-0.650563	8.128307	-0.557477
C	0.054406	6.835200	-0.121699
C	-0.610778	5.605347	-0.752585
H	-2.426065	6.855030	-2.334448
H	-1.380935	6.954529	-3.759076
H	-1.330902	9.078637	-2.400344
H	0.256528	8.327494	-2.517987
H	-0.125542	9.005254	-0.162568
H	-1.662356	8.148908	-0.128951
H	1.109637	6.867917	-0.428814
H	0.046522	6.736168	0.970020
H	-1.633460	5.494710	-0.372684
H	-0.070298	4.691171	-0.492768
N	-0.697148	5.686874	-2.233643
H	0.259842	5.729924	-2.588918

Statistical Thermodynamic Analysis
Temperature 298.150 Kelvin. Pressure 1.00000 Atm.

SCF = -5375.16457161 l Predicted change in Energy=-2.021715D-08
Zero-point correction (ZPE) = -5374.5080996100005 0.656472
Internal Energy (U) = -5374.44982661 0.714745
Enthalpy (H) = -5374.44888161 0.71569
Gibbs Free Energy (G) = -5374.61483461 0.549737

Frequencies
5.9923 14.8395 17.1991
19.2519 21.9611 23.3701
25.8277 28.3571 29.3405
30.0469 33.8019 35.0127
36.6717 38.5131 39.1786
42.0001 46.7439 49.3649
51.6831 55.7958 58.3083
61.3733 65.6021 67.1760
73.2569 76.8777 78.6206
83.6103 86.4625 94.3394
97.5779 99.5055 104.6858
105.0482 108.7699 111.7658
115.7710 164.8709 175.0245
181.3717 184.5571 189.8968
196.4831 201.6182 212.7886
218.3435 230.5479 238.0293
246.1192 272.4290 277.3753
280.6078 281.3424 283.8006
291.8358 292.4328 294.7911
314.5361 315.6850 321.9527
324.4844 327.7340 329.1720
332.5699 335.6364 336.6620
341.2946 393.3348 396.5059
405.8704 411.2604 417.5574
420.6505 422.5750 425.1104
426.8680 428.2173 457.1456
460.8588 491.8803 496.3857
511.9790 519.8400 538.1878
538.9656 549.4240 549.6568
555.9792 559.5756 562.2436
563.9579 580.3543 581.5414
581.9665 586.5592 587.8681
589.0021 590.6851 614.0237
615.7660 627.0973 639.1543
721.1709 722.5824 760.1863
761.8624 777.6033 780.6546
784.2478 792.8074 806.1641
815.7033 816.5165 816.9609
819.6784 823.7450 839.5178
841.1566 844.3041 846.9093
857.1604 876.7324 885.4924
902.5723 905.3197 906.4972
910.3777 958.6232 977.7853
981.4764 991.0138 1001.7060
1002.8876 1018.5394 1021.5733
1023.7099 1027.2978 1028.8681
1029.4722 1031.8324 1034.1859
1040.8917 1061.7693 1064.6028
1072.2491 1073.1814 1075.2766
1076.9754 1079.5359 1091.6265
1097.0910 1101.0213 1104.9655
1132.0692 1202.7830 1203.9793
1205.1009 1205.7547 1206.3882
1207.3491 1207.8720 1208.1318
1209.7481 1211.9596 1216.5618
1217.3146 1224.4027 1226.7902
1232.5455 1244.0474 1255.4422
1262.4287 1264.0724 1267.8719
1271.1912 1272.0734 1277.4428
1285.3133 1297.3795 1306.3827
1308.9307 1313.8998 1320.3995
1333.3797 1334.7974 1337.0558
1341.4685 1344.9100 1347.2066
1347.5057 1349.3150 1355.1024
1355.8500 1356.7629 1360.1577
1360.5952 1360.9084 1362.0170
1362.9329 1364.9300 1380.1679
1383.9467 1387.3749 1392.2177
1397.5754 1404.2830 1413.2205
1427.8960 1476.8930 1496.6527
1498.9343 1499.2374 1499.3167
1501.2619 1502.2225 1504.8416
1506.6346 1508.5536 1511.4719
1514.3205 1514.6518 1516.9375
1518.3502 1522.1272 1527.9254
1532.8783 1543.5561 1562.2051
2075.2173 3020.5385 3023.9045
3028.9356 3038.0293 3043.7876
3044.7768 3048.4641 3057.0759
3057.3542 3064.9669 3066.6934
3070.4615 3076.3864 3078.8109
3081.6340 3083.5221 3086.8574
3088.5253 3089.7986 3090.8570
3092.4340 3093.0985 3103.5205
3111.1360 3113.7117 3116.6721
3118.6280 3123.4546 3127.1120
3134.7602 3145.9790 3150.5225

3153.2379 3186.3920 3474.5754

Transition State Complex 19

Gaussian 16: ES64L-G16RevB.01 20-Dec-2017

B3LYP/6-31G(d,p) gfprint gfinput scf=(direct,tight,maxcycle=300,xqc) opt=(maxcycle=250,ts,calcfc,noeigentest) freq=noraman N.EDU\10-Jun-2021\0\\# B3LYP/6-31G(d,p) gfprint gfinput scf=(direct,ti ght,maxcycle=300,xqc) opt=(maxcycle=250,ts,calcfc,noeigentest) freq=no

----

Full point group C1 NOp 1
Stoichiometry C20H25CaF13N4O11S5
Framework group C1[X(C20H25CaF13N4O11S5)]

Num atoms: 79
Charge = 0 Multiplicity = 1

SCF = -5789.89435321 | Predicted change in Energy=-2.330782D-09

Optimization completed.
Maximum Force 0.000002 0.000450 YES
RMS Force 0.000000 0.000300 YES
Maximum Displacement 0.001558 0.001800 YES
RMS Displacement 0.000186 0.001200 YES

Atom Type	Coordinates (in Angstroms) X	Y	Z
S	1.175290	3.778793	0.656707
O	1.779884	4.764858	-0.260160
F	0.721222	1.824363	-0.180018
O	1.292545	3.794657	2.124906
C	-3.308322	4.288015	-0.086586
C	-2.866198	3.661457	1.079889
C	-1.500744	3.476951	1.302412
C	-0.605002	3.931489	0.338194
C	-1.021306	4.557066	-0.836964
C	-2.389566	4.738290	-1.038568
H	-4.371911	4.425797	-0.254465
H	-3.574567	3.291243	1.813301
H	-1.147083	2.989342	2.201652
H	-0.293809	4.913339	-1.555473
H	-2.734666	5.237642	-1.938684
Ca	-0.552726	-0.020622	-0.430253
S	-3.914077	-1.471404	-0.474896
O	-2.472241	-1.376654	-0.859357
O	-4.610364	-2.704635	-0.801725
C	-4.692506	-0.129581	-1.519029
F	-4.125828	1.050516	-1.213378
F	-5.999890	-0.058485	-1.301090
F	-4.472574	-0.388549	-2.814000
N	-4.260513	-0.993879	1.017632
S	-3.304010	-0.138047	1.984671
O	-4.077342	0.804110	2.785411
O	-2.070324	0.400435	1.337487
C	-2.643794	-1.431088	3.166100
F	-3.634563	-1.917612	3.906516
F	-2.074947	-2.416575	2.469873
F	-1.732357	-0.861278	3.958508
S	2.449797	-1.921610	-1.453285
O	1.159633	-1.197133	-1.667396
O	3.662380	-1.281193	-1.958252
C	2.217558	-3.470233	-2.480878
F	1.134487	-4.122681	-2.064180
F	2.055633	-3.112516	-3.760967
F	3.287141	-4.251143	-2.372225
N	2.678464	-2.414237	0.053678
S	1.432083	-2.623954	1.077092
O	0.746866	-3.909558	0.990423
O	0.576683	-1.402959	1.185090
C	2.406629	-2.623337	2.672452
F	3.320932	-3.585764	2.655242
F	1.546933	-2.834132	3.667062
F	3.004638	-1.440001	2.854244
H	2.896865	1.734886	2.433954
H	2.361612	0.753315	1.063406
N	3.152482	2.620176	0.562787
C	3.145377	1.416461	1.419340
C	4.570767	0.742557	1.334633
C	5.553756	2.900377	1.127571
C	4.138527	3.591912	1.096414
C	3.512537	2.264444	-0.825705
C	4.973077	1.671871	-0.827282
H	4.986262	0.613384	2.337852

H 4.489548 -0.242353 0.870855
H 6.276635 3.501474 0.568028
H 5.912281 2.816250 2.157685
H 3.808027 3.889940 2.092494
H 4.125744 4.468259 0.447729
H 2.781299 1.539131 -1.176388
H 3.433651 3.171589 -1.429908
H 4.961400 0.680701 -1.284849
H 5.647261 2.313392 -1.402531
N 5.483710 1.566117 0.541118
C -1.427860 -0.125887 -3.791409
C -0.205870 -0.242565 -4.716124
C 0.634889 1.026035 -4.398573
C -0.190935 1.763586 -3.333083
H -2.280994 0.341020 -4.298698
H -1.756027 -1.064799 -3.345750
H -0.509850 -0.291643 -5.764826
H 0.365382 -1.143696 -4.487341
H 1.611551 0.742876 -3.999735
H 0.798802 1.653844 -5.278260
H 0.393880 2.227781 -2.539200
H -0.853310 2.515616 -3.782678
O -1.006045 0.744444 -2.713243

Statistical Thermodynamic Analysis
Temperature 298.150 Kelvin. Pressure 1.00000 Atm.

SCF = -5789.89435321 | Predicted change in Energy=-2.330782D-09
Zero-point correction (ZPE) = -5789.37571221 0.518641
Internal Energy (U) = -5789.31916721 0.575186
Enthalpy (H) = -5789.31822221 0.576131
Gibbs Free Energy (G) = -5789.47721621 0.417137

Frequencies
-69.4811 10.0022 11.0209
19.1989 22.5832 25.2874
26.8359 27.1492 29.0680
32.5698 35.5264 39.7059
41.1137 42.7248 46.8153
48.7752 50.4070 52.8008
53.6404 59.2534 61.0380
66.0348 70.4215 74.1828
79.5108 84.8748 88.3210
93.7466 98.3206 102.6721
106.9533 109.8400 120.5466
131.4904 135.1422 155.8734
162.3693 167.2337 178.3727
182.4447 196.3677 200.0516
202.1875 203.4967 207.2049
210.9305 214.3370 231.7099
244.0787 258.8756 268.9584
270.9156 277.3026 283.3950
288.3928 294.7432 298.4012
309.3677 313.0735 316.5619
320.6339 324.3641 325.1938
327.8877 332.6468 341.2667
355.0659 357.0379 360.8751
395.8010 397.0323 400.6736
406.8608 416.0723 434.8939
443.5570 445.7727 450.7244
492.7018 501.6117 504.4971
517.9119 523.4303 536.4451
537.7799 537.9825 548.3158
550.2894 554.7000 562.0619
564.8744 574.9010 577.6031
581.4404 590.9660 593.3060
611.5613 620.5107 644.6148
645.4143 647.1357 651.5945
684.6137 699.8580 719.5120
720.5344 760.3910 762.7247
764.7591 779.4976 781.5959
786.0208 809.4135 810.0048
832.6424 848.6631 856.2939
861.3728 868.4961 893.9343
899.7907 908.5471 925.2038
926.3807 936.3459 949.0126
971.9928 984.5472 997.7536
1003.2927 1005.4069 1012.3179
1020.2806 1033.4790 1040.0364
1041.6218 1049.2422 1055.2179
1056.2998 1062.8143 1064.2249
1066.8859 1073.0308 1078.5451
1083.9642 1084.8498 1096.9636
1108.8806 1156.5503 1191.3350
1194.9534 1197.7287 1203.3655
1204.8269 1207.0732 1207.6436
1209.7490 1210.5249 1222.1257
1230.8995 1240.4695 1241.8699
1245.7514 1250.6955 1252.5300
1263.3301 1264.8070 1267.1941
1271.6800 1274.6586 1288.2294
1290.6976 1291.7992 1295.0613
1296.1694 1311.3262 1318.1756

1318.5522 1330.6207 1332.0276
1336.4167 1336.9188 1339.8795
1355.0625 1358.7812 1359.5566
1360.9177 1365.2101 1367.8634
1378.3834 1394.9707 1408.8167
1487.1127 1501.2373 1502.7897
1505.6903 1512.4577 1518.9342
1520.0908 1522.1324 1524.3988
1525.8175 1542.6637 1547.1744
1626.0660 1639.4646 3029.2850
3047.9724 3064.1869 3073.1602
3078.8107 3082.1484 3092.9916
3096.5289 3104.7537 3108.7445
3113.3052 3125.7238 3139.0800
3142.3339 3145.1691 3157.1487
3160.8646 3167.3733 3182.6674
3192.9527 3199.4548 3207.2725
3220.4077 3237.8324 3246.2821

DABCO-HF Adduct 20

Gaussian 16: ES64L-G16RevB.01 20-Dec-2017

B3LYP/6-31G(d,p) gfprint gfinput scf=(direct,tight,maxcycle=300,xqc) opt=(maxcycle=250) freq=noraman Mar-2021\0\\# B3LYP/6-31G(d,p) gfprint gfinput scf=(direct,tight,maxcy cle=300,xqc) opt=(maxcycle=250) freq=noraman\\999_dabcoh_f.xyz\\0,1\C,
----
Full point group C1 NOp 1
Stoichiometry C6H13FN2 Framework group C1[X(C6H13FN2)]

Num atoms: 22
Charge = 0 Multiplicity = 1

SCF = -445.797533704 l Predicted change in Energy=-1.194066D-08

Optimization completed.
Maximum Force 0.000012 0.000450 YES
RMS Force 0.000002 0.000300 YES
Maximum Displacement 0.001651 0.001800 YES
RMS Displacement 0.000189 0.001200 YES

Atom Coordinates (in Angstroms)
Type X Y Z

C 0.192058 0.952021 -0.098051
C -0.380833 2.398674 0.011132
H -0.145174 0.446254 -1.008734
H -0.085155 0.317732 0.748222
H -1.031601 2.634411 -0.837815
H -0.974695 2.524219 0.922777
C 2.101185 1.801093 -1.308798
H 3.195747 1.807619 -1.334325
H 1.752300 1.289160 -2.211543
C 1.512013 3.246422 -1.193464
H 2.308392 3.998042 -1.171062
H 0.865762 3.479896 -2.046403
C 1.577769 3.101328 1.199714
H 2.372112 3.855018 1.227076
H 0.978199 3.225441 2.107813
C 2.166417 1.660284 1.101059
H 3.260550 1.667286 1.060060
H 1.876242 1.021872 1.939848
N 1.674620 1.006493 -0.137189
N 0.715928 3.382563 0.037882
H 2.014443 -0.561374 0.230941
F 1.980721 -1.364451 0.762858

Statistical Thermodynamic Analysis
Temperature 298.150 Kelvin. Pressure 1.00000 Atm.

SCF = -445.797533704 l Predicted change in Energy=-1.194066D-08
Zero-point correction (ZPE) = -445.600273704 0.19726
Internal Energy (U) = -445.591827704 0.205706
Enthalpy (H) = -445.590882704 0.206651
Gibbs Free Energy (G) = -445.634211704 0.163322

Frequencies
65.5497 78.4302 92.5364
222.3577 336.7530 338.2481
425.5597 429.7381 586.1627
590.1778 627.5743 782.0043

810.3067 814.3886 835.0006
835.9349 888.8500 893.5522
973.6663 982.6813 995.7463
1026.5837 1032.8167 1039.0759
1058.4295 1075.0963 1082.3969
1195.8718 1207.7462 1210.9285
1264.7766 1330.7218 1332.9423
1335.2517 1339.6710 1348.6621
1352.8845 1357.5173 1357.8997
1375.7059 1394.5128 1497.2765
1498.7527 1507.9126 1510.7312
1512.0433 1525.2450 3046.3566
3047.2834 3050.4123 3056.0797
3063.0432 3064.9829 3087.4186
3090.9821 3093.0775 3110.1105
3119.7463 3123.0769 3257.9447

Single Point Energies in ωB97xd/def2-TZVP/PCM(THF)

Structure	E (Hartrees)
**1**	-880.1713
**2**	-251.9250
**DABCO**	-345.3540
**Sulfonamide product**	-1067.3173
**4a**	-4797.6570
**4b**	-5030.1535
**4c**	-5262.6411
**5a**	-5445.3541
**5b**	-5677.8490
**5c**	-5910.3377
**6a**	-4910.5472
**6b**	-5143.0382
**6c**	-5375.5231
**7a**	-4817.1166
**7b**	-5049.6126
**7c**	-5282.1003
**8a**	-5558.2425
**8b**	-5790.7369
**8c**	-6023.2215
**9a**	-5464.8128
**9b**	-5697.3082
**9c**	-5929.7931
**10a**	-4930.0063
**10b**	-5162.4992
**10c**	-5394.9822
**11a**	-5810.1980
**11b**	-6042.6772

Structure	E (Hartrees)
**12**	-5255.9288
**13**	-5069.0709
**14**	-5088.5287
**15**	-6275.1485
**Transition State Complex 16**	-5810.1713
**17**	-6042.7212
**18**	-5375.8975
**Transition State Complex 19**	-5790.6700
**20**	-445.8444

Determining Relative Stability of Ca^{2+} Complexes

To compare the relative stabilities of the ground state Ca^{2+} complexes, we modeled chemical equilibria in which the geometries and energies of non-coordinating ligands were computed separately (i.e., not interacting with the calcium complex or with other species).

For example, to determine the relative stability of $[Ca(NTf_2)_2(THF)_2]$ compared to $[Ca(NTf_2)_2(THF)_4]$, we utilized the equilibrium as seen in eq 1., where each THF molecule on the right side of the equilibrium is evaluated separately from each other and from the Ca^{2+} center. Consequently, the computed thermodynamic quantities for each equilibrium directly correlate to the relative tendency of each species in the reaction to coordinate to the Ca^{2+} center.

$$[Ca(NTf_2)_2(THF)_4] \rightleftharpoons [Ca(NTf_2)_2(THF)_2] + 2\ THF \qquad \text{eq 1}$$

Enthalpic and Entropic Contributions To Ground State $Ca(NTf_2)_2$ Complexes

To understand the energetic origin of this preference for 7-coordinate Ca^{2+} species, we disaggregated the overall thermodynamic stability into enthalpic and entropic contributions. We find that adding THF yielded more enthalpically stabilizing Ca^{2+}. In contrast, increased coordination around Ca^{2+} incurred greater entropic cost. The entropic cost overrides the enthalpic gain from 7- to 8-coordinate $Ca(NTf_2)_2$ species (leading to 7-coordinate species being the most thermodynamically stable. Thus, we observe effects of the so-called "entropy-enthalpy compensation" invoked in protein-ligand association studies to the relative thermodynamic stability of 6-, 7-, and 8- coordinate $Ca(NTf_2)_2$ complexes.

Table S1. Enthalpic and entropic contributions to the relative thermodynamic stability of 6-, 7-, and 8- coordinate $Ca(NTf_2)_2$.

Structure	Coordination #	ΔΔG[a]	ΔΔH[a]	TΔΔS[a]
$Ca(NTf_2)_2(THF)_2$ **4a**	6	2.0	23.6	21.6
$Ca(NTf_2)_2(THF)_3$ **4b**	7	0.0	8.9	8.9
$Ca(NTf_2)_2(THF)_4$ **4c**	8	0.6	0.0	-0.6

Table S2. Enthalpic and entropic contributions to the relative thermodynamic stability of 6-, 7-, and 8- coordinate $Ca(NTf_2)_2(PhSO_2F)$

Structure	Coordination #	ΔΔG[a]	ΔΔH[a]	TΔΔS[a]
$Ca(NTf_2)_2(PhSO_2F)(THF)$ **5a**	6	4.2	23.6	19.5
$Ca(NTf_2)_2(PhSO_2F)(THF)_2$ **5b**	7	0.0	9.9	9.9
$Ca(NTf_2)_2(PhSO_2F)(THF)_3$ **5c**	8	2.2	0.0	-2.2

Table S3. Enthalpic and entropic contributions to the relative thermodynamic stability of 6-, 7-, and 8- coordinate $Ca(NTf_2)_2(DABCO)$

Structure	Coordination #	ΔΔG[a]	ΔΔH[a]	TΔΔS[a]
$Ca(NTf_2)_2(DABCO)(THF)$ **6a**	6	2.9	18.8	15.9
$Ca(NTf_2)_2(DABCO)(THF)_2$ **6b**	7	0.0	7.4	7.4
$Ca(NTf_2)_2(DABCO)(THF)_3$ **6c**	8	5.4	0.0	-5.4

Table S4. Enthalpic and entropic contributions to the relative thermodynamic stability of 6-, 7-, and 8- coordinate $Ca(NTf_2)_2(Pip)$

Structure	Coordination #	ΔΔG[a]	ΔΔH[a]	TΔΔS[a]
$Ca(NTf_2)_2(Pip)(THF)$ **7a**	6	3.5	23.5	20.0
$Ca(NTf_2)_2(Pip)(THF)_2$ **7b**	7	0.0	9.1	9.1
$Ca(NTf_2)_2(Pip)(THF)_3$ **7c**	8	1.1	0.0	-1.1

Table S5. Enthalpic and entropic contributions to the relative thermodynamic stability of 6-, 7-, and 8- coordinate $Ca(NTf_2)_2(PhSO_2F)(DABCO)$

Structure	Coordination #	ΔΔG[a]	ΔΔH[a]	TΔΔS[a]
$Ca(NTf_2)_2(PhSO_2F)(DABCO)$ **8a**	6	2.7	20.7	18.0
$Ca(NTf_2)_2(PhSO_2F)(DABCO)(THF)$ **8b**	7	0.0	7.4	7.4
$Ca(NTf_2)_2(PhSO_2F)(DABCO)(THF)_2$ **8c**	8	4.5	0.0	-4.5

Table S6. Enthalpic and entropic contributions to the relative thermodynamic stability of 6-, 7-, and 8- coordinate $Ca(NTf_2)_2(PhSO_2F)(Pip)$

Structure	Coordination #	ΔΔG[a]	ΔΔH[a]	TΔΔS[a]
$Ca(NTf_2)_2(PhSO_2F)(Pip)$ **9a**	6	4.0	21.4	17.3
$Ca(NTf_2)_2(PhSO_2F)(Pip)(THF)$ **9b**	7	0.0	7.4	7.4
$Ca(NTf_2)_2(PhSO_2F)(Pip)(THF)_2$ **9c**	8	1.9	0.0	-1.9

Table S7. Enthalpic and entropic contributions to the relative thermodynamic stability of 6-, 7-, and 8- coordinate $Ca(NTf_2)_2(DABCO)(Pip)$

Structure	Coordination #	ΔΔG[a]	ΔΔH[a]	TΔΔS[a]
$Ca(NTf_2)_2(DABCO)(Pip)$ **10a**	6	2.5	18.9	16.4
$Ca(NTf_2)_2(DABCO)(Pip)(THF)$ **10b**	7	0.0	6.3	6.3

Structure	Coordination #	ΔΔG[a]	ΔΔH[a]	TΔΔS[a]
$Ca(NTf_2)_2(DABCO)(Pip)(THF)_2$ **10c**	8	3.7	0.0	-3.7

Table S8. Enthalpic and entropic contributions to the relative thermodynamic stability of 7- and 8-coordinate $Ca(NTf_2)_2(PhSO_2F)(DABCO)(Pip)$

Structure	Coordination #	ΔΔG[a]	ΔΔH[a]	TΔΔS[a]
$Ca(NTf_2)_2(PhSO_2F)(DABCO)(Pip)$ **11a**	7	0.0	4.0	4.0
$Ca(NTf_2)_2(PhSO_2F)(DABCO)(Pip)(THF)$ **11b**	8	4.5	0.0	-4.5

Estimated Gibbs Free Energy Barriers

Table S9. Estimated Gibbs free energy barriers from reaction yield and time data for $Ca(NTf_2)_2$ and DABCO mediated nucleophilic substitution of sulfonyl fluorides with amines to sulfonamides.

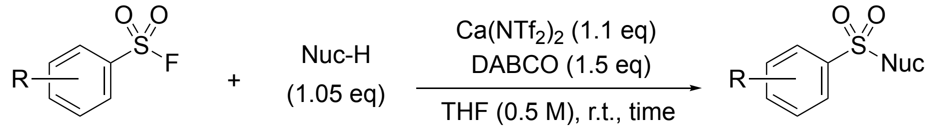

Sulfur(VI) fluoride	Nuc-H	Yield (%)	Time (h)	Est. $\Delta G^{\ddagger}$ (kcal/mol)
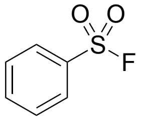	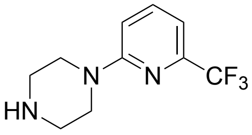	94	0.5	21.3
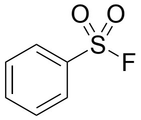	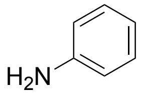	94	3	22.3
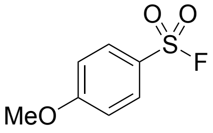	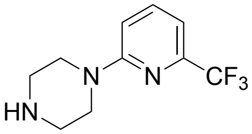	93	0.5	21.3
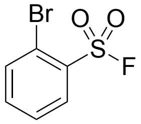	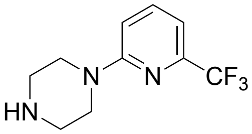	79	1	22.0
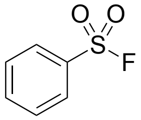	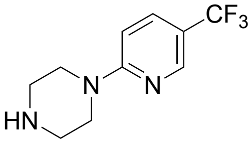	82	0.5	21.6
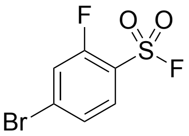	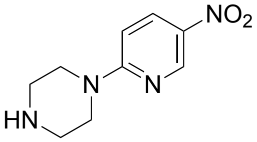	89	0.75	21.7
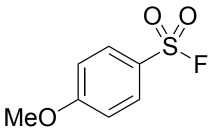	NH_3	74	1	22.1
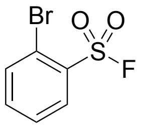	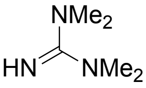	64	1	22.3

DABCO-As-Bronsted-Base Transition States Within 3.0 kcal/mol

Shown below are the geometries of the transition state conformational isomers within 3.0 kcal/mol of the lowest energy complex **16.** The bond-forming/breaking distances are conserved across the isomers.

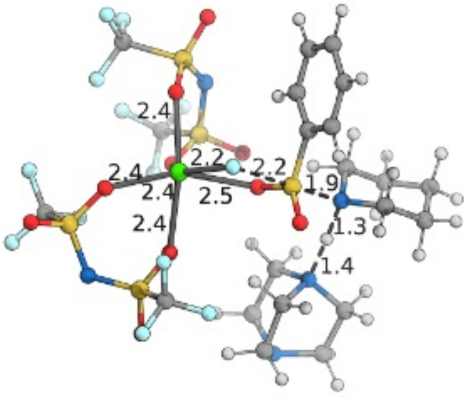

16 ΔΔG‡ = 0.0 kcal/mol

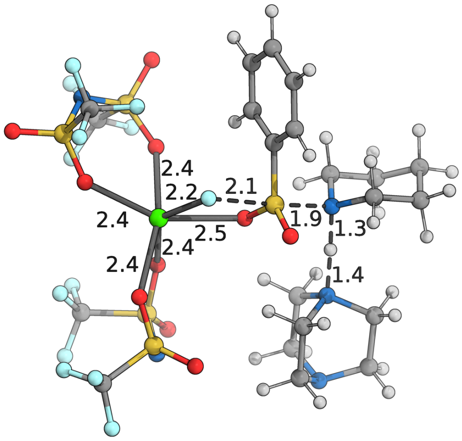

S1 ΔΔG‡ = +1.9 kcal/mol

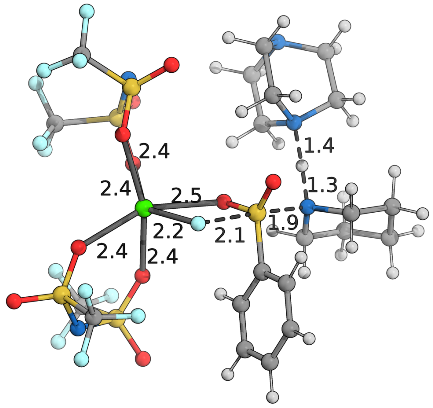

S2 ΔΔG‡ = +2.1 kcal/mol

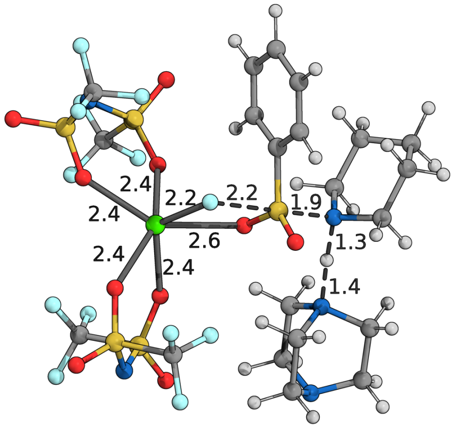

S3 ΔΔG‡ = +2.4 kcal/mol

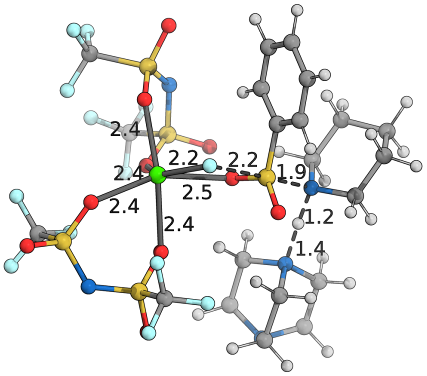

S4 ΔΔG‡ = +3.0 kcal/mol

Calculation Parameters, Geometries, Energies, and Vibrational Frequencies

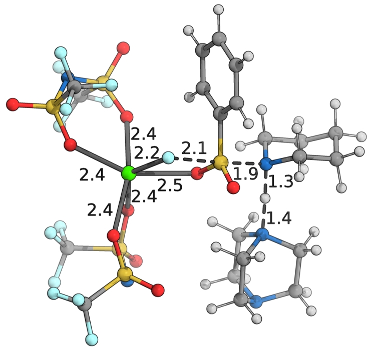

S1 ΔΔG‡ = +1.9 kcal/mol

Gaussian 16: ES64L-G16RevB.01 20-Dec-2017

B3LYP/6-31G(d,p) gfprint gfinput scf=(direct,tight,maxcycle=300,xqc) opt=(maxcycle=250,ts,calcfc,noeigentest) freq=noraman
N.EDU\12-Feb-2021\0\\# B3LYP/6-31G(d,p) gfprint gfinput scf=(direct,ti ght,maxcycle=300,xqc) opt=(maxcycle=250,ts,calcfc,noeigentest) freq=no

----

Full point group C1 NOp 1
Stoichiometry C21H28CaF13N5O10S5
Framework group C1[X(C21H28CaF13N5O10S5)]

Num atoms: 83
Charge = 0 Multiplicity = 1

SCF = -5809.36840413 l Predicted change in Energy=-9.532459D-10

Optimization completed.
Maximum Force 0.000013 0.000450 YES
RMS Force 0.000001 0.000300 YES
Maximum Displacement 0.000835 0.001800 YES
RMS Displacement 0.000139 0.001200 YES

Atom Type	Coordinates (in Angstroms) X	Y	Z
Ca	1.017977	0.248800	0.916229
S	2.317182	-2.887130	-0.384599
O	1.926300	-4.254030	-0.710576
O	1.216632	-1.960889	0.021042
C	2.924132	-2.123847	-1.980720
F	3.375926	-0.888127	-1.732862
F	3.896290	-2.855810	-2.511807
F	1.903038	-2.048840	-2.845060
N	3.613164	-2.870882	0.557460
S	3.975232	-1.722048	1.631960
O	3.208152	-0.448933	1.470917
O	5.415604	-1.616791	1.794234
C	3.290592	-2.409788	3.231676
F	1.973805	-2.613374	3.087581
F	3.886694	-3.557134	3.537304
F	3.493486	-1.524346	4.207735
S	0.061166	2.099348	-1.989757
O	-1.269199	1.682502	-2.436885
O	0.940330	1.058258	-1.384834
C	0.963588	2.588048	-3.556178
F	2.156863	3.104982	-3.258808
F	1.127584	1.498073	-4.307745
F	0.244359	3.485923	-4.224739
N	-0.040423	3.474727	-1.155226
S	0.777728	3.824182	0.192439
O	1.102176	2.632286	1.025526
O	0.120868	4.947244	0.848842
C	2.471922	4.440747	-0.327327
F	2.350687	5.339370	-1.302619
F	3.066594	4.998226	0.724984
F	3.207116	3.411462	-0.759071
S	-2.222562	-0.289954	1.539911
O	-1.363864	-0.026851	0.351609
F	-0.366532	0.018792	2.613172
O	-2.353761	-1.558715	2.255402
C	-3.185564	3.391357	4.036505
C	-3.439050	2.114331	4.542907
C	-3.149562	0.983530	3.779580
C	-2.618736	1.167544	2.503109
C	-2.357933	2.432433	1.976461
C	-2.642518	3.550373	2.760042
H	-3.399563	4.264960	4.644224
H	-3.845693	1.993591	5.541963

H	-3.294882	-0.016224	4.172181
H	-1.925126	2.549764	0.990745
H	-2.412083	4.536206	2.370667
C	-5.308671	0.561311	-1.232683
C	-4.066997	0.754987	-0.356073
C	-4.983054	-0.803360	1.334610
C	-6.249784	-1.003852	0.494996
H	-5.124783	-0.227085	-1.973251
H	-5.465984	1.485421	-1.798738
H	-4.227782	1.614481	0.304640
H	-3.178384	0.946203	-0.959457
H	-4.728382	-1.704071	1.894661
H	-5.147589	0.009012	2.053733
H	-6.144229	-1.899672	-0.127648
H	-7.081342	-1.198253	1.181279
N	-3.802113	-0.450468	0.491947
H	-3.443004	-1.417613	-0.260800
N	-2.990218	-2.430315	-1.092482
C	-4.132751	-3.217487	-1.645186
C	-3.591692	-4.272634	-2.660388
C	-1.651238	-4.528638	-1.287609
C	-2.082793	-3.366428	-0.343495
C	-2.220222	-1.829265	-2.232556
C	-1.633941	-2.975629	-3.112106
H	-4.645586	-3.689662	-0.801410
H	-4.831067	-2.520286	-2.117914
H	-3.954171	-5.273165	-2.404149
H	-3.936293	-4.047145	-3.674747
H	-2.064145	-5.484539	-0.949503
H	-0.562457	-4.623668	-1.298912
H	-2.620696	-3.715872	0.541073
H	-1.223800	-2.788257	-0.003887
H	-2.909182	-1.191991	-2.794970
H	-1.446494	-1.189501	-1.809226
H	-1.911681	-2.839351	-4.162248
H	-0.542639	-2.983160	-3.056679
N	-2.126759	-4.285411	-2.659698
C	-6.541101	0.213750	-0.390134
H	-6.802421	1.072248	0.242948
H	-7.406693	0.021016	-1.032526

Statistical Thermodynamic Analysis
Temperature 298.150 Kelvin. Pressure
1.00000 Atm.

SCF = -5809.36840413 l Predicted change in Energy=-9.532459D-10
Zero-point correction (ZPE) = -5808.81014913 0.558255
Internal Energy (U) = -5808.75338413 0.61502
Enthalpy (H) = -5808.75244013 0.615964
Gibbs Free Energy (G) = -5808.91006513 0.458339

Frequencies
-1054.9493 12.0004 13.4204
15.3206 16.8944 22.7487
28.9439 31.2611 32.0710
33.9253 35.4393 37.1242
38.4913 42.7205 43.8409
46.9300 49.0409 54.9007
56.8160 65.6363 68.4144
80.1580 84.7905 86.5303
90.8363 99.7986 102.9897
105.4565 108.4780 114.7474
120.0156 125.6212 134.0652
154.0118 165.2495 171.5069
184.7900 186.0323 195.7236
197.8856 200.6210 203.1260
205.0547 209.9436 215.5960
228.6969 238.1136 242.5205
252.0933 266.1467 270.2724
278.1024 279.8447 281.9541
283.1388 288.4429 295.7865
297.2977 305.9123 308.8114
312.9695 325.5705 327.3362
331.3085 332.2717 336.2168
339.2625 349.7960 363.3439
391.2142 394.6036 395.8376
411.2950 416.7678 424.5839
427.5609 454.4728 455.9299
461.2340 466.4098 493.4631
494.5977 498.2447 503.5767
519.1921 522.5861 537.2787
539.6338 547.7614 548.9104
556.6863 561.2991 565.4711
567.5748 570.0102 578.3657
581.8430 582.1256 584.1069
595.8123 607.0502 622.7598
626.9009 632.9834 678.2470
693.7584 713.5759 715.3723
718.9507 760.9706 762.3861
763.1877 781.0393 782.5516
797.5333 804.6309 815.7797
819.9205 834.6914 843.1069
847.2651 863.3025 872.3924
903.2040 908.1609 911.8594

916.6403 945.9158 978.0824
980.7294 990.7058 1004.1325
1004.9651 1014.6869 1015.5988
1015.8923 1024.1234 1027.2828
1031.4967 1035.5177 1041.5864
1045.7068 1055.8119 1067.5000
1069.1032 1073.2283 1077.5795
1080.0140 1085.1951 1097.5507
1098.5641 1108.5875 1113.5677
1178.3933 1185.4291 1191.8942
1198.8486 1200.8531 1205.9832
1207.1543 1208.6191 1209.2777
1210.4318 1211.5713 1228.9281
1229.4950 1241.3867 1242.9343
1252.7653 1260.1963 1263.6533
1269.3609 1270.7450 1274.0215
1283.1859 1287.7183 1292.8206
1293.6055 1307.8233 1316.8616
1321.6141 1323.6100 1332.9983
1341.3005 1344.6868 1349.0420
1352.9058 1357.1616 1359.2168
1359.7036 1363.6543 1364.5407
1368.0384 1389.5212 1391.2332
1394.7097 1400.0592 1414.9406
1424.2660 1488.4216 1493.6401
1499.4034 1500.7321 1501.2153
1508.5225 1511.0076 1512.7411
1515.8454 1517.5632 1519.5004
1530.5286 1537.0465 1634.3088
1641.1990 1671.0881 1692.1113
3035.6756 3045.8252 3047.1264
3053.7700 3056.9377 3060.3813
3061.4378 3064.5109 3066.5304
3080.3983 3090.0297 3094.7202
3096.3952 3097.3508 3102.2486
3118.0555 3123.5060 3126.4464
3143.9424 3150.9619 3165.1983
3169.1968 3195.3409 3208.7668
3220.1659 3227.9861 3236.8682

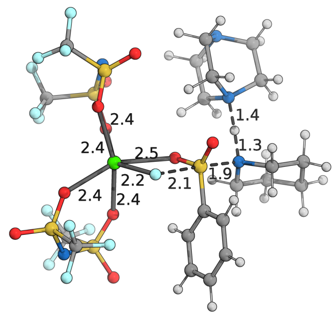

S2 ΔΔG‡ = +2.1 kcal/mol

Gaussian 16: ES64L-G16RevB.01 20-Dec-2017

B3LYP/6-31G(d,p) gfprint gfinput scf=(direct,tight,maxcycle=300,xqc) opt=(maxcycle=250,ts,calcfc,noeigentest) freq=noraman
N.EDU\11-Feb-2021\0\\# B3LYP/6-31G(d,p) gfprint gfinput scf=(direct,ti ght,maxcycle=300,xqc) opt=(maxcycle=250,ts,calcfc,noeigentest) freq=no

----

Full point group C1 NOp 1
Stoichiometry C21H28CaF13N5O10S5
Framework group C1[X(C21H28CaF13N5O10S5)]

Num atoms: 83
Charge = 0 Multiplicity = 1

SCF = -5809.36646312 l Predicted change in Energy=-9.601922D-11

Optimization completed.
Maximum Force 0.000002 0.000450 YES
RMS Force 0.000000 0.000300 YES
Maximum Displacement 0.000240 0.001800 YES
RMS Displacement 0.000047 0.001200 YES

Atom Type	X	Y	Z
Ca	-0.887088	-0.297727	-0.532399
S	1.120627	-3.135467	-1.507812
O	-0.065313	-2.242754	-1.654131
O	2.330644	-2.766595	-2.239490
C	0.543820	-4.756533	-2.244431
F	1.544264	-5.633359	-2.228729
F	-0.481144	-5.235557	-1.536903
F	0.156278	-4.539117	-3.499759
N	1.490230	-3.535382	0.012266

S	0.717139	-3.025164	1.328061
O	0.078846	-1.685903	1.173834
O	1.582019	-3.221491	2.484895
C	-0.755016	-4.160605	1.572558
F	-1.296345	-3.916610	2.764605
F	-1.660224	-3.918589	0.616701
F	-0.370168	-5.432468	1.506139
S	-4.505885	0.021074	-0.650081
O	-5.718225	-0.766526	-0.801896
O	-3.202968	-0.679949	-0.852931
C	-4.500502	1.290474	-2.022728
F	-4.364786	0.669484	-3.194598
F	-5.630541	1.990437	-2.016184
F	-3.461575	2.118842	-1.840402
N	-4.586278	0.941516	0.668317
S	-3.365954	1.383257	1.605540
O	-3.517402	2.760652	2.065674
O	-2.005218	1.001570	1.119645
C	-3.583546	0.290032	3.105137
F	-4.748469	0.538091	3.694807
F	-3.536624	-0.990361	2.723149
F	-2.588961	0.530504	3.963450
S	1.428836	2.087639	-0.995039
O	1.111521	1.076678	0.056123
F	-0.265290	1.265568	-1.968200
O	2.205630	1.848703	-2.211133
C	-1.361810	5.683576	-1.211209
C	-0.416051	5.466157	-2.215786
C	0.441165	4.367185	-2.151152
C	0.334596	3.510970	-1.055234
C	-0.605672	3.703112	-0.043644
C	-1.461482	4.801398	-0.133353
H	-2.034068	6.533299	-1.277634
H	-0.353993	6.141064	-3.063607
H	1.148385	4.156168	-2.944962
H	-0.708474	2.994331	0.768698
H	-2.217623	4.938858	0.632601
C	3.584166	3.885198	2.195240
C	2.415639	3.327515	1.375030
C	3.563223	3.924493	-0.729010
C	4.741707	4.513670	0.054958
H	4.289755	3.080782	2.438118
H	3.185866	4.247436	3.149118
H	1.666249	4.115522	1.238316
H	1.935786	2.488092	1.880019
H	3.889038	3.494079	-1.676937
H	2.842461	4.721820	-0.948590
H	5.533925	3.763660	0.158373
H	5.164233	5.332917	-0.537064
N	2.859704	2.849610	0.030969
H	3.600270	1.807358	0.177675
N	4.338596	0.665424	0.363129
C	3.826554	-0.060184	1.573609
C	4.631769	-1.383629	1.762167
C	6.572909	-0.336950	0.833935
C	5.783802	0.984164	0.567469
C	4.215563	-0.239385	-0.830629
C	5.064030	-1.524417	-0.591343
H	3.939291	0.612642	2.429948
H	2.760815	-0.237216	1.421849
H	5.100047	-1.413513	2.751057
H	3.970177	-2.249381	1.685866
H	7.025944	-0.321927	1.830373
H	7.381418	-0.454546	0.105566
H	5.861766	1.680662	1.407795
H	6.138384	1.497560	-0.331546
H	3.160669	-0.472208	-0.965327
H	4.537963	0.325794	-1.708373
H	4.432747	-2.411307	-0.673333
H	5.858451	-1.612174	-1.339149
N	5.684613	-1.497176	0.742267
C	4.308175	5.005929	1.440666
H	3.632744	5.864724	1.330029
H	5.174243	5.357477	2.010989

Statistical Thermodynamic Analysis
Temperature 298.150 Kelvin. Pressure 1.00000 Atm.

SCF = -5809.36646312 l Predicted change in Energy=-9.601922D-11
Zero-point correction (ZPE) = -5808.80845712 0.558006
Internal Energy (U) = -5808.75150912 0.614954
Enthalpy (H) = -5808.75056512 0.615898
Gibbs Free Energy (G) = -5808.91062412 0.455839

Frequencies
-1082.9870 8.2918 11.2840
13.7261 15.5271 16.6262
20.6161 25.2541 28.4059
31.9639 33.4045 34.5305
37.7385 40.3118 41.2899
45.4662 48.7678 54.2457
55.7336 57.3088 67.8721
78.6352 81.8878 84.5889

86.4031 92.2521 101.2563
105.9064 110.3392 115.0635
118.3882 125.5019 133.4046
154.5569 169.0021 178.3965
179.5808 188.7105 195.1593
199.3115 202.3984 203.6178
205.0269 206.1082 210.0019
225.3011 235.4838 242.3035
260.8171 264.8245 270.2336
270.9175 277.1241 279.6410
282.8774 286.5452 295.6211
297.8370 306.0030 311.4657
313.3838 324.5473 325.6326
326.2099 330.6759 335.5756
341.1223 348.8799 358.6129
391.2115 396.9725 399.3583
415.7032 418.1132 423.0413
425.5402 451.7451 455.2756
462.6352 464.8396 490.2403
496.0307 498.6607 503.1980
518.3593 523.0478 538.7345
538.8410 548.9528 549.1322
556.5260 561.2796 564.5469
567.3736 569.0096 574.0919
582.0175 583.4268 583.7686
592.7844 612.9917 623.1022
628.4738 632.8936 674.9739
693.8665 709.7745 716.6943
719.2164 762.2057 763.3358
764.6201 782.5698 783.2418
798.1177 805.4701 815.3891
821.8225 832.8431 843.8030
846.5430 865.8096 873.6541
902.2680 906.1049 910.8283
914.0269 944.6973 976.6124
979.3197 990.5288 1001.5520
1008.7389 1014.0071 1017.5168
1023.6563 1024.5351 1025.5525
1029.5913 1031.0936 1039.3418
1046.7457 1055.0652 1068.4025
1070.0075 1075.6833 1077.1971
1084.3534 1088.6815 1098.5573
1100.2537 1106.7834 1113.1513
1178.5236 1184.3050 1191.9374
1198.5893 1204.0807 1206.7206
1206.9505 1209.1442 1209.3926
1211.6892 1213.6500 1230.0580
1239.4696 1240.6355 1244.1595
1255.0937 1264.1533 1266.2154
1269.5939 1271.6708 1272.0004
1282.8881 1286.5962 1290.2263
1293.9050 1305.8161 1314.0641
1320.5198 1326.7135 1329.2162
1339.5513 1342.6320 1343.5884
1346.6389 1354.3045 1355.3084
1359.9451 1361.3671 1363.1231
1365.9063 1388.3327 1389.8080
1393.8268 1397.1837 1408.9409
1421.6941 1487.9621 1493.6229
1498.5540 1500.8047 1502.5612
1508.2157 1510.4199 1513.6481
1515.4825 1517.0346 1519.3442
1530.4895 1533.8017 1633.4403
1640.6626 1667.7924 1699.2542
3036.4784 3045.2683 3049.1485
3053.9807 3058.2581 3060.4754
3061.7532 3064.4324 3069.9478
3073.5726 3090.4324 3094.9153
3096.9545 3099.1855 3103.1286
3118.8186 3129.7254 3137.3112
3143.2529 3146.5003 3150.5121
3176.0983 3194.6507 3207.6978
3218.0998 3227.2319 3239.0738

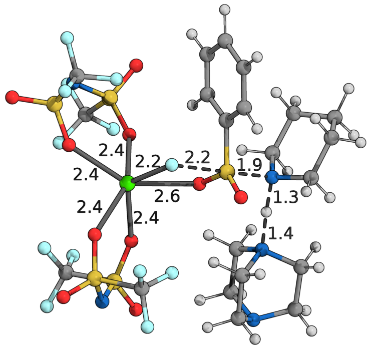

S3 ΔΔG‡ = +2.4 kcal/mol

Gaussian 16: ES64L-G16RevB.01 20-Dec-2017

B3LYP/6-31G(d,p) gfprint gfinput scf=(direct,tight,maxcycle=300,xqc) opt=(maxcycle=250,ts,calcfc,noeigentest) freq=noraman
N.EDU\11-Feb-2021\0\\# B3LYP/6-31G(d,p) gfprint gfinput scf=(direct,ti ght,maxcycle=300,xqc) opt=(maxcycle=250,ts,calcfc,noeigentest) freq=no

----
Full point group C1 NOp 1
Stoichiometry C21H28CaF13N5O10S5
Framework group
C1[X(C21H28CaF13N5O10S5)]

Num atoms: 83
Charge = 0 Multiplicity = 1

SCF = -5809.36065853 | Predicted change in Energy=-9.970363D-11

Optimization completed.
Maximum Force 0.000006 0.000450 YES
RMS Force 0.000000 0.000300 YES
Maximum Displacement 0.000776 0.001800 YES
RMS Displacement 0.000105 0.001200 YES

Atom Coordinates (in Angstroms)
Type X Y Z

Ca	0.751641	0.710382	-0.590766
S	-1.647026	3.177846	0.685636
O	-2.891611	3.214737	1.451367
O	-0.925056	1.868020	0.673242
C	-0.442733	4.301418	1.572160
F	0.699666	4.341303	0.875555
F	-0.943240	5.525973	1.682069
F	-0.199964	3.804621	2.787187
N	-1.858657	3.868096	-0.741603
S	-1.022045	3.531968	-2.083773
O	0.138038	2.620997	-1.880926
O	-0.815159	4.732667	-2.875579
C	-2.223506	2.462368	-3.036301
F	-3.361616	3.112526	-3.259927
F	-1.673823	2.107298	-4.194519
F	-2.484709	1.353628	-2.317583
S	4.387287	0.999951	-0.355503
O	5.459192	1.980507	-0.397198
O	3.009728	1.463709	-0.685916
C	4.728987	-0.244089	-1.707613
F	4.634855	0.356529	-2.892971
F	5.941479	-0.770879	-1.567576
F	3.809343	-1.220745	-1.635658
N	4.496686	0.092568	0.972333
S	3.281548	-0.528403	1.805255
O	3.564922	-1.898610	2.228924
O	1.916758	-0.292506	1.244492
C	3.271546	0.516495	3.353444
F	4.420234	0.387216	4.008810
F	3.085472	1.796374	3.017999
F	2.264460	0.116363	4.136368
S	-1.021443	-2.183204	-0.898509
O	-0.810321	-1.162321	0.159549
F	0.383506	-0.905186	-2.021040
O	-1.933799	-2.063345	-2.034264
C	2.541701	-4.922481	-1.605334
C	2.508684	-4.114094	-0.466957
C	1.419847	-3.273067	-0.234793
C	0.381778	-3.264779	-1.164512
C	0.399711	-4.044738	-2.319454
C	1.493810	-4.884291	-2.529130
H	3.393639	-5.571354	-1.782778
H	3.330378	-4.111732	0.241648
H	1.407657	-2.620044	0.629093
H	-0.401689	-3.968878	-3.045383
H	1.533326	-5.495145	-3.425440
C	-2.325035	-5.678418	-0.538945
C	-2.964603	-4.288706	-0.503432
C	-1.598519	-3.708548	1.511557
C	-0.982594	-5.111121	1.499376
H	-1.444137	-5.672219	-1.188723
H	-3.047149	-6.368428	-0.989729
H	-3.918644	-4.351342	0.036191
H	-3.179558	-3.905769	-1.502079
H	-0.892040	-2.948922	1.850938
H	-2.440685	-3.698823	2.216729
H	-0.033704	-5.102325	0.956943
H	-0.749576	-5.377973	2.536257
N	-2.165766	-3.239324	0.204096
H	-2.984348	-2.341000	0.532639
N	-3.920082	-1.416097	0.964749
C	-3.426274	-0.715362	2.196690
C	-4.476779	0.352209	2.635951
C	-6.243346	-1.020135	1.777846
C	-5.220666	-2.085975	1.272214
C	-4.142748	-0.389121	-0.106867
C	-5.220345	0.630107	0.375985
H	-3.267910	-1.476699	2.966960
H	-2.461018	-0.269413	1.950099
H	-4.812702	0.167822	3.661445
H	-4.044003	1.353911	2.594208
H	-6.556818	-1.239474	2.803430

H -7.141004 -1.020149 1.151489
H -5.031197 -2.860732 2.022449
H -5.563592 -2.577706 0.356897
H -3.182202 0.088804 -0.294607
H -4.435651 -0.917182 -1.018232
H -4.819605 1.646413 0.364271
H -6.100776 0.606482 -0.273994
N -5.649309 0.317925 1.748372
C -1.927912 -6.138697 0.867666
H -2.828687 -6.250740 1.487964
H -1.448499 -7.121946 0.829069

Statistical Thermodynamic Analysis
Temperature 298.150 Kelvin. Pressure 1.00000 Atm.

SCF = -5809.36065853 l Predicted change in Energy=-9.970363D-11
Zero-point correction (ZPE) = -5808.80272753 0.557931
Internal Energy (U) = -5808.74563653 0.615022
Enthalpy (H) = -5808.74469253 0.615966
Gibbs Free Energy (G) = -5808.90572253 0.454936

Frequencies
-938.8702 8.3264 11.0356
14.8000 15.8933 18.0876
20.3949 23.8287 25.0979
26.0604 33.0452 33.6355
38.5223 39.7486 41.3315
42.4244 44.8656 46.2355
51.6349 57.1988 57.6716
69.8655 77.5337 85.9625
88.5123 92.3951 99.9006
105.8515 107.8894 117.0282
118.2216 124.8382 135.3359
151.4473 165.5727 172.0787
180.2943 192.2034 195.0419
199.5253 200.9455 202.7381
205.3677 206.8413 211.5859
216.2768 225.0128 236.3254
250.8239 264.9586 267.5665
273.8924 278.6454 282.5681
288.8590 296.0496 296.7867
306.1075 308.9335 312.7148
317.0179 324.0631 324.5288
325.4014 328.2721 330.5333
337.7346 355.8213 363.3383
394.1457 395.7586 397.9208
401.6235 417.3107 422.5490
429.9951 431.9267 449.8746
468.3540 480.1662 486.1221
495.9863 498.1632 502.7640
522.2641 522.3508 535.0929
538.6807 539.4182 549.1222
549.2651 562.6491 565.8690
571.7686 573.4167 576.1495
582.9577 584.4599 589.8004
596.1164 611.3963 617.0593
622.9273 627.0982 683.3874
694.7912 713.2504 719.1249
720.1861 761.2708 762.0530
766.4041 781.4455 782.7947
791.8974 803.7225 804.1294
822.8331 843.5550 844.1865
848.6373 868.2734 875.0100
886.7542 893.9522 908.1184
908.6808 946.8260 963.1178
978.1993 993.0563 1003.8487
1015.9779 1018.0851 1020.2182
1023.2407 1025.7212 1028.6078
1033.0897 1036.0300 1044.4165
1048.2095 1058.3904 1067.8581
1072.2901 1075.0520 1078.6325
1086.2570 1088.9146 1099.6655
1107.0389 1109.2329 1115.3625
1172.4148 1189.3867 1190.3290
1199.3113 1204.4798 1205.8926
1206.7781 1207.8108 1210.0703
1211.6531 1213.6331 1218.3475
1234.7417 1237.8623 1240.4382
1245.1384 1263.5153 1267.8512
1269.8720 1272.8962 1275.4933
1282.8817 1287.8065 1294.1278
1298.6738 1316.5815 1317.5982
1327.6762 1329.8683 1337.8347
1341.3900 1341.5155 1349.1483
1352.8022 1355.8382 1356.9642
1358.9066 1364.0325 1366.5477
1371.0296 1393.3688 1395.0627
1398.5234 1402.0801 1413.7875
1421.6628 1487.9411 1498.4890
1498.8509 1500.9634 1502.6694
1504.6423 1506.7750 1509.6876
1513.2714 1515.7238 1518.2279
1520.9519 1533.0622 1632.0938

1641.4239 1689.9642 1703.6487
3023.6694 3030.2746 3040.3083
3053.2518 3060.1934 3060.2861
3063.6548 3066.0712 3066.9821
3072.8787 3091.1815 3092.1534
3096.1488 3107.5576 3117.1567
3122.5269 3127.1166 3132.6163
3137.1916 3142.0456 3148.0519
3171.3064 3195.2028 3208.0091
3218.1220 3226.7780 3239.1564

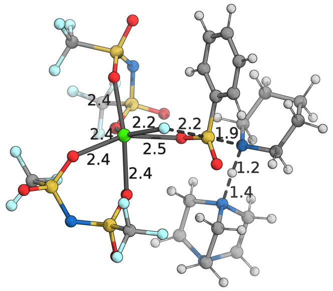

S4 ΔΔG‡ = +3.0 kcal/mol

Gaussian 16: ES64L-G16RevB.01 20-Dec-2017

B3LYP/6-31G(d,p) gfprint gfinput scf=(direct,tight,maxcycle=300,xqc) opt=(maxcycle=250,ts,calcfc,noeigentest) freq=noraman
N.EDU\09-Feb-2021\0\\# B3LYP/6-31G(d,p) gfprint gfinput scf=(direct,ti ght,maxcycle=300,xqc) opt=(maxcycle=250,ts,calcfc,noeigentest) freq=no

----

Full point group C1 NOp 1
Stoichiometry C21H28CaF13N5O10S5
Framework group C1[X(C21H28CaF13N5O10S5)]

Num atoms: 83
Charge = 0 Multiplicity = 1

SCF = -5809.36322012 | Predicted change in Energy=-2.227655D-09

Optimization completed.
Maximum Force 0.000014 0.000450 YES
RMS Force 0.000001 0.000300 YES
Maximum Displacement 0.001135 0.001800 YES
RMS Displacement 0.000186 0.001200 YES

Atom Type	Coordinates (in Angstroms) X	Y	Z
Ca	-0.688893	-0.190788	-1.182586
S	-2.468140	-3.307624	-1.764922
O	-2.139158	-1.870080	-2.006694
O	-3.303696	-3.975976	-2.748394
C	-3.446782	-3.236224	-0.172566
F	-3.841513	-4.451993	0.188235
F	-2.672738	-2.712222	0.788856
F	-4.511858	-2.452843	-0.350677
N	-1.209091	-4.236325	-1.370457
S	0.234869	-3.711135	-0.917129
O	0.875549	-4.659186	-0.010219
O	0.303195	-2.271427	-0.524019
C	1.230236	-3.748175	-2.499512
F	1.274868	-4.979028	-2.997425
F	0.670149	-2.922661	-3.389524
F	2.476529	-3.332655	-2.237287
S	-1.291392	1.401864	1.961062
O	-0.027403	1.642323	2.660045
O	-1.397498	0.171621	1.127053
C	-2.545751	1.124088	3.323072
F	-3.771128	1.015371	2.806481
F	-2.233082	-0.003547	3.963162
F	-2.512930	2.144640	4.176525
N	-1.786967	2.758094	1.242834
S	-2.451806	2.866585	-0.224367
O	-2.023657	1.793946	-1.165067
O	-2.335973	4.247301	-0.677724
C	-4.288227	2.538908	-0.026978
F	-4.769422	3.241845	0.996648
F	-4.910160	2.896950	-1.148386
F	-4.488331	1.236316	0.194311
S	2.411817	1.100121	-1.071822
O	1.335605	0.743277	-0.113421
F	0.825004	0.577146	-2.560981
O	3.269740	0.173652	-1.808348
C	1.653518	5.032399	-3.220215
C	0.925878	4.725512	-2.068355
C	1.164031	3.531487	-1.388482

C	2.130643	2.663011	-1.892827
C	2.856786	2.937553	-3.050542
C	2.614151	4.142844	-3.708664
H	1.460029	5.960520	-3.748814
H	0.152243	5.391837	-1.702410
H	0.594583	3.282229	-0.501454
H	3.561462	2.211971	-3.439929
H	3.162431	4.375237	-4.616058
C	5.379225	3.239336	-0.374740
C	5.053721	1.762642	-0.138603
C	3.193214	2.415538	1.406200
C	3.531351	3.892794	1.192277
H	4.844609	3.604947	-1.257430
H	6.449228	3.315150	-0.599027
H	5.700775	1.375441	0.659508
H	5.230036	1.150910	-1.024730
H	2.126989	2.244737	1.563804
H	3.706803	2.061079	2.310012
H	2.908224	4.313148	0.397918
H	3.268341	4.426860	2.111831
N	3.651608	1.493216	0.311138
H	3.669778	0.371945	0.846285
N	3.743617	-0.868422	1.515283
C	4.788884	-0.838681	2.582669
C	4.867822	-2.239952	3.268618
C	4.147830	-3.317353	1.254545
C	4.096401	-1.939180	0.526082
C	2.421018	-1.206886	2.143145
C	2.533564	-2.584641	2.863815
H	5.739535	-0.560759	2.116214
H	4.517451	-0.052176	3.293587
H	5.863448	-2.677370	3.142193
H	4.679422	-2.154485	4.343503
H	5.130393	-3.785676	1.135703
H	3.399041	-3.997207	0.839990
H	5.059017	-1.669321	0.080426
H	3.349147	-1.918606	-0.266856
H	2.155282	-0.393913	2.824243
H	1.677934	-1.227340	1.347936
H	2.335443	-2.485987	3.935938
H	1.807627	-3.290312	2.451390
N	3.879733	-3.154703	2.691652
C	5.011355	4.090972	0.845699
H	5.636886	3.798677	1.701149
H	5.219527	5.147822	0.650606

Statistical Thermodynamic Analysis
Temperature 298.150 Kelvin. Pressure
1.00000 Atm.

SCF = -5809.36322012 l Predicted change in
Energy=-2.227655D-09
Zero-point correction (ZPE) = -5808.80520612
0.558014
Internal Energy (U) = -5808.74826812
0.614952
Enthalpy (H) = -5808.74732412   0.615896
Gibbs Free Energy (G) = -5808.90670712
0.456513

Frequencies
-855.7315 7.2071 10.7975
14.7586 18.4966 23.2018
27.7430 30.1613 30.5113
32.1484 32.6377 34.9059
37.3193 38.7384 40.7677
43.5733 47.3486 50.4433
54.0196 66.7596 69.9440
72.7213 75.9433 86.3909
88.3837 93.1682 97.1149
105.1666 107.3070 113.6282
122.4829 125.7754 134.4502
151.8894 159.2843 164.6722
183.7444 186.0133 195.3363
197.3810 199.7934 203.6306
204.6698 209.7975 214.5076
216.9209 228.6209 240.5974
243.3873 266.5845 270.3596
277.8532 280.4296 283.5934
287.8359 296.2565 296.6563
308.0761 309.8935 313.5333
323.0617 326.0160 326.4802
328.5315 330.8201 332.5562
339.1376 357.3468 367.8139
391.3072 395.1079 395.9526
410.5580 416.0401 422.9166
426.9235 432.1917 452.1724
469.8861 483.7221 484.9382
497.4721 500.7359 503.9282
519.4001 522.8183 533.3961
537.4733 539.9641 548.1728
548.9279 561.4160 565.1305
569.4803 577.7430 580.6781
582.2725 583.6465 584.0502
594.6716 607.5628 622.3155
626.5343 626.9583 686.9003
694.6122 714.6636 715.4080
718.8068 761.2266 762.3526

763.2600 781.2019 782.4737
791.6097 803.3642 803.9916
820.5237 842.1719 843.1256
848.2707 861.8803 874.9375
887.4846 895.0978 908.8906
911.4954 944.8806 963.2143
980.4958 990.0541 1004.7417
1005.4879 1015.4595 1015.7551
1018.0788 1022.1890 1028.0233
1033.6033 1033.9370 1044.0321
1049.9947 1060.0452 1066.8239
1071.1502 1074.6313 1077.8568
1080.0878 1085.8621 1098.9110
1105.8295 1109.6377 1115.7060
1172.3653 1190.2574 1191.6036
1199.4372 1201.6000 1206.2036
1206.6382 1207.5490 1210.5142
1212.1601 1212.2959 1218.4528
1235.1083 1237.9969 1242.2604
1254.3107 1259.9045 1261.9884
1268.7749 1270.5786 1274.8909
1283.3717 1288.2516 1295.0724
1299.0555 1306.6639 1318.3866
1321.6249 1331.6988 1340.1151
1340.8621 1342.4680 1351.2607
1353.7749 1355.8947 1358.7700
1360.0775 1364.4632 1366.1220
1368.1592 1391.3358 1396.1844
1399.3678 1404.4270 1416.4002
1424.7170 1488.7353 1497.4778
1498.7216 1500.9387 1501.4996
1505.9009 1508.2238 1510.2956
1511.3514 1515.7831 1516.5423
1522.9945 1529.9002 1632.9716
1642.0627 1684.7244 1698.3846
3024.6565 3034.0042 3039.3276
3052.6780 3059.3641 3059.4114
3061.1472 3064.6093 3067.8435
3074.9938 3091.0642 3091.9464
3094.9226 3106.3974 3115.5143
3117.6624 3119.9401 3122.8708
3139.8148 3145.3871 3163.8862
3179.1786 3195.9592 3209.4024
3220.8516 3228.5547 3234.5961

Single Point Energies in ωB97x-D/def2-TZVP/PCM(THF)

Structure	E (Hartrees)
**S1,** $\Delta\Delta G^{\ddagger}$ = 1.9 kcal/mol	-5810.1706
**S2,** $\Delta\Delta G^{\ddagger}$ = 2.1 kcal/mol	-5810.1678
**S3,** $\Delta\Delta G^{\ddagger}$ = 2.4 kcal/mol	-5810.1664
**S4,** $\Delta\Delta G^{\ddagger}$ = 3.0 kcal/mol	-5810.1672

Natural Charges from Natural Population Analysis

Natural population analysis (NPA) was performed on the optimized geometries of benzenesulfonyl fluoride **1** in the ground state and the transition state complex **16**. All NPA calculations were performed using the same level of theory as that used in the rest of the manuscript – ωB97xD/def2-TZVP/PCM(THF) natural charges on B3LYP/6-31G(d,p) geometries. The natural charges of the atoms at the SO_2F moiety was compared across both states.

Input Parameters:
# wb97xd/def2tzvp gfprint gfinput scf=(direct,tight,maxcycle=300,xqc)
SCRF=(PCM,SOLVENT=THF) pop=npa

Summary of Natural Population Analysis for benzenesulfonyl fluoride 1
(Highlighted atoms represent the SO_2F moiety):

Atom	No	Natural Charge	Natural Population Core	Valence	Rydberg	Total
C	1	-0.17071	1.99909	4.15209	0.01953	6.17071
C	2	-0.37169	1.99876	4.34248	0.03045	6.37169
C	3	-0.17070	1.99909	4.15208	0.01953	6.17070
C	4	-0.20588	1.99922	4.18774	0.01892	6.20588
C	5	-0.16815	1.99923	4.15074	0.01818	6.16815
C	6	-0.20587	1.99922	4.18773	0.01892	6.20587
H	7	0.24837	0.00000	0.75040	0.00123	0.75163
H	8	0.24837	0.00000	0.75040	0.00123	0.75163
H	9	0.23417	0.00000	0.76470	0.00113	0.76583
H	10	0.23122	0.00000	0.76772	0.00105	0.76878
H	11	0.23417	0.00000	0.76470	0.00113	0.76583
S	12	2.36919	9.99845	3.40298	0.22938	13.63081
O	13	-0.89373	1.99981	6.85939	0.03453	8.89373
F	14	-0.48499	1.99996	7.47550	0.00954	9.48499
O	15	-0.89375	1.99981	6.85941	0.03453	8.89375

Summary of Natural Population Analysis for transition state complex 16
(Highlighted atoms represent the SO_2F moiety):

Atom	No	Natural Charge	Natural Population Core	Valence	Rydberg	Total
Ca	1	1.85764	17.99079	0.09003	0.06154	18.14236
S	2	2.20370	9.99867	3.57027	0.22736	13.79630
O	3	-1.02375	1.99982	6.99766	0.02628	9.02375
O	4	-0.88649	1.99982	6.85303	0.03364	8.88649
C	5	0.71863	1.99979	3.20759	0.07399	5.28137
F	6	-0.29250	1.99991	7.28572	0.00687	9.29250

F	7	-0.28774	1.99991	7.28089	0.00694	9.28774
F	8	-0.29989	1.99991	7.29278	0.00720	9.29989
N	9	-1.20113	1.99953	6.15301	0.04859	8.20113
S	10	2.20581	9.99865	3.56774	0.22781	13.79419
O	11	-0.89787	1.99982	6.86413	0.03392	8.89787
O	12	-1.02594	1.99982	6.99928	0.02684	9.02594
C	13	0.71797	1.99979	3.20852	0.07373	5.28203
F	14	-0.28847	1.99991	7.28163	0.00693	9.28847
F	15	-0.29679	1.99991	7.28970	0.00718	9.29679
F	16	-0.29839	1.99991	7.29127	0.00721	9.29839
S	17	2.19284	9.99866	3.58154	0.22696	13.80716
O	18	-0.90873	1.99982	6.87538	0.03353	8.90873
O	19	-1.00694	1.99982	6.97862	0.02849	9.00694
C	20	0.72539	1.99979	3.20075	0.07407	5.27461
F	21	-0.29033	1.99991	7.28349	0.00693	9.29033
F	22	-0.29662	1.99991	7.28970	0.00702	9.29662
F	23	-0.29191	1.99991	7.28508	0.00691	9.29191
N	24	-1.20356	1.99955	6.15591	0.04810	8.20356
S	25	2.19601	9.99866	3.57718	0.22816	13.80399
O	26	-1.01717	1.99981	6.99002	0.02733	9.01717
O	27	-0.89690	1.99982	6.86341	0.03367	8.89690
C	28	0.72339	1.99979	3.20271	0.07411	5.27661
F	29	-0.29650	1.99991	7.28957	0.00702	9.29650
F	30	-0.29151	1.99991	7.28467	0.00693	9.29151
F	31	-0.28914	1.99991	7.28229	0.00694	9.28914
S	32	2.29662	9.99812	3.50500	0.20026	13.70338
O	33	-1.01046	1.99983	6.97678	0.03385	9.01046
F	34	-0.82802	1.99999	7.82313	0.00491	9.82802
O	35	-0.90765	1.99982	6.87193	0.03590	8.90765
C	36	-0.18025	1.99923	4.16343	0.01759	6.18025
C	37	-0.19624	1.99922	4.17888	0.01815	6.19624
C	38	-0.20046	1.99904	4.17995	0.02146	6.20046
C	39	-0.29573	1.99878	4.26349	0.03347	6.29573
C	40	-0.19238	1.99906	4.17399	0.01932	6.19238
C	41	-0.19909	1.99922	4.18161	0.01825	6.19909
H	42	0.23097	0.00000	0.76805	0.00098	0.76903
H	43	0.23937	0.00000	0.75955	0.00108	0.76063
H	44	0.24054	0.00000	0.75653	0.00293	0.75946
H	45	0.24939	0.00000	0.74938	0.00123	0.75061
H	46	0.23316	0.00000	0.76580	0.00104	0.76684
C	47	-0.40577	1.99943	4.39274	0.01361	6.40577
C	48	-0.25030	1.99933	4.23395	0.01702	6.25030
C	49	-0.24071	1.99933	4.22370	0.01767	6.24071
C	50	-0.40716	1.99943	4.39399	0.01375	6.40716
H	51	0.19812	0.00000	0.79977	0.00211	0.80188
H	52	0.23207	0.00000	0.76694	0.00099	0.76793
H	53	0.20575	0.00000	0.79204	0.00221	0.79425
H	54	0.24969	0.00000	0.74886	0.00145	0.75031
H	55	0.23426	0.00000	0.76445	0.00129	0.76574

H	56	0.20369	0.00000	0.79401	0.00230	0.79631
H	57	0.20339	0.00000	0.79490	0.00171	0.79661
H	58	0.22951	0.00000	0.76949	0.00100	0.77049
N	59	-0.61513	1.99958	5.58010	0.03544	7.61513
H	60	0.49269	0.00000	0.50419	0.00312	0.50731
N	61	-0.43350	1.99964	5.40666	0.02720	7.43350
C	62	-0.25572	1.99939	4.24125	0.01508	6.25572
C	63	-0.24907	1.99941	4.23383	0.01584	6.24907
C	64	-0.25716	1.99940	4.24226	0.01550	6.25716
C	65	-0.25825	1.99938	4.24327	0.01561	6.25825
C	66	-0.25853	1.99938	4.24441	0.01474	6.25853
C	67	-0.25145	1.99941	4.23677	0.01527	6.25145
H	68	0.21306	0.00000	0.78534	0.00160	0.78694
H	69	0.21085	0.00000	0.78720	0.00195	0.78915
H	70	0.21025	0.00000	0.78831	0.00145	0.78975
H	71	0.20787	0.00000	0.79065	0.00148	0.79213
H	72	0.20591	0.00000	0.79264	0.00145	0.79409
H	73	0.22198	0.00000	0.77664	0.00138	0.77802
H	74	0.21934	0.00000	0.77924	0.00143	0.78066
H	75	0.22612	0.00000	0.77176	0.00211	0.77388
H	76	0.20953	0.00000	0.78889	0.00158	0.79047
H	77	0.23478	0.00000	0.76333	0.00190	0.76522
H	78	0.20909	0.00000	0.78947	0.00144	0.79091
H	79	0.21313	0.00000	0.78546	0.00141	0.78687
N	80	-0.40568	1.99960	5.37899	0.02710	7.40568
C	81	-0.39946	1.99944	4.38680	0.01322	6.39946
H	82	0.20386	0.00000	0.79472	0.00143	0.79614

Table S10. Natural charges of atoms at the sulfur(VI) of benzenesulfonyl fluoride in ground state 1 and transition state complex **16**.

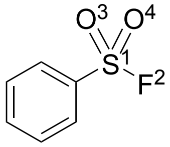

Atom	Natural charge in **1**	Natural charge in **16**
S-1	2.369	2.297
F-2	-0.485	-0.828
O-3	-0.894	-1.010[a]
O-4	-0.894	-0.908

[a] This atom coordinates directly to Ca^{2+} (see complex **16**).

General Experimental Methods:

All commercially available chemicals, reagents, and solvents were used as received. Calcium(II) bis(trifluoromethanesulfonimide) ($Ca(NTf_2)_2$) (CAS # 165324-09-4) was purchased from TCI America. Reactions were monitored by thin layer chromatography (TLC) performed on Merck silica gel plates (60 F254) (80:20 hexanes: ethyl acetate mobile phase) and were visualized with ultraviolet (UV) light (254 nm). Proton nuclear magnetic resonance (^1H NMR) spectra, carbon nuclear magnetic resonance (^{13}C NMR) spectra, and fluorine nuclear magnetic resonance (^{19}F NMR) spectra were recorded on a Bruker 400 (400, 101, and 376 MHz, respectively) equipped with cryoprobes and using the Bruker TOPSPIN 1.3 software. Chemical shifts are reported in parts per million (ppm) relative to chloroform (^1H δ = 7.26 and ^{13}C δ = 77.16). The NMR peak multiplicities were reported as follows: singlet (s), doublet (d), triplet (t); quartet (q). High-resolution mass spectra (HRMS) were acquired on an Agilent model 6220 MS(TOF). Column chromatography was performed on a Teledyne ISCO Combi*Flash* NextGen 300 system using a pre-packed 25 gram 60 Å silica column. Aluminum heating blocks were used for reactions that required elevated temperature.

General ^{19}F NMR Kinetic Trial Procedure and Data Extraction:

2D ^{19}F NMR experiments were conducted with the Bruker TOPSPIN 1.3 software using a pulse program, "zgkinetics_F", developed by Professor Daniel O'Leary at Pomona College. The pulse parameters for this method are included (*vide infra*). The number of scans (NS) for each FID was set to 4. To prevent differences in acquisition parameters between the first scan and subsequent scans, the number of dummy scans (DS) was set to 0. The sweep width (SHW) was set to 200 ppm and the spectrum was centered (O1P) around 0 ppm. The relaxation delay (D1) and delay between scans (D2) were set to 3 and 0 seconds respectively. The time between measurements (D3) was set to 60. The number of measurements of each scan was set by the size of the FID (TD), this size was adjusted based on the speed of the reaction but was typically between 25 and 60. These delay parameters and FID sizes yielded sets of four ^{19}F scans, spaced 80 seconds apart, for a total experiment duration of between 32 minutes and 1 hour 20 minutes.

Once the sample was loaded into the NMR, it was locked to d-THF. The appropriate receiver gain was automatically determined and set using the RGA command in Bruker TOPSPIN 1.3, then the pulse program was initiated. Once the run was complete, a slice of the 2D spectra was extracted with the RSR command and phased. The zero (PCH0) and first (PCH1) order phase corrections were copied over to the 2D spectrum, and the data were reprocessed, applying the phasing to every scan. The Bruker T1/T2 Relaxation subroutine was used to analyze each run in Bruker TOPSPIN 1.3. Following the instructions of the subroutine, a slice of the 2D experiment was extracted, then the range of the signal of interest (the $PhSO_2F$ peak) was integrated. This range was saved and exported to the relaxation module. Opening the Relaxation Window showed the $PhSO_2F$ peak integrations across all FIDs. The area of each measurement was expressed as values between 0 and 1, normalized to the measurement with the largest integration area (1). The measurement with the largest integration area was typically the first measurement. The

normalized integration data were then exported to Excel where the data were scaled such that the measurement with the largest integration area (1) was calibrated to match the starting concentration of $PhSO_2F$. Consequently, in proportion to their relative integration, all subsequent measurements were expressed as concentrations of $PhSO_2F$ lower than the initial concentration of the reaction. These data were taken to be representative of the concentration of $PhSO_2F$ in solution over time.

Pulse parameters for the “zgkinetics_F” NMR method

```
;zghfigqn OLeary for 19F{1H} during kinetics
;2D sequence for kinetics
;avance-version (02/05/31)
;1D sequence for H-1 observe with inverse gated F-19 decoupling
;for QNP-operation (F-19 via X-QNP output of switchbox)
;
;$CLASS=HighRes
;$DIM=1D
;$TYPE=
;$SUBTYPE=
;$COMMENT=

#include <Avance.incl>

"d11=30m"
"d12=20u"

  d11 QNP_X
1 ze
  d11 pl12:f2
2 d1 do:f2
  d12 SWITO_F
  p1 ph1
  d12 SWITO_H
  go=2 ph31 cpd2:f2
  d3 wr #0 if #0 do:f2
  lo to 2 times td1
  30m do:f2
exit

ph1=0 2 2 0 1 3 3 1
ph31=0 2 2 0 1 3 3 1

;pl1 : f1 channel - power level for pulse (default)
;pl12: f2 channel - power level for CPD/BB decoupling
;p1 : f1 channel - 90 degree high power pulse
;d1 : relaxation delay; 1-5 * T1
;d11: delay for disk I/O [30 msec]
;d12: delay for power switching [20 usec]
```

```
;NS: 1 * n, total number of scans: NS * TD0
;cpd2: decoupling according to sequence defined by cpdprg2
;pcpd2: f2 channel - 90 degree pulse for decoupling sequence


;$Id: zghfigqn,v 1.7.10.1 2005/11/10 13:19:00 ber Exp $
```

Variable Temperature ^{19}F NMR Setup Procedure

Compressed air was run over the shim coils to maintain the uniformity of the magnetic field over the duration of all variable temperature NMR experiments. The probe temperature and heater were set and monitored using the EDTE command in the Bruker TOPSPIN 1.3 Program. For low temperature experiments the probe temperature was maintained using both the probe heater and cooled air. A copper coil was spliced into the NMR airline such that compressed air ran through the coil prior to running over the probe. The copper coil was placed within a cooling dewar and crush dried ice was packed around the coil. For all variable temperature experiments, the probe was allowed to equilibrate at the reported temperature for 5 minutes prior to introducing the sample. The probe temperature was monitored throughout the experiments to ensure the desired temperature was maintained.

Activation Parameters Experimental Determination

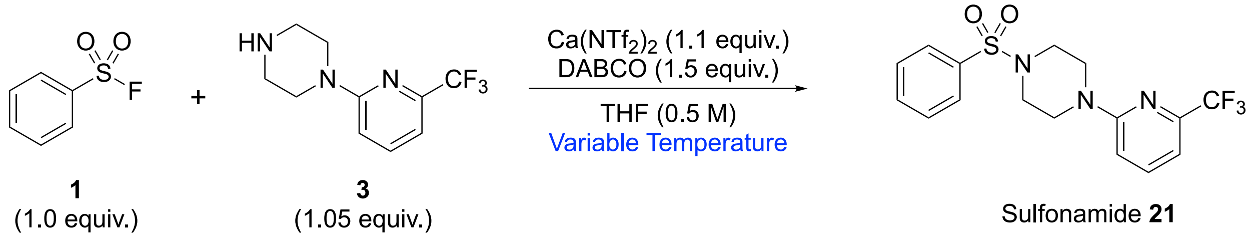

Scheme 1. Model Reaction for Activation Parameter Determination

To a 3.7 mL scintillating glass vial **3** (78.9 mg, 0.341 mmol, 1.05 equiv.), $Ca(NTf_2)_2$ (214.6 mg, 0.356 mmol, 1.1 equiv.), and 1,4-diazabicyclo[2.2.2]octane (DABCO) (54.7 mg, 0.488 mmol, 1.5 equiv.) were added and dissolved in anhydrous THF (0.65 mL). The solution was transferred to a 0.5 mm NMR tube and loaded into a Bruker 400 NMR spectrometer with the probe temperature pre-equilibrated. The solution temperature was allowed to equilibrate for five minutes. Removing the NMR tube from the NMR, **1** (52.1 mg, 40 µL, 0.325 mmol, 1 equiv.) was added to the solution and the NMR tube was inverted five times to thoroughly mix. The NMR tube was immediately loaded back into the NMR spectrometer and the reaction progress was studied using ^{19}F NMR kinetic spectroscopy (376 MHz) by monitoring the disappearance of the $PhSO_2F$ signal using the protocol described in the **General Fluorine Nuclear Magnetic Resonance Kinetic Trial Procedure and Data Extraction** section. Experiments were conducted with an FID size of 25 (32 minutes total) or longer, with most reactions monitored for 40 measurements (52 minutes total).

The activation parameters were estimated using the initial rates of the reaction. The initial rates were determined by plotting the kinetic data as a function of the concentration of $PhSO_2F$ (**1**) versus time (Figure S2). A linear regression was performed on a plot of the concentration of $PhSO_2F$ over the first ten minutes and the slope was used as the initial rate. The initial rates were determined from trials at 5 °C, 15 °C, 25 °C, 35 °C, and 45 °C and reported in Table S1. The initial rate was used as an approximation of k, the rate constant. The value of ln(k/T) is reported for each trial (Table S1). These data were plotted as ln(k/T) versus 1/T in an Eyring Plot (Figure S3). Following a method of eyring plot analysis previously used[1], in NCSS a linear regression was performed on $R\ln(hk/k_BT)$ vs -1/T, where R is the gas constant (1.9872 cal mol^{-1} K^{-1}), k is the initial

rate of reaction, h is Planck's constant (1.58367 X 10^{-34} cal s), k_B is Boltzmann's constant (3.29983 X 10^{-24} cal K^{-1}), and T is the Kelvin temperature. This linear regression yielded the values of $\Delta H^‡$ as the slope and $\Delta S^‡$ as the y-intercept. The standard error in $\Delta H^‡$ and $\Delta S^‡$ from the linear regression were reported (Figure S3). Using the relationship: $\Delta G^‡ = \Delta H^‡ - T\Delta S^‡$, the experimental Gibbs free energy of activation was then determined at 25 °C ($\Delta G^{0‡}_{exp}$). The error in $\Delta G^{0‡}_{exp}$ was calculated using the error in $\Delta H^‡$ and $\Delta S^‡$ and the error in their covariance, which was calculated in the variance-covariance matrix generated during the linear regression calculation in NCSS.

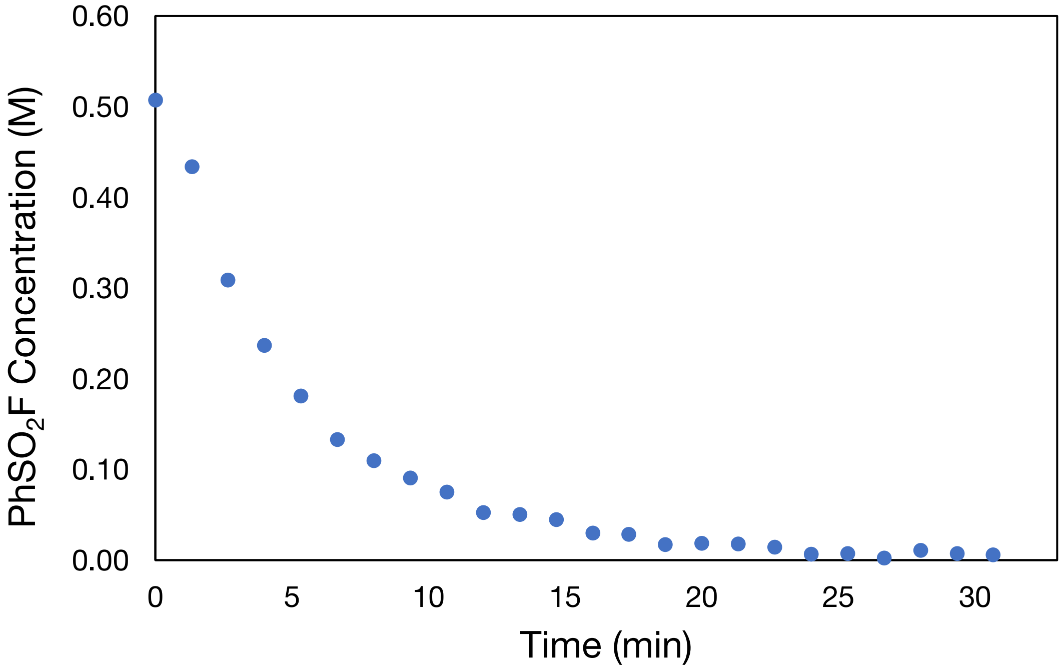

Figure S2. Representative Kinetic Data of $PhSO_2F$ Consumption at 25°C

Table S1. Initial Reaction Rates as a Function of Temperature

Temperature (°C)	Temperature (K)	Initial Rate (M s^{-1})	ln(k/T)	1/T
5	278.15	9.52 X 10^{-4}	-12.58	0.0036
5	278.15	7.07 X 10^{-4}	-12.88	0.0036
5	278.15	7.89 X 10^{-4}	-12.77	0.0036
15	288.15	5.59 X 10^{-4}	-13.15	0.0035
15	288.15	2.81 X 10^{-4}	-13.84	0.0035
15	288.15	8.90 X 10^{-4}	-12.69	0.0035
15	288.15	1.01 X 10^{-3}	-12.56	0.0035
25	298.15	1.06 X 10^{-3}	-12.55	0.0034
25	298.15	1.09 X 10^{-3}	-12.51	0.0034
35	308.15	1.23 X 10^{-3}	-12.43	0.0032
35	308.15	1.35 X 10^{-3}	-12.34	0.0032
45	318.15	1.47 X 10^{-3}	-12.28	0.0031

45	318.15	1.42×10^{-3}	-12.32	0.0031

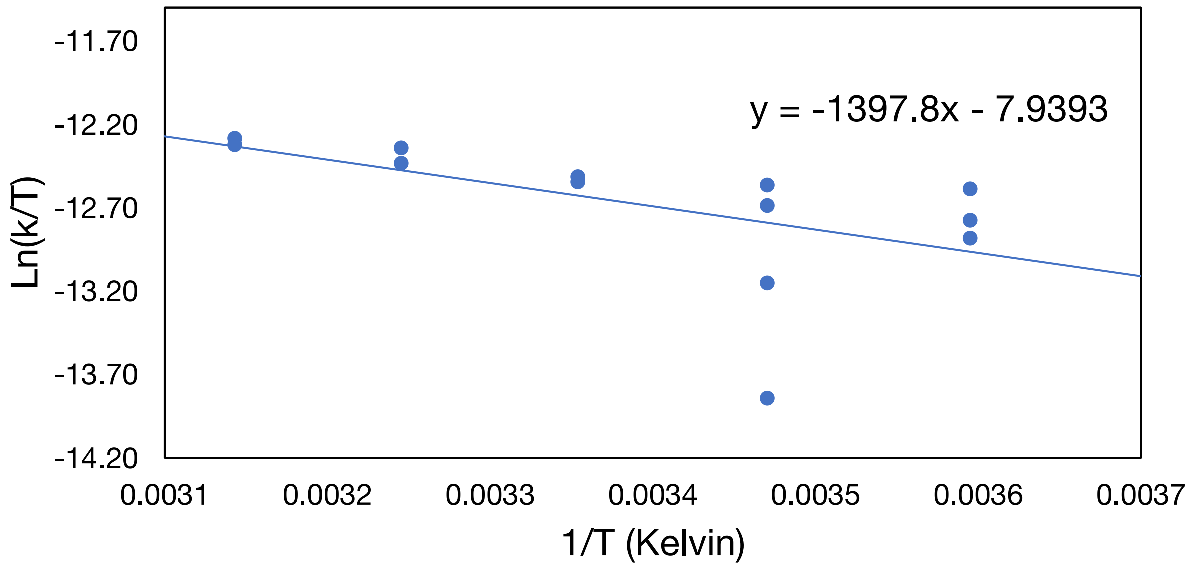

$\Delta H^{\ddagger}$ = 2.8 ± 1.3 kcalmol^{-1}, $\Delta S^{\ddagger}$ = -63 ± 4.5 calmol^{-1}k^{-1}
$\Delta G^{0\ddagger}_{exp}$ = 21.5 ± 0.14 kcalmol^{-1})

Figure S3. $PhSO_2F$ and Piperazine **2** Eyring Plot

$Ca(NTf_2)_2$ Rate Order Experimentation

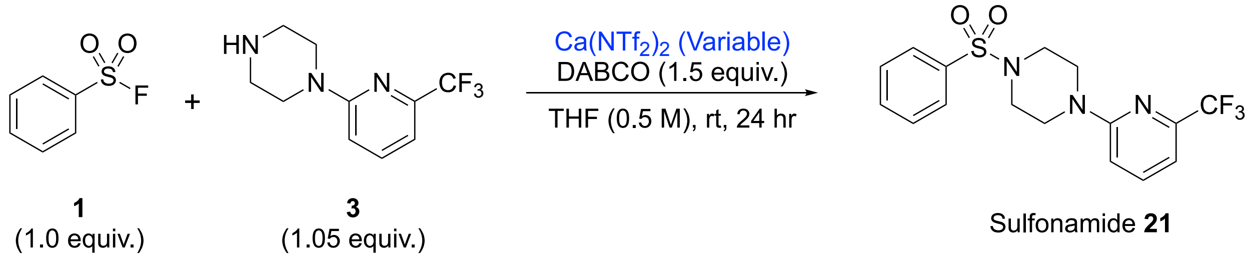

Sulfonamide **21**

Scheme 2. $Ca(NTf_2)_2$ Rate Study Reaction

To a 3.7 mL (1 dram) scintillating glass vial **3** (78.9 mg, 0.341 mmol, 1.05 equiv.), $Ca(NTf_2)_2$ (ranging from 19.5 - 390 mg, 0.0325 mmol - 0.650 mmol, 0.1 - 2 equiv.), and DABCO (54.7 mg, 0.488 mmol, 1.5 equiv.) were added and dissolved in anhydrous THF (0.65 mL). The solution was transferred to an NMR tube. **1** (52.1 mg, 40 µL, 0.325 mmol, 1 equiv.) was added to the solution and the NMR tube was inverted five times to thoroughly mix. The NMR tube was immediately placed into the NMR spectrometer and the reaction progress was studied using ^{19}F NMR spectroscopy (376 MHz) by monitoring the disappearance of the $PhSO_2F$ (**1**) signal using the protocol described in the **General Fluorine Nuclear Magnetic Resonance Kinetic Trial Procedure and Data Extraction** section. Experiments were monitored with an FID size of 40 (52 minutes total).

The initial rates were determined by plotting the data as a function of the concentration of $PhSO_2F$ versus time. A linear regression was performed on a plot of the concentration of $PhSO_2F$ over the first ten minutes and the slope was used as the initial rate. The initial rate was determined at each $Ca(NTf_2)_2$ concentration and reported in Table S2 as an average of duplicate runs. The error in the initial rate was calculated as two times the standard deviation between the initial rate values determined from each trial. Initial rate data were plotted as a function of $Ca(NTf_2)_2$ (Figure S4a). It was observed that this plot resembled a saturation kinetics curve, with a linear region from 0.1 to 0.5 M $Ca(NTf_2)_2$. The initial rates at these concentrations were plotted separately and a linear regression was performed to assess how well the kinetic data fit a first order rate approximation with respect to $Ca(NTf_2)_2$ (Figure S4b). A second order approximation was also modeled by plotting the squared concentration of $Ca(NTf_2)_2$ against the initial rates from 0.1 to 0.5 M and the second order model's fit was assessed using a linear regression (Figure S4c). It was found that the first order model produced a higher R-squared value than the second order model, indicating that the first order model better explains the observed relationship between the concentration of $Ca(NTf_2)_2$ and the reaction rate.

Table S2. Initial Reaction Rates as a Function of $Ca(NTf_2)_2$ Concentration

**$Ca(NTf_2)_2$ Concentration (M)**	**Initial Rate ($M\ s^{-1}$)**	**Error in Initial Rate ($M\ s^{-1}$)**
0.10	4.35×10^{-4}	$\pm 9.73 \times 10^{-4}$
0.25	9.64×10^{-3}	$\pm 1.59 \times 10^{-3}$
0.375	2.59×10^{-2}	$\pm 2.88 \times 10^{-4}$
0.50	3.21×10^{-2}	$\pm 2.69 \times 10^{-3}$
1.5	3.77×10^{-2}	$\pm 1.00 \times 10^{-2}$

2.0	3.77 X 10^{-2}	± 1.00 X 10^{-2}

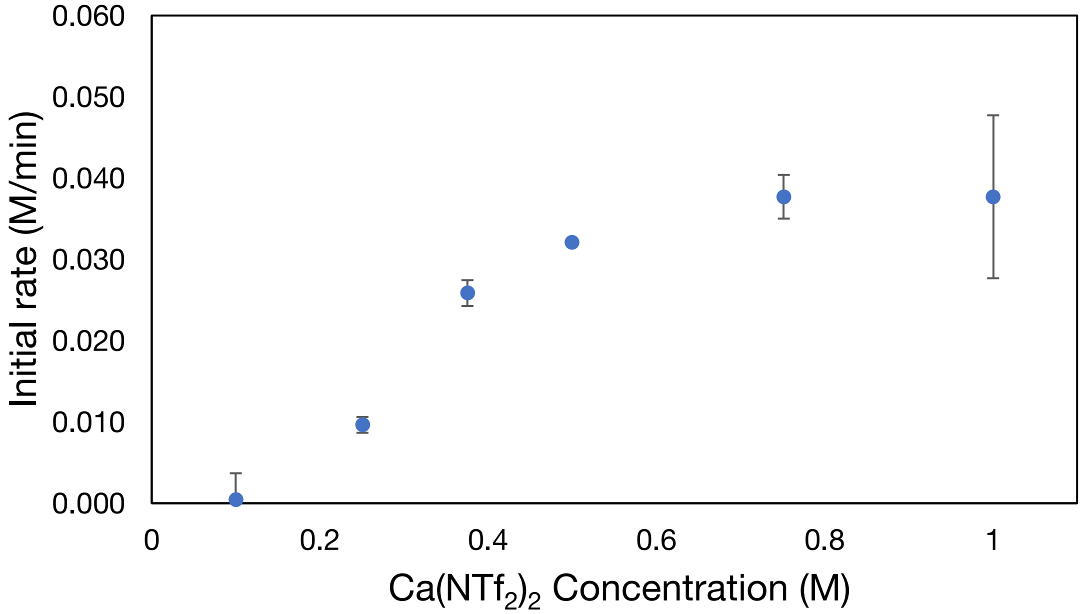

Figure S4a. $Ca(NTf_2)_2$ Concentration and Initial Rate

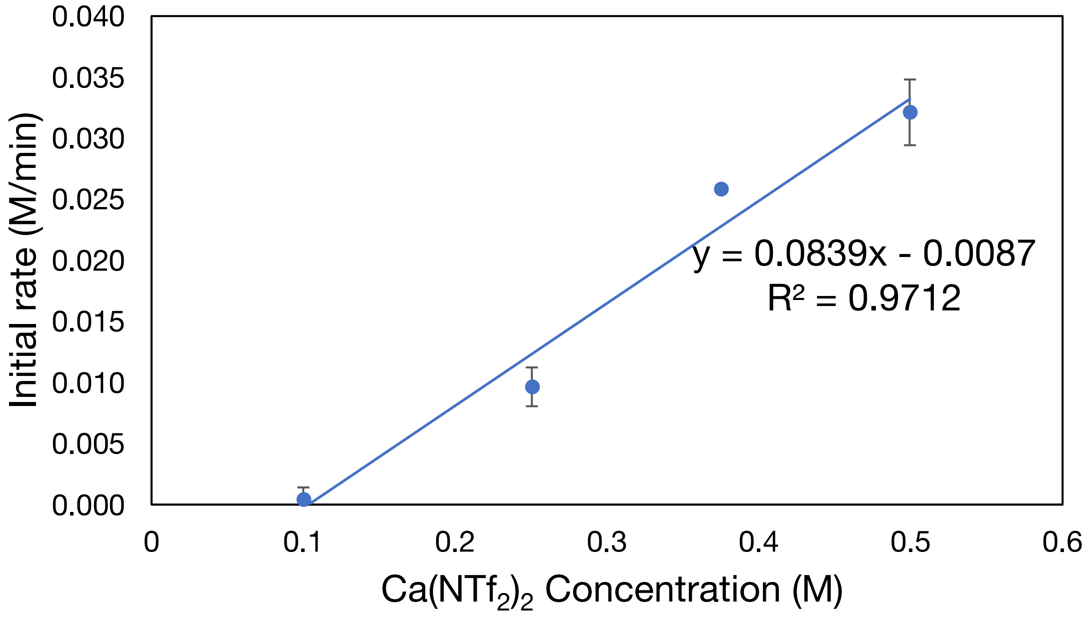

Figure S4b. First Order Model of Initial Rates Between 0.1 M and 0.5 M $Ca(NTf_2)_2$

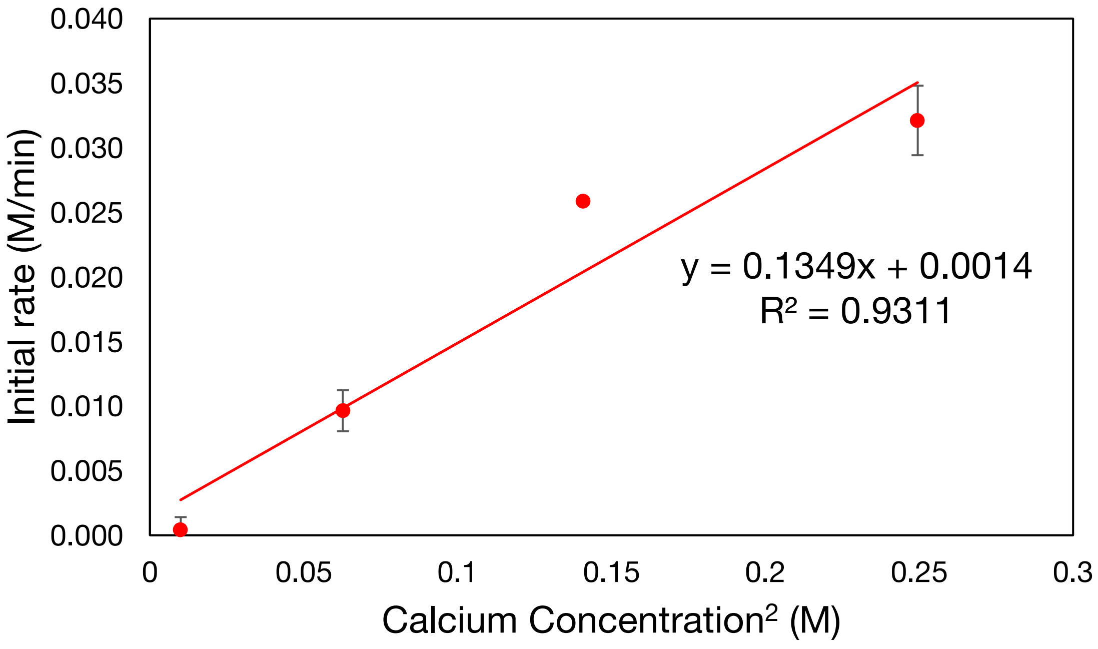

Figure S4c. Second Order Model of Initial Rates Between 0.1 M and 0.5 M $Ca(NTf_2)_2$

^{19}F NMR Yield Method

Reactions were carried out in 3.7 mL (1 dram) reaction vessels, then trifluorotoluene (9.74 mg, 8.2 µL, 0.067 mmol, 0.33 equiv.) was added directly to the reaction mixture. The amount of trifluorotoluene was adjusted to match the fluorine content of the starting amount of sulfonyl fluoride added. This was done so that trifluorotoluene could serve as an internal standard with an integrated ^{19}F NMR signal of corresponding area to the starting amount of sulfonyl fluoride reagent. The reaction vessel was vortexed to thoroughly mix. The reaction mixture was transferred to a 0.5 mm NMR tube. The reaction vessel was washed with deuterated chloroform (approx. 0.5 ml) to spike the sample and promote complete transfer of the reaction mixture and fluorine standard to the NMR tube, which was then vortexed and inverted to mix. Spiking the sample with a deuterated solvent allowed for the sample to be locked onto by the NMR instrument, which was found to help produce consistent phasing and chemical shifts of the peaks.

The sample was loaded into a Bruker 400 NMR spectrometer, locked to $CDCl_3$, and the shims were manually adjusted. A preliminary ^{19}F NMR spectrum was acquired with a spectral width of 200 ppm and an offset of 0 ppm. This preliminary spectrum was used to determine the chemical shifts of the sulfonyl fluoride fluorine (approx. 65 ppm) and trifluorotoluene fluorines (approx. -64 ppm). Then the acquisition offset (O1P) was adjusted to center a scan about the sulfonyl fluoride signal and the spectral width was reduced to approximately 5 ppm. An ^{19}F scan was taken of the sulfonyl fluoride peak. Immediately following this, and with the exact same acquisition parameters, the offset was adjusted to center the scan about the trifluorotoluene signal and a third scan was

taken. It was ensured that both signals were centered in their respective spectra to avoid signal distortion. Both the sulfonyl fluoride and trifluorotoluene signals were roughly phased using the automatic phasing command, APK, in the Bruker TOPSPIN 1.3 software, followed by manual phasing and inspection to ensure each signal were satisfactorily phased.
The signals from both the sulfonyl fluoride and trifluorotoluene spectra were integrated using the same scale and the trifluorotoluene integration was calibrated to 1.00. Adjusting for the exact quantity of sulfonyl fluoride and trifluorotoluene added, the relative integration of the sulfonyl fluoride signal was used to determine the remaining sulfonyl fluoride as a percentage of the initial amount of sulfonyl fluoride present. Subtracting the percentage of sulfonyl fluoride that remained from 100% yielded the percentage of sulfonyl fluoride consumed, which was reported as the reaction yield by NMR. Trials completed in duplicate were reported as an average of both measurements. Where applicable, the excess yield due to $Ca(NTf_2)_2$ was calculated by subtracting the reaction yield from the yield of the corresponding reaction without $Ca(NTf_2)_2$.

Additive and Reaction Conditions Screening with Secondary Amine Nucleophile

To a 3.7 mL (1 dram) scintillating glass vial **3** (48.6 mg, 0.210 mmol, 1.05 equiv.), $Ca(NTf_2)_2$ (12.0 mg, 0.0200 mmol, 0.1 equiv.), DABCO (ranging from 0 to 44.9 mg, 0 to 0.400 mmol, 0 to 2.0 equiv.), and either methyldiethoxysilane (MDES) (53.7 mg, 64.1 µL, 0.400 mmol, 2 equiv.) or 1,1,3,3 tetramethyldisiloxane (TMDS) (53.7mg, 70.7 µL, 0.400 mmol, 2 equiv.) additive were added and dissolved in anhydrous THF (0.8 mL). **1** (32.0 mg, 24 µL, 0.200 mmol, 1 equiv.) was added to the vial and the reaction was run for 24 hours with stirring at either room temperature or 50 °C. Trifluorotoluene (9.7 mg, 8.2 µL, 0.067 mmol, 0.33 eq) was used as an internal ^{19}F NMR standard and the **^{19}F NMR Yield Method** was used to calculate the percent yield and excess percent yield due to $Ca(NTf_2)_2$ (Table S3a).

Some reactions were left in their NMR tubes at room temperature and scanned after several days using the **^{19}F NMR Yield Method**. The yields of these reactions, and the extended time after 24 hours were also reported (Table S3b).

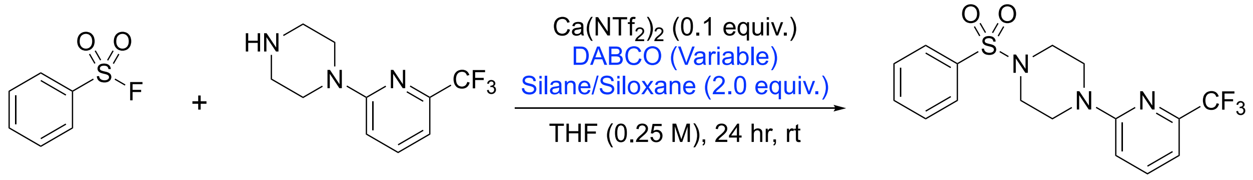

1
(1.0 equiv.)

3
(1.05 equiv.)

Sulfonamide **21**

Scheme 3. Silanes/Siloxanes with Secondary Amine **3** Reaction

Table S3a. ^{19}F NMR Yields of $PhSO_2F$ and Piperazine **2** Reaction

**Condition/Additive**	**% Yield***	**Excess % Yield Due to $Ca(NTf_2)_2$**
No $Ca(NTf_2)_2$ Controls		
0.2 equiv. DABCO	Trace[a]	-
0.5 equiv. DABCO	1	-
1.0 equiv. DABCO	Trace[a]	-
1.5 equiv. DABCO	Trace[a]	-
2.0 equiv. DABCO	Trace[a]	-
0.2 equiv. DABCO + 50 °C	1	-
0.2 equiv. DABCO + MDES	Trace[a]	-
0.5 equiv. DABCO + MDES	3[b]	-
0.2 equiv. DABCO + MDES + 50 °C	2[b]	-
0.2 equiv. DABCO + TMDS	Trace[a]	-
0.2 equiv. DABCO + TMDS + 50 °C	Trace[a]	-
Reactions with $Ca(NTf_2)_2$		
0.0 equiv. DABCO	22	-
0.2 equiv. DABCO	35	35
0.5 equiv. DABCO	39	39
1.0 equiv. DABCO	41	40
1.5 equiv. DABCO	42	42
2.0 equiv. DABCO	46	46
0.2 equiv. DABCO + 50 °C	65	64
0.0 equiv. DABCO + MDES	28	-
0.2 equiv. DABCO + MDES	36	36
0.5 equiv. DABCO + MDES	44	41
0.2 equiv. DABCO + MDES + 50 °C	64	62
0.2 equiv. DABCO + TMDS	35	35
0.2 equiv. DABCO + TMDS + 50 °C	71	71

[a] < 1% yield
[b] Yield averaged over duplicate runs
*Unless otherwise stated, trials without $Ca(NTf_2)_2$ were conducted in singlet and trials with $Ca(NTf_2)_2$ were conducted in duplicate, with averaged yields reported.

Table S3b. ^{19}F NMR Yields of Piperazine Derivative Reaction Left in NMR Tube

**Condition/Additive**	**% Yield**	**Excess % Yield Due to $Ca(NTf_2)_2$**
No $Ca(NTf_2)_2$ Controls		
0.2 equiv. DABCO + TMDS + 2 days rt	Trace[a]	-
0.2 equiv. DABCO + TMDS + 50 °C + 2 days rt	Trace[a]	-
Reactions with $Ca(NTf_2)_2$		
0.2 equiv. DABCO + TMDS + 2 days rt	61	61
0.2 equiv. DABCO + TMDS + 50 °C + 4 days rt	82[b]	82

[a] < 1% yield

[b] Yield averaged over duplicate runs

Replacing DABCO with Alternative Nitrogenous Bases

To a 3.7 mL (1 dram) scintillating glass vial **3** (48.6 mg, 0.210 mmol, 1.05 equiv.), $Ca(NTf_2)_2$ (12.0 mg, 0.0200 mmol, 0.1 equiv.), and N,N-diisopropylethylamine (DIPEA) (5.2 – 51.7 mg, 0.040 – 0.400 mmol, 0.2 – 2.0 equiv.) were added and dissolved in anhydrous THF (0.8 mL). **1** (32.0 mg, 24 µL, 0.200 mmol, 1 equiv.) was added to the vial and the reaction was run for 24 hours with stirring at 50°C. Trifluorotoluene (9.74 mg, 8.2 µL, 0.067 mmol, 0.33 eq) was used as an internal ^{19}F NMR standard and the **^{19}F NMR Yield Method** was used to calculate the percent yield and excess % yield due to $Ca(NTf_2)_2$ (Table S4).

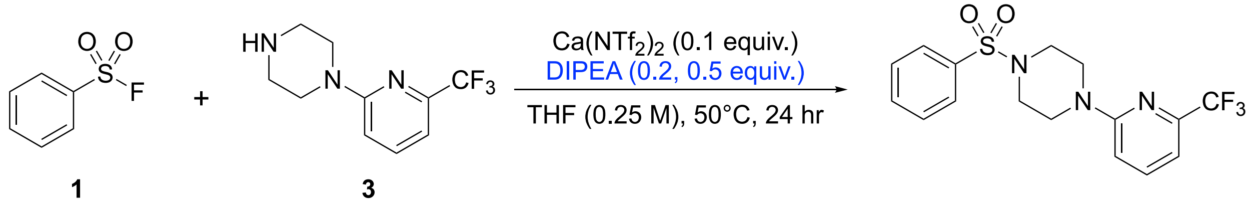

1
(1.0 equiv.)

3
(1.05 equiv.)

Sulfonamide **21**

Scheme 4. Hunig's Base with Catalytic Conditions

Table S4. ^{19}F NMR Yields of Piperazine Derivative Reaction with DIPEA

Condition/Additive	% Yield*	Excess % Yield Due to $Ca(NTf_2)_2$
No $Ca(NTf_2)_2$ Controls		
0.2 equiv. DIPEA	Trace[a]	-
0.5 equiv. DIPEA	Trace[a]	-
1.0 equiv. DIPEA	Trace[a]	-
1.5 equiv. DIPEA	Trace[a]	-
2.0 equiv. DIPEA	Trace[a]	-
Reactions with $Ca(NTf_2)_2$		
0.2 equiv. DIPEA	47	47
0.5 equiv. DIPEA	54	54
1.0 equiv. DIPEA	45	45
1.5 equiv. DIPEA	44	44
2.0 equiv. DIPEA	44	44

[a] < 1% yield

*Trials without $Ca(NTf_2)_2$ were conducted in singlet and trials with $Ca(NTf_2)_2$ were conducted in duplicate, with averaged yields reported.

Additional amine bases were also screened using the catalytic conditions with siloxanes. To a 3.7 mL (1 dram) scintillating glass vial **3** (48.6 mg, 0.210 mmol, 1.05 equiv.), $Ca(NTf_2)_2$ (12.0 mg, 0.0200 mmol, 0.1 equiv.), an amine base (0.2 equiv.) and 1,1,3,3 tetramethyldisiloxane (TMDS) (53.7mg, 70.7 µL, 0.400 mmol, 2 equiv.) were added and dissolved in anhydrous THF (0.8 mL). **1** (32.0 mg, 24 µL, 0.200 mmol, 1 equiv.) was added to the vial and the reaction was run for 24 hours with stirring at 50°C. Trifluorotoluene (9.74 mg, 8.2 µL, 0.067 mmol, 0.33 eq) was used as an internal ^{19}F NMR standard and the **^{19}F NMR Yield Method** was used to calculate the percent yield and excess % yield due to $Ca(NTf_2)_2$ (Table S5). It should be noted that the formation of side products was not analyzed and the consumption of sulfonyl fluoride was assumed to represent the reaction yields.

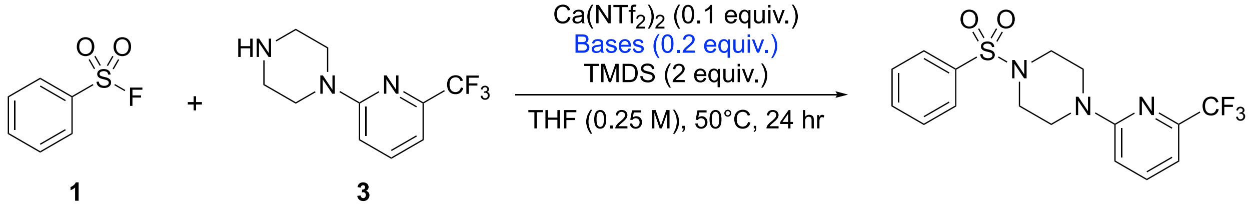

1
(1.0 equiv.)

3
(1.05 equiv.)

Sulfonamide **21**

Scheme 5. Alternative Amine Bases with Catalytic Conditions

Table S5. ^{19}F NMR Yields of Piperazine Derivative Reaction with Alternative Amine Bases

**Condition/Additive**	**% Yield of 21***	**Excess % Yield Due to $Ca(NTf_2)_2$**
No $Ca(NTf_2)_2$ Controls		
DABCO	Trace[a]	-
Triethylamine	Trace[a]	-
1,1,3,3-Tetramethylguanine	Trace[a]	-
Imidazole	Trace[a]	-
1,8-Diazabicyclo 5.4.0 undec-7-ene (DBU)	3	-
Reactions with $Ca(NTf_2)_2$		
DABCO	71	71
Triethylamine	59	59
1,1,3,3-Tetramethylguanine	23	23
Imidazole	64	64
1,8-Diazabicyclo 5.4.0 undec-7-ene (DBU)	29	26

[a] < 1% yield

*Trials without $Ca(NTf_2)_2$ were conducted in single runs and trials with $Ca(NTf_2)_2$ were conducted in duplicate, with averaged yields reported.

Catalytic Conditions with Diverse Sulfonyl Fluorides and Amines

To a 3.7 mL (1 dram) scintillating glass vial the respective amine (0.210 mmol, 1.05 equiv), $Ca(NTf_2)_2$ (12.0 mg, 0.020 mmol, 0.1 equiv.), DABCO (4.5 mg, 0.040 mmol, 0.2 equiv.), and 1,1,3,3 tetramethyldisiloxane (TMDS) (53.7mg, 70.7 µL, 0.400 mmol, 2 equiv.) additive were added and dissolved in anhydrous THF (0.8 mL). The respective sulfonyl fluoride (0.200 mmol, 1 equiv.) was added to the vial and the reaction was run for 24 hours with stirring at 50 °C. Control reactions, without $Ca(NTf_2)_2$, were also performed. For experiments analyzed using NMR yields, trifluorotoluene (9.74 mg, 8.2 µL, 0.067 mmol, 0.33 eq) was used as an internal ^{19}F NMR standard and the **^{19}F NMR Yield Method** was used to calculate the percent yield and excess percent yield due to $Ca(NTf_2)_2$ (Figure S5). For experiments where the products were isolated, the products were prepared using the **General Procedure for Synthesizing and Isolating Sulfonamides 21-24**.

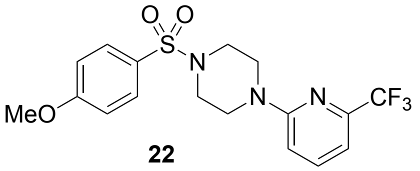

22

51%[a]
7%[b]
66%[c]
trace[d]

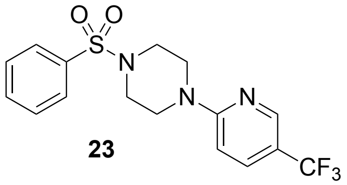

23

53%[a]
trace[b]

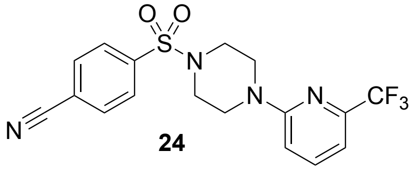

24

67%[a]
24%[b]
79%[c]
31%[d]

a. Isolated Yield
b. Isolated Yield, No $Ca(NTf_2)_2$
c. NMR Yield
d. NMR Yield, No $Ca(NTf_2)_2$

Figure S5. Additional Substrates and Yields with Catalytic Conditions

References

[1] J. S. Cannon, L. Zou, P. Liu, Y. Lan, D. J. O'Leary, K. N. Houk, R. H. Grubbs, *J. Am. Chem. Soc.* **2014**, *136*, 6733–6743.

[2] S. Mahapatra, C. P. Woroch, T. W. Butler, S. N. Carneiro, S. C. Kwan, S. R. Khasnavis, J. Gu, J. K. Dutra, B. C. Vetelino, J. Bellenger, C. W. am Ende, N. D. Ball, *Org. Lett.* **2020**, *22*, 4389–4394.

NMR Spectra of Compounds 21-24

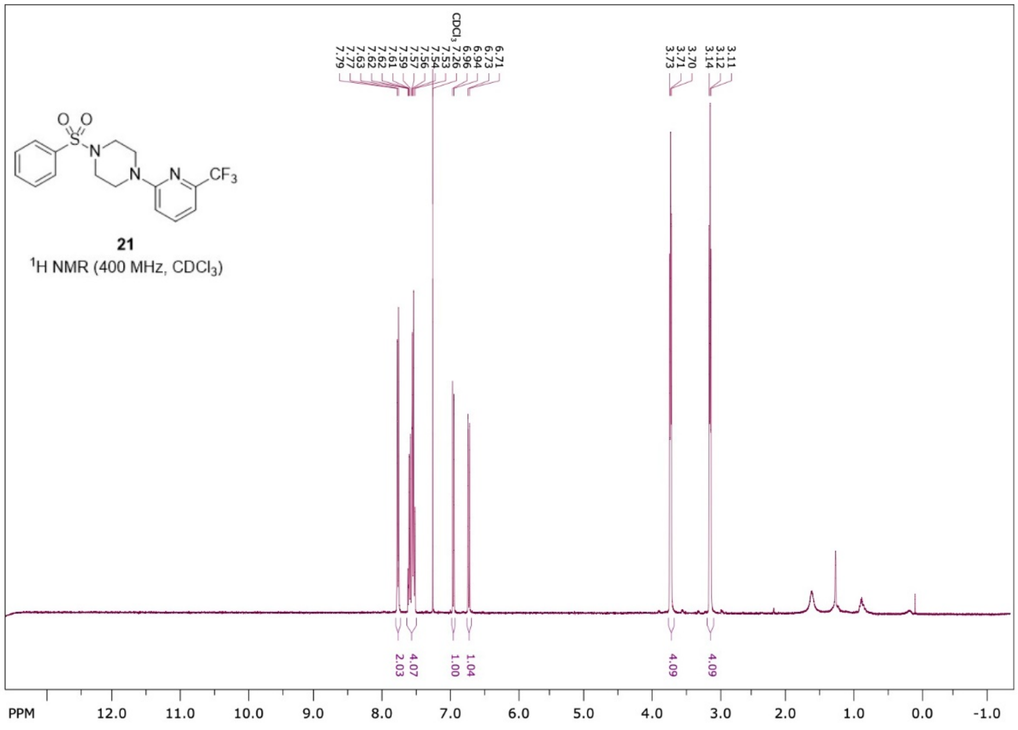

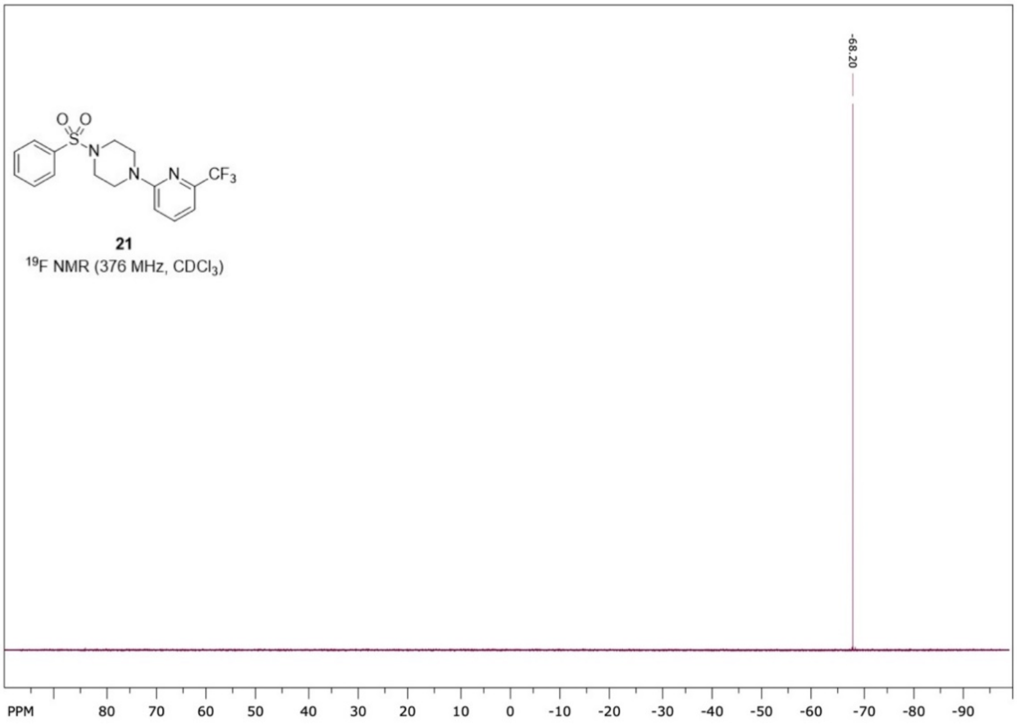

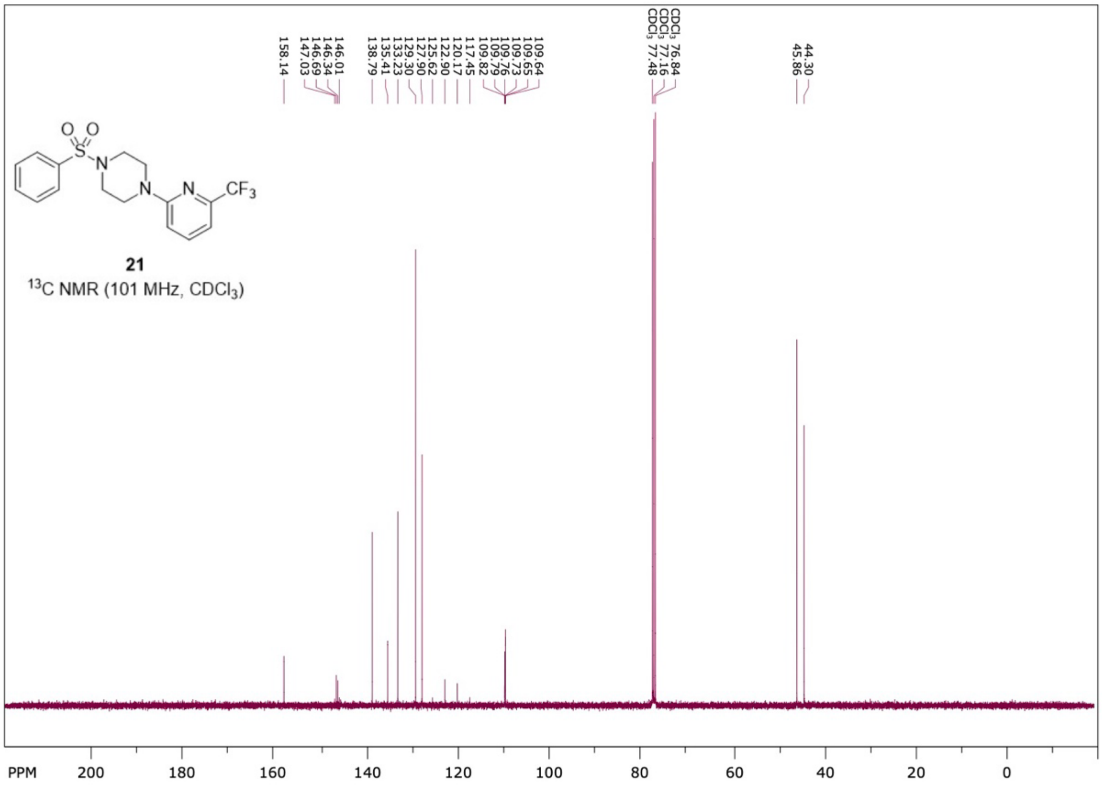

21
^{13}C NMR (101 MHz, CDCl$_3$)
PPM

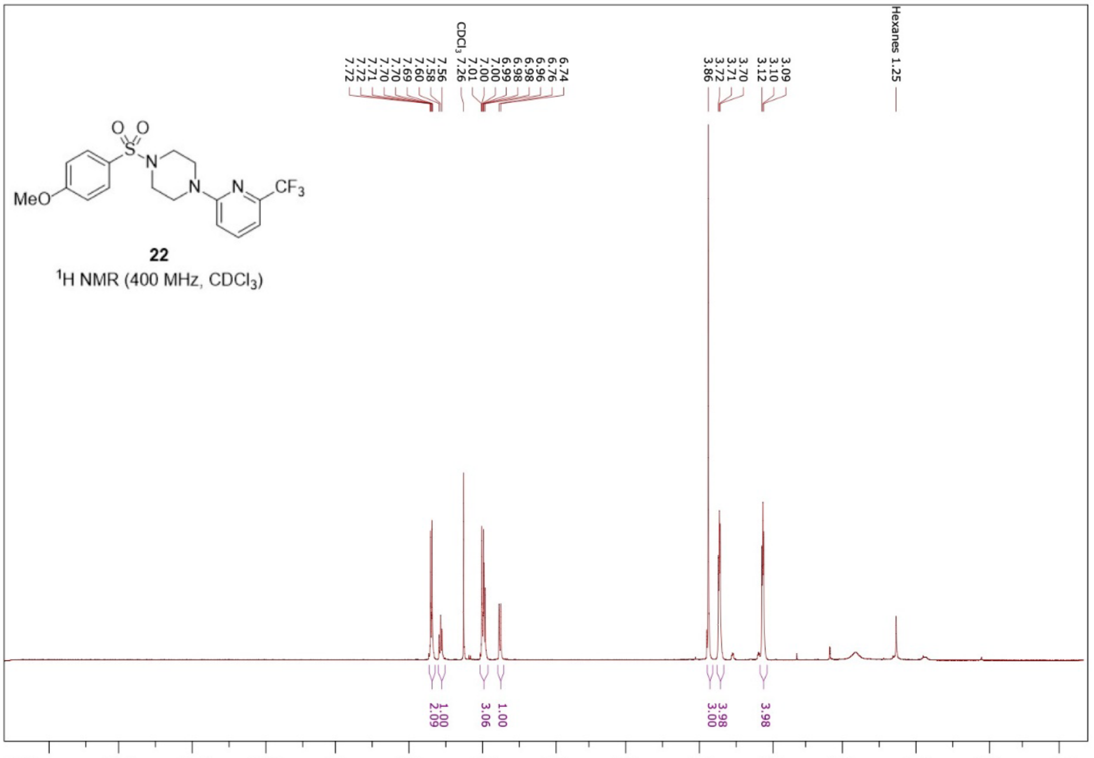

MeO
22
^{1}H NMR (400 MHz, CDCl$_3$)
Hexanes 1.25
PPM

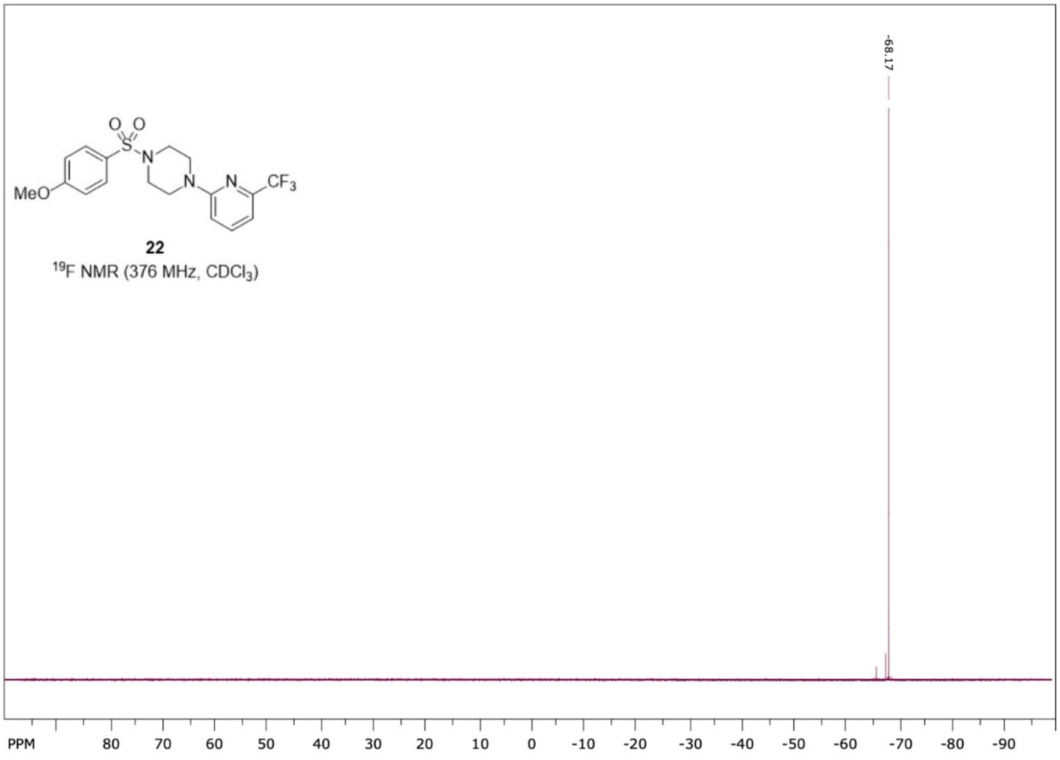

22
^{19}F NMR (376 MHz, $CDCl_3$)

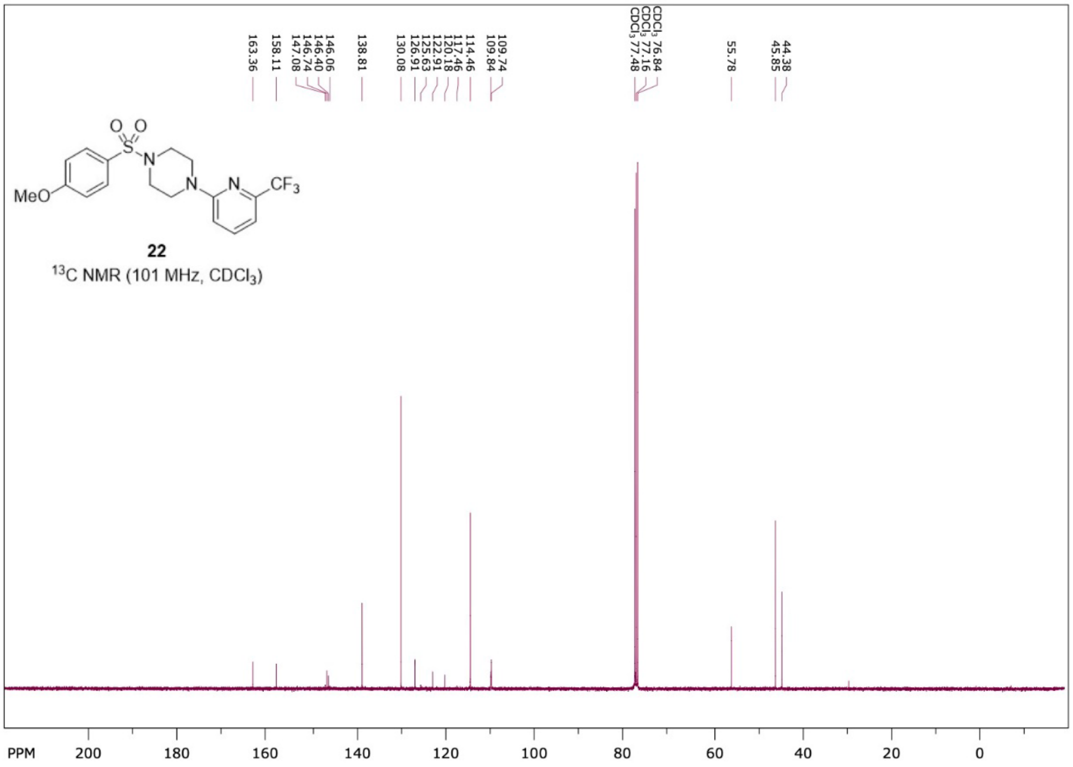

22
^{13}C NMR (101 MHz, $CDCl_3$)

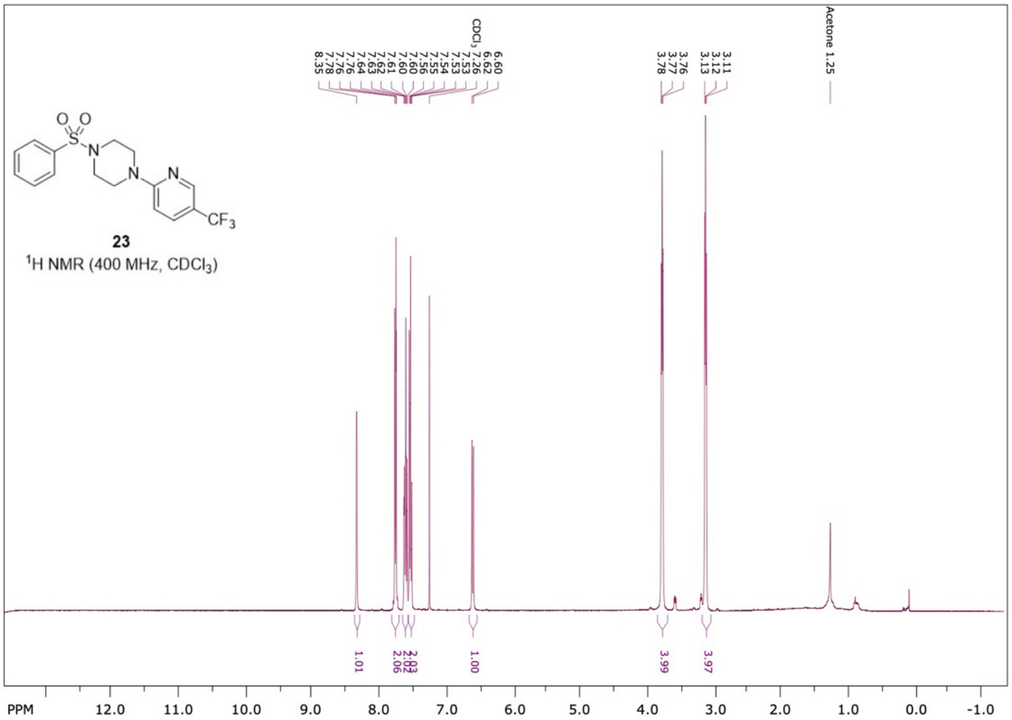

23

^{1}H NMR (400 MHz, $CDCl_3$)

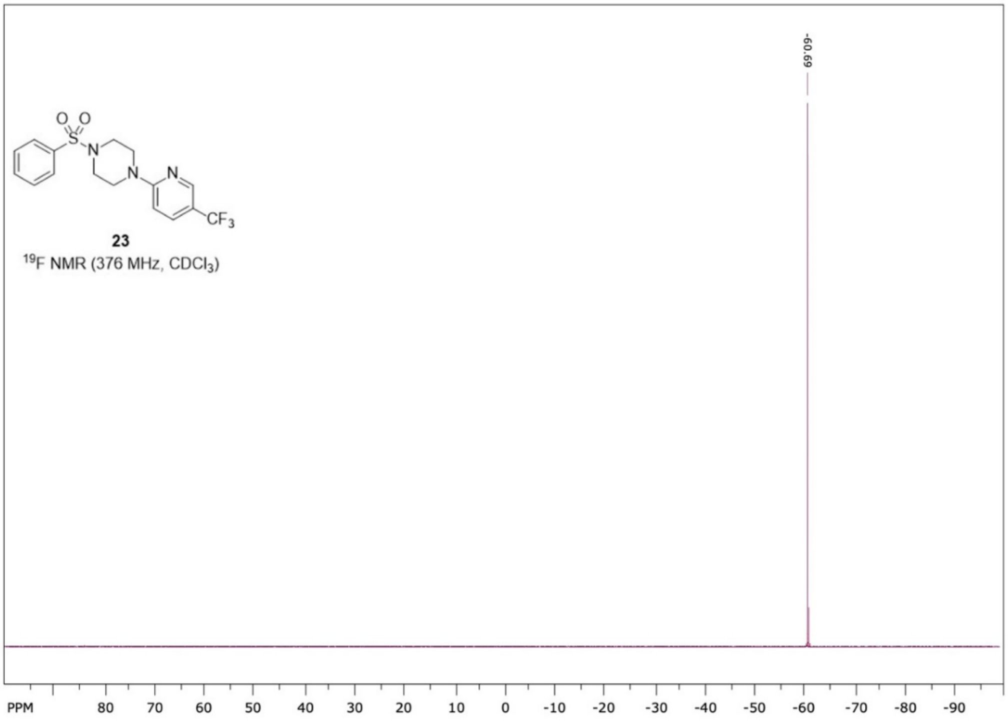

23

^{19}F NMR (376 MHz, $CDCl_3$)

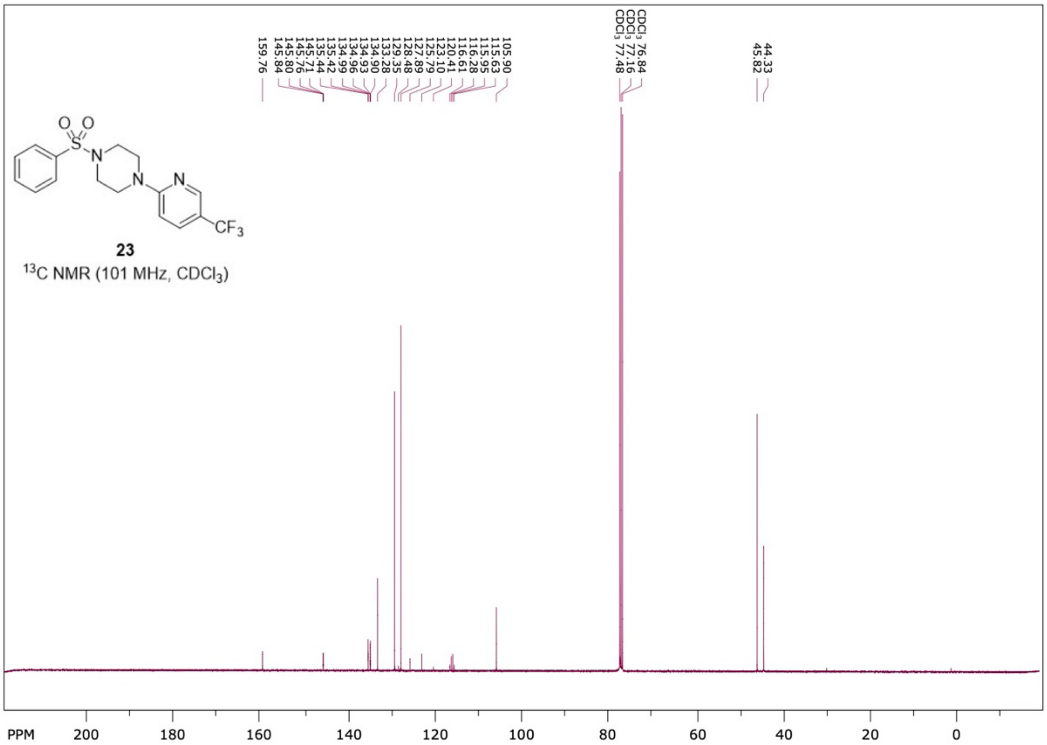

159.76
145.84
145.80
145.76
145.71
135.44
135.42
134.99
134.96
134.93
134.90
133.28
129.35
128.48
127.89
125.79
123.10
120.41
116.61
116.28
115.95
115.63
105.90
CDCl3 76.84
CDCl3 77.16
CDCl3 77.48
44.33
45.82
23
13C NMR (101 MHz, CDCl3)
PPM
200
180
160
140
120
100
80
60
40
20
0

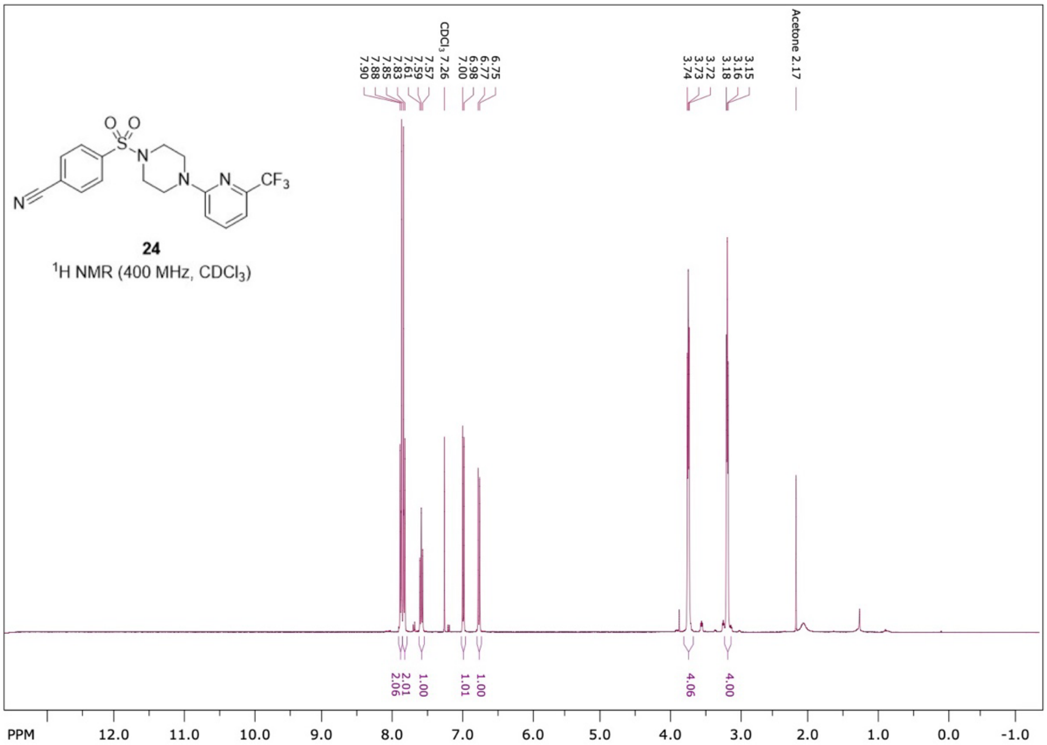

7.90
7.88
7.85
7.83
7.61
7.59
7.57
CDCl3 7.26
7.00
6.98
6.77
6.75
3.74
3.73
3.72
3.18
3.16
3.15
Acetone 2.17
24
1H NMR (400 MHz, CDCl3)
2.06
2.01
1.00
1.01
1.00
4.06
4.00
PPM
12.0
11.0
10.0
9.0
8.0
7.0
6.0
5.0
4.0
3.0
2.0
1.0
0.0
-1.0

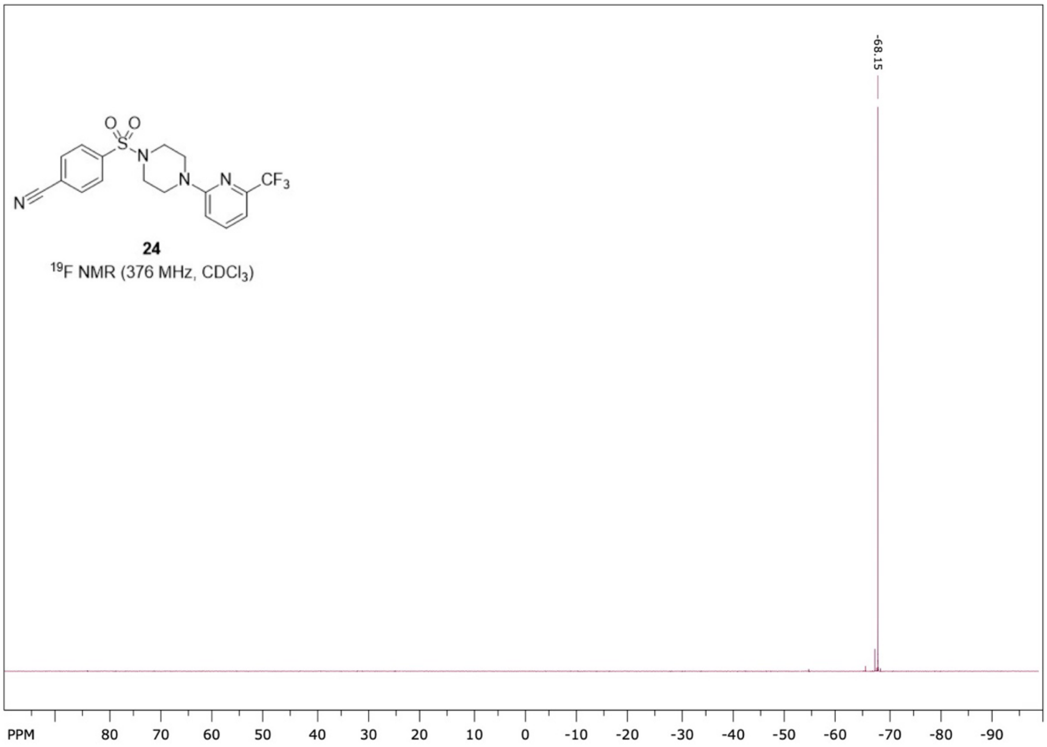
24
^{19}F NMR (376 MHz, $CDCl_3$)
-68.15
PPM 80 70 60 50 40 30 20 10 0 -10 -20 -30 -40 -50 -60 -70 -80 -90

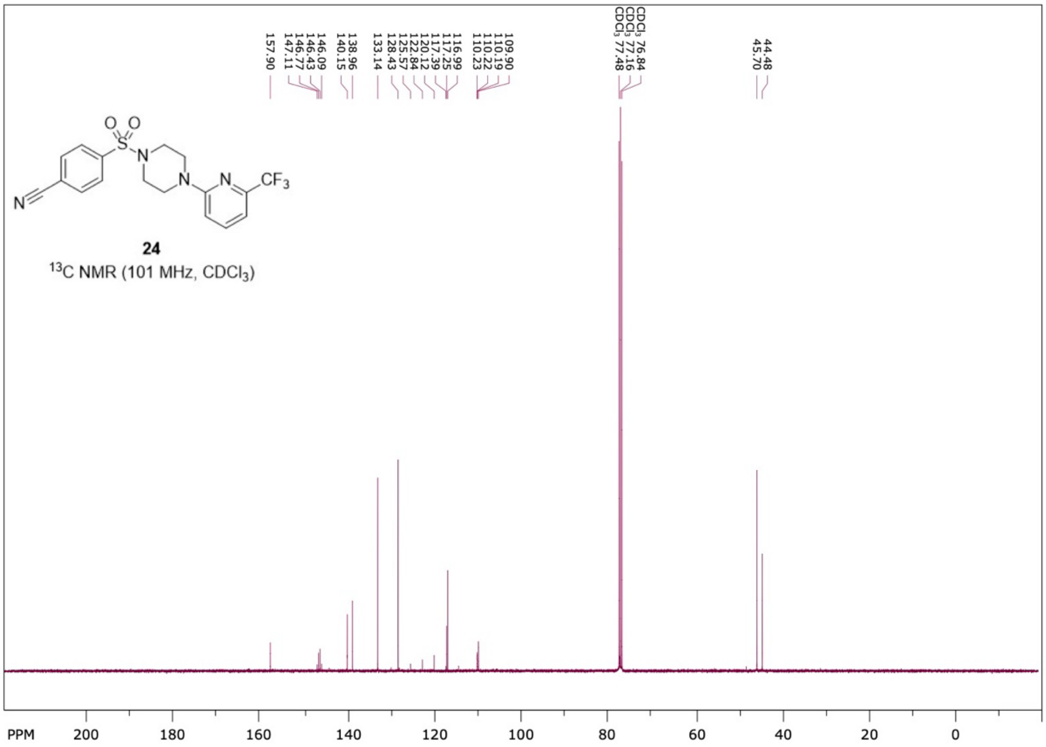
24
^{13}C NMR (101 MHz, $CDCl_3$)
157.90
147.11
146.77
146.43
146.09
140.15
138.96
133.14
128.43
125.57
122.84
120.12
117.39
117.25
116.99
110.23
110.22
110.19
109.90
$CDCl_3$ 77.48
$CDCl_3$ 77.16
$CDCl_3$ 76.84
45.70
44.48
PPM 200 180 160 140 120 100 80 60 40 20 0
